# Functional Al/Cd Heterometallics—From Controlled Al(I) Transfer to Nucleophilic Transfer of Cadmium Ions

**DOI:** 10.1021/jacs.5c12746

**Published:** 2025-08-25

**Authors:** Dominic Herle, Sara Sommer, Fabian Dankert

**Affiliations:** Institute of Chemistry, 9178 University of Kassel, Heinrich-Plett-Str. 40, Kassel 34132, Germany

## Abstract

Low-valent cadmium compounds have remained largely unexplored as electron reservoirs, with no precedent for their use in reduction or bond activation chemistry. Here, we address this gap by integrating low-valent aluminum into the cadmium coordination sphere. Aluminylene insertion into Cd­{N­(TMS)_2_}_2_ affords bi- and trimetallic cadmium aluminyls **1** and **2**, featuring covalent yet tunable Al–Cd bonding. While **1** is irreversibly formed, **2** exhibits dynamic reactivity, enabling reversible [AlCp*]^0^ transfer—a rare and previously undocumented feature in heterobimetallic aluminum chemistry. Acting as a thermodynamically stable yet chemically reactive surrogate for free Al­(I), **2** enables selective Al­(I) shuttling to B, Cd, Zn, and Ag substrates (compounds **1** ^**Zn**^, **3**–**5**). The cooperative Al–Cd framework in **1** and **2** further promotes bond activation of heterocumulenes such as carbodiimides and CO_2_, with **1** serving as a source of nucleophilic cadmium—an unprecedented reactivity mode for this element (compounds **6**–**8**). Detailed quantum chemical calculations elucidate the electronic structures of **1** and **2** as well as the mechanism for both Al­(I) transfer and heterocumulene insertion. These findings establish a platform for bimetallic cooperativity in small-molecule activation and lay the groundwork for Al­(I)-based multimetallic systems relevant to future catalysis and main group/transition-metal synergy.

## Graphical abstract

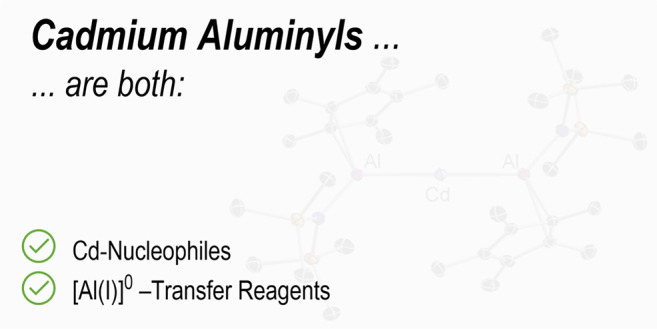

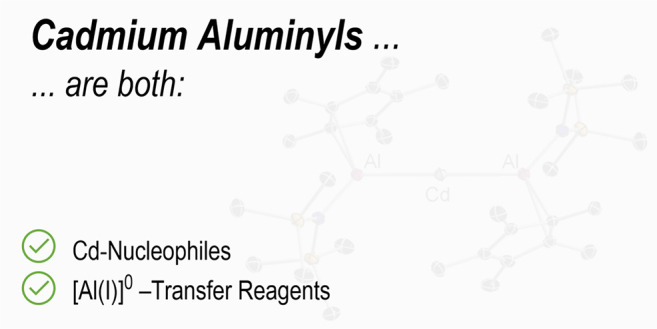

## Introduction

Cadmium, best known for its role in Ni/Cd batteries, is chemically akin to zinc and typically exhibits Lewis acidic behavior in its common +2 oxidation state. As a soft Lewis acid, Cd­(II) readily forms a wide variety of coordination complexes with nitrogen, oxygen, and sulfur donor ligands. Contemporary applications of cadmium coordination compounds span a broad spectrum. These include the construction of nanostructures,^1–12^ Zintl phases,^13–18^ and metal-rich clusters,^19,20^ as well as MOF-based sensing platforms,^21–26^ C–H cadmation of arenes,^27,28^ and hydrocadmation reactions with cadmium hydrides.^29^ Yet, in stark contrast to its wide utility in coordination chemistry, the reactivity of low-valent cadmium species remains underexplored. Compounds based on the [Cd_2_]^2+^ dication, including “inorganic” species **I** ^30,31^ and cluster **II** ^32,33^, represent seminal examples of low-valent cadmium, structurally reminiscent of the well-known [Hg_2_]^2^^+^ core (Scheme 1). These species have paved the way for the isolation of hydrocarbon soluble low-valent cadmium compounds (e.g., **III** ^34–37^ and **IV** ^38^), but their synthetic and reactive potential remains largely untapped.^39^ This contrasts with the well-established role of [Zn_2_]^2+^ species, as originally pioneered by *Carmona*,^40^ in small molecule activation, metal–metal bond transfer and intriguing reductive addition chemistry.^41,42^ Moreover, cadmium’s comparison to the alkaline earth metals, whose low-valent (+1) chemistry has seen rapid progress,^43–45^ suggests promising parallels that have yet to be realized. Notably, aluminum-based metallo-ligands are emerging as powerful tools for constructing heterobimetallic platforms capable of synergistic bond activation.^46–48^ Valence isoelectronic Al^*X*^–M frameworks involving main group- (s- ^49–61^ and p-block^62–64^ ), transition- (early,^65–67^ mid^68–71^ and late^68–82^ ) and even f-*block*-metals^83^ unlocked pathways for a variety of challenging chemical transformations.

**Scheme 1:**
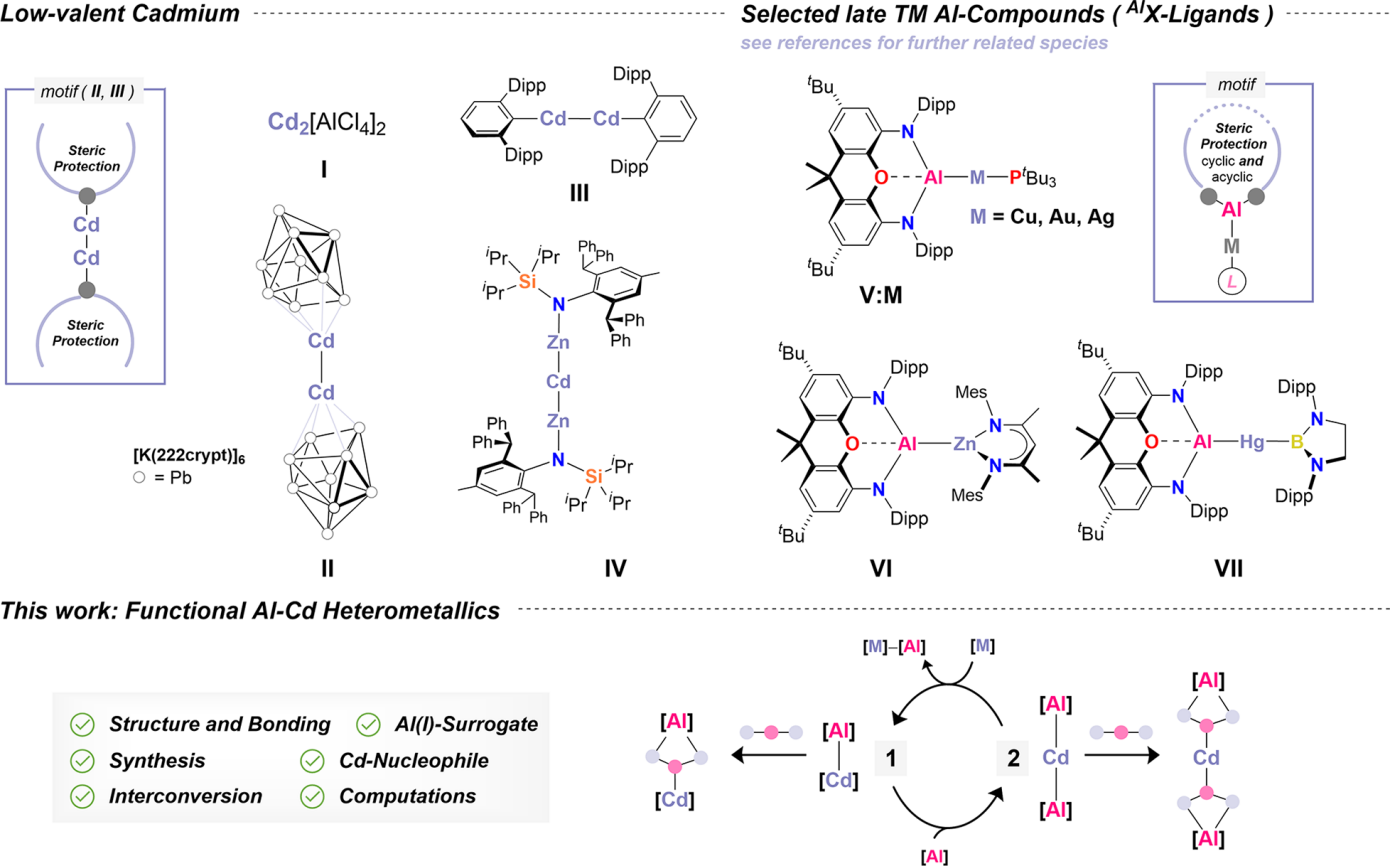
Selected Cadmium^30–38^ and Aluminum^73,77,78,84^ Homo– and Heterobimetallics Including an Outline of this Study

Central to these advances is the availability of kinetically stabilized Al­(I) compounds, such as *Aldridge*’s and *Goicoechea*’s landmark compound K_2_[Al­(NON)]_2_ (NON = 4,5-bis­(2,6-diisopropylanilido)-2,7-di-*tert*-butyl-9,9-dimethylxanthene), which serves as a potent metallo-ligand and nucleophilic Al­(I) transfer reagent.^49^ Such a transfer reagent has enabled the formation of unique Al–M bonds including Al–Au (i.e., **V:Au**; “nucleophilic gold”),^73^ Al–Cu (i.e., **V:Cu**),^77,78^ Al–Ag (i.e., **V:Ag**),^77^ Al–Zn (i.e., **VI**),^80^ and Al–Hg (**VII**).^84^ Each are displaying cooperative reactivity except **VII** due to instability.^84^ Somewhat consistent with this instability, cadmium remains absent from this series, despite early indications of promising metal–metal bonding scenarios in low-valent Cd compounds. Reports of Cd complexes with main group elements such as magnesium,^85^ gallium,^86,87^ indium,^88^ and tin^89,90^ suggest feasibility, yet merging aluminum and cadmium was not realized yet. Herein, we address this gap by introducing authentic covalent Al–Cd bonds, formed *via* insertion of an aluminylene ligand into cadmium amides. This strategy affords a series of bi- and trimetallic Al/Cd species that interconvert reversibly, transfer Al­(I) and ultimately promoted cadmium as a nucleophilic reductant harnessing a suitable heterobimetallic platform. Computational analyses reveal key insights into electronic structure and explain transfer processes at cadmium.

## Results and Discussion

To introduce low-valent aluminum to a cadmium center, we explored a convenient synthetic route. As the Al­(I) source, we employed the robust aluminylene [AlCp*]_4_. Reaction of Cd­{N­(TMS)_2_}_2_ with a slight excess of monomeric [AlCp*] in C_6_D_6_ yielded a clear, metallic-gray solution. The ^1^H NMR spectrum indicated selective and quantitative formation of a new species, with resonances at 1.92, 0.31, and 0.21 ppm. After workup, the cadmium aluminyl (also referred to as “alumanyl”) [({N­(TMS)_2_})­(Cp*)­Al–Cd­({N­(TMS)_2_})] (**1**) was obtained in 87% yield. Colorless single crystals suitable for X-ray diffraction were isolated, and the molecular structure was determined. Compound **1** features a nearly linear cadmium center (∠Al1–Cd1–N1 = 174.4(1)°) and an Al1–Cd1 bond length of 2.528(2) Å—slightly shorter than the sum of the covalent radii (Σ*r*_cov_(Al–Cd) = 2.62 Å).^91^ The Cp* ligand binds to aluminum *via* η^2^-coordination in the solid state, with the closest Al–C distances measuring 2.147(8) and 2.178(8) Å. In solution, however, rapid exchange of Cp* coordination sites results in the observation of a single resonance, even at –40 °C.

While isolated **1** is prone to decompose slowly in crystalline form, it is extremely stable in solution. Heating a solution of **1** at 75 °C for over 24 h results in no significant decomposition (Figure S74). Reaction of isolated **1**, or Cd­{N­(TMS)_2_}_2_, with one or two equivalents of [AlCp*] yields the trimetallic complex [{N­(TMS)_2_}­(Cp*)­Al]_2_Cd (**2**) in 83% yield after workup. Its formation is marked by a characteristic yellow color (Scheme 2). ^1^H NMR spectroscopy of **2** shows two new resonances corresponding to the Cp* ligands and [{N­(TMS)_2_}]^−^ groups at 2.06 and 0.30 ppm. Like **1**, compound **2** displays high thermal stability (Figure S78). The UV/vis electronic absorption spectrum of **2** in benzene reveals absorption in the blue-to-green region, attributed to HOMO→LUMO and HOMO→LUMO+1 transitions (σ_*AlCd*_→π_*AlCd*_; Figures S21, S99–S102). Single-crystal X-ray diffraction confirms the trimetallic core structure. The cadmium center retains linear coordination (∠Al1–Cd1–Al1’ = 180°), and the Cd1–Al1 bond length is slightly elongated at 2.588(2) Å compared to **1** (2.528(2) Å), likely due to steric effects. This is supported by elongated Al–C distances to the η^2^-bound Cp* ligands (Al1–C7: 2.182(7) Å; Al1–C8: 2.210(8) Å), indicating increased repulsion from the flanking groups. The rapid formation of **1** and **2** observed experimentally is supported by computational analysis. Insertion pathways were investigated at the ZORA-DLPNO–CCSD­(T) CPCM­(C_6_H_6_)/ZORA-def2-TZVPP­{SARC-ZORA-TZVPP­(Cd)}//r^2^SCAN-3c level of theory. Barrierless coordination of monomeric AlCp* initially yields intermediate **IM1** (Δ_R_ *G* = +14 kJ mol^–1^). Insertion of AlCp* into one Cd–N bond proceeds *via* transition state **TS1** (Δ_R_ *G* ^‡^ = +41 kJ mol^–1^), forming **1** with a clear favorable reaction energy (Δ_R_ *G* = –91 kJ mol^–1^). Similarly, AlCp* coordination to **1** is also barrierless, leading to intermediate **IM2** (Δ_R_ *G* = –65 kJ mol^–1^), which undergoes insertion through **TS2** (Δ_R_ *G* ^‡^ = +47 kJ mol^–1^) to form **2** (Δ_R_ *G* = –146 kJ mol^–1^). Notably, this second AlCp* transfer is energetically favorable (ΔΔ_R_ *G* = +57 kJ mol^–1^). Crucially, this energy gain is 34 kJ mol^–1^ lower in energy than the first Al­(I) transfer to the Cd­{N­(TMS)_2_}_2_ precursor, though. Intrinsic bond orbital (IBO) analysis (Scheme 2c, r^2^SCAN-3c; major HOMO components shown in Figure S95) reveals distinct electronic features for **1** and **2**. In **1**, the Al–Cd bond exhibits nearly equal partial charge distributions—q_σ‑IBO_(Al, Cd) = (0.95, 0.94)—indicative of near-ideal electron sharing in a covalent two-center two-electron bond (net IAO charges: see Figure S95: top). The deviation from an idealized distribution (1.00, 1.00) is minimal. In contrast, **2** displays significantly polarized Al–Cd bonds, with q_σ‑IBO_(Al, Cd) values of (1.18, 0.68) and (1.18, 0.69), suggesting increased localization toward the aluminum centers (net IAO charges: see Figure S95: bottom). While still consistent with electron-sharing (“X-type”)^92^ ligands for the [({N­(TMS)_2_})­Cp*Al]^−^ fragments and cadmium, the Al–Cd bonds in **2** are more activated than in **1**. This enhanced activation is reflected in the electronic structure. Calculated frontier orbital energies show that the trimetallic core in **2** narrows the HOMO–LUMO gap by at least 0.5 eV (Figures S97 and S98; σ_*AlCd*_ and delocalized π_*AlCd*_), increasing the respective orbital accessibility and reactivity. Based on these calculations, we proposed that the second equivalent of [AlCp*] in **2** is transferable. Indeed, treatment of **2** with one equivalent of B­(C_6_F_5_)_3_ leads to near quantitative regeneration of **1**, accompanied by equimolar formation of the known compound [(Cp*)­Al–B­(C_6_F_5_)_3_] (**3**; Figures S59–S65).^93^ This AlCp* transfer from **2** proceeds *via* a formal activation barrier of only Δ_R_ *G* ^‡^ = (146–47) = +99 kJ mol^–1^, consistent with the experimental reactivity (Scheme 3). Similarly, reaction of **2** with a slight excess of Cd­{N­(TMS)_2_}_2_ enables Al­(I) transfer to generate two equivalents of **1** (Scheme 3, Figures S66). Transfer to the bulkier Cd­(TMP)_2_ system yields [(TMP)­(Cp*)­Al–Cd­(TMP)] (**4**) with 40% conversion, though a significant side product is observed (Figure S67). Compound **4** was isolated and structurally characterized by X-ray diffraction (Scheme 3, bottom left).

**Scheme 2:**
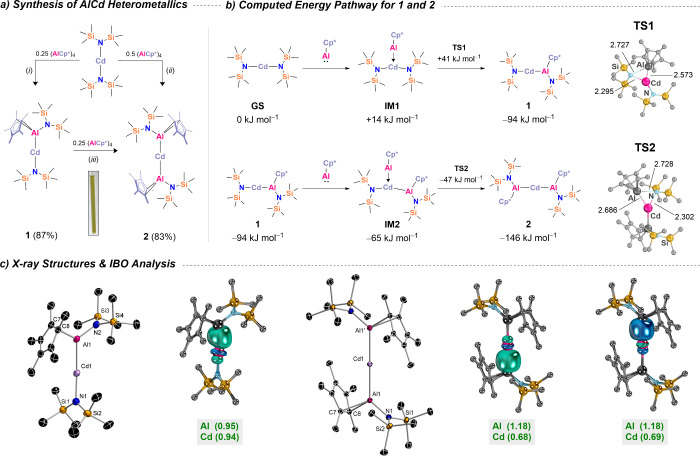
a) Synthetic Access; b) Gibbs Free Energy Pathway (Δ_R_ *G*) for the Generation of 1 and 2 at the ZORA-DLPNO–CCSD­(T) CPCM­(C_6_H_6_)/ZORA def2-TZVPP­{SARC-ZORA-TZVPP­(Cd)}//r^2^SCAN-3c Level of Theory Including a Depiction of Transition States; c) Determined Molecular Structures in the Crystal and Intrinsic Bond Orbitals (r^2^SCAN-3c) with IAO Partial Charge Distributions for a Respective Al–Cd Bond^a^

a Thermal ellipsoids at 50% probability. Selected bond lengths [Å] and angles [°] in **1**: Al1–Cd1 2.528(2), Al1–N2 1.824(6), Al1–C7 2.147(8), Al1–C8 2.178(8), Cd1–N1 2.094(6), Al1–Cd1–N1 174.4(1), **2**: Al1–Cd1 2.579(1), Al1–N1 1.844(2), Al1–C7 2.210(3), Al1–C8 2.177(3); Al1–Cd1–Al1’ 180. (*i*) benzene or toluene, RT→50 °C (sonication, 20 min.), (*ii*) benzene or toluene, RT→50 °C (sonication, 30 min.), (*iii*) benzene or toluene, RT→50 °C (sonication, 10 min.).

**Scheme 3:**
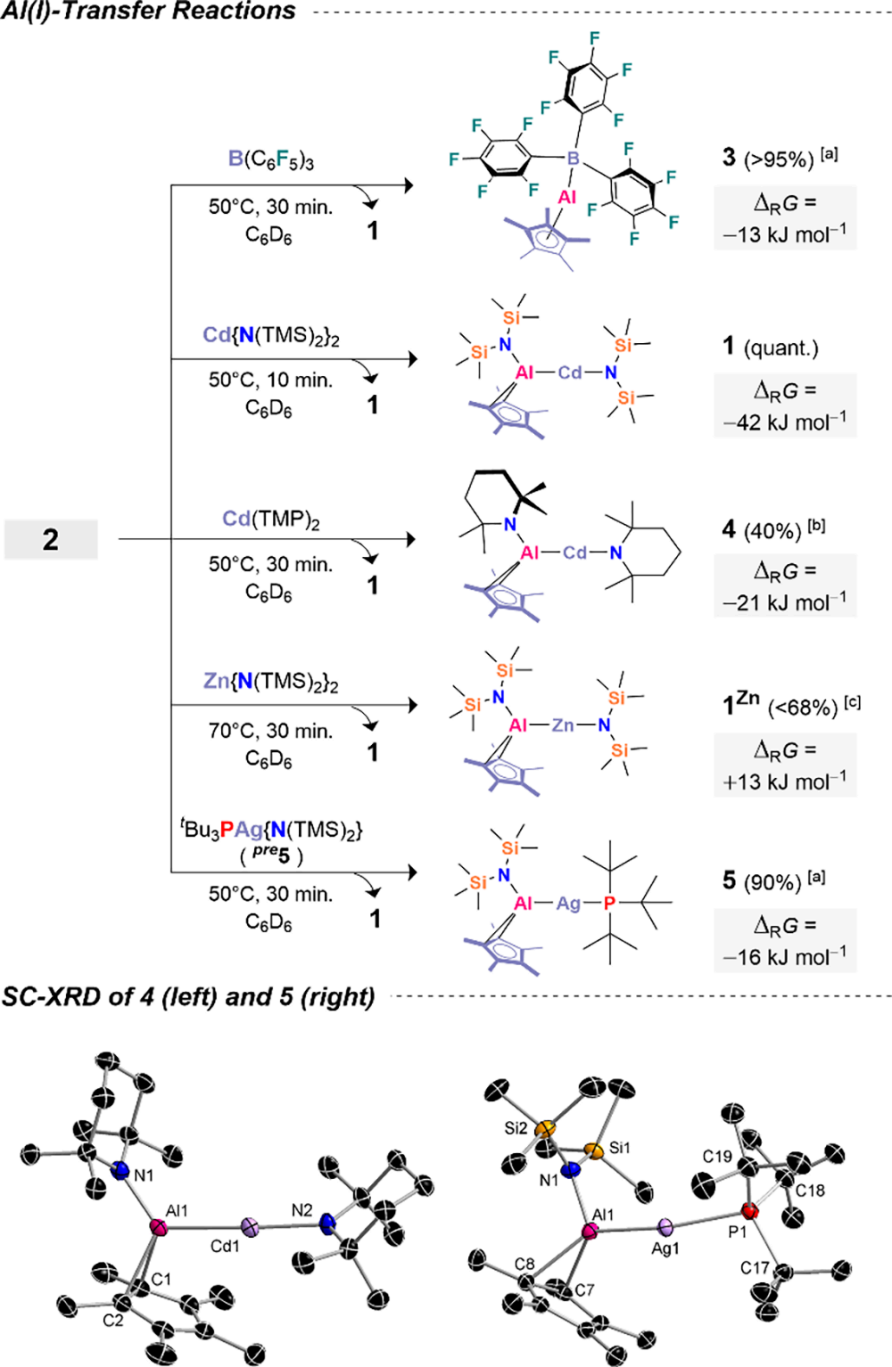
Al­(I)-Transfer Reactions Employing a Trimetallic Cadmium Aluminyl^a^

a Conversions determined with TMS or naphthalene as internal standard: ^[a]^ = selective conversion; ^[b]^ = accompanied by a major side product; ^[c]^ = maximum conversion at 3h with an excess of Zn­{N­(TMS)_2_}_2_. Gibbs free energies calculated at the ZORA-DLPNO–CCSD­(T) SMD­(C_6_H_6_)/ZORA def2-TZVPP­{SARC-ZORA-TZVPP­(Cd)}//r^2^SCAN-3c level of theory. Thermal ellipsoids at 50% probability. Selected bond lengths [Å] and angles [°] in **4**: Al1–Cd1 2.529(1), Al1–N1 1.824(3), Cd1–N2 2.067(2), Al1–Cd1–N2 173.72(8), N1–Al1–Cd1 123.8(1); **5**: Al1–Ag1 2.467(1), Ag1–P1 2.458(1), Al1–N1 1.872(3), Al1–C7 2.210(3), Al1–C8 2.226(3), Al1–Ag1–P1 166.10(3), N1–Al1–P1 114.01(8).

Like **1**, it features a near-linear Cd coordination environment (∠Al1–Cd1–N1 = 173.72(8)°), and the Al–Cd bond length is comparable to that in **1** within experimental error. We next explored whether [AlCp*]^0^ could be shuttled to metals beyond cadmium. First, Zn­{N­(TMS)_2_}_2_ was used to assess transferability across group 12. This resulted in formation of [{N­(TMS)_2_}­(Cp*)­Al–Zn­{N­(TMS)_2_}] (**1** ^**Zn**^),^81^ with up to 68% conversion after 3 h at 70 °C (Figure S68). Most notably, we extended this concept to a group 11 system: reaction of **2** with [{N­(TMS)_2_}­Ag­(P^*t*^Bu_3_)] (^***pre***^ **5**) furnished the silver complex [{N­(TMS)_2_}­(Cp*)­Al–Ag­(P^*t*^Bu_3_)] (**5**) in 90% overall conversion. To further substantiate [Al­(Cp*)]^0^ transfer from the cadmium scaffold, the solid-state structure of the silver aluminyl **5** was determined (Scheme 3, bottom). Overall, these results qualify **2** as a heteromultimetallic Al­(I) transfer agent. The transmetalation of Al­(I) with the various substrates can be rationalized in thermodynamic terms, as the calculated free energy changes are negative for all cases except **1** ^**Zn**^, which is slightly endergonic. We therefore propose that this particular transfer proceeds under kinetic control.

Separately, we investigated the reactivity of **1** and **2** to probe potential bimetallic cooperativity within their metal–metal architectures (Scheme 4). To assess whether **1** and **2** can act as sources of nucleophilic cadmium, we selected heterocumulenes, thereof specifically, carbodiimides and CO_2_, as test substrates. Reaction of **1** with one equivalent of either DIC or DCC afforded [{N­(TMS)_2_}­(Cp*)­Al­{(NR)_2_C}­Cd­{N­(TMS)_2_}] (R = ^*i*^Pr: **6a**; R = Cy: **6b**). NMR spectroscopy confirmed quantitative conversion in each case (see SI), and excellent isolated yields were obtained following workup. Most notably, in the ^13^C­{^1^H} NMR spectra, resonances for the central carbon atoms of the former carbodiimides appear around 196 ppm that is consistent with insertion into an Al–M heterometallic framework and the involvement of cadmium as a nucleophile. Reactions with carbodiimides have been widely employed to probe the polarity of Al–M (M = Au,^73^ Ag,^77^ Cu,^77,78^ Zn^80,81^ ) bonds,^75^ and NMR spectroscopy has proven to be a powerful tool in evidencing heterometallic nucleophilic behavior in such systems. Recrystallization from *n*-pentane afforded crystals suitable for X-ray diffraction (Scheme 4: b), unambiguously confirming the connectivities. The structure reveals a short Cd–C bond of 2.132(4) Å (cf. [(IPr)­Cd­(OTf)­(μ-I)]_2_; d­(Cd–C_carbene_) = 2.191(4) Å),^94^ along with a nearly linear coordination environment at cadmium (i.e., **6a**: ∠C1–Cd1–N3 = 177.1(2)°). Interestingly, the unreacted amide ligand on cadmium is susceptible to rapid insertion of a second equivalent of carbodiimide. Addition of two equivalents of a carbodiimide to a solution of isolated **1** results in the swift formation of mixed guanidinate–carbene complexes across cadmium, yielding [{N­(TMS)_2_}­(Cp*)­Al­{(NR)_2_C}­Cd­{(NR)_2_C–N­(TMS)_2_}] (R = ^*i*^Pr: **7a**; R = Cy: **7b**). In solution, two distinct ^13^C­{^1^H} NMR resonances are observed for the central carbon atoms of the inserted heterocumulenes.

**Scheme 4:**
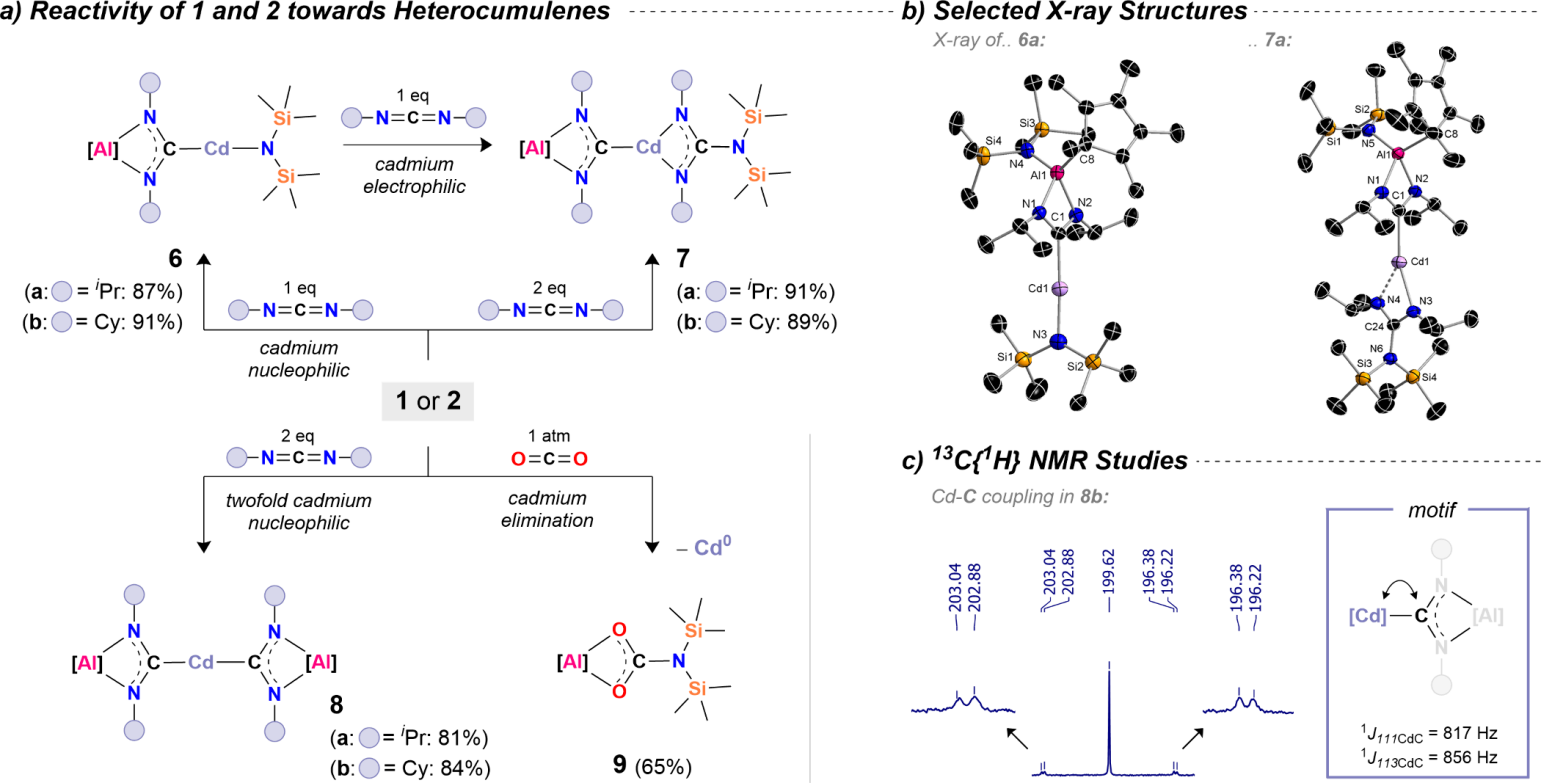
a) Reactivity Studies of the Al/Cd Heterometallics 1 and 2 toward Heterocumulenes, b) Molecular SC-XRD Structures of Selected Bond Activation Products and c) Illustrations of Cd–C Coupling Observed in ^13^C NMR.^a^

a All reactions performed in benzene or toluene (see SI for details). Thermal ellipsoids are given at 50% probability. For selected bond lengths [Å] and angles [°] in **6a**: Al1–N1 1.928(3), Al1–N2 1.952(4), Al1–N4 1.822(5), Al1–C8 2.041(5), C1–Cd1 2.132(4), Cd1–N3 2.026(4); C1–Cd1–N3 177.1(2); **7a**: Al1–N5 1.833(3), Al1–N1 1.932(3), Al1–N2 1.915(3), Al1–C8 2.049(4), Cd1–C1 2.135(3), Cd1–N3 2.181(3), Cd1–N4 2.202(3), N6–C24 1.421(4); C1–Cd1–N3 156.1(1), C1–Cd1–N4 143.0(1). For further structures and metrics, see figures S89–S93.

The carbene carbon atoms in **7** are characteristically downfield-shifted (∼202 ppm), whereas the central guanidinate carbon atoms resonate further upfield (∼168 ppm). The solid-state structure of **7a** (Scheme 4) shows a three-coordinate cadmium center with a similarly short Cd–C bond length of 2.135(3) Å. The slight elongation relative to **6** is likely due to the increased coordination number at cadmium. When **2** is used for insertion chemistry, clean formation of the double-insertion products [{(TMS)_2_N}­(Cp*)­Al­{(NR)__2__C}]_2_Cd (R = ^*i*^Pr: **8a**; R = Cy: **8b**) is observed, accompanied by comparably low-field ^13^C­{^1^H} NMR resonances for the coordinating carbene carbon atoms (∼200 ppm; see Figures S47 and S52). All ^13^C­{^1^H} NMR spectra provide additional evidence for proximity of the former carbodiimide to cadmium, most notably *via* detectable ^n^ *J*_Cd–C_ couplings (n = 2, 3; see SI). For **8a** and **8b**, where crystal structures remain elusive, further insight is provided by solution-state NMR. In **8a**, broad satellites reveal a one-bond coupling constant of ∼865 Hz (^1^ *J*_Cd–C_), confirming Cd–C coordination in solution. For **8b**, couplings to suitable cadmium isotopes were fully resolved, revealing ^1^ *J* ^111^_Cd–C_ = 817 Hz and ^1^ *J* ^113^_Cd–C_ = 856 Hz (Scheme 4c).

Given its isoelectronic relationship to carbodiimides, carbon dioxide was next investigated as a substrate for insertion reactivity. Exposing a dry ice–frozen benzene solution of **1** to an atmosphere of CO_2_, followed by slow warming to ambient temperature, immediately produces black metallic precipitates. NMR spectroscopic analysis of the resulting mixture reveals a diagnostic ^13^C­{^1^H} NMR resonance at 171.5 ppm, consistent with the formation of a carbamate. This assignment was confirmed by SC-XRD, which revealed the structure of [{N­(TMS)_2_}­(Cp*)­Al­{(O_2_C–N­(TMS)_2_)}] (**9**). Here, CO_2_ is incorporated as a carbamate ligand while cadmium is fully extruded. Given our findings with carbodiimides and the successful isolation of compounds **6** and **7**, we propose that CO_2_ undergoes insertion at various positions within the framework. However, insufficient kinetic stabilization paired with the strong oxophilicity of aluminum appears to disfavor the formation of a stable heterobimetallic entity. Attempts to react **2** with CO_2_ also proved unselective, likely due to the known susceptibility of [{N­(TMS)_2_}]^−^ fragments to CO_2_-induced decomposition at metal centers.^95^ Despite these limitations, the structural authentication of the elusive carbamate complex **9** was achieved (Figure S93).

To the best of our knowledge, nucleophilic behavior at cadmium has not been previously reported. The reaction of **1** with DIC presents a computationally accessible model to explore this reactivity (Figure 1). According to our calculations (ZORA-DLPNO–CCSD­(T) CPCM­(C_6_H_6_)/ZORA def2-TZVPP­{SARC-ZORA-TZVPP­(Cd)}//r^2^SCAN-3c), the pathway begins with the coordination of DIC to the aluminum center, forming **IM1′** (Δ_R_ *G* = +26 kJ mol^–1^), an energetically uphill intermediate. This is followed by Al–Cd bond cleavage *via* transition state **TS1′** (Δ_R_ *G* ^‡^ = +99 kJ mol^–1^), yielding **IM2′** (Δ_R_ *G* = +36 kJ mol^–1^), a key four-membered Al–N–C–Cd intermediate. From **IM2′**, two pathways to product formation are accessible. The first proceeds directly to **6a** *via* **TS2’A** (Δ_R_ *G* ^‡^ = +50 kJ mol^–1^), ultimately affording the product in a strongly exergonic step (Δ_R_ *G* = –88 kJ mol^–1^). The second involves formation of a distinct intermediate, **IM3** (Δ*G* = –10 mol^–1^), *via* **TS2’B** (Δ_R_ *G* ^‡^ = +100 mol^–1^). **IM3** may then rearrange to form **6a** through **TS3**; however, the associated energy barrier (Δ_R_ *G* ^‡^ = +144 kJ mol^–1^) is prohibitively high for a process occurring at ambient temperature. Subsequent addition of a second equivalent of carbodiimide leads to scission of the Cd–N bond and formation of the guanidinate product **7a**, with a further release of energy (Δ_R_ *G* = –120 kJ mol^–1^; net Δ_R_ *G* = –32 kJ mol^–1^ for this step). Overall, these data support the operation of bimetallic cooperativity and highlight two plausible mechanistic pathways, with the low-energy route *via* **TS2’A** being the most viable.

**Figure 1:**
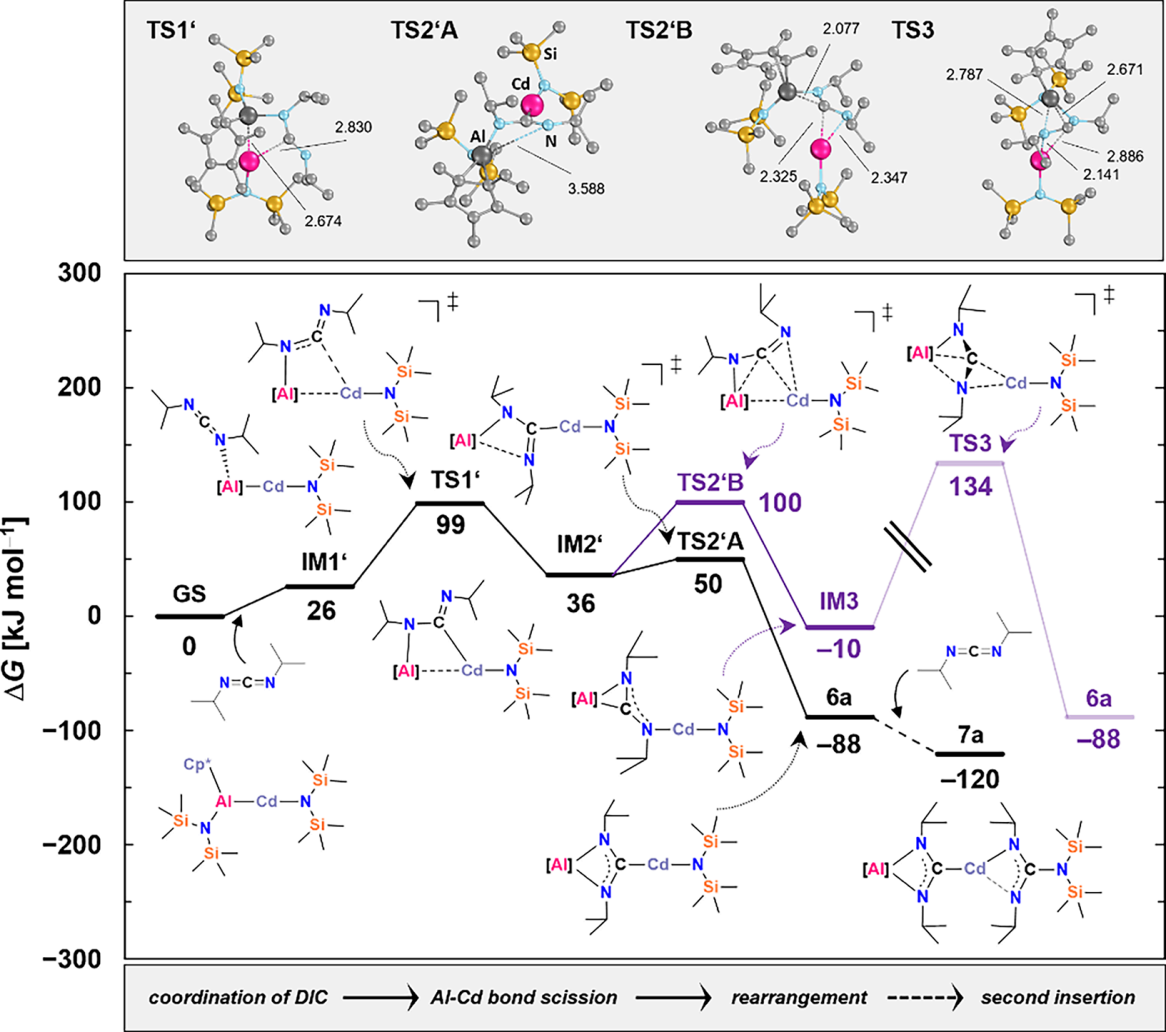
Gibbs Free Energy Pathway (Δ_R_ *G*) for the Insertion of DIC at the ZORA-DLPNO–CCSD­(T) CPCM­(C_6_H_6_)/ZORA def2-TZVPP­{SARC-ZORA-TZVPP­(Cd)}//r^2^SCAN-3c Level of Theory**.** Atom distances depicted in Å.

## Conclusion

In summation, we report a versatile and modular approach for accessing Al–Cd heterobimetallic complexes *via* reaction of Cd­{N­(TMS)_2_}_2_ with monomeric [AlCp*], leading to the selective formation of the bimetallic cadmium aluminyl **1** and, upon further Al­(I) addition, the trimetallic species **2**. Structural and spectroscopic characterization, supported by high-level quantum chemical calculations, confirms high, yet tunable Al–Cd covalency and reveals **2** as a platform for efficient Al­(I) transfer. **2** enables heterometal-templated Al­(I) shuttling to B, Cd, Zn, and Ag substrates (**1** ^**Zn**^, **3**–**5**). This dynamic behavior highlights **2** as a rare example of a well-defined Al­(I) transfer reagent. The reactivity toward heterocumulenes such as carbodiimides (and CO_2_) highlights synergism between Al and Cd with cadmium exhibiting nucleophilic reactivity (**6**–**8**), a hitherto unobserved feature for this element. By enabling cadmium to engage in (reversible) electron transfer and bond activation processes, this work addresses a longstanding gap in molecular low-valent cadmium chemistry.

## Supplementary Material



## Notes

Synthetic procedures, spectroscopic data, computational and crystallographic details (PDF)

## Abbreviations

CCSD­(T): Coupled cluster with single, double, and perturbative triple excitations
Cp*: Pentamethylcyclopentadienyl (=C_1__0_H_1__5_)
CPCM: Conductor-like polarizable continuum model
DCC: *N*,*N*′-dicyclohexylcarbodiimide (=C_1__3_H_2__2_N_2_)
def2-TZVPP: def2 triple-zeta valence plus polarization
DIC: *N*,*N*′-diisopropylcarbodiimide
DLPNO: Domain-based local pair natural orbital
GS: Ground state
HOMO: Highest occupied molecular orbital
IAO: Intrinsic atomic orbital
IBO: Intrinsic bond orbital
IM: Intermediate
IPr: 1,3-Bis­(2,6-diisopropylphenyl)­imidazol-2-ylidene
LUMO: Lowest unoccupied molecular orbital
MOF: Metal–organic framework
NMR: Nuclear magnetic resonance
OTf: Trifluoromethanesulfonate (=triflate; CF_3_SO_3_^–^)
r^2^SCAN-3c: Regularized strongly constrained and appropriately normed functional with 3c corrections
SARC: Segmented all-electron relativistically contracted
SC-XRD: Single-crystal x-ray diffraction
TMP: 2,2,6,6-tetramethylpiperidine (-ide)
TMS: Trimethylsilyl (-ane) (= −Si­(CH_3_)_3_ ; =Si­(CH_3_)_4_)
TS: Transition state
ZORA: Zeroth order regular approximation
Δ_R_ *G*: Change in Gibbs-free energy
