# Supplementary material for: Functional Al/Cd Heterometallics—From Controlled Al(I) Transfer to Nucleophilic Transfer of Cadmium Ions
Source: J Am Chem Soc. 2025 Aug 25;147(36):33315–23. doi: 10.1021/jacs.5c12746 (PMC12426937; doi:10.1021/jacs.5c12746)
Supplement: Supplementary file 1 [file ja5c12746_si_001.pdf]

SUPPORTING INFORMATION

Functional Al/Cd Heterometallics – From Controlled Al(I) Transfer to Nucleophilic Transfer of Cadmium Ions

D. Herle,[a] S. Sommer,[a] F. Dankert [a]*

[a] Dominic Herle, Sara Sommer, Dr. Fabian Dankert

Institute of Chemistry, University of Kassel, Heinrich-Plett-Str. 40,

34132 Kassel, Germany

* Fabian.Dankert@uni-kassel.de

This file includes:

1 Experimental details S2

2 Synthesis and spectroscopy of selected starting materials S3

3 Syntheses of compounds and crystallization S15

4 NMR studies of Al(I)-transfer reactions S53

5 Additional NMR studies S66

6 X-ray structure elucidation and refinement S73

7 Computational details S84

8 References S109

1 Experimental details

General Information. All manipulations were carried out under oxygen- and moisture-free conditions under an inert atmosphere of argon using standard Schlenk techniques or a glovebox.[1] All reactants were stored and handled in an UNIlab mBraun glovebox with integrated freezer. Solvents and reactants were either obtained from commercial sources, local trade or synthesized according to literature procedures. Activation of molecular sieves (3Å, 4Å) was achieved through several microwave irradiation cycles (700W; one minute each). Further activation followed through applying vacuum for several hours. All sieves were subsequently stored under inert atmosphere. Internal standards $SiMe_4$ (TMS) and Naphthalene used were purchased from Thermo Scientific. TMS was dried over molecular sieves (3 Å) and subsequently stored under inert atmosphere in an ampule with PTFE valve (FengTecEx). Benzene-d_6 (Deutero) was degassed using the freeze-pump thaw method and subsequently dried over molecular sieves (4 Å). Once transferred to the glovebox, it was stored over a new set of freshly activated molecular sieves. N,N''-Diisopropylcarbodiimide (DIC) was purchased from Carl Roth and was treated *via* the freeze-pump thaw method. Subsequently it was dried over molecular sieves (3 Å) and stored under argon. The dry DIC was subsequently transferred to the glovebox and stored over a fresh, second set of sieves. N,N"-Dicyclohexylcarbodiimide (DCC) was purchased from CarlRoth and stored in the glovebox. $B(C_6F_5)_3$ (>98%) was purchased from TCI, stored at –30°C in the glovebox and used as received. CO_2 (Carbagas) was used as received. $(AlCp^*)_4$ was synthesized *via* reductive elimination according to a procedure reported by FISCHER.[2] $Zn\{N(TMS)_2\}_2$ was synthesized according to a procedure reported by HARTWIG with slight modifications.[3] $Cd\{N(TMS)_2\}_2$ was synthesized according to a procedure reported by WANNAGAT with slight modifications (see section 2.2).[4] $Cd(TMP)_2$ was synthesized according to a procedure reported by MULVEY and ROBERTSON yet with some modifications (subliming the “waxy yellow solid” while gradually heating the flask up to 120°C *in vacuo* ($1\cdot10^{-3}$ mbar) to obtain pure crystalline material (see section 2.3)).[5] NMR-data were recorded on Jeol JNM-ECZL500 or Varian VNMRS-500 MHz spectrometers at 25°C. NMR spectra are referenced internally to the deuterated solvent (^{13}C: C_6D_6 δref = 128.06 ppm) or to protic impurities in the deuterated solvent (^{1}H: C_6HD_5 δref = 7.16 ppm) or externally (^{11}B: $BF_3\cdot Et_2O$, δref = 0 ppm; ^{19}F: $CFCl_3$, δref = 0 ppm; ^{27}Al: $Al(NO_3)_3$ in H_2O, δref = 0 ppm; ^{29}Si: TMS, δref = 0 ppm; ^{31}P: H_3PO_4 (85% in H_2O), δref = 0 ppm; ^{113}Cd: $CdMe_2$, δref = 0 ppm. The full width at half maximum (FWHM) for the signals in ^{27}Al NMR spectra is denoted as $\omega_{1/2}$. CHN elemental microanalyses were conducted with a HEKAtech Euro EA CHNS elemental analyser. UV-VIS spectra were recorded on a Shimadzu UV-2600 spectrometer in quartz cells with a path length of 1 cm.

2 Synthesis and spectroscopy of selected starting materials

This section reports NMR spectra for selected starting materials that were synthesized in our laboratory.

2.1 $(AlCp^*)_4$

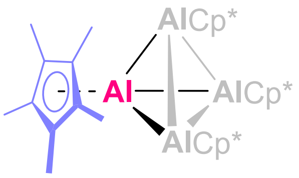

---

^{1}H NMR (500 MHz, C_6D_6) ***δ***: 1.90 (s, 60H, C*H*$_3$ of Cp*) ppm.

^{13}C{^{1}H} NMR (101 MHz, C_6D_6) ***δ***: 11.5 (s, *C*H$_3$ of Cp*), 114.0 (s, C_q of Cp*) ppm.

^{27}Al NMR (130 MHz, C_6D_6, 298 K) ***δ***: −79.3 (ω = 101 Hz) ppm.

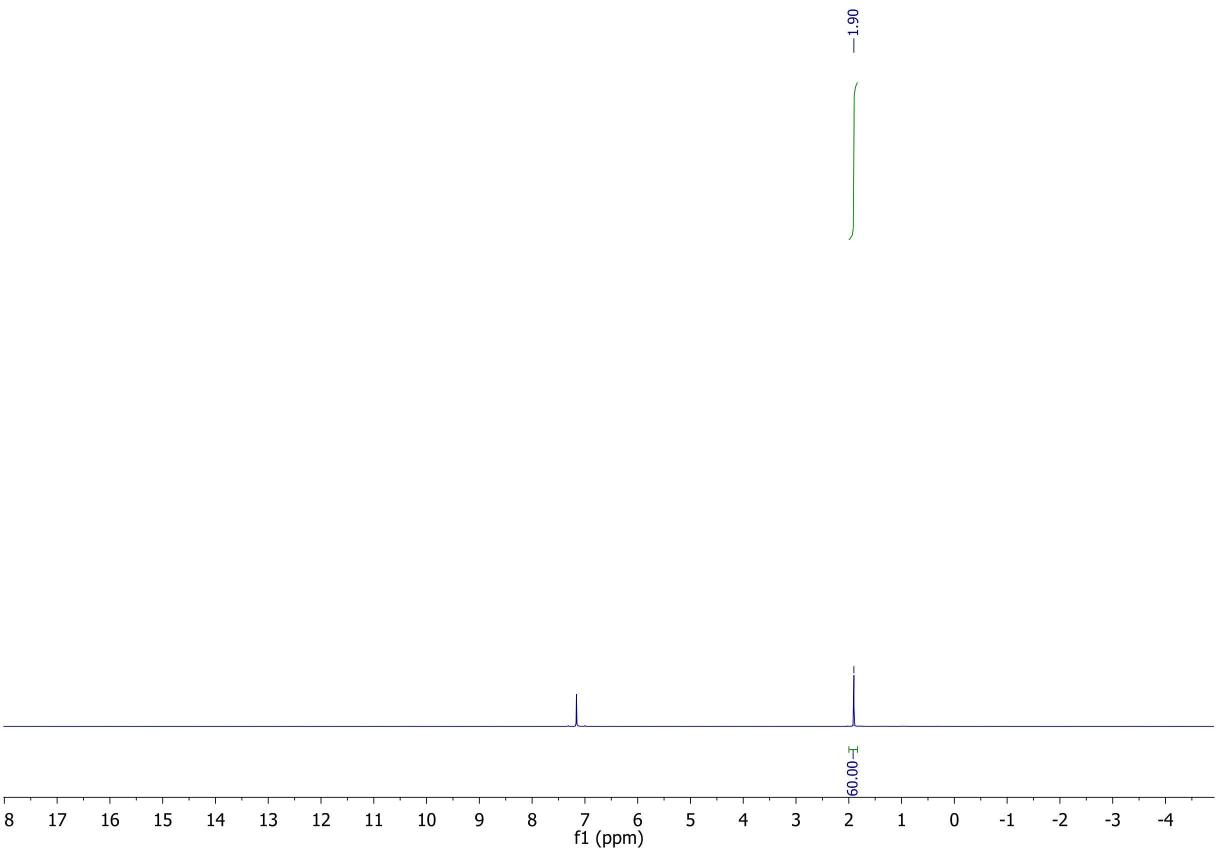

Figure S1: ^{1}H NMR (500 MHz, C_6D_6) of **$(AlCp^*)_4$**.

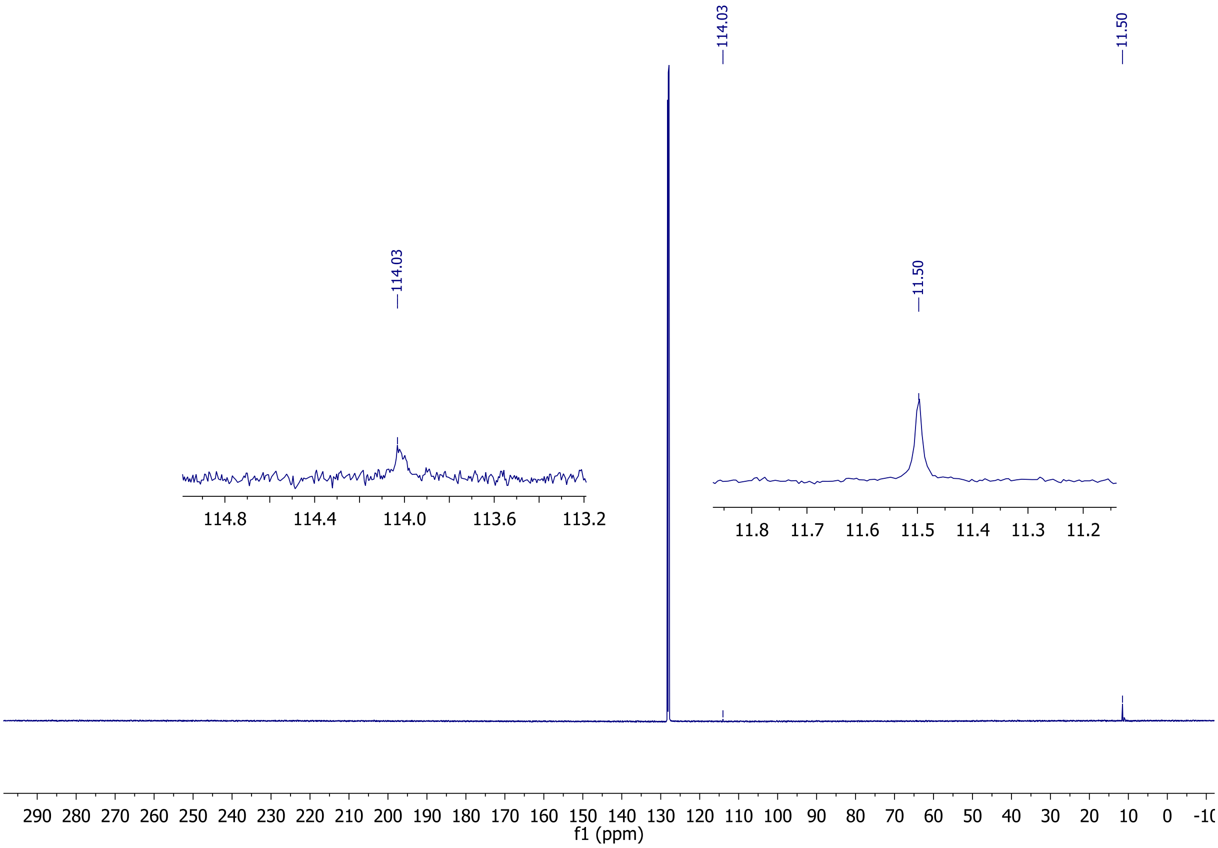

Figure S2: $^{13}C\{^{1}H\}$ NMR (101 MHz, C_6D_6) of **$(AlCp^*)_4$**.

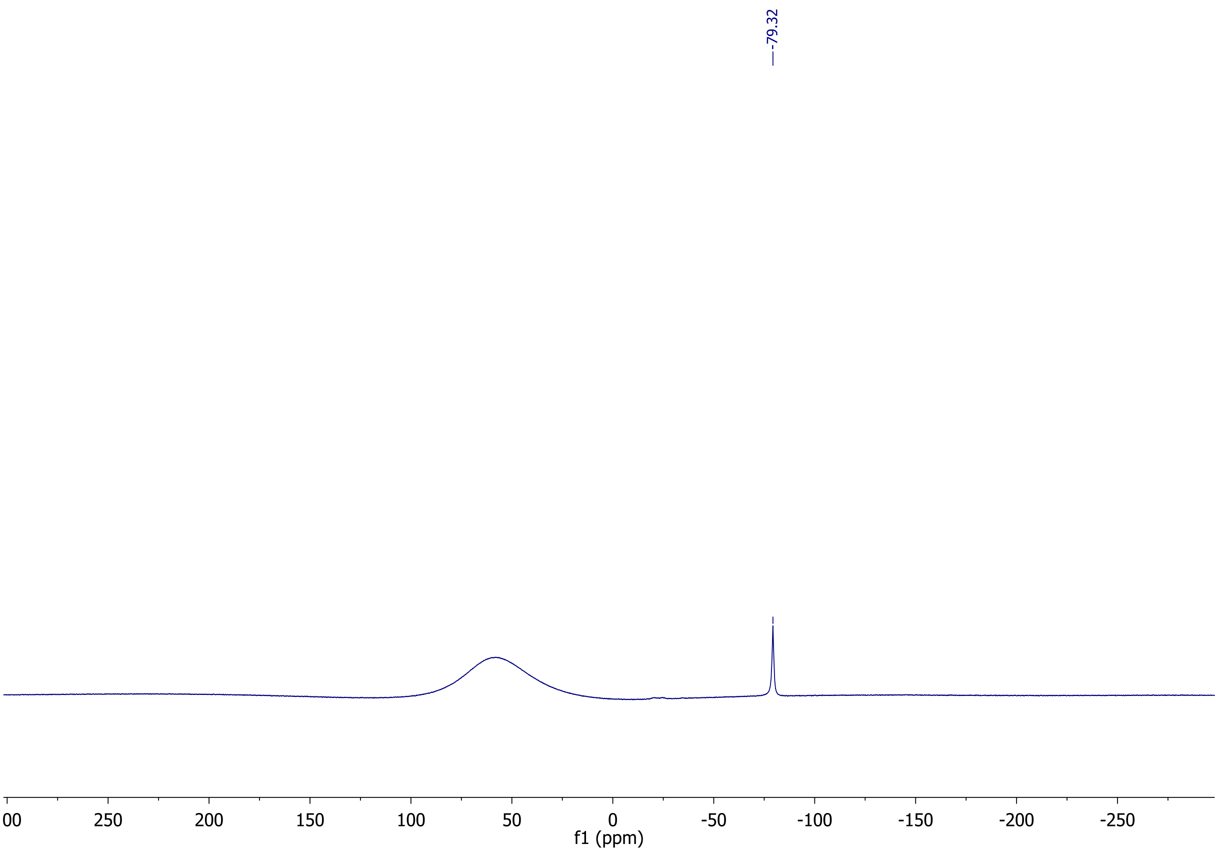

Figure S3: ^{27}Al NMR (101 MHz, C_6D_6) of **(AlCp*)$_4$**.

2.2 Cd{N(TMS)$_2$}$_2$

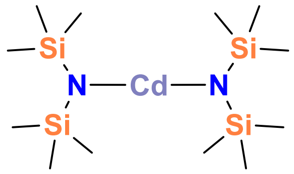

The compound was synthesized according to the literature.[4] However, we used $CdCl_2$ instead of CdI_2, THF instead of Et_2O and Li{N(TMS)$_2$} instead of Na{N(TMS)$_2$}. Based on a 10 mmol scale, we stirred the mixture for three hours at ambient temperature, removed the solvent *in vacuo* and extracted with 60 mL *n*-pentane. After filtration, all volatiles were removed and the residue dried in vacuo for at least an hour ($1 \cdot 10^{-3}$ mbar) to obtain pure material as a colourless oil. Yield: 1.600 g, 37%
Note: We recommend storing the neat compound in a glove-box integrated freezer at –30°C.

---

^{1}H NMR (500 MHz, C_6D_6) ***δ***: 0.19 (s, 36H, C*H*$_3$ of Cp*) ppm.

^{13}C{^{1}H} NMR (101 MHz, C_6D_6) ***δ***: 5.9 (s, *C*H$_3$ of –TMS) ppm.

^{29}Si{^{1}H} NMR (100 MHz, C_6D_6, 298 K) ***δ***: −0.5 (s, *Si* of HMDS) ppm.

^{113}Cd NMR (111 MHz, C_6D_6, 298 K) ***δ***: −348.7 ppm.

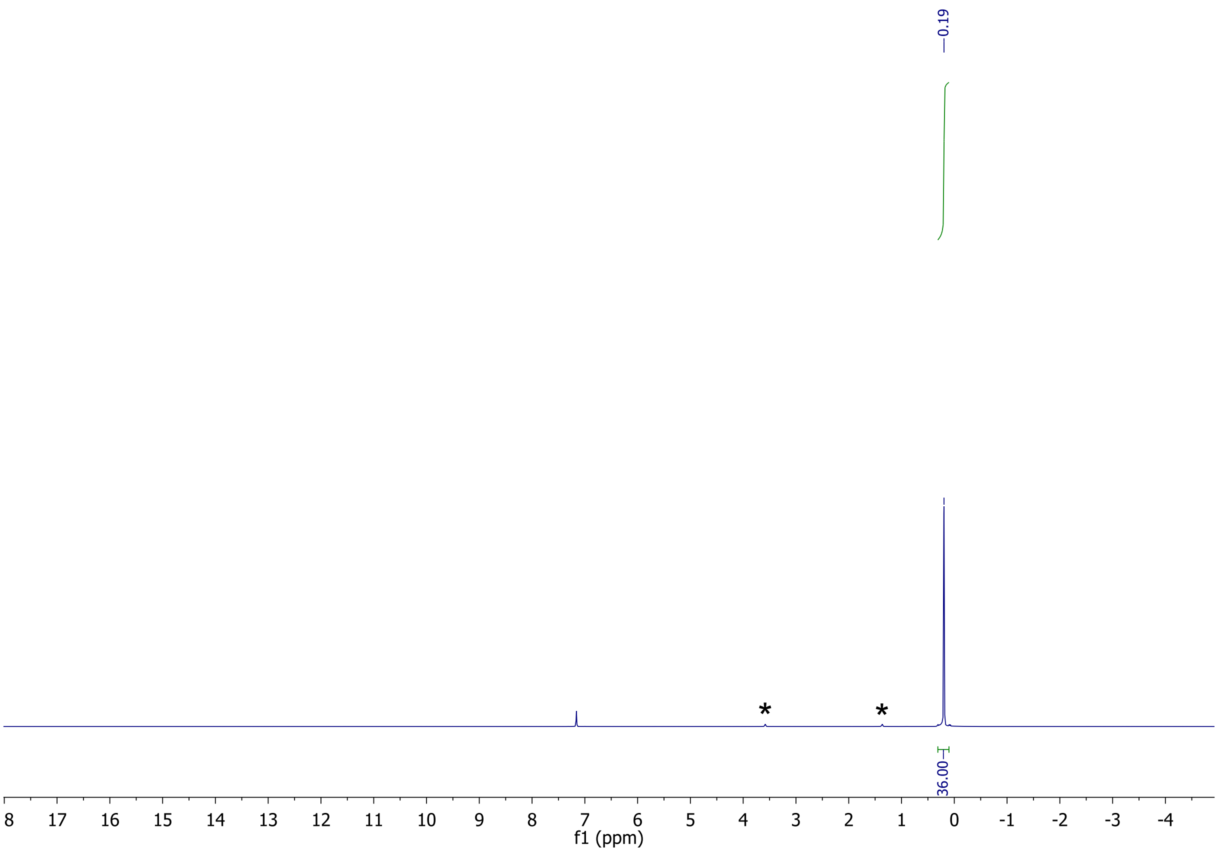

Figure S4: ^{1}H NMR (500 MHz, C_6D_6) of Cd{N(TMS)$_2$}$_2$. * trace THF.

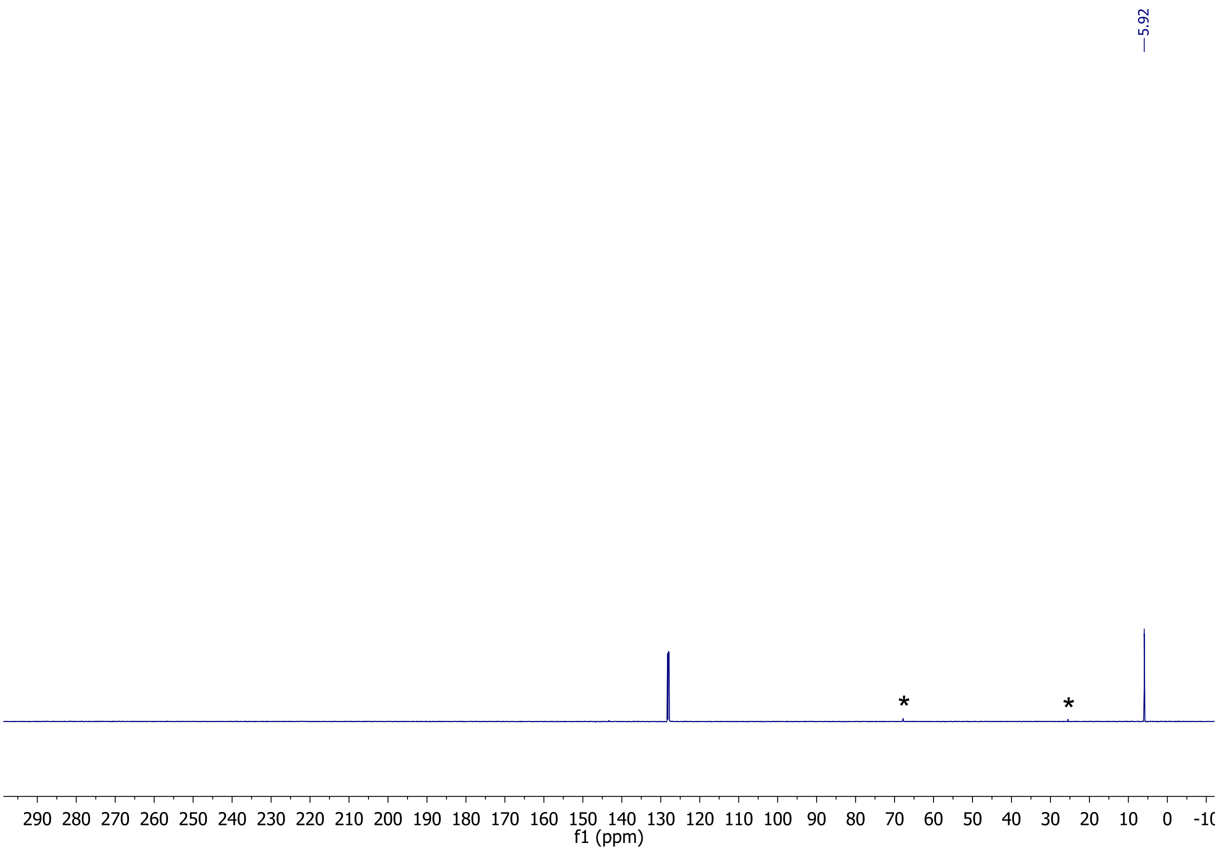

Figure S5: ^{13}C{^{1}H} NMR (101 MHz, C_6D_6) of Cd{N(TMS)$_2$}$_2$. * trace THF.

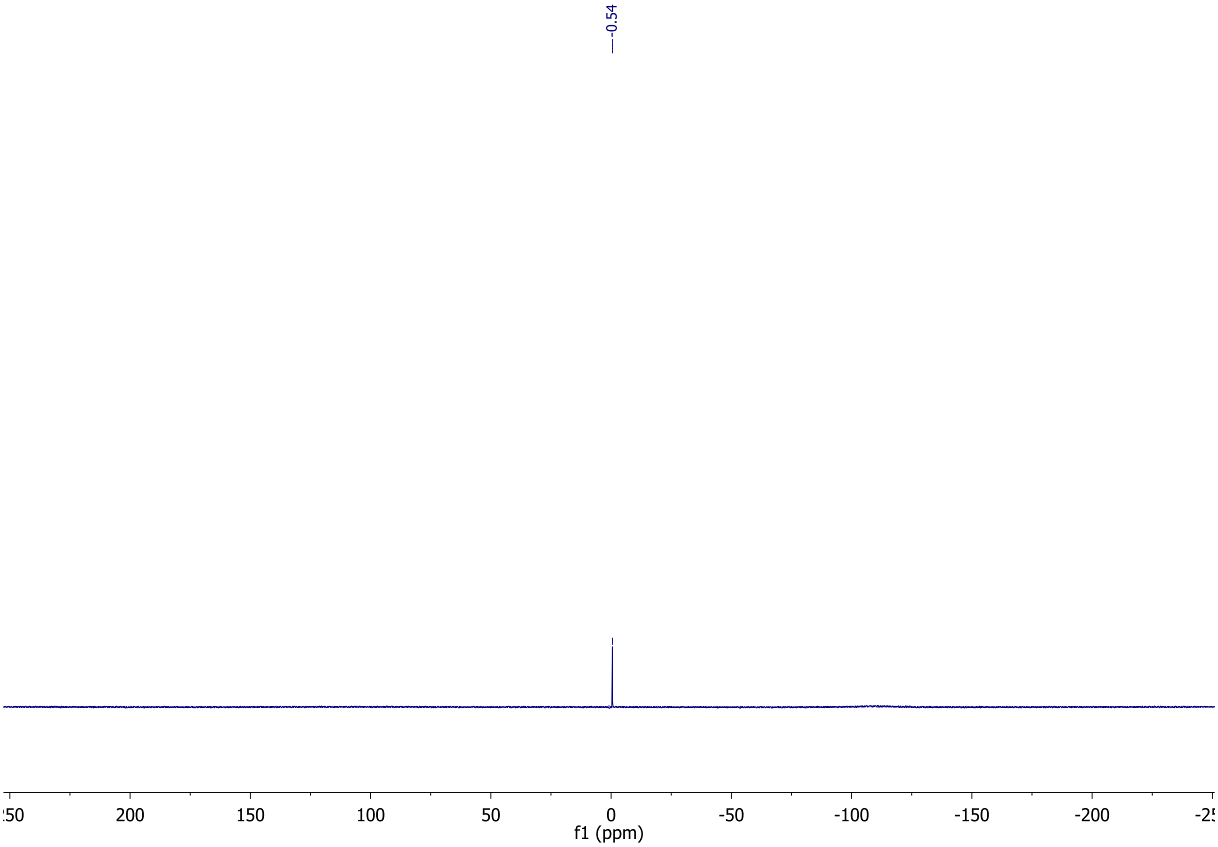

Figure S6: $^{29}Si\{^{1}H\}$ NMR (99 MHz, C_6D_6) of $Cd\{N(TMS)_2\}_2$.

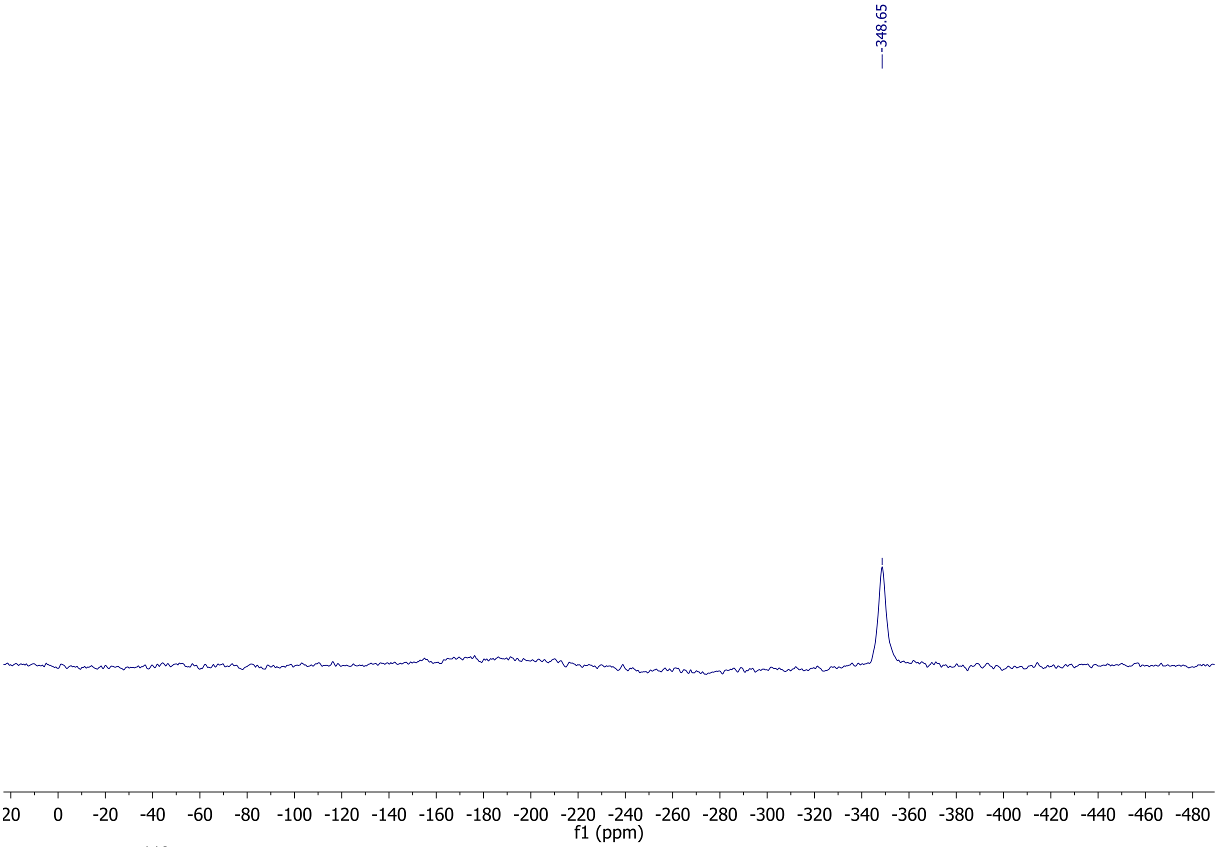

Figure S7: ^{113}Cd NMR (111 MHz, C_6D_6) of $Cd\{N(TMS)_2\}_2$.

2.3 Cd(TMP)$_2$

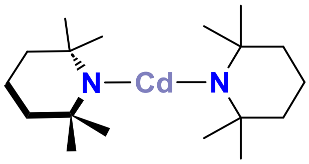

The compound was synthesized according to the literature.[5] However, we purified the compound by sublimation (see a depiction of the crystalline solid on the right; see Experimental Details for the respective conditions). Yield after sublimation: 1.970 g, 50% based on a 10 mmol scale. Note: We recommend storing the compound in a glove-box integrated freezer as it appears to be very sensitive.

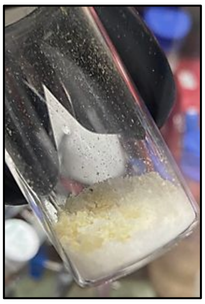

^{1}H NMR (500 MHz, C_6D_6) ***δ***: 1.25 (s, 24H, C*H*$_3$ of TMP), 1.42–1.45 (m, 8H, C*H*$_2$ of TMP), 1.68–1.73 (m, 4H, C*H*$_2$ of TMP) ppm.

^{13}C{^{1}H} NMR (101 MHz, C_6D_6) ***δ***: 54.1 (s, *C*$_q$ of *C*H$_3$), 39.5 (s, $^3J_{CdC}$ = 38 Hz (*Cadmium satellites*) *C*H$_2$ of TMP), 38.3 (s, *C*H$_3$ of TMP), 19.8 (s, *C*H$_2$ of TMP) ppm.

^{113}Cd NMR (111 MHz, C_6D_6, 298 K) ***δ***: –278.2 ppm.

Elemental Analysis calc. for $C_{18}H_{36}Cd_1N_2$ (found) C 55.02 (54.98), H 9.24 (9.58), N 7.13 (7.34).

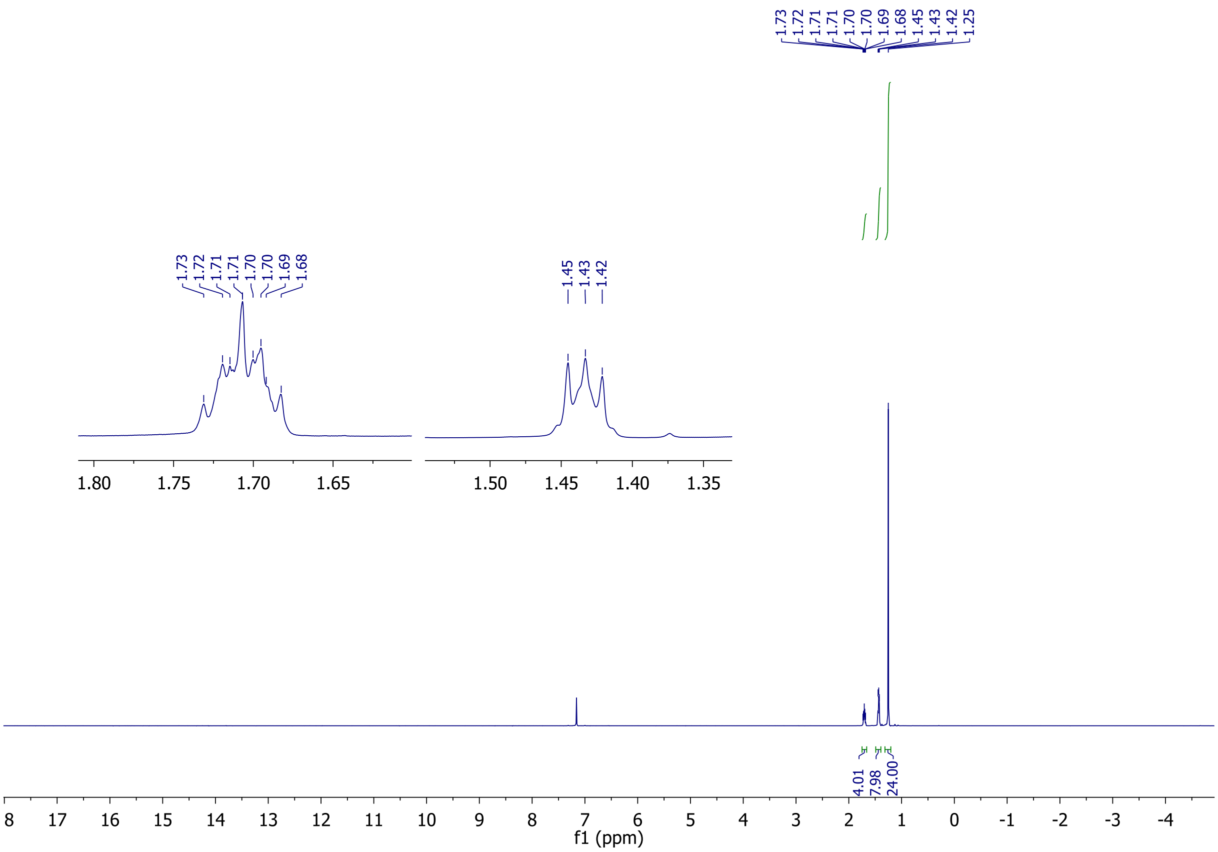

Figure S8: ^{1}H NMR (500 MHz, C_6D_6) of $Cd(TMP)_2$.

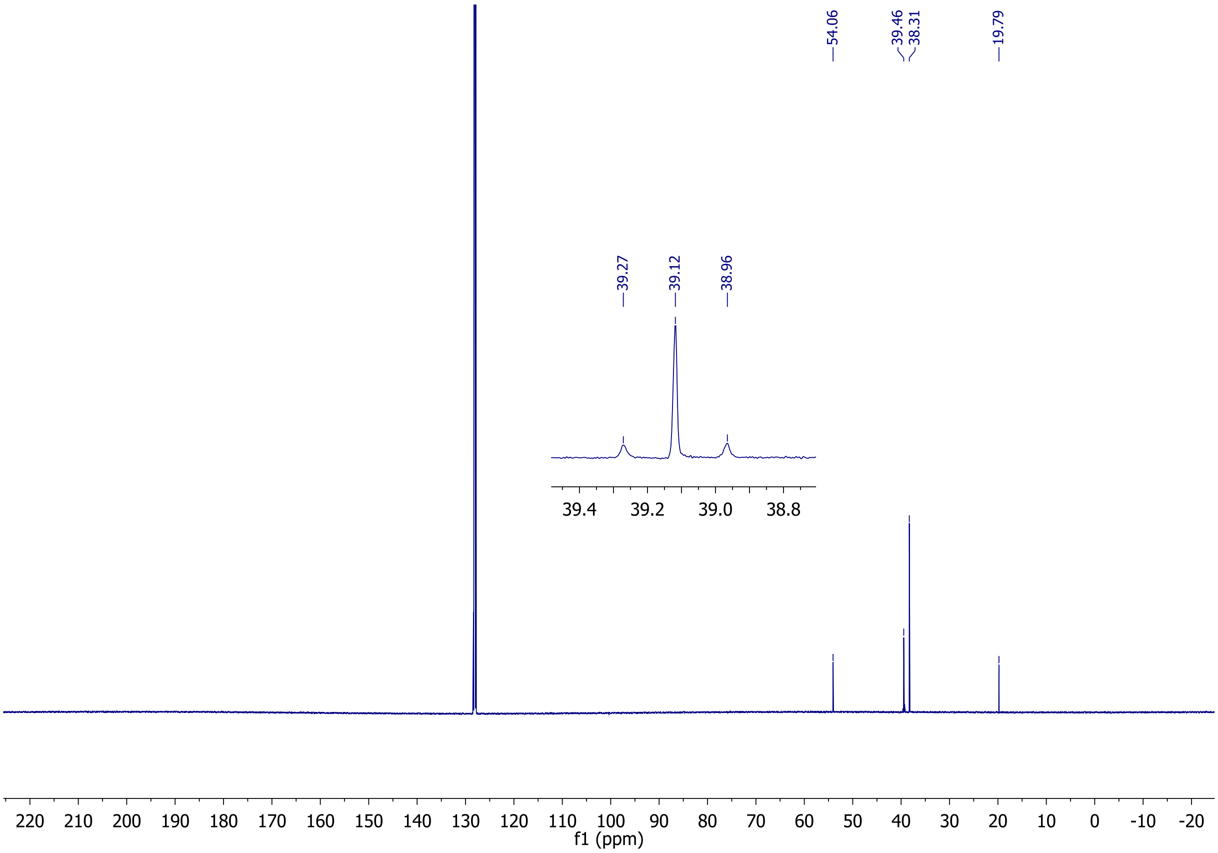

Figure S9: $^{13}C\{^{1}H\}$ NMR (101 MHz, C_6D_6) of $Cd(TMP)_2$.

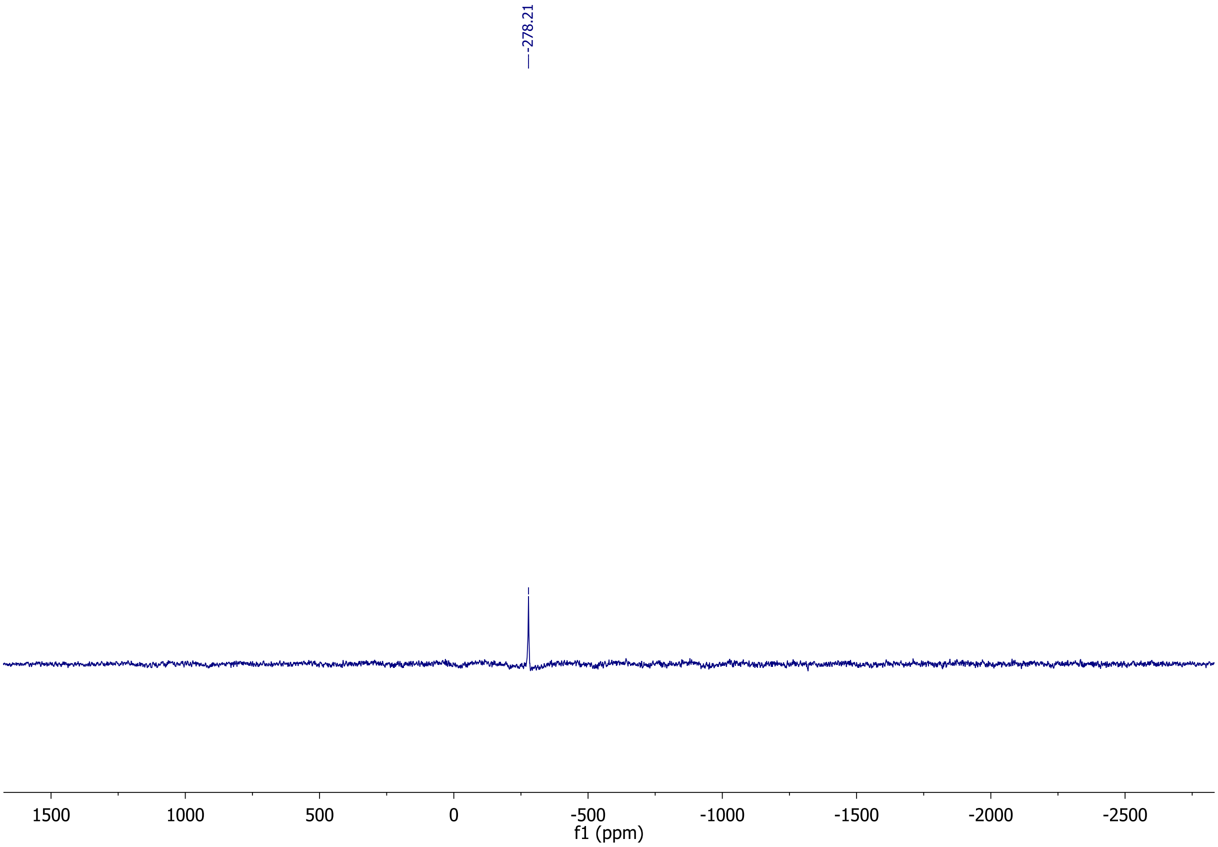

Figure S10: ^{113}Cd NMR (111 MHz, C_6D_6) of $Cd(TMP)_2$.

2.4 $Zn\{N(TMS)_2\}_2$

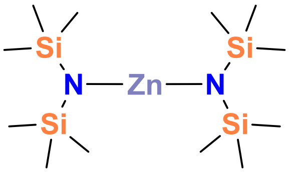

^{1}H NMR (500 MHz, C_6D_6) δ: 0.20 (s, 36H, C*H*$_3$ of Cp*) ppm.

^{13}C{^{1}H} NMR (101 MHz, C_6D_6) δ: 5.2 (s, *C*H$_3$ of –TMS) ppm.

^{29}Si{^{1}H} NMR (100 MHz, C_6D_6, 298 K) δ: 0.1 (s, *Si* of HMDS) ppm.

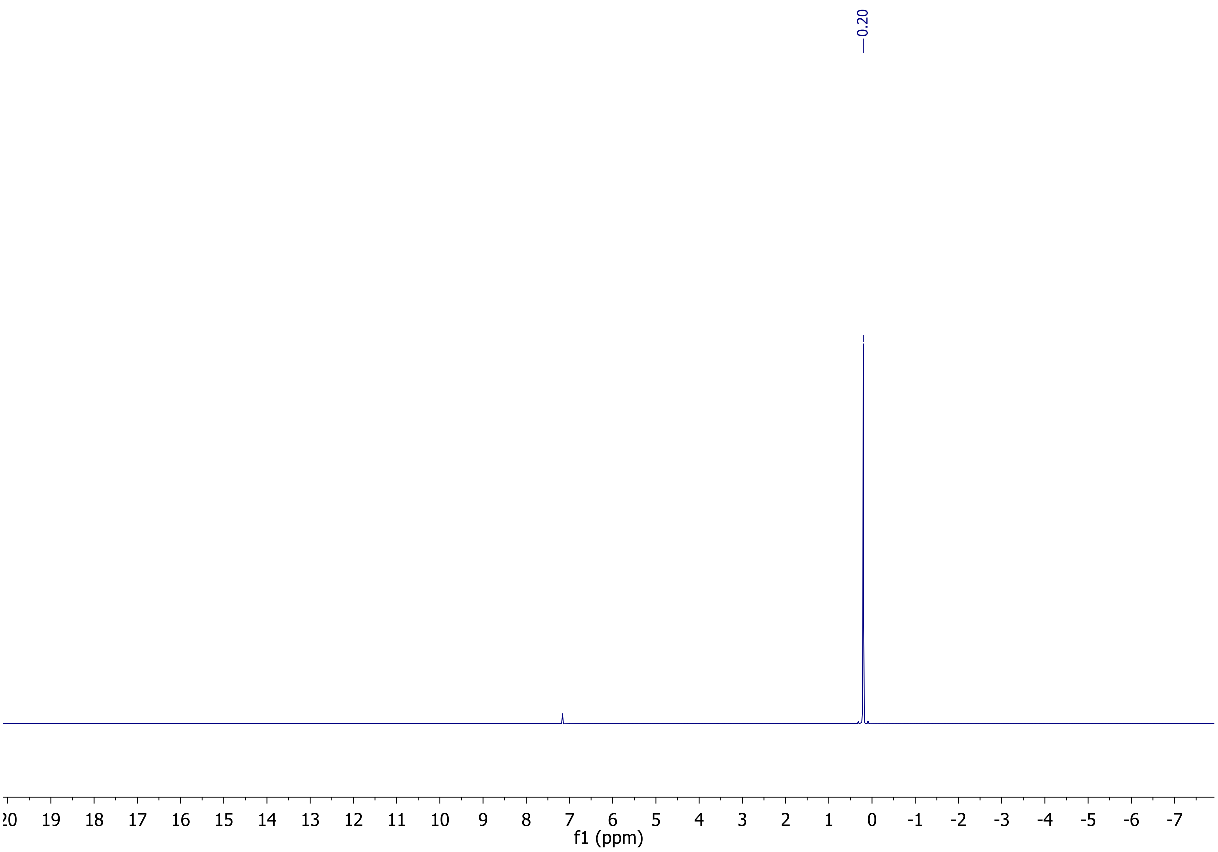

Figure S11: ^{1}H NMR (500 MHz, C_6D_6) of Zn{N(TMS)$_2$}$_2$.

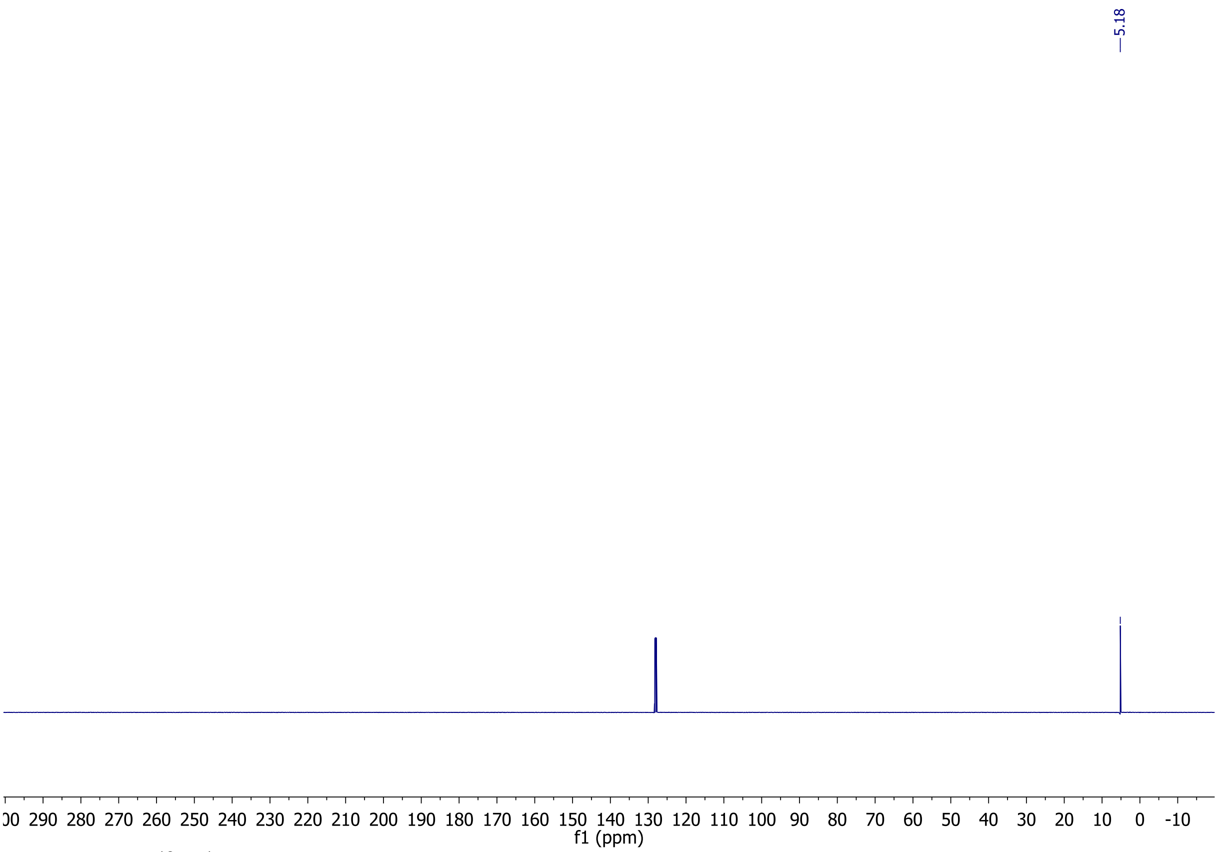

Figure S12: $^{13}C\{^{1}H\}$ NMR (101 MHz, C_6D_6) of Zn{N(TMS)$_2$}$_2$.

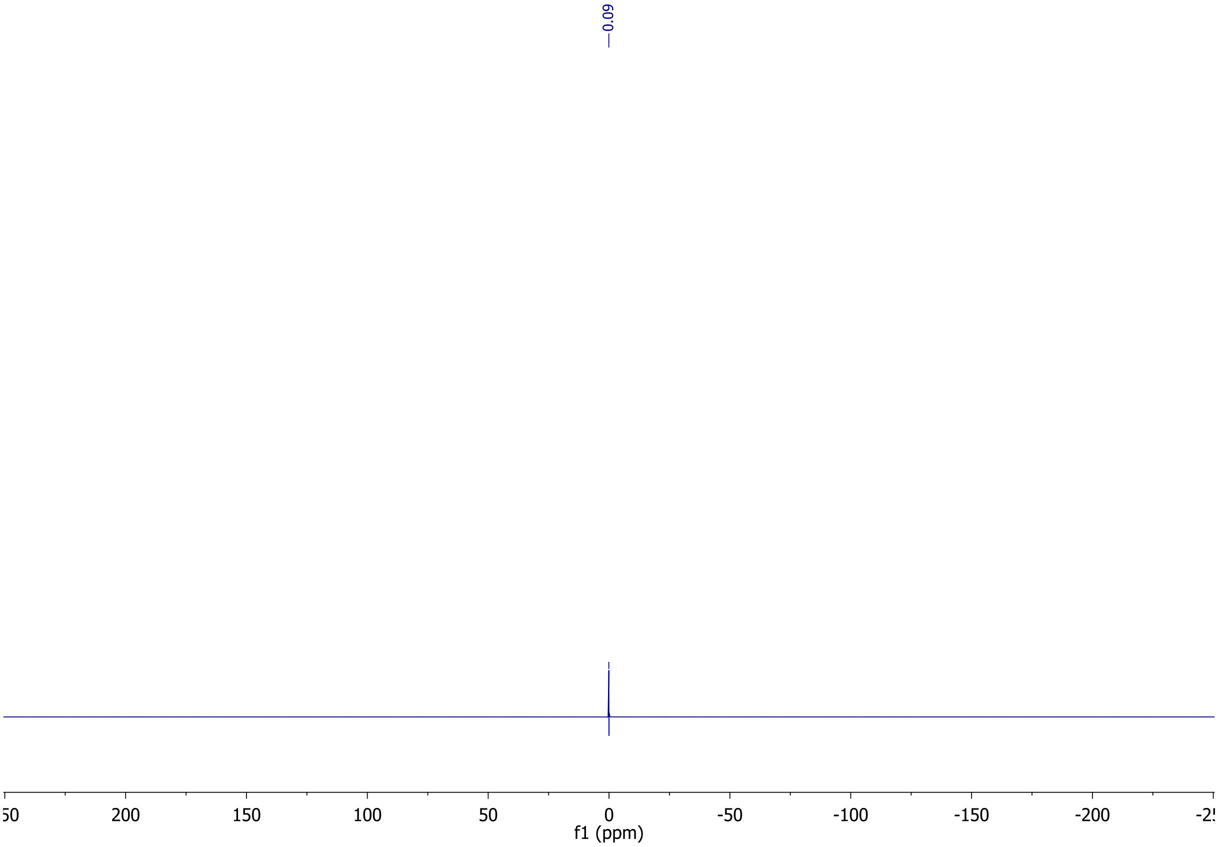

Figure S13: $^{29}Si\{^1H\}$ NMR (99 MHz, C_6D_6) of Cd{N(TMS)$_2$}$_2$.

3 Syntheses of compounds and crystallization

3.1 [({N(TMS)$_2$})(Cp*)Al–Cd{N(TMS)$_2$}] (**1**)

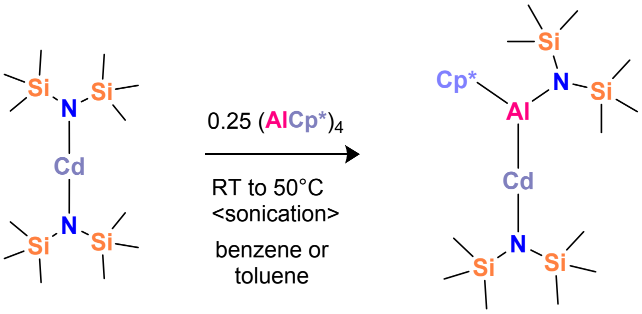

<u>*NMR Scale:*</u>

7.5 mg of finely ground [Cp*Al]$_4$ (0.012 mmol, 1.15 eq.) [1] and 17.3 mg Cd{N(TMS)$_2$}$_2$ (0.04 mmol, 1 eq.) were suspended in 1 mL of C_6D_6. The mixture was then transferred to a *J. Young* NMR tube. To convert the [Cp*Al]$_4$, the mixture was sonicated at 50°C for thirty minutes and afterwards thermally treated without sonication for an additional period of ten minutes. The conversion was gradually checked *via* ^{1}H-NMR. The yield was determined with tetramethylsilane as an internal standard and is quantitative. Isolated yields are determined in the experiment described below.

<u>*Upscaled Experiment:*</u>

In an ampoule with PTFE valve (FengTecEx), 18.7 mg of finely ground [Cp*Al]$_4$ (0.029 mmol; 1.0 eq. of "AlCp*") and 50 mg Cd{N(TMS)$_2$}$_2$ (0.115 mmol, 1 eq.) were suspended in 4 mL of toluene. To convert the [Cp*Al]$_4$, the mixture was sonicated at 50°C for thirty minutes and afterwards treated thermally without sonication for an additional period of ten minutes. Next, the solvent was removed under reduced pressure and the resulting grey residue thoroughly dried in vacuo. In the glovebox, 4 mL of *n*-pentane was then added followed by filtration through a PTFE syringe filter (d = 13 mm; pore size 0.22 µm). Slow evaporation of the *n*-pentane yields colorless plates of **1** (yield: 71 mg, 87%). <u>***Note:***</u> **1** is only marginally stable in neat form as a grey metallic coating appears after short periods (<12h), yet **1** is isolable. Coating occurs even when stored in a glovebox integrated freezer. However, we recommend using freshly, *in-situ* generated samples of **1** for reactivity studies and follow-up chemistry (*vide supra*).

The obtained crystals throughout work-up were suitable for SC XRD studies.

[1] Full conversion of Cd{N(TMS)$_2$}$_2$ is best reproducibly using a slight excess of [Cp*Al]$_4$.

Analytical Data:

^{1}H NMR (500 MHz, C_6D_6, 298 K) ***δ***: 1.92 (s, 15H, −*C*H_3 of Cp*), 0.31 (s, 18H, −*C*H_3 of HMDS), 0.21 (s, 18H, −*C*H_3 of HMDS) ppm. **^{1}H NMR** (500 MHz, toluene-d_8, 233 K) ***δ***: 1.91 (s, 15H, −*C*H_3 of Cp*), 0.35 (s, 18H, −*C*H_3 of HMDS), 0.22 (s, 18H, −*C*H_3 of HMDS) ppm.* **^{13}C{^{1}H} NMR** (126 MHz, C_6D_6, 298 K) ***δ***: 118.4 (s, C_q of Cp*), 11.6 (s, −*C*H_3 of Cp*), 6.2 (s, −*C*H_3 of HMDS), 5.4 (s, −*C*H_3 of HMDS) ppm. **^{27}Al NMR** (130 MHz, C_6D_6, 298 K) ***δ***: no resonance observed in a range of +300 – -300 ppm. **^{29}Si{^{1}H} NMR** (100 MHz, C_6D_6, 298 K) ***δ***: −4.2 (s, *Si* of HMDS), −2.6 (s, *Si* of HMDS) ppm. **^{113}Cd NMR** (111 MHz, C_6D_6, 298 K) ***δ***: no resonance observed in a range of +850 – -2000 ppm. **Elemental Analysis** calc. for $C_{22}H_{51}Al_1Cd_1N_2Si_4$ (found) C 44.38 (44.53), H 8.63 (8.81), N 4.71 (4.53).

* no splitting of CH_3 resonances observed.

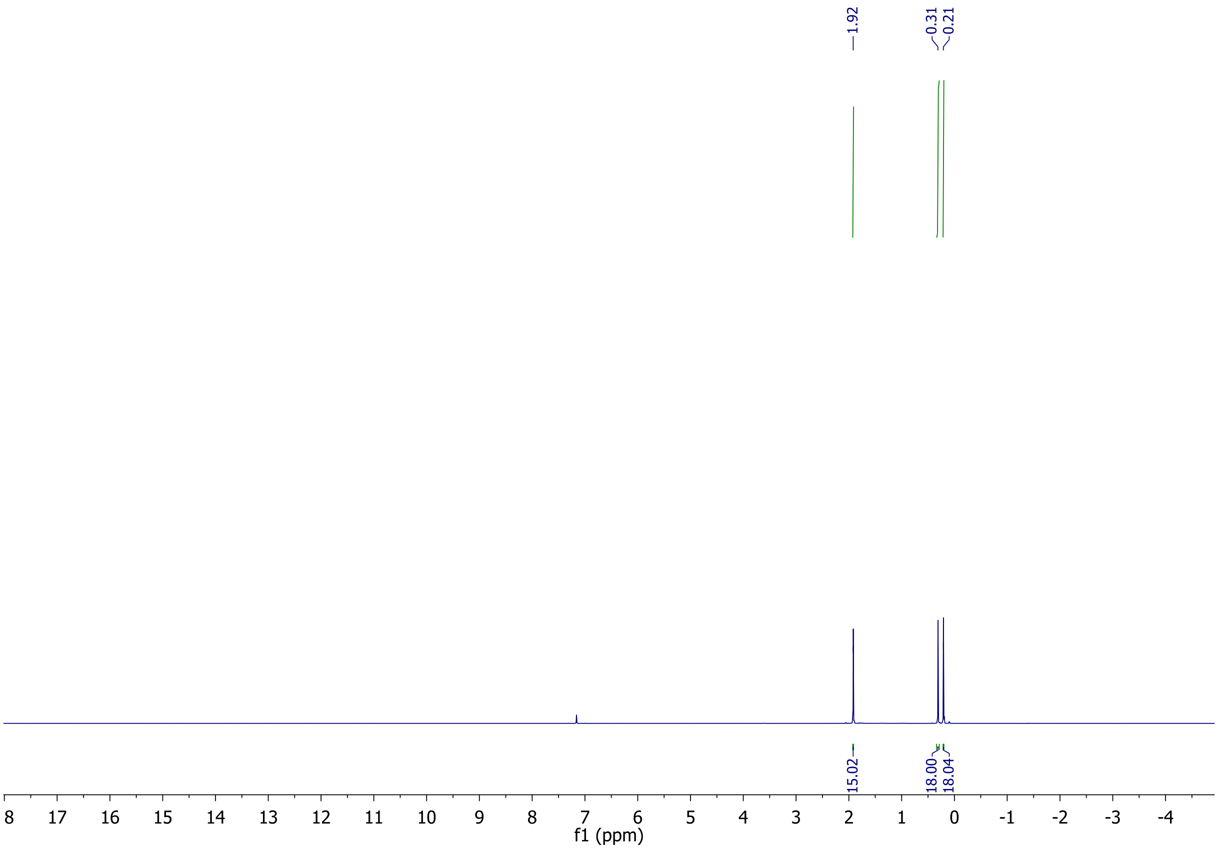

Figure S14: ^{1}H NMR (500 MHz, C_6D_6) of **1**.

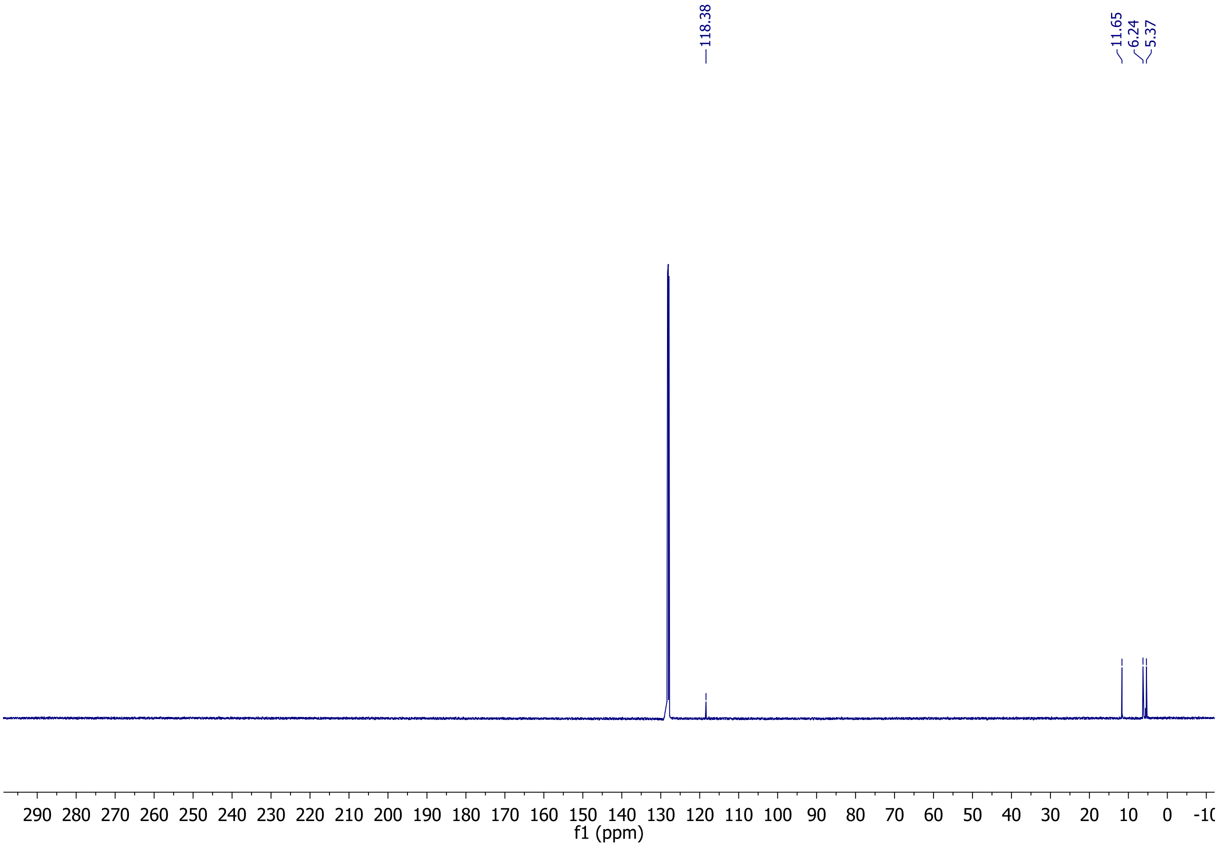

Figure S15: $^{13}C\{^1H\}$ NMR (126 MHz, C_6D_6) of **1**.

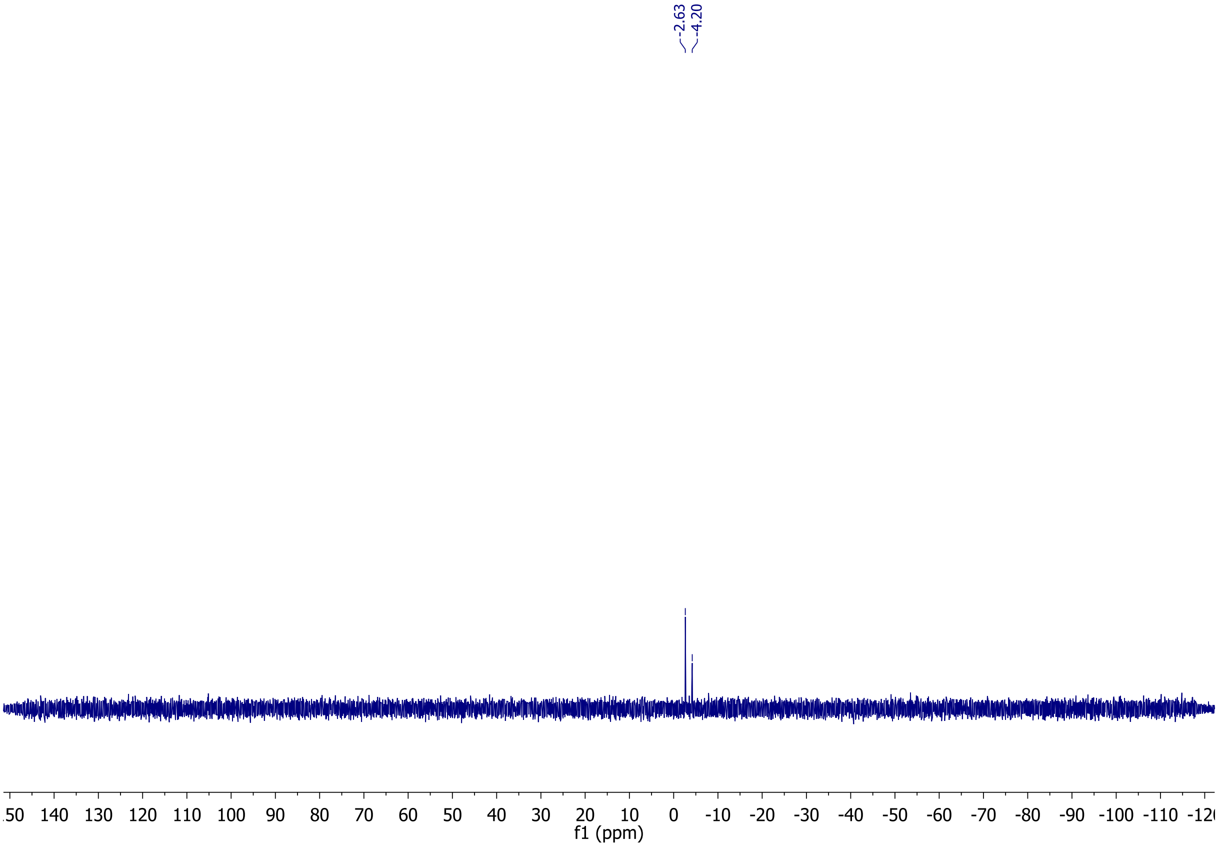

Figure S16: $^{29}Si\{^1H\}$ NMR (99 MHz, C_6D_6) of **1**.

3.2 [{N(TMS)$_2$}(Cp*)Al]$_2$Cd (**2**)

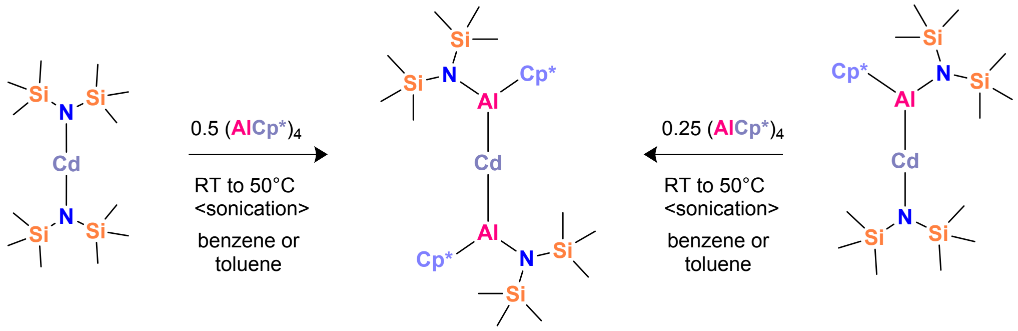

<u>*NMR Scale:*</u>

Method 1: 14.6 mg of finely ground [Cp*Al]$_4$ (0.022 mmol, 2.2 eq.[2]) and 17.3 mg Cd{N(TMS)$_2$}$_2$ (0.04 mmol, 1 eq.) were suspended in 1 mL of C_6D_6. The mixture was then transferred to a *J. Young* NMR tube. To dissolve the [Cp*Al]$_4$, the mixture was sonicated at 50°C for 20 minutes and afterwards heated without sonication for ten minutes. The conversion was gradually checked via ^{1}H NMR. Throughout the reaction, the color of the solution turned yellow. The yield was determined with tetramethylsilane as an internal standard and is quantitative. Isolated yields are determined in the experiment described below.

Method 2: Equimolar Reaction of one equivalent of isolated **1** and a slight excess of [Cp*Al]$_4$ (0.046 mmol, 1.15 eq.) can similarly and as well quantitatively be converted into **2** in C_6D_6 (10 min. sonication, 50°C).

<u>*Upscaled Experiment:*</u>

In an ampoule with PTFE valve (FengTecEx), 36.0 mg of finely ground [Cp*Al]$_4$ (0.058 mmol; a 2.0 eq. of "AlCp*") and 50 mg Cd{N(TMS)$_2$}$_2$ (0.115 mmol, 1 eq.) were suspended in 4 mL of toluene. To convert the [Cp*Al]$_4$, the mixture was sonicated at 50°C for twenty minutes and afterwards treated thermally without sonication for an additional period of ten minutes. Next, the solvent was removed under reduced pressure and the resulting grey residue thoroughly dried in vacuo. In the glovebox, 7 mL of *n*-pentane was then added followed by filtration through a PTFE syringe filter (d = 13 mm; pore size 0.22 µm). Slow evaporation of the *n*-pentane yields golden plates (see on the right) of **2** (yield: 71 mg, 87%). <u>***Note:***</u> **2** is only marginally stable in neat form as a grey metallic coating appears after short periods (<12h), yet **2** is isolable. Coating occurs even when stored in a glovebox integrated freezer. However, we recommend using freshly, *in-situ* gene-rated samples of **2** for reactivity studies and follow-up chemistry (*vide supra*). Crystals suitable for SC-XRD studies were obtained from an NMR sample at ambient temperature.

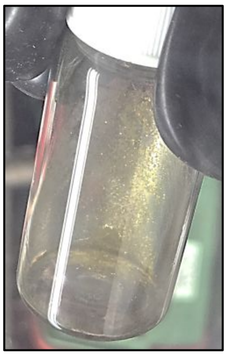

[2] Full conversion of Cd{N(TMS)$_2$}$_2$ is best reproducibly with a slight excess of [Cp*Al]$_4$.

Analytical Data:

^{1}H NMR (500 MHz, C_6D_6, 298 K) ***δ***: 2.06 (s, 30H, CH_3 of Cp*), 0.31 (s, 36H, CH_3 of HMDS) ppm. **^{13}C{^{1}H} NMR** (126 MHz, C_6D_6, 298 K) ***δ***: 117.6 (s, C_q of Cp*), 11.8 (s, −CH_3 of Cp*), 5.6 (s, −CH_3 of HMDS) ppm. **^{27}Al NMR** (130 MHz, C_6D_6, 298 K) ***δ***: 134 (ω = 5100 Hz) ppm. **^{29}Si{^{1}H} NMR** (100 MHz, C_6D_6, 298 K) ***δ***: −4.1 (s, *Si* of HMDS) ppm. **^{113}Cd NMR** (111 MHz, C_6D_6, 298 K) ***δ***: no resonance observed in a range of +850 – -2000 ppm. **Elemental Analysis** calc. for $C_{32}H_{66}Al_2Cd_1N_2Si_4$ (found) C 50.73 (50.19), H 8.78 (8.90), N 3.70 (3.58). **UV-VIS (C_6D_6):** visible absorption ranging from 380–500 nm.

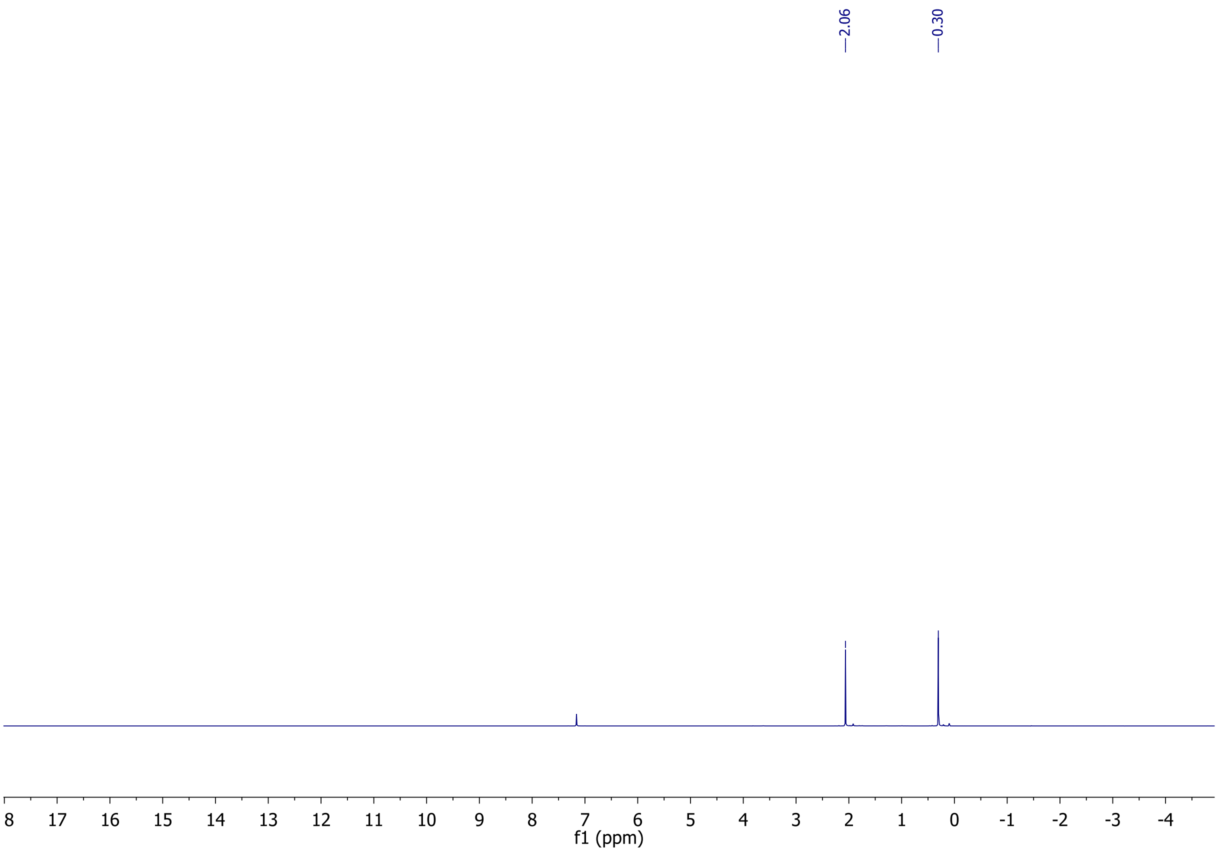

Figure S17: ^{1}H NMR (500 MHz, C_6D_6) of **2**.

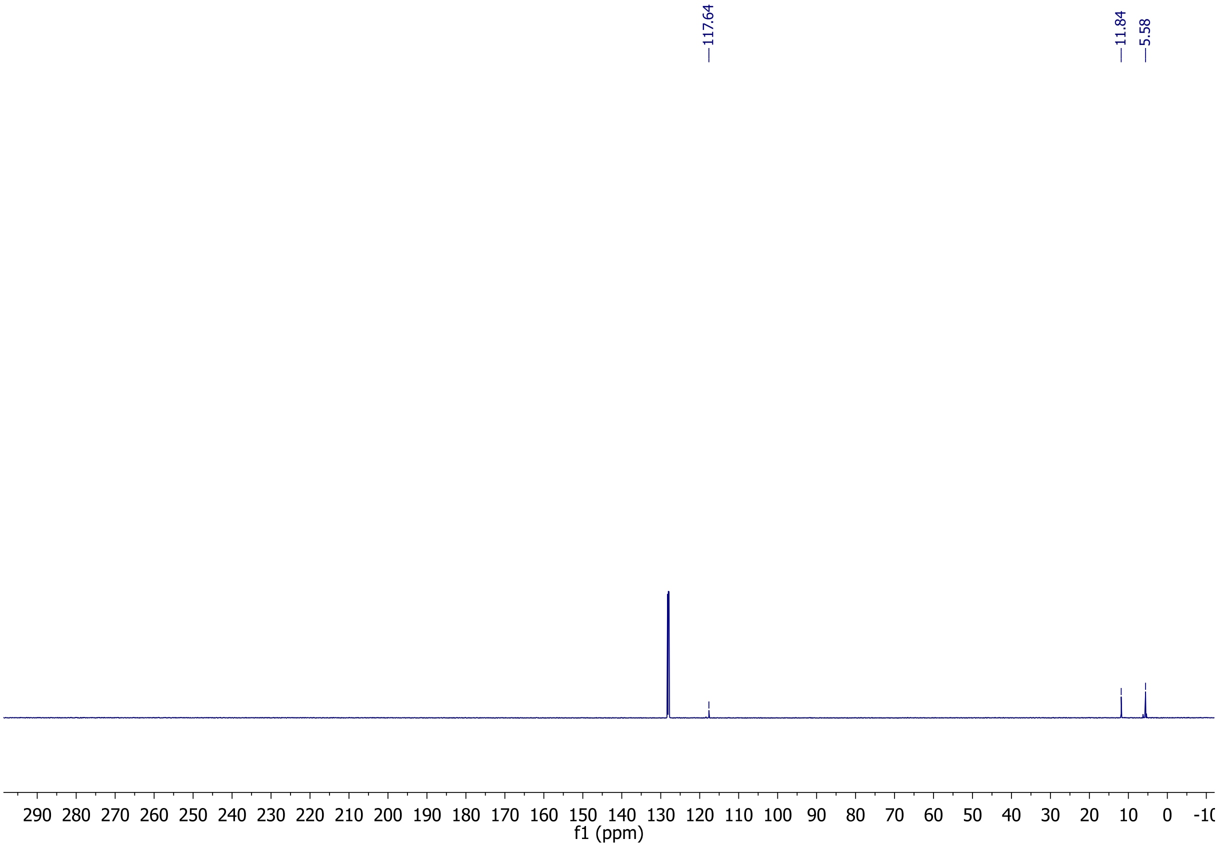

Figure S18: $^{13}C\{^1H\}$ NMR (126 MHz, C_6D_6) of **2**.

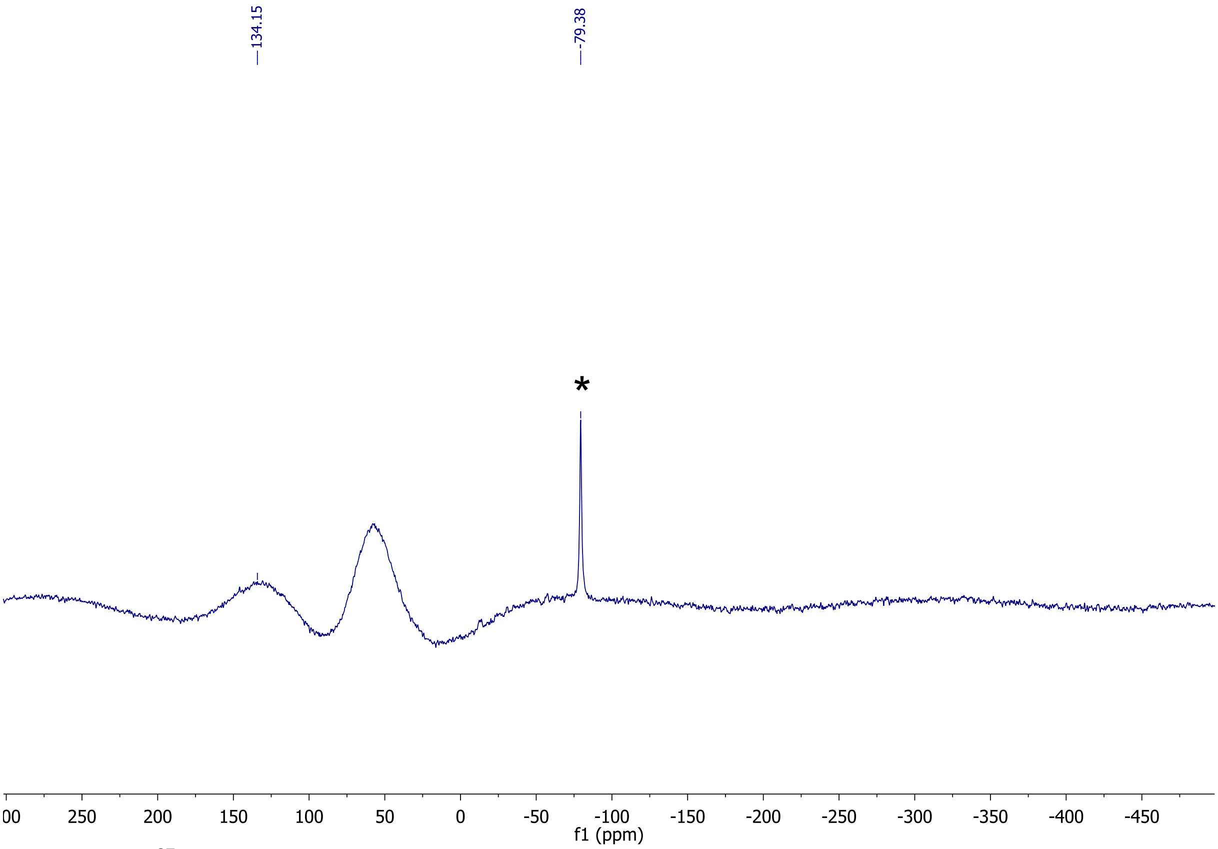

Figure S19: ^{27}Al NMR (130 MHz, C_6D_6) of **2**. Resonance at ~50 ppm = probe head; * = trace $(AlCp^*)_4$ as internal standard.

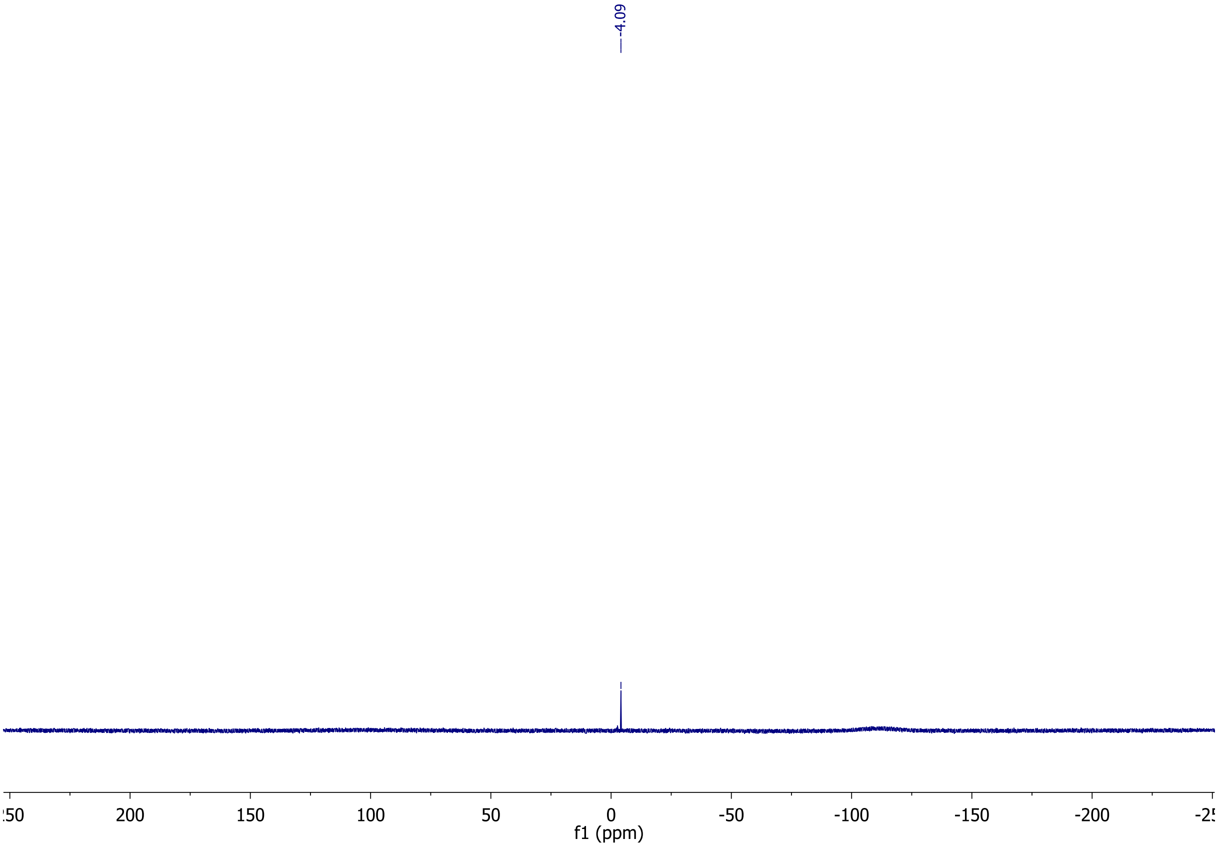

Figure S20: $^{29}Si\{^{1}H\}$ NMR (99 MHz, C_6D_6) of **2**.

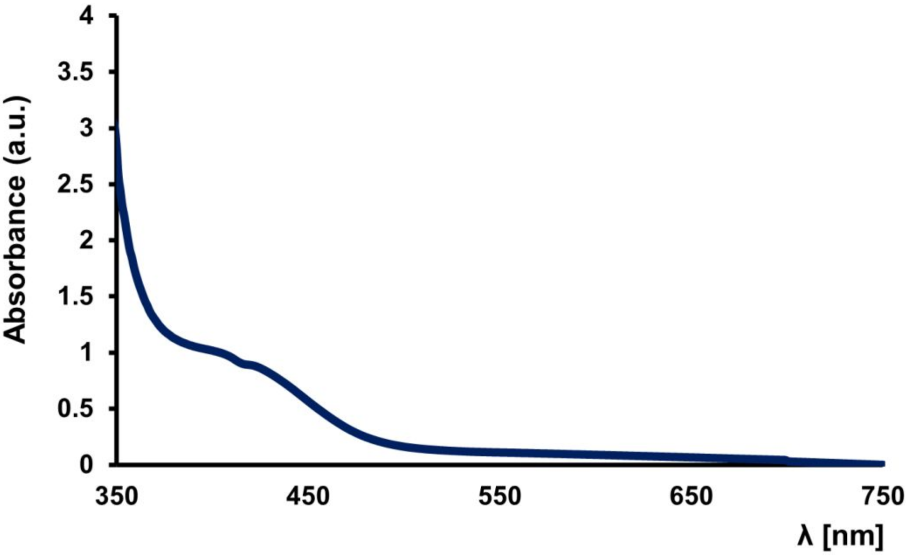

Figure S21: UV–VIS absorption spectrum (350-750 nm) of a freshly prepared solution of **2** in C_6D_6.

3.3 [(TMP)(Cp*)Al–Cd(TMP)] (4)

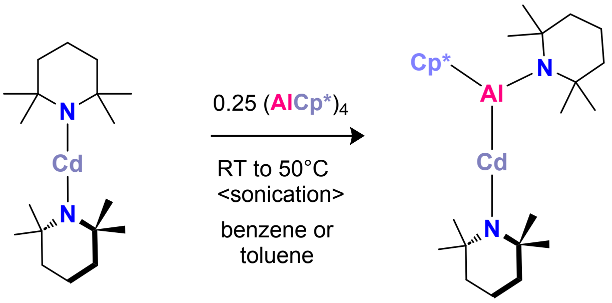

NMR Scale:

7.0 mg of finely ground $[Cp^*Al]_4$ (0.011 mmol; a 1.1 eq. of "AlCp*")) and 15.7 mg $Cd(TMP)_2$ (0.04 mmol, 1 eq.) were suspended in 1 mL of C_6D_6. The mixture was then transferred to a *J. Young* NMR tube. To convert the $[Cp^*Al]_4$, the mixture was sonicated at 50°C for 15 minutes and afterwards heated without sonication for an additional period of twenty minutes. Throughout, the conversion of $[Cp^*Al]_4$ was gradually checked *via* ^{1}H NMR and the reaction stopped when no $[Cp^*Al]_4$ was left. 4 appears as the main species among minor other species. Yields could not be reliably determined due to the sensitivity of the compound (see below).

Attempted Upscale Experiment:

In an ampoule with PTFE valve (FengTecEx), 18.7 mg of finely ground $[Cp^*Al]_4$ (0.029 mmol; a 1.0 eq. of "AlCp*") and 45.18 mg $Cd(TMP)_2$ (0.115 mmol, 1 eq.) were suspended in 4 mL of toluene. To convert the $[Cp^*Al]_4$, the mixture was sonicated at 50°C for 15 minutes and afterwards treated thermally without sonication for an additional period of ten minutes. Next, the solvent was removed under reduced pressure and the resulting grey residue thoroughly dried *in vacuo*. In the glovebox, 4 mL of *n*-pentane was then added followed by filtration through a PTFE syringe filter (d = 13 mm; pore size 0.22 µm). Evaporation of the solvent is accompanied by large amounts of black metallic precipitates (see right). The isolation of **4** unfortunately fails – even when exclusively working in the glovebox for work-up. However, a few colorless plates were identified from the black precipitates and suitable for SC-XRD.

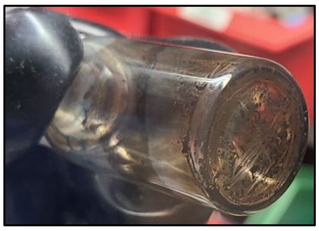

Analytical Data:

^{1}H NMR (500 MHz, C_6D_6, 298 K) ***δ***: 2.00 (s, 15H, CH_3 of Cp*), 1.85–1.82 (m, 2H, CH_2 of TMP), 1.50–1.47 (m, 4H, CH_2 of TMP), 1.40 (s, 12H, CH_3 of TMP), 1.25 (br s, 12H CH_3 of TMP)* ppm. *Superimposed with CH_2-multiplet of TMP. **^{13}C{^{1}H} NMR** (126 MHz, C_6D_6, 298 K) ***δ***: 118.1 (s, $^{2}J_{CdC}$ = 21 Hz (*Cd-satellites*), C_q of Cp*), 54.9 (s, J_{CdC} = 6.4 Hz (*Cd-satellites*), C_q of TMP), 53.1 (s, C_q of TMP), 42.0 (s, *C*H_2 of TMP), 41.0 (s, J_{CdC} = 7.9 Hz (*Cd-satellites*), *C*H_2 of TMP), 37.4 (s, J_{CdC} = 9.6 Hz (*Cd-satellites*), 35.3+31.8 (br s, *C*H_3 of TMP), 20.4 (s, *C*H_2 of TMP), 19.2 (s, *C*H_2 of TMP), 12.0 (s, *C*H_3 of Cp*) ppm. **^{27}Al NMR** (130 MHz, C_6D_6, 298 K) ***δ***: no resonance observed in a range of +300 – -300 ppm. **^{113}Cd NMR** (111 MHz, C_6D_6, 298 K) ***δ***: no resonance observed in a range of +850 – -2000 ppm.

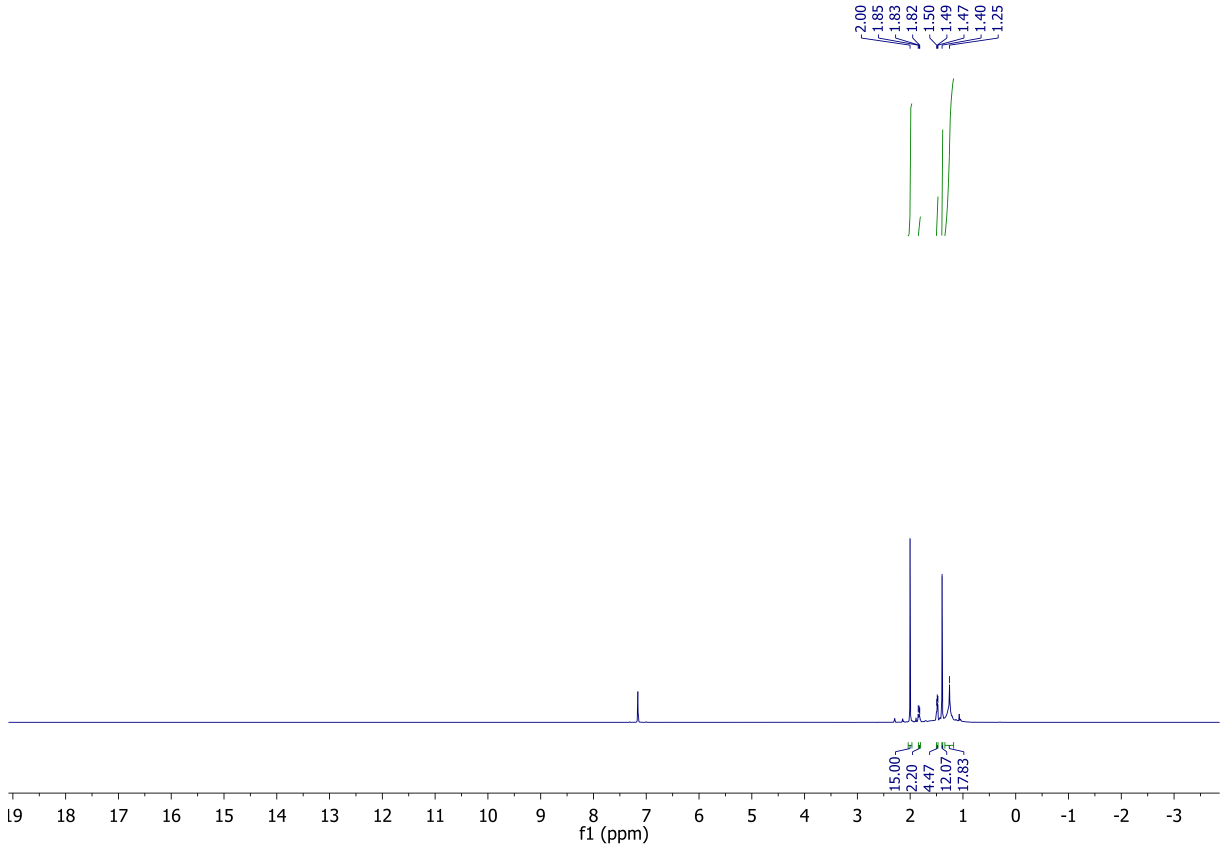

Figure S22: ^{1}H NMR (500 MHz, C_6D_6) of **4**.

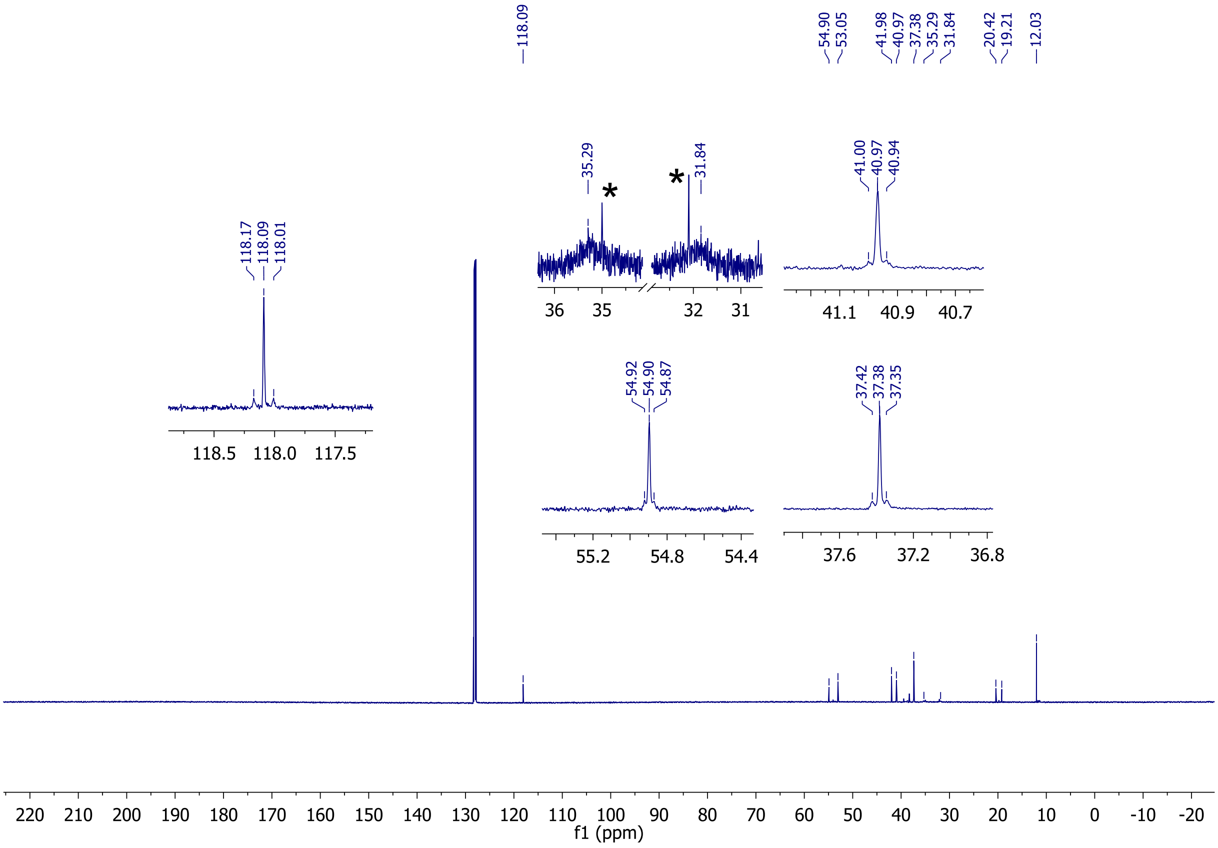

Figure S23: $^{13}C\{^{1}H\}$ NMR (126 MHz, C_6D_6) of **4**. * = trace of an unidentified side and/or decomposition product.

3.4 [{N(TMS)$_2$}Ag(P^{t}Bu$_3$)] (^{pre}**5**)

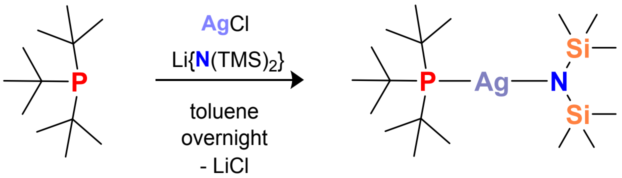

In the glovebox, P^{t}Bu$_3$ (3.66 mmol, 0.741 g, 1.05 eq), AgCl (3.48 mmol, 0.5 g, 1 eq.), and Li{N(TMS)$_2$} (3.68 mmol, 0.615 g, 1 eq.) were placed in an ampoule with PTFE valve (FengTecEx). Then, the mixture was suspended in 30 mL of toluene and stirred overnight in the dark (*using tinfoil).* The obtained suspension is subsequently filtered via cannula filtration and the solvent is removed under reduced pressure. 10 mL of n-hexane are added to the solution followed by another cannula filtration. The solvent is removed under reduced pressure to obtain a crystalline, analytically clean powder of ^{pre}**5**. After isolation in the glovebox, 1.255 g were obtained (79%). Suitable crystals for X-ray diffraction were obtained from a saturated *n*-pentane solution in the cold when placed at –30°C in a glovebox integrated freezer.

^{1}H NMR (500 MHz, C_6D_6, 298 K) ***δ***: 1.04 (d, $^3J_{PH}$ = 13.0 Hz, 27H, –C*H*$_3$ of tBu$_3$P), 0.57 (s, 18H, –C*H*$_3$ of HMDS) ppm. **^{13}C{^{1}H} NMR** (126 MHz, C_6D_6, 298 K) ***δ***: 37.0 (d, $^1J_{PC}$ = 4.3 Hz, (*C*(CH$_3$)$_3$)$_3$ of ^{t}Bu$_3$P), 32.2 (d, $^2J_{PC}$ = 7.3 Hz, (C(*C*H$_3$)$_3$)$_3$ of ^{t}Bu$_3$P), 7.6 (s, *C*H$_3$ of HMDS) ppm. **^{29}Si{^{1}H} NMR** (100 MHz, C_6D_6, 298 K) ***δ***: −8.8 (s, *Si* of HMDS) ppm. **^{31}P{^{1}H} NMR** (202 MHz, C_6D_6) ***δ***: 78.29 (dd, $^1J_{107AgP}$ = 495.4 Hz; $^1J_{109AgP}$ = 571.2 Hz, ^{t}Bu$_3$*P*) ppm. **Elemental Analysis** calc. for $C_{18}H_{45}Ag_1N_1P_1Si_2$ (found) C 45.94 (45.38), H 9.64 (9.86), N 2.98 (2.40).

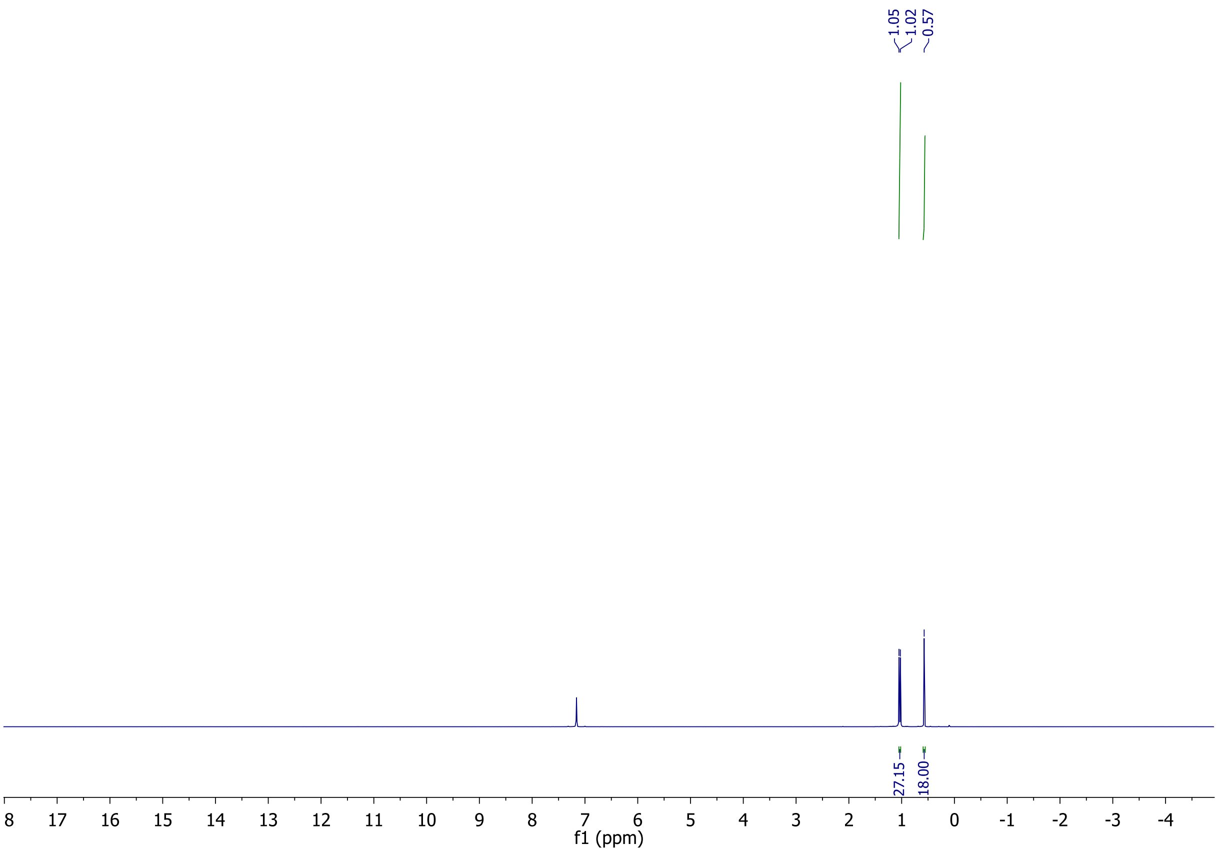

Figure S24: ^{1}H NMR (500 MHz, C_6D_6) of ***^{pre}5***.

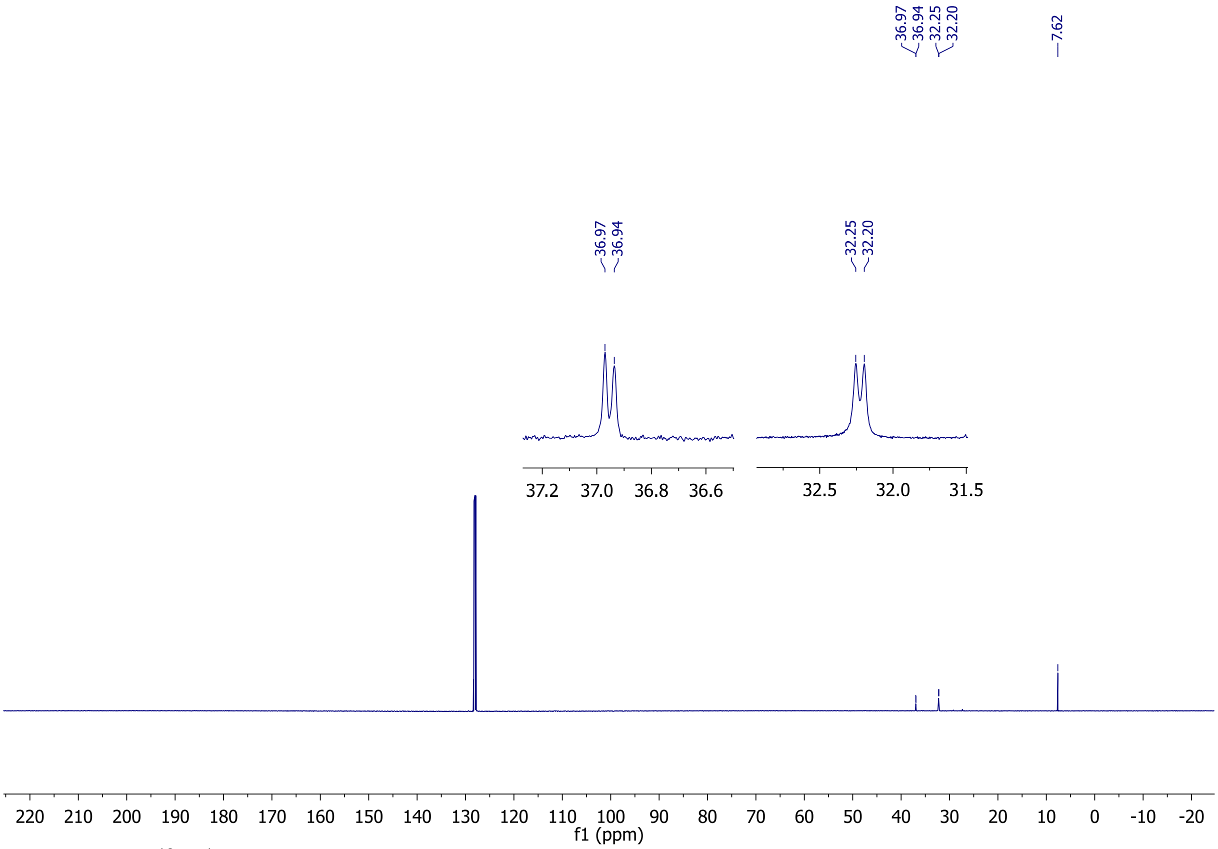

Figure S25: $^{13}C\{^{1}H\}$ NMR (126 MHz, C_6D_6) of ***^{pre}5***.

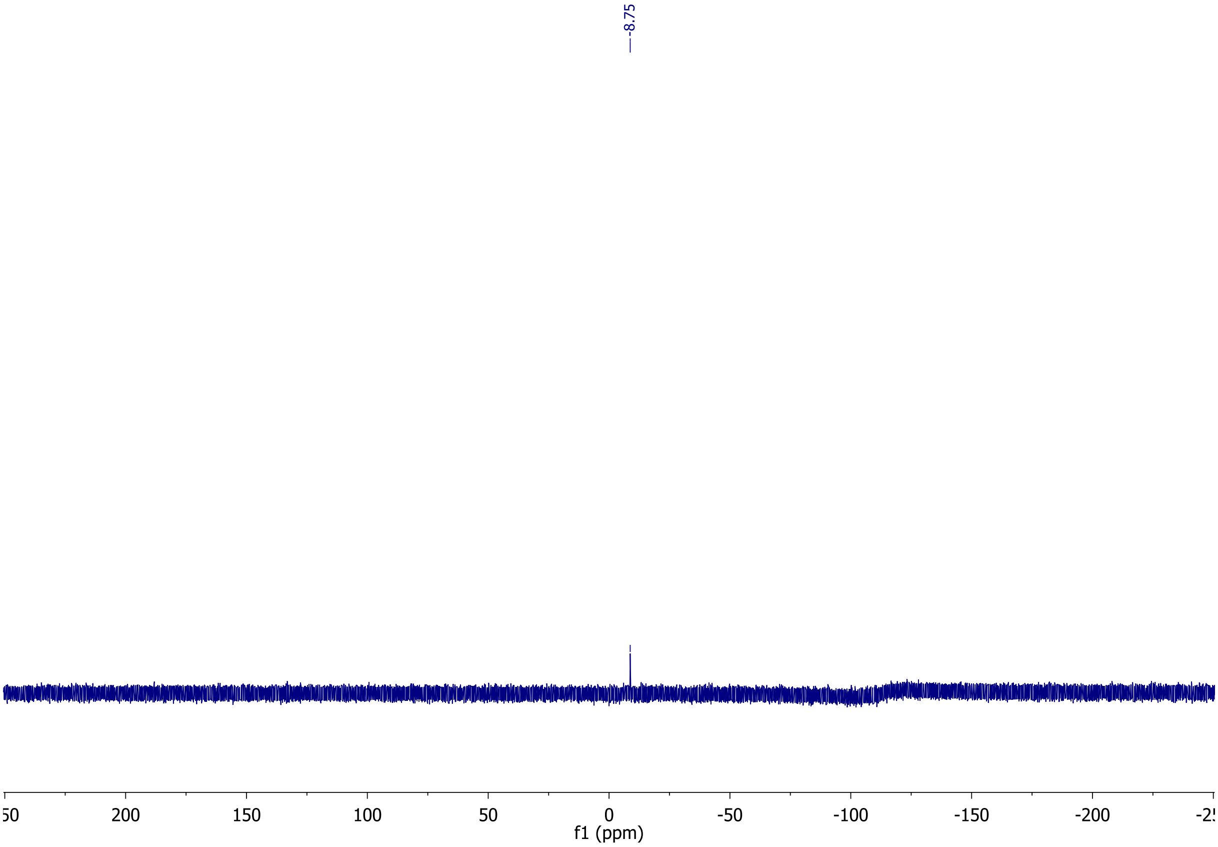

Figure S26: $^{29}Si\{^{1}H\}$ NMR (99 MHz, C_6D_6) of ***pre*5**.

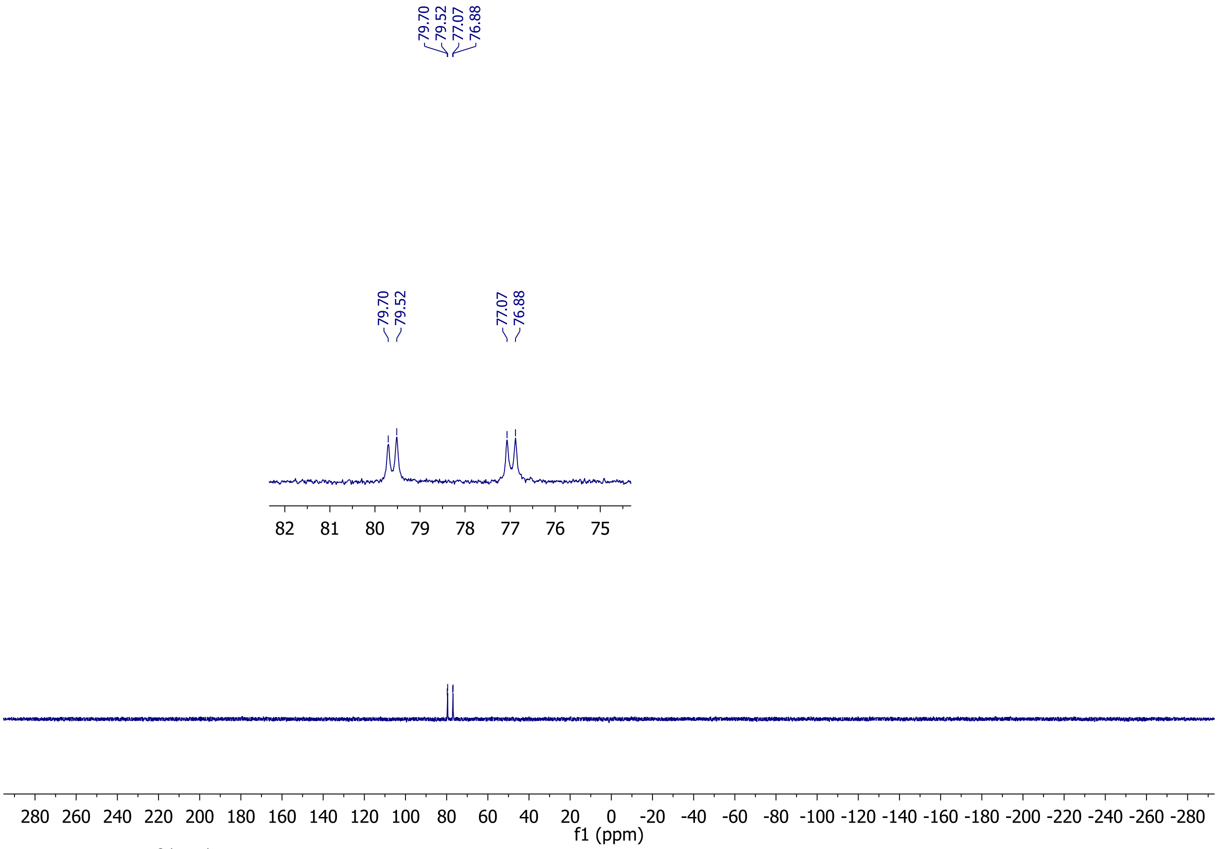

Figure S27: $^{31}P\{^{1}H\}$ NMR (99 MHz, C_6D_6) of ***pre*5**.

3.5 [{N(TMS)$_2$}(Cp*)Al–Ag(P^{t}Bu$_3$)] (**5**)

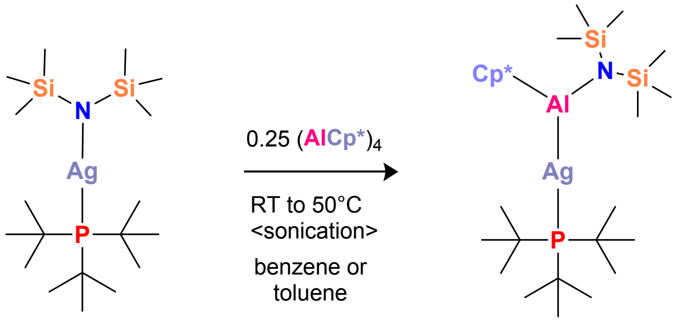

In an ampoule with PTFE valve (FengTecEx), 36.0 mg of finely ground [Cp*Al]$_4$ (0.23 mmol; a 1.1 eq. of "AlCp*") and 100 mg of [{N(TMS)$_2$}Ag(PtBu$_3$)] (***pre*5**; 0.21 mmol) were suspended in 5 mL of toluene. To convert the [Cp*Al]$_4$, the mixture was sonicated at 50°C for twenty minutes and afterwards treated thermally without sonication for an additional period of ten minutes to give a brown solution. Next, the solvent was removed under reduced, the light-brown residue extracted with 20 mL of *n*-pentane and cannula filtered. The solvent is removed again under reduced pressure to obtain crude **5** (71 mg, 53%) as a light-brown powder. Attempts to recrystallize the compound afforded crystals suitable for SC-XRD but these proved extremely sensitive due to the formation of elemental silver (<12 h in the glovebox).

**We provide the best fitting elemental analysis data of the product obtained to date.*

^{1}H NMR (500 MHz, C_6D_6, 298 K) ***δ***: 2.26 (s, 15H, CH_3 of Cp*), 1.12 (d, $^3J_{PH}$ = 12.1 Hz, 27H, –CH_3 of tBu$_3$P), 0.48 (s, 18H, –CH_3 of HMDS) ppm. **^{13}C{^{1}H} NMR** (126 MHz, C_6D_6, 298 K) ***δ***: 116.0 (d, $^3J_{CP}$ = 4.2 Hz, C_q of Cp*), 37.1 (br s, (C(CH$_3$)$_3$)$_3$ of ^{t}Bu$_3$P), 32.3 (br s, (C(CH$_3$)$_3$)$_3$ of ^{t}Bu$_3$P), 12.5 (s, CH$_3$ of Cp*), 6.3+6.3 (s, CH$_3$ of HMDS) ppm. **^{29}Si{^{1}H} NMR** (100 MHz, C_6D_6, 298 K) ***δ***: −5.2 (s, *Si* of HMDS) ppm. **^{31}P{^{1}H} NMR** (202 MHz, C_6D_6) ***δ***: 61.3 (br d, *J* = 158 Hz; ^{t}Bu$_3$*P*) ppm. **Elemental Analysis** calc. for $C_{28}H_{60}Ag_1Al_1N_1P_1Si_2$ (found) C 53.15 (51.22), H 9.56 (9.40), N 2.21 (1.05).*

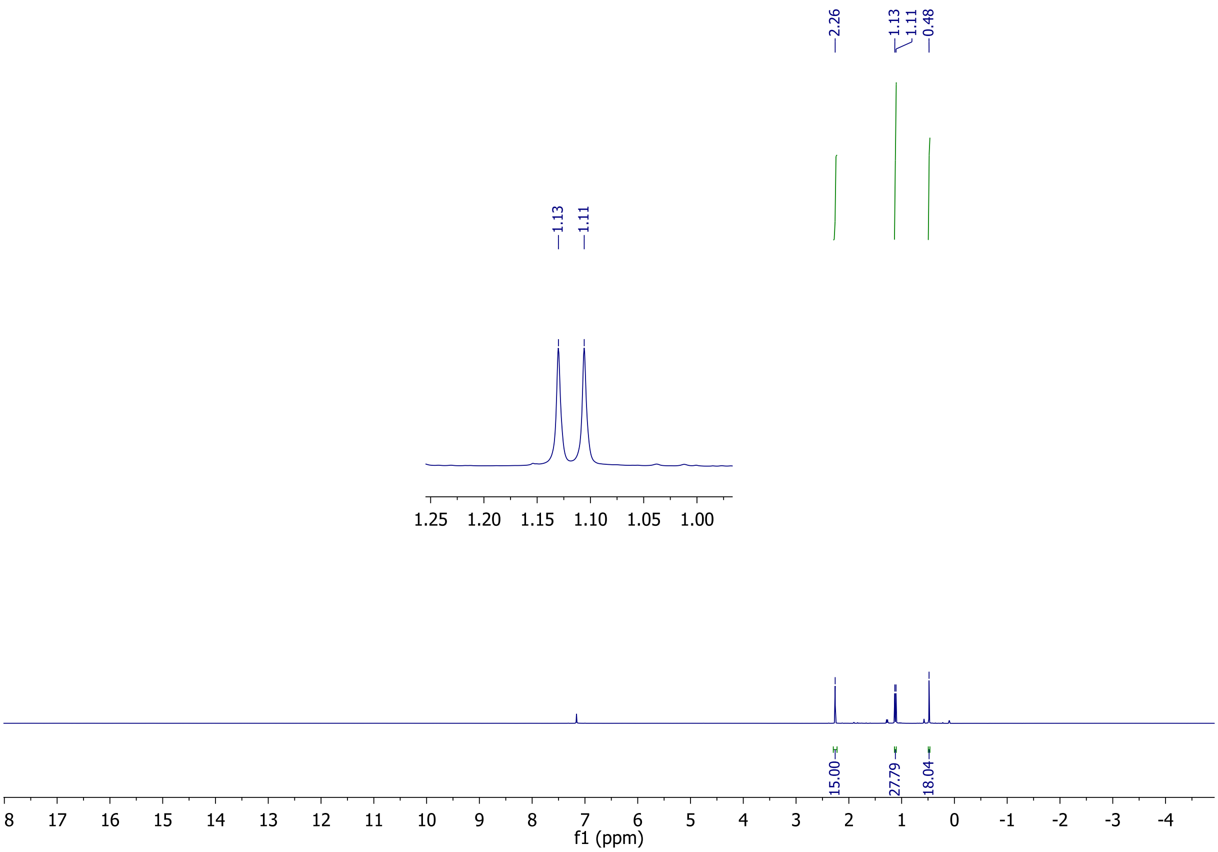

Figure S28: ^{1}H NMR (500 MHz, C_6D_6) of **5**.

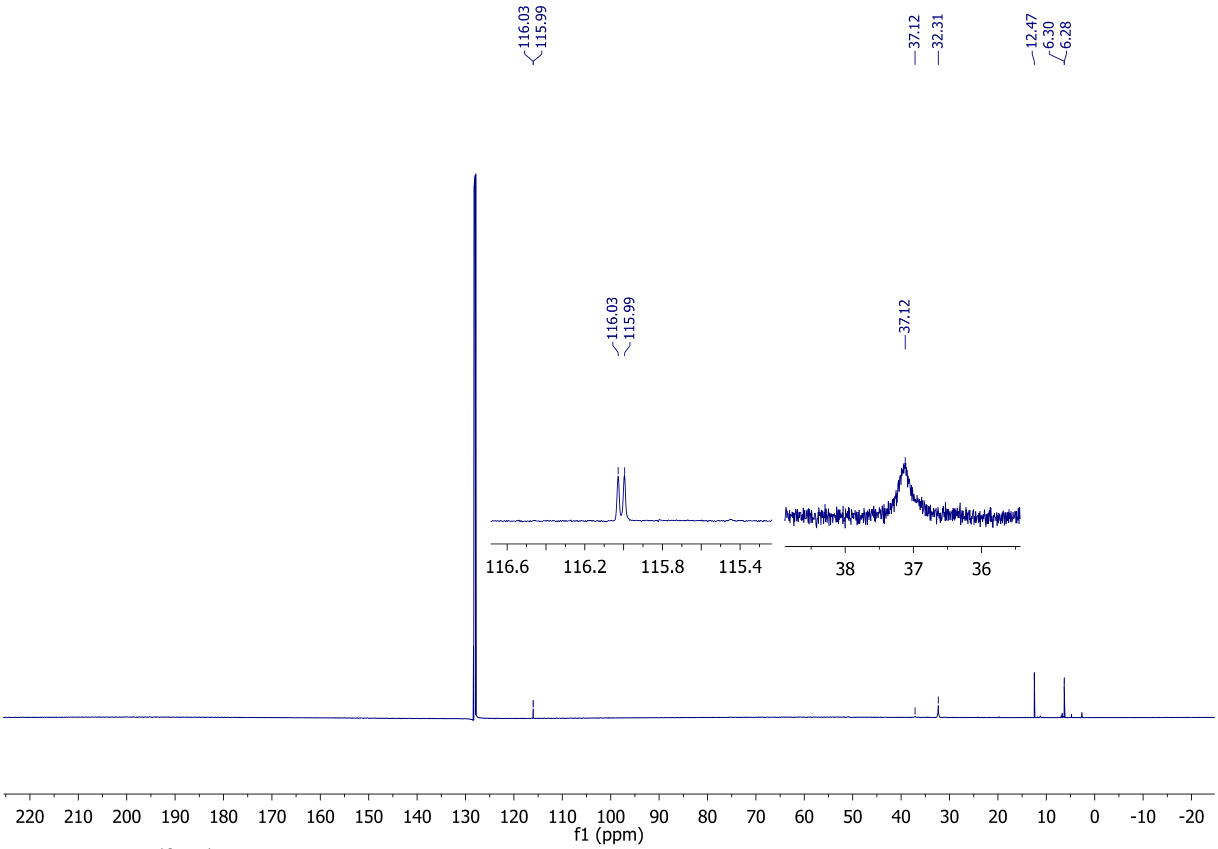

Figure S29: $^{13}C\{^{1}H\}$ NMR (126 MHz, C_6D_6) of **5**.

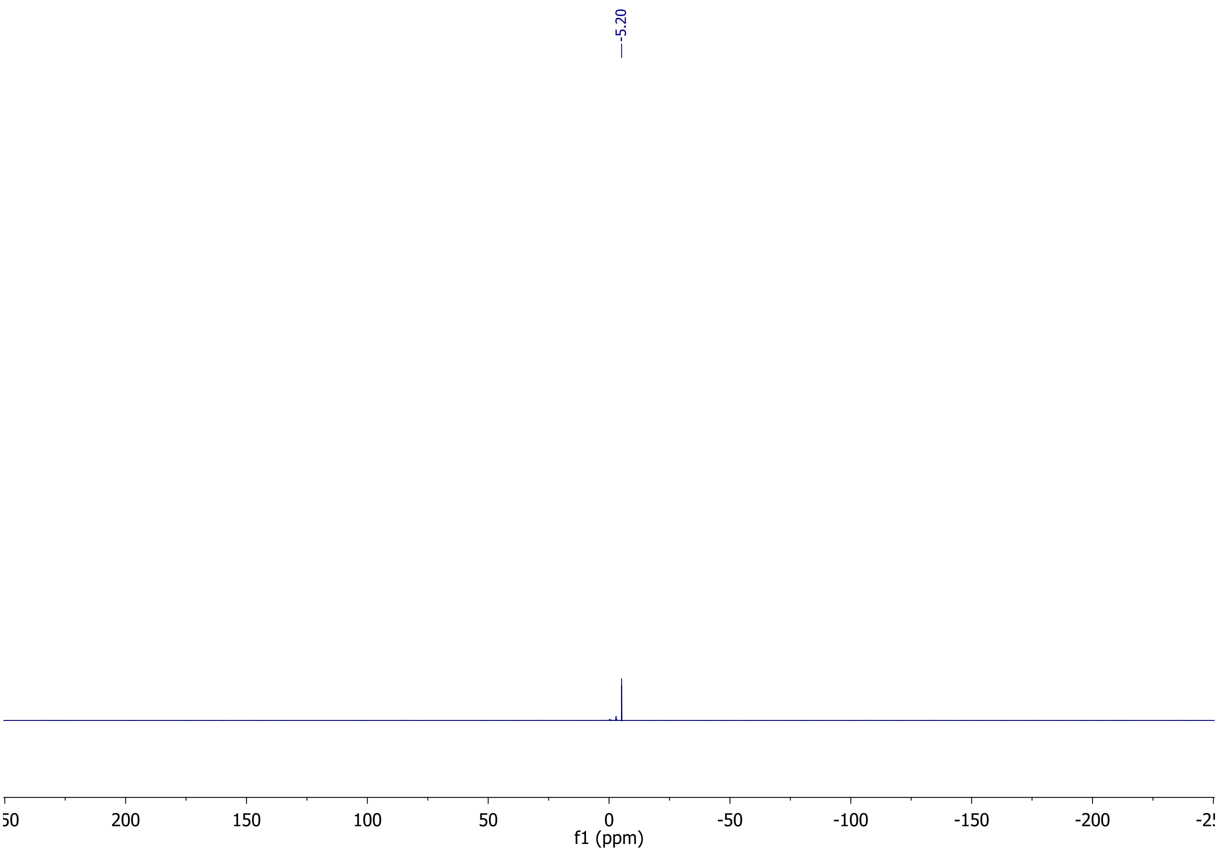

Figure S30: $^{29}Si\{^1H\}$ NMR (99 MHz, C_6D_6) of **5**.

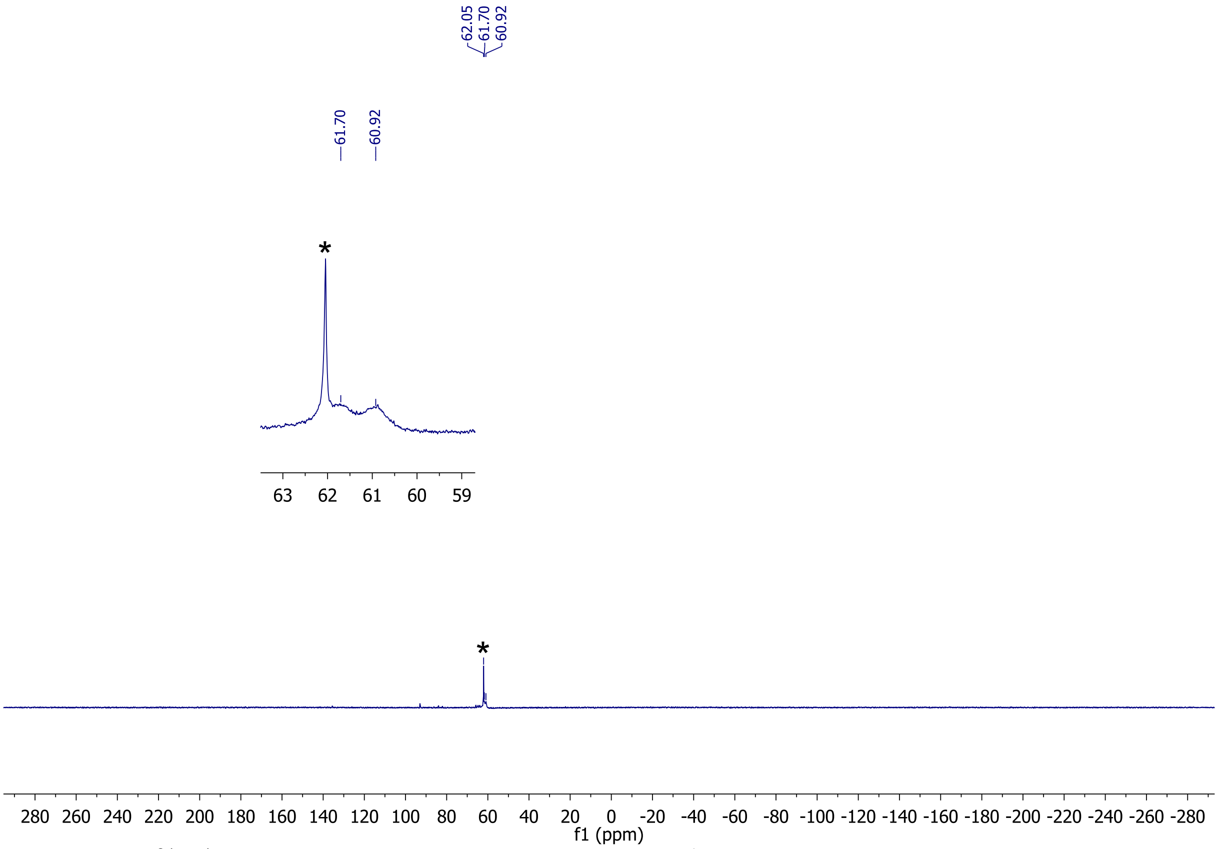

Figure S31: $^{31}P\{^1H\}$ NMR (99 MHz, C_6D_6) of **5**. * = trace P^tBu_3.

3.6 [{N(TMS)$_2$}(Cp*)Al{(N^{i}Pr)$_2$C}Cd{N(TMS)$_2$}] (6a)

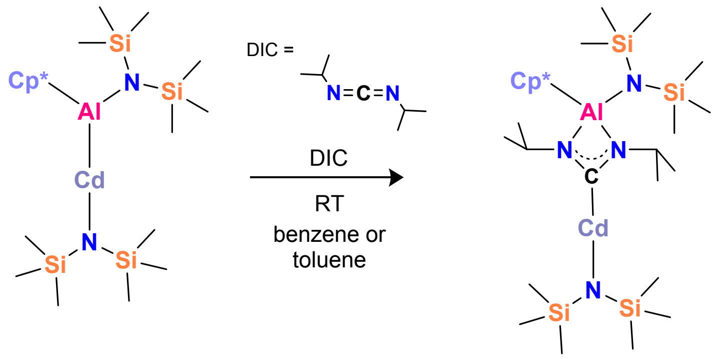

In an ampoule with PTFE valve (FengTecEx), 18.7 mg of finely ground [Cp*Al]$_4$ (0.029 mmol; a 1.0 eq. of “AlCp*”) and 50 mg Cd{N(TMS)$_2$}$_2$ (0.115 mmol, 1 eq.) were suspended in 4 mL of toluene. To convert the [Cp*Al]$_4$, the mixture was sonicated at 50°C for twenty minutes and afterwards treated thermally without sonication for an additional period of ten minutes. Next, DIC (16 µL; 13.1 mg, 0.104 mmol, 0.9 eq.) was dissolved in 1 mL of toluene and added to the grey-metallic solution. Immediately after mixing, the solvent is removed under reduced pressure. The grey residue is extracted with 4 mL of *n*-pentane and subsequently filtered through a PTFE syringe filter (d = 13 mm; pore size 0.22 µm) inside the glovebox. Slow evaporation yields colorless crystals of **6a** (yield: 71 mg, 87%). Note: Depending on the evaporation rate, the compound can be obtained as a colorless oil, which can be solidified upon dissolving in neat tetramethylsilane (TMS) followed by lyophilization.

^{1}H NMR (500 MHz, C_6D_6, 298 K) ***δ***: 3.26 (hept, $^3J_{HH}$ = 6.8, 2H, (−C*H* of ^{i}Pr), 1.96 (s, 15H, −C*H*$_3$ of Cp*), 1.20 (ps t, $^3J_{HH}$ = 7.1 Hz, 24H, −C*H*$_3$ of ^{i}Pr),* 0.32 (s, 18H, −CH$_3$ of HMDS), 0.21 (s, 18H, −CH$_3$ of HMDS) ppm. * = two overlapping doublets as a pseudo triplet. **^{13}C{^{1}H} NMR** (126 MHz, C_6D_6, 298 K) ***δ***: 196.4 (s, *C*N$_2$ of DIC), 119.8 (s, C_q of Cp*), 50.9 (s, $^3J_{CdC}$ = 26 Hz (*Cd-satellites*), −CH of ^{i}Pr), 27.1 (s, −*C*H$_3$ of ^{i}Pr), 25.9 (s, −*C*H$_3$ of ^{i}Pr), 13.7 (s, −*C*H$_3$ of Cp*), 5.8 (−*C*H$_3$ of HMDS), 5.7 (−*C*H$_3$ of HMDS) ppm. **^{27}Al NMR** (130 MHz, C_6D_6, 298 K) ***δ***: 115 (s (br), $\omega_{1/2}$ = 3640 Hz) ppm. **^{29}Si{^{1}H} NMR** (100 MHz, C_6D_6, 298 K) ***δ***: 0.0 (s, *Si* of HMDS), −2.7 (s, *Si* of HMDS) ppm. **^{113}Cd NMR** (111 MHz, C_6D_6, 298 K) ***δ***: no resonance observed in a range of +850 – -2000 ppm. **Elemental Analysis** calc. for $C_{29}H_{65}Al_1Cd_1N_4Si_4$ (found) C 48.27 (47.68), H 9.08 (9.05), N 7.76 (7.47).

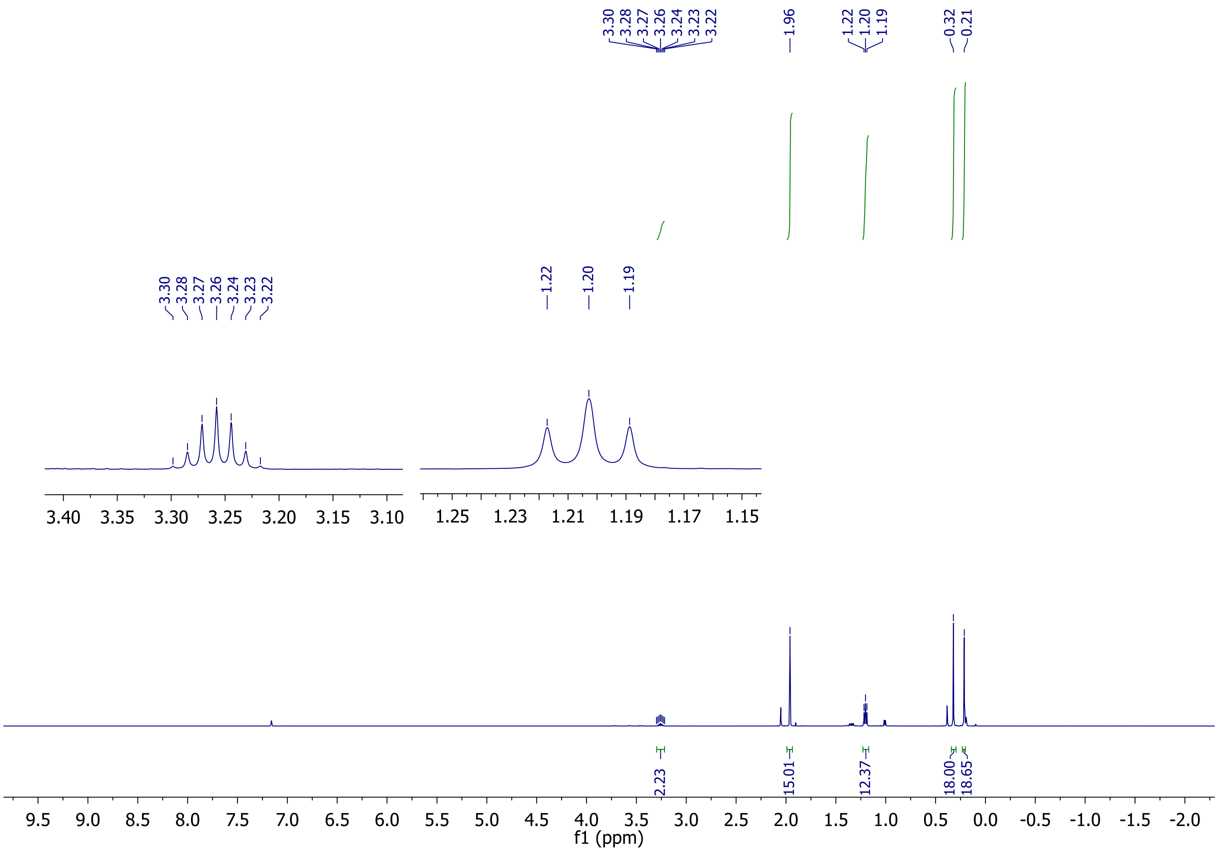

Figure S32: ^{1}H NMR (500 MHz, C_6D_6) of **6a**.

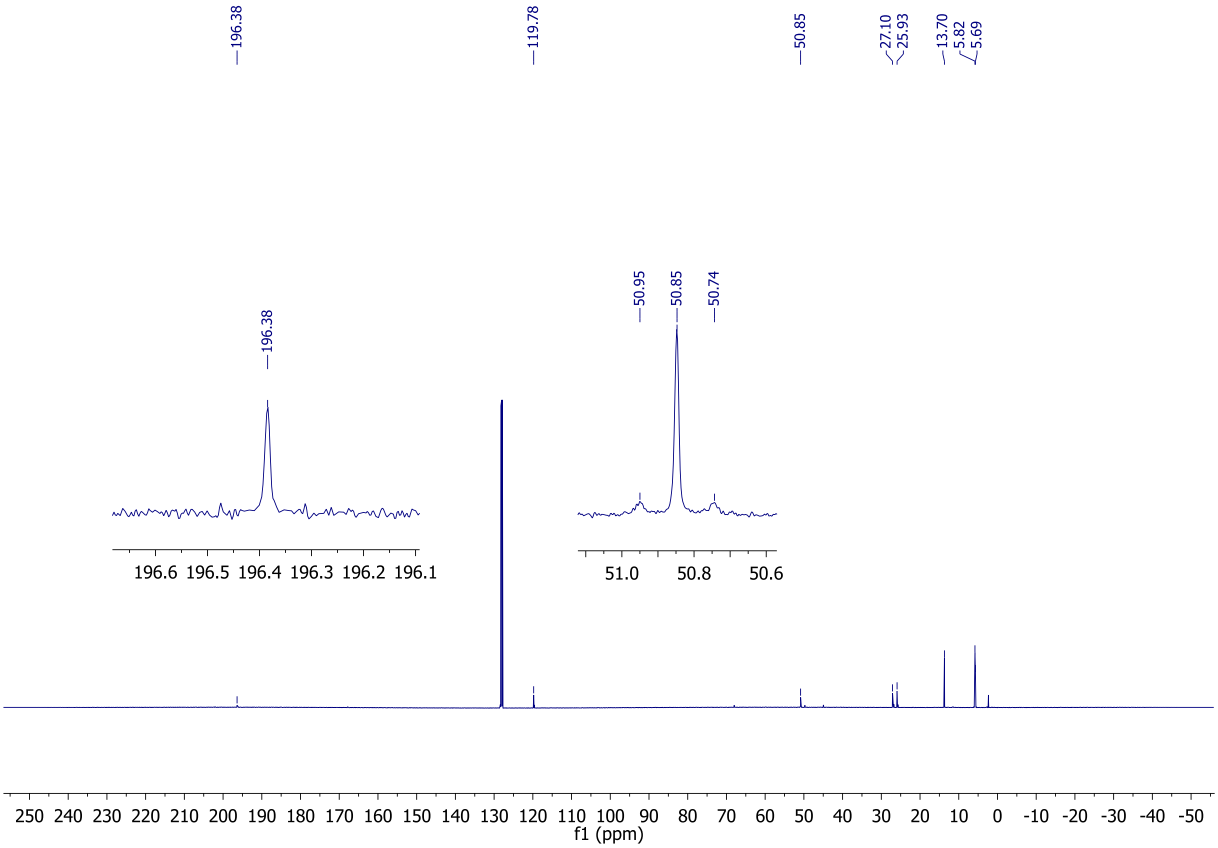

Figure S33: $^{13}C\{^{1}H\}$ NMR (126 MHz, C_6D_6) of **6a**.

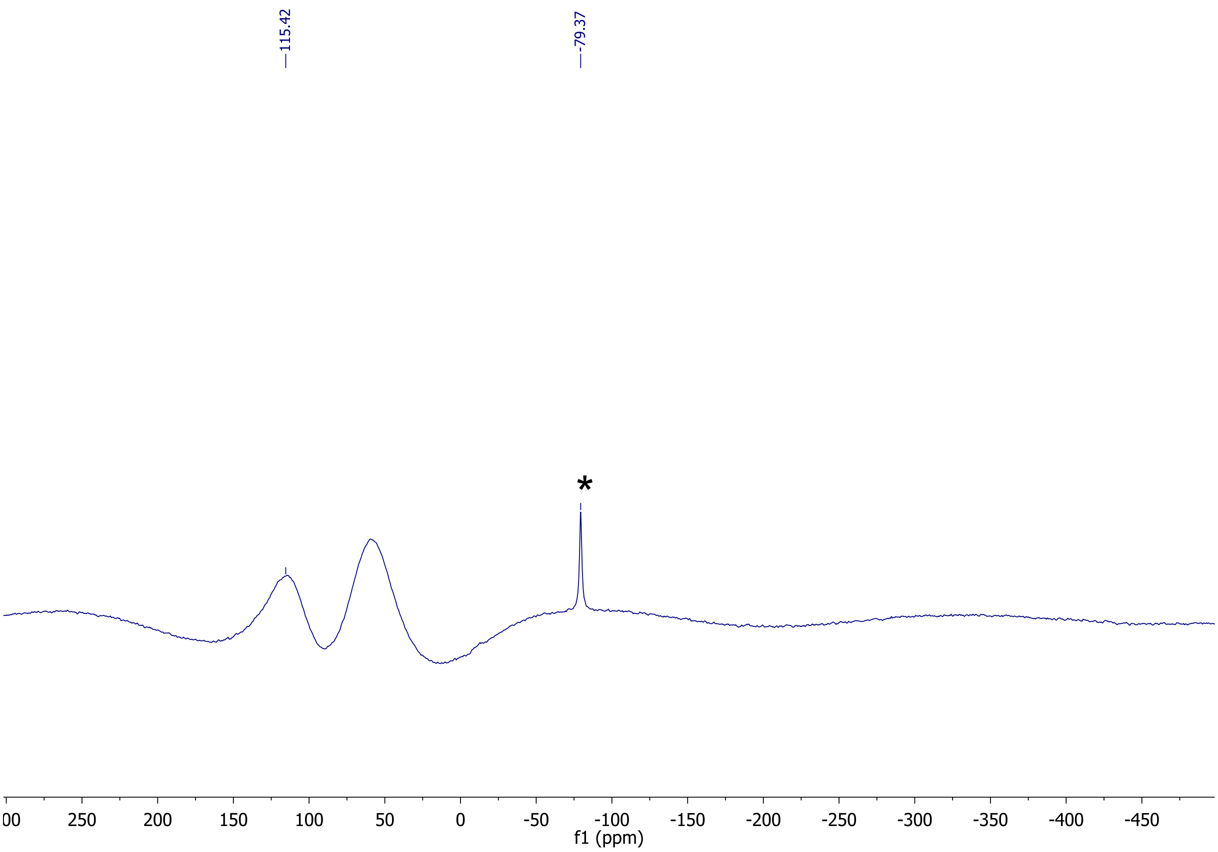

Figure S34: ^{27}Al NMR (130 MHz, C_6D_6) of **6a**. Resonance at ~50 ppm = probe head; * = trace $(AlCp^*)_4$ as internal standard.

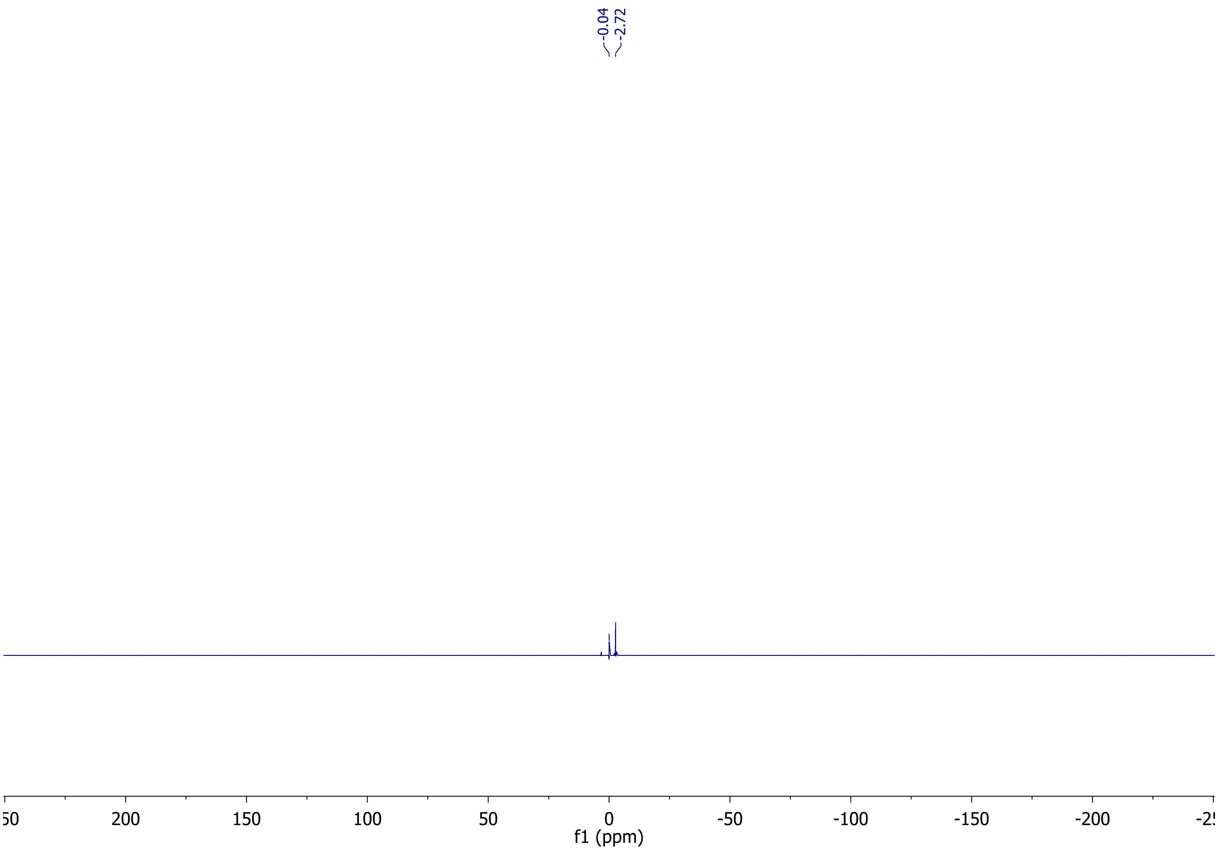

Figure S35: $^{29}Si\{^1H\}$ NMR (99 MHz, C_6D_6) of **6a**.

3.7 [{N(TMS)$_2$}(Cp*)Al{(Cy)$_2$C}Cd{N(TMS)$_2$}] (**6b**)

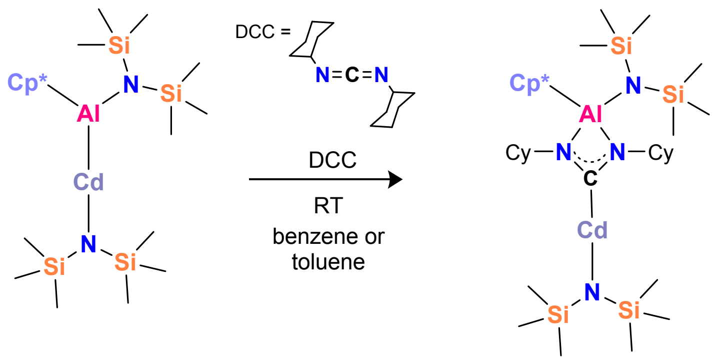

In an ampoule with PTFE valve (FengTecEx), 18.7 mg of finely ground [Cp*Al]$_4$ (0.029 mmol, a 1.0 eq. of AlCp*) and 50 mg Cd{N(TMS)$_2$}$_2$ (0.115 mmol, 1 eq.) were suspended in 4 mL of toluene. To convert the [Cp*Al]$_4$, the mixture was sonicated at 50°C for twenty minutes and afterwards treated thermally without sonication for an additional period of ten minutes. Next, DCC (21.4 mg, 0.104 mmol, 0.9 eq.) was dissolved in 1 mL of toluene and added to the grey-metallic solution. Immediately after mixing, the solvent is removed under reduced pressure. The grey residue is extracted with 4 mL of *n*-pentane and subsequently filtered through a PTFE syringe filter (d = 13 mm; pore size 0.22 µm) inside the glovebox. Slow evaporation of the solvent yields colorless crystals of **6b** (yield: 82 mg, 91%). Note: Depending on the evaporation rate, the compound can be obtained as a colorless oil, which can be solidified with neat tetramethylsilane (TMS) and subsequent lyophilization.

^{1}H NMR (500 MHz, C_6D_6, 298 K) ***δ***: 2.86 (t, $^3J_{HH}$ = 12.0 Hz, 2H, –CH of Cy), 2.08+2.06 (s, 2H, –C*H*$_2$ of Cy), 1.97+1.93 (s, 17H, –C*H*$_2$ of Cy; –C*H*$_3$ of Cp*), 1.67–1.61 (m, 4H, –C*H*$_2$ of Cy), 1.52–1.36 (m, 6H, –C*H*$_2$ of Cy), 1.14–0.99 (m, 6H, –C*H*$_2$ of Cy), 0.29 (s, 18H, C*H*$_3$ of HMDS), 0.16 (s, 18H, C*H*$_3$ of HMDS) ppm. **^{13}C{^{1}H} NMR** (126 MHz, C_6D_6, 298 K) ***δ***: 196.5 (s, *C*N$_2$ of DCC), 119.8 (s, Cq of Cp*), 59.5 (s, $^3J_{CdC}$ = 26 Hz (*Cd-satellites*), –*C*H of Cy), 38.4 (br s, –*C*H$_2$ of Cy), 37.8 (s, –*C*H$_2$ of Cy), 26.9 (br s, –*C*H$_2$ of Cy), 26.6 (s, –*C*H$_2$ of Cy), 26.3 (s, –*C*H$_2$ of Cy), 13.7 (s, –*C*H$_3$ of Cp*), 5.9 (s, –*C*H$_3$ of HMDS), 5.7 (s, –*C*H$_3$ of HMDS) ppm. **^{27}Al NMR** (130 MHz, C_6D_6, 298 K) ***δ***: no resonance observed in a range of +300 – -300 ppm. **^{29}Si{^{1}H} NMR** (100 MHz, C_6D_6, 298 K) ***δ***: 0.0 (s, *Si* of HMDS), −2.7 (s, *Si* of HMDS) ppm. **^{113}Cd NMR** (111 MHz, C_6D_6, 298 K) ***δ***: no resonance observed in a range of +850 – -2000 ppm. **Elemental Analysis** calc. for $C_{35}H_{73}Al_1Cd_1N_4Si_4$ (found) C 52.43 (51.56), H 9.18 (9.20), N 6.99 (6.56).

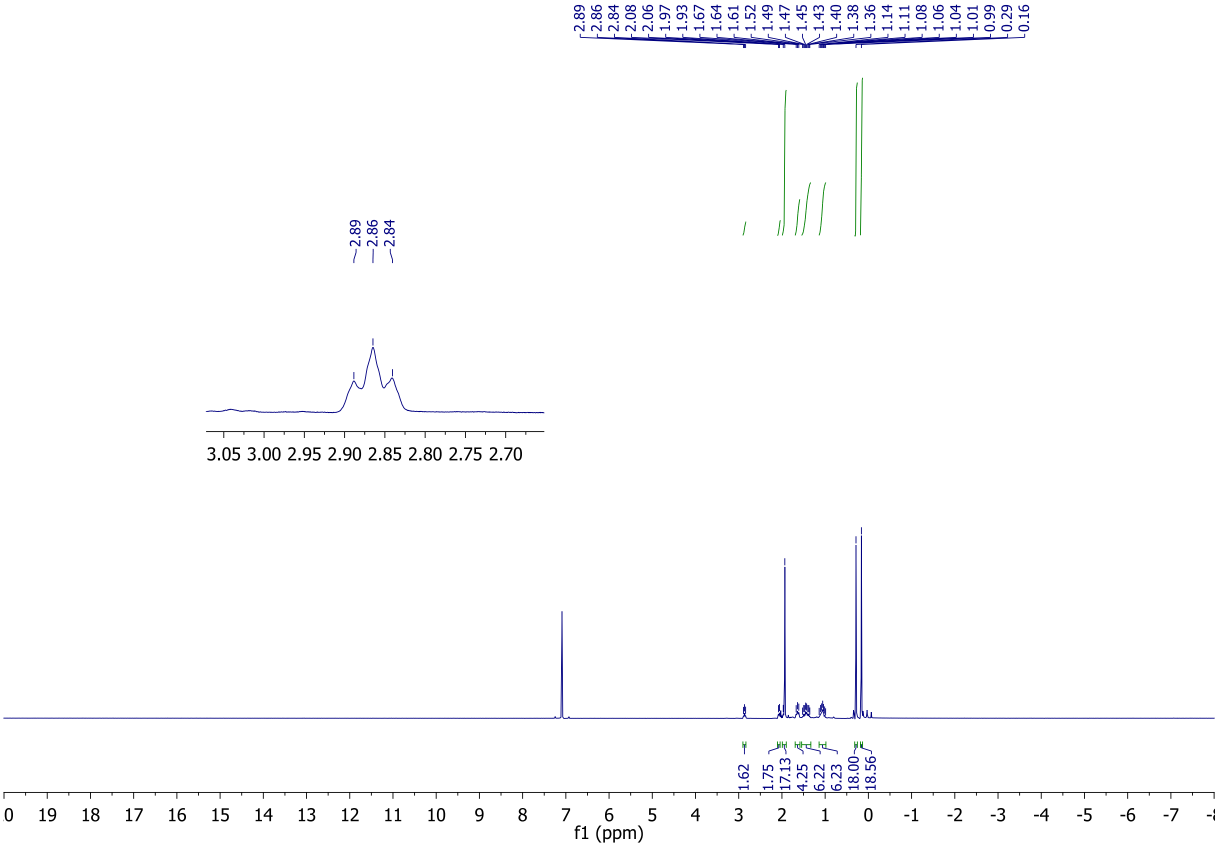

Figure S36: ^{1}H NMR (500 MHz, C_6D_6) of **6b**.

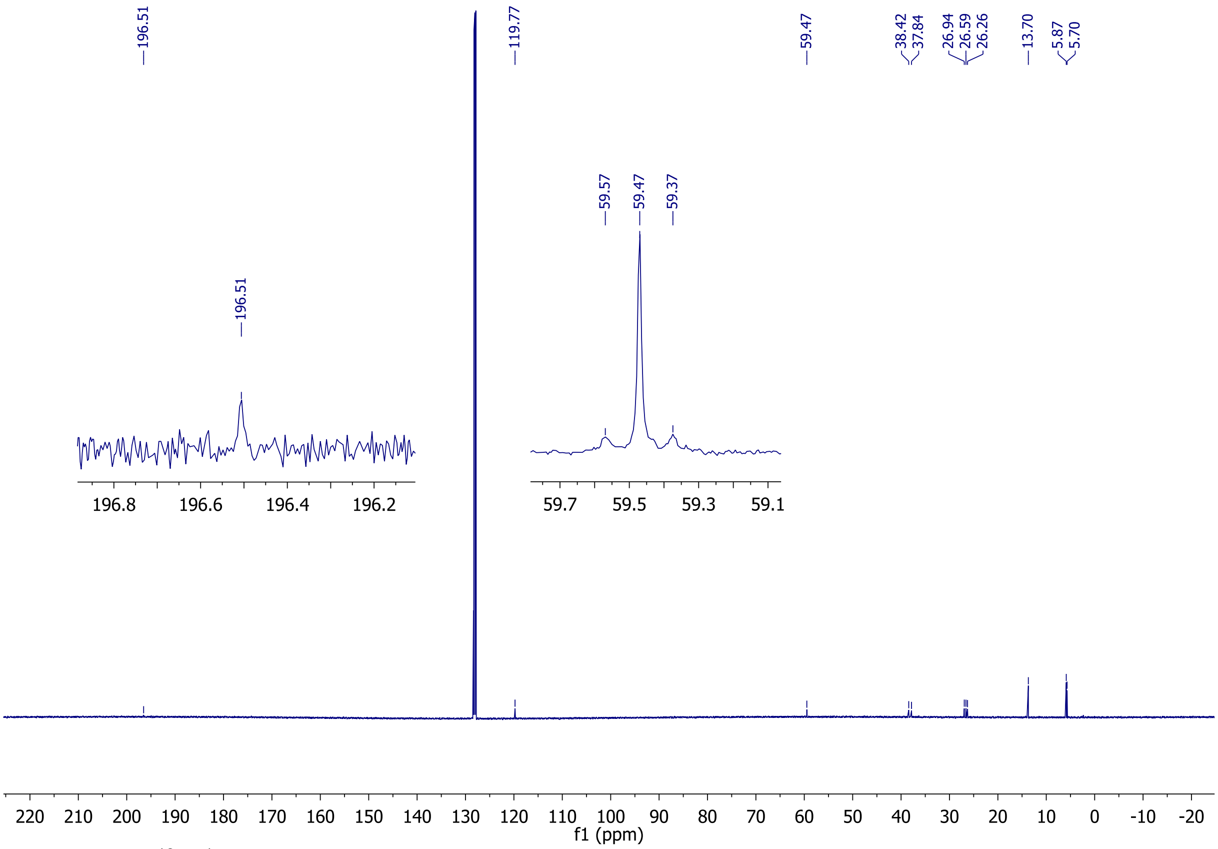

Figure S37: $^{13}C\{^1H\}$ NMR (126 MHz, C_6D_6) of **6b.**

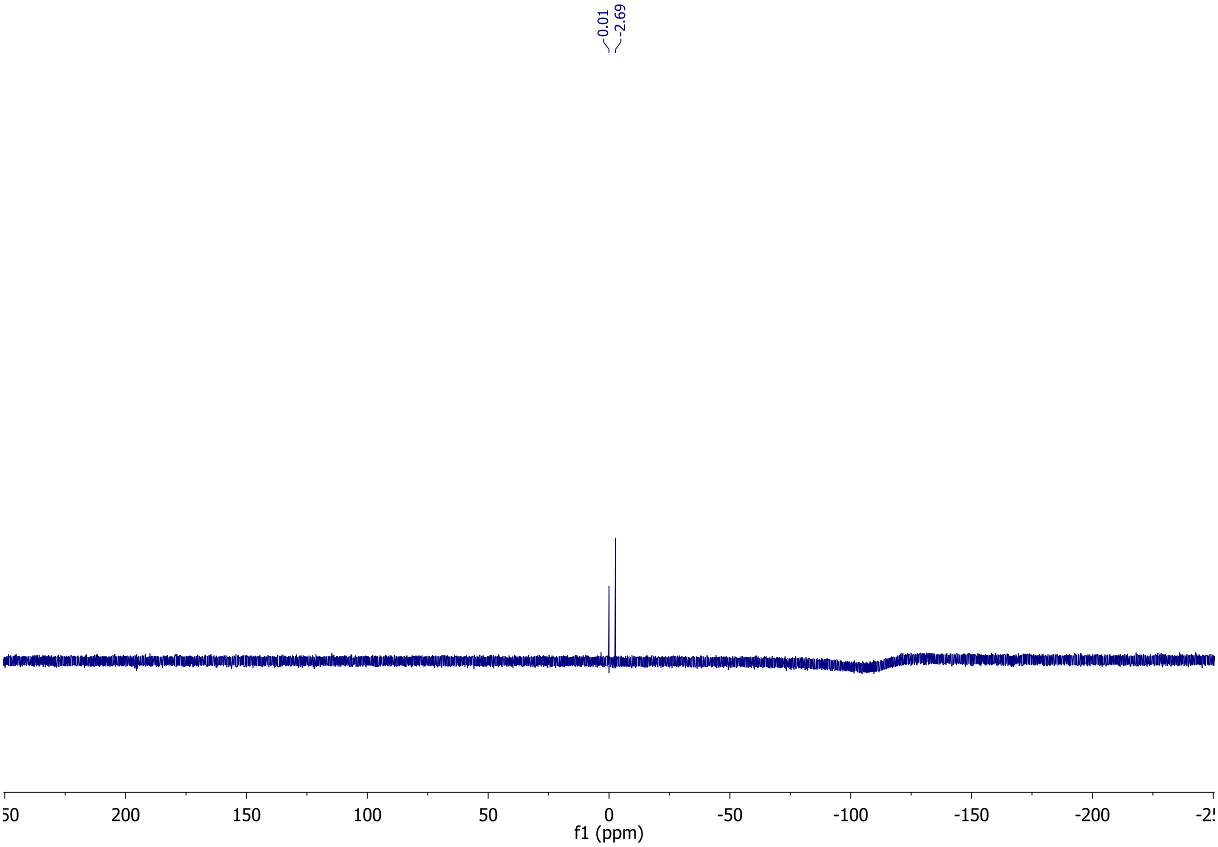

Figure S38: $^{29}Si\{^{1}H\}$ NMR (99 MHz, C_6D_6) of **6b**.

3.8 [{N(TMS)$_2$}(Cp*)Al{(N^{i}Pr)$_2$C}Cd{(N^{i}Pr)$_2$C–N(TMS)$_2$}] (7a)

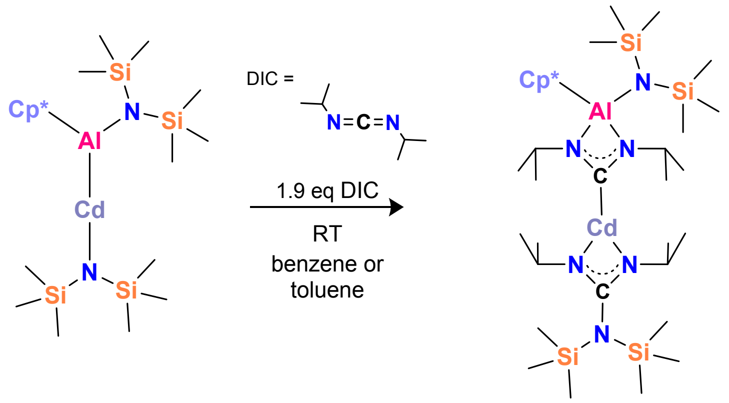

In an ampoule with PTFE valve (FengTecEx), 20.0 mg of finely ground [Cp*Al]$_4$ (0.031 mmol; 1.1 eq. of “AlCp*”) and 50 mg Cd{N(TMS)$_2$}$_2$ (0.115 mmol, 1 eq.) were suspended in 4 mL of toluene. To convert the [Cp*Al]$_4$, the mixture was sonicated at 50°C for twenty minutes and afterwards treated thermally without sonication for an additional period of ten minutes. Next, DIC (33.8 µL; 27.6 mg, 0.218 mmol, 1.9 eq.) was dissolved in 1 mL of toluene and added to the grey-metallic solution. Immediately after mixing, the solvent is removed under reduced pressure. The grey residue is extracted with 4 mL of *n*-pentane and subsequently filtered through a PTFE syringe filter (d = 13 mm; pore size 0.22 µm) inside the glovebox. Slow evaporation of the solvent furnishes colorless crystals of **7a** (yield: 89 mg, 91%).

^{1}H NMR (500 MHz, C_6D_6, 298 K) ***δ***: 3.79–3.65 (m, 2H, (−C*H* of ^{i}Pr), 3.46 (hept, $^3J_{HH}$ = 6.8 Hz, 2H, −C*H* of ^{i}Pr), 2.05 (s, 15H, −C*H*$_3$ of Cp*), 1.36+1.33* (d, $^3J_{HH}$ = 6.8 Hz, 12H, −C*H*$_3$ of ^{i}Pr), 1.01 (d, $^3J_{HH}$ = 6.1 Hz, 12H, −CH$_3$ of ^{i}Pr), 0.38 (s, 18H, −CH$_3$ of HMDS), 0.21 (s, 18H, −CH$_3$ of HMDS) ppm. * = two overlapping doublets. **^{13}C{^{1}H} NMR** (126 MHz, C_6D_6, 298 K) ***δ***: 202.4 (s, Cd-*C*N$_2$ of DIC), 167.8 (s, N-*C*N$_2$ of DIC), 119.6 (s, *C*$_q$ of Cp*), 49.8 (s, $^3J_{CdC}$ = 24.0 Hz (*Cd-satellites*), −*C*H of ^{i}Pr), 49.8 (s, −*C*H of ^{i}Pr), 27.0 (s, −*C*H$_3$ of ^{i}Pr), 26.7 (s, −*C*H$_3$ of ^{i}Pr), 25.9 (s, −*C*H$_3$ of ^{i}Pr), 13.7 (s, −*C*H$_3$ of Cp*), 5.8 (−*C*H$_3$ of HMDS), 2.3 (−*C*H$_3$ of HMDS) ppm. **^{27}Al NMR** (130 MHz, C_6D_6, 298 K) ***δ***: 111.0 (s (br), $\omega_{1/2}$ = 3674 Hz) ppm. **^{29}Si{^{1}H} NMR** (100 MHz, C_6D_6, 298 K) ***δ***: 3.19 (s, *Si* of HMDS), −3.0 (s, *Si* of HMDS) ppm. **^{113}Cd NMR** (111 MHz, C_6D_6, 298 K) ***δ***: no resonance observed in a range of +850 – -2000 ppm. **Elemental Analysis** calc. for $C_{36}H_{79}Al_1Cd_1N_6Si_4$ (found) C 51.00 (50.72), H 9.39 (9.27), N 9.91 (9.58).

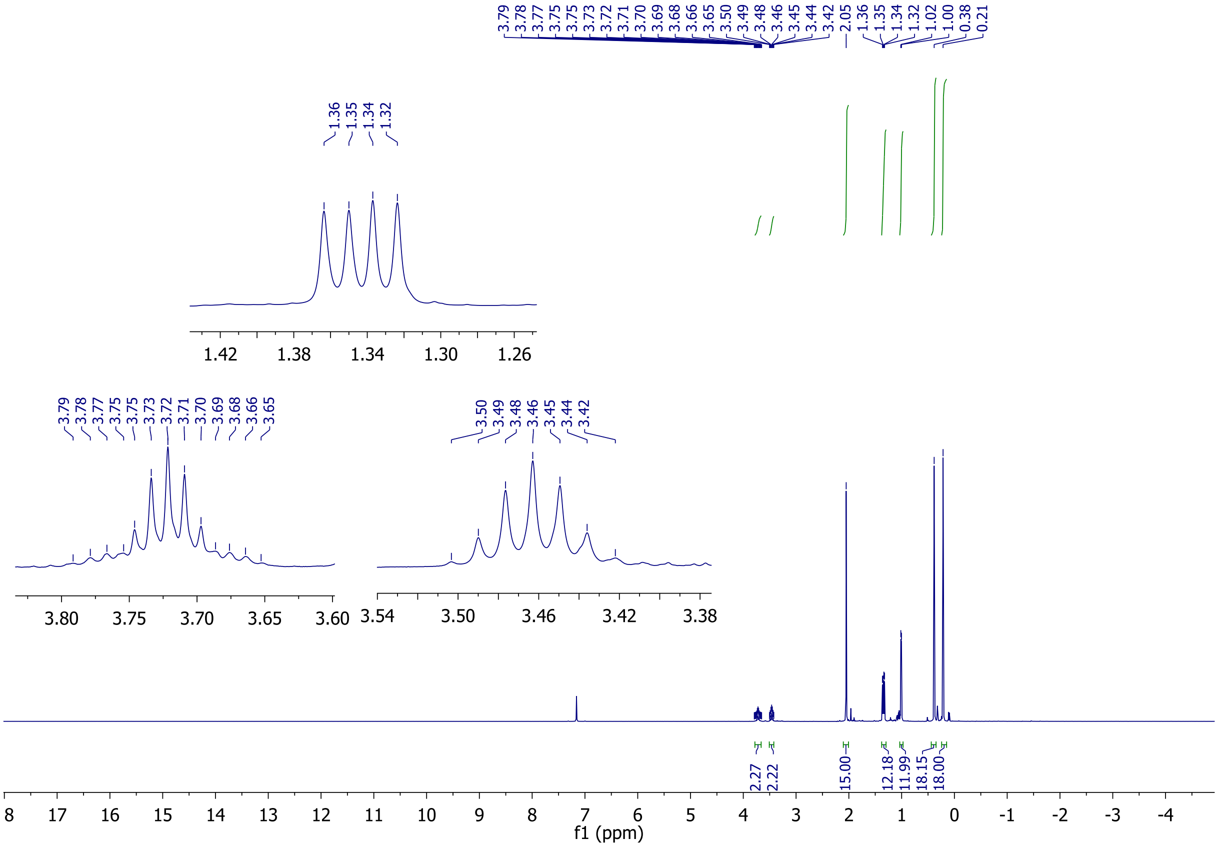

Figure S39: ^{1}H NMR (500 MHz, C_6D_6) of **7a**.

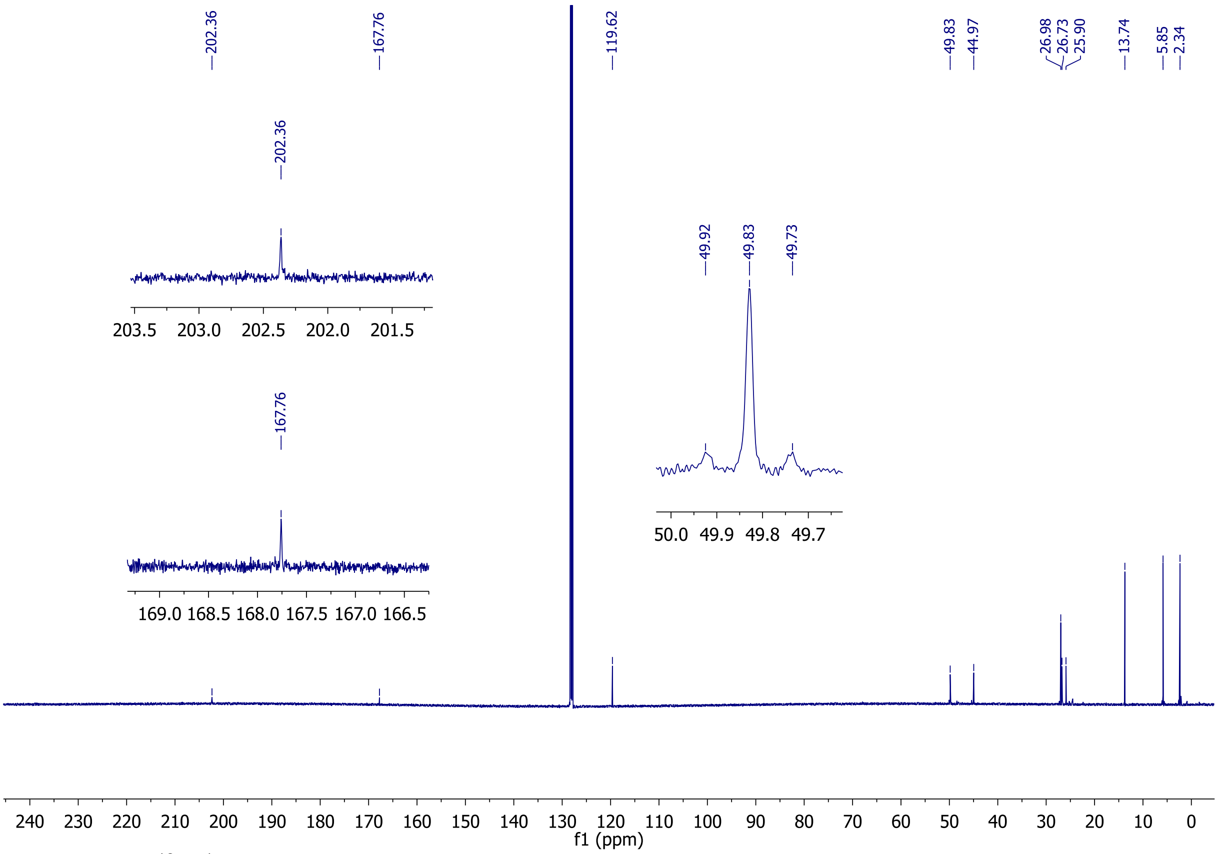

Figure S40: ^{13}C{^{1}H} NMR (126 MHz, C_6D_6) of **7a**.

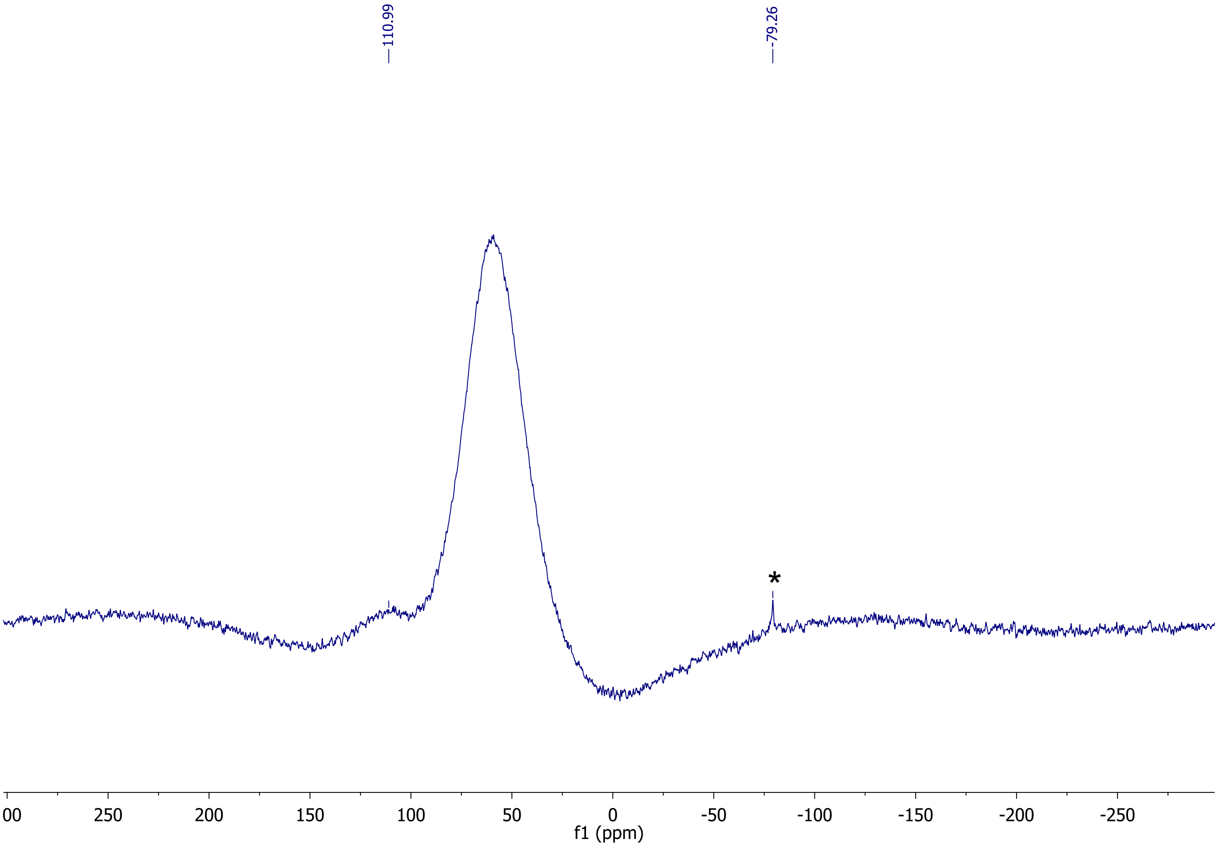

Figure S41: ^{27}Al NMR (130 MHz, C_6D_6) of **7a**. Resonance at ~50 ppm = probe head; * = trace $(AlCp^*)_4$ as internal standard.

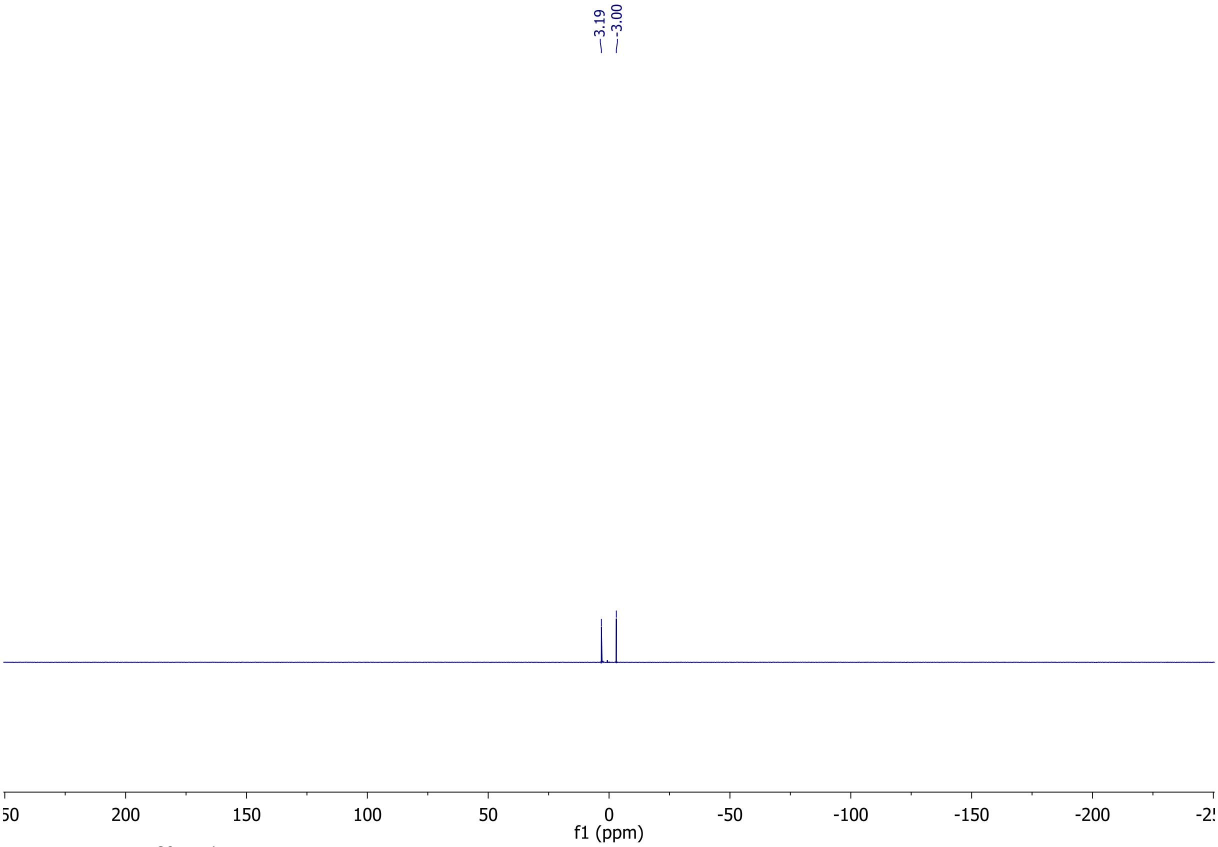

Figure S42: $^{29}Si\{^1H\}$ NMR (99 MHz, C_6D_6) of **7a**.

3.9 [{N(TMS)$_2$}(Cp*)Al{(NCy)$_2$C}Cd{(NCy)$_2$C–N(TMS)$_2$}] (7b)

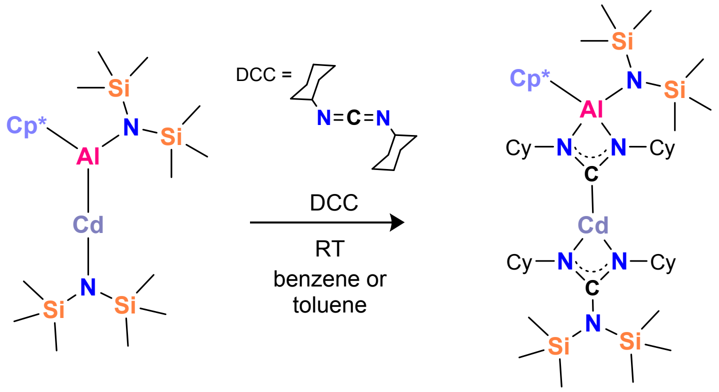

In an ampoule with PTFE valve (FengTecEx), 20.0 mg of finely ground [Cp*Al]$_4$ (0.031 mmol; 1.1 eq. of "AlCp*") and 50 mg Cd{N(TMS)$_2$}$_2$ (0.115 mmol, 1 eq.) were suspended in 4 mL of toluene. To convert the [Cp*Al]$_4$, the mixture was sonicated at 50°C for twenty minutes and afterwards treated thermally without sonication for an additional period of ten minutes. Next, DCC (45.1 mg, 0.218 mmol, 1.9 eq.) was dissolved in 1 mL of toluene and added to the grey-metallic solution. Immediately after mixing, the solvent is removed under reduced pressure. The grey residue is extracted with 4 mL of *n*-pentane and subsequently filtered through a PTFE syringe filter (d = 13 mm; pore size 0.22 µm) inside the glovebox. Slow evaporation yields a colorless oil of neat **6b** (yield: 103 mg, 89%). The oil can be transformed into crystalline **7b·TMS** when recrystallizing from tetramethylsilane at –30°C.

^{1}H NMR (500 MHz, C_6D_6, 298 K) ***δ***: 3.43–3.27 (m, 2H, –C*H* of Cy), 3.14–3.08 (m, 2H, –C*H* of Cy), 2.27–2.20 (s, 4H, –C*H*$_2$ of Cy), 2.09 (s, 15H, –C*H*$_2$ of Cy; –C*H*$_3$ of Cp*), 1.86–1.81 (m, 8H, –C*H*$_2$ of Cy), 1.70–1.59 (m, 8H, –C*H*$_2$ of Cy), 1.32–1.06 (m, 20H, –C*H*$_2$ of Cy), 0.41 (s, 18H, C*H*$_3$ of HMDS), 0.25 (s, 18H, C*H*$_3$ of HMDS) ppm. **^{13}C{^{1}H} NMR** (126 MHz, C_6D_6, 298 K) ***δ***: 202.8 (s, Cd-*C*N$_2$ of DCC), 167.8 (s, $^2J_{CdC}$ = 16 Hz (*Cd-satellites*), N-*C*N$_2$ of DCC),119.5 (s, Cq of Cp*), 58.0 (s, $^3J_{CdC}$ = 23 Hz (*Cd-satellites*), –*C*H of Cy), 53.3 (s, –*C*H of Cy), 38.1 (s, –*C*H$_2$ of Cy), 38.0 (br s, –*C*H$_2$ of Cy), 27.2 (br s, –*C*H$_2$ of Cy), 26.9 (s, –*C*H$_2$ of Cy), 26.6 (s, –*C*H$_2$ of Cy), 26.1 (s, –*C*H$_2$ of Cy), 25.9 (s, –*C*H$_2$ of Cy), 13.7 (s, –*C*H$_2$ of Cy), 5.8 (s, –*C*H$_3$ of HMDS), 2.4 (s, –*C*H$_3$ of HMDS) ppm.**^{27}Al NMR** (130 MHz, C_6D_6, 298 K) ***δ***: no resonance observed in a range of +300 – -300 ppm.**^{29}Si{^{1}H} NMR** (100 MHz, C_6D_6, 298 K) ***δ***: 3.3 (s, *Si* of HMDS), −3.0 (s, *Si* of HMDS) ppm. **^{113}Cd NMR** (111 MHz, C_6D_6, 298 K) ***δ***: no resonance observed in a range of +850 – -2000 ppm. **Elemental Analysis** calc. for $C_{48}H_{95}Al_1Cd_1N_6Si_4$ (found) C 57.19 (56.49), H 9.39 (9.93), N 8.34 (7.83).

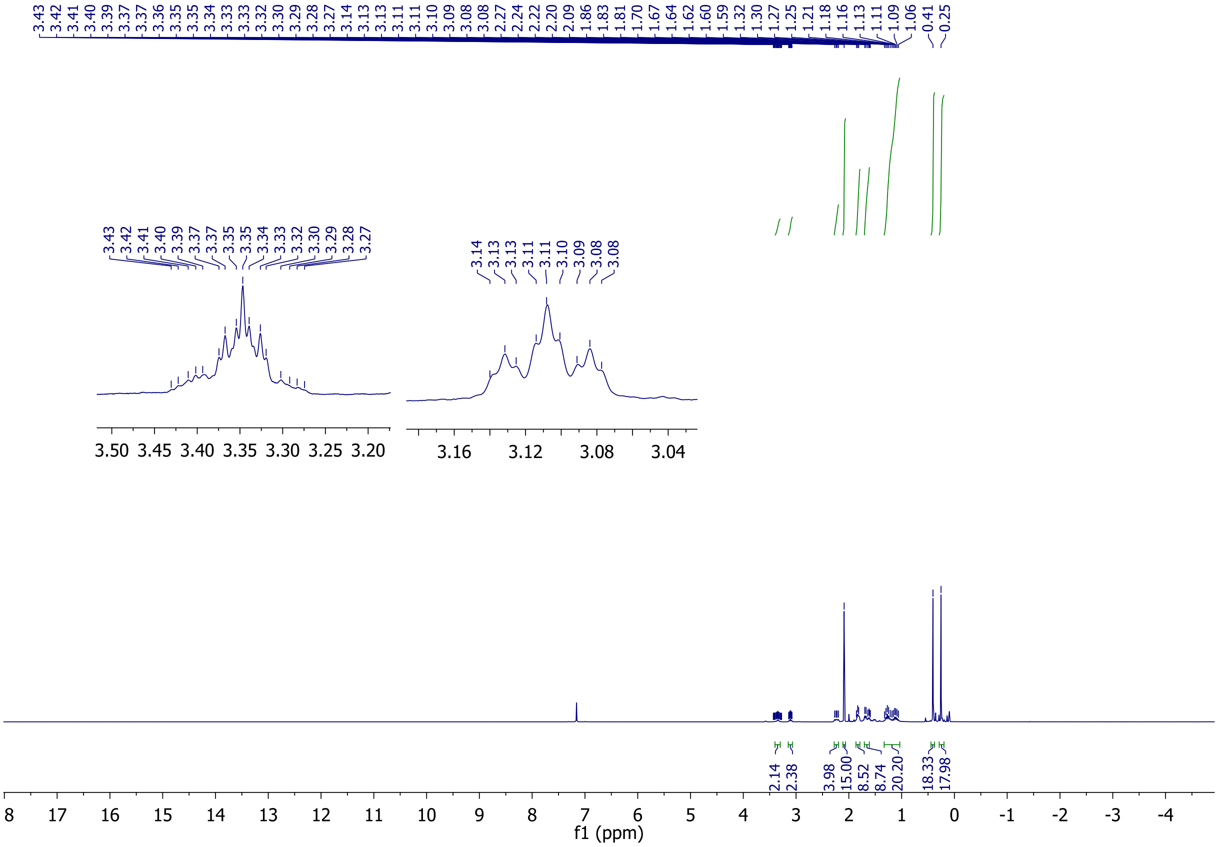

Figure S43: ^{1}H NMR (500 MHz, C_6D_6) of **7b**.

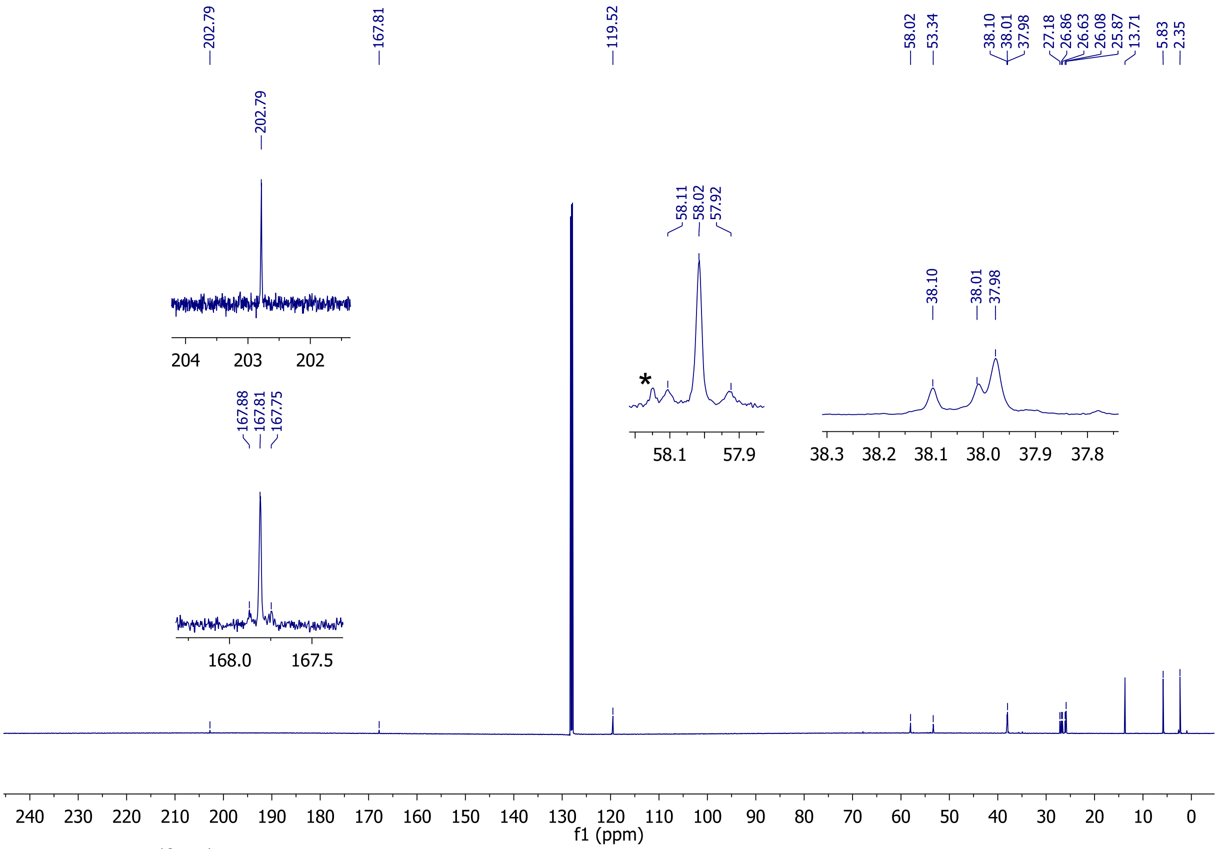

Figure S44: ^{13}C{^{1}H} NMR (126 MHz, C_6D_6) of **7b**. * = trace impurity or decomposition product.

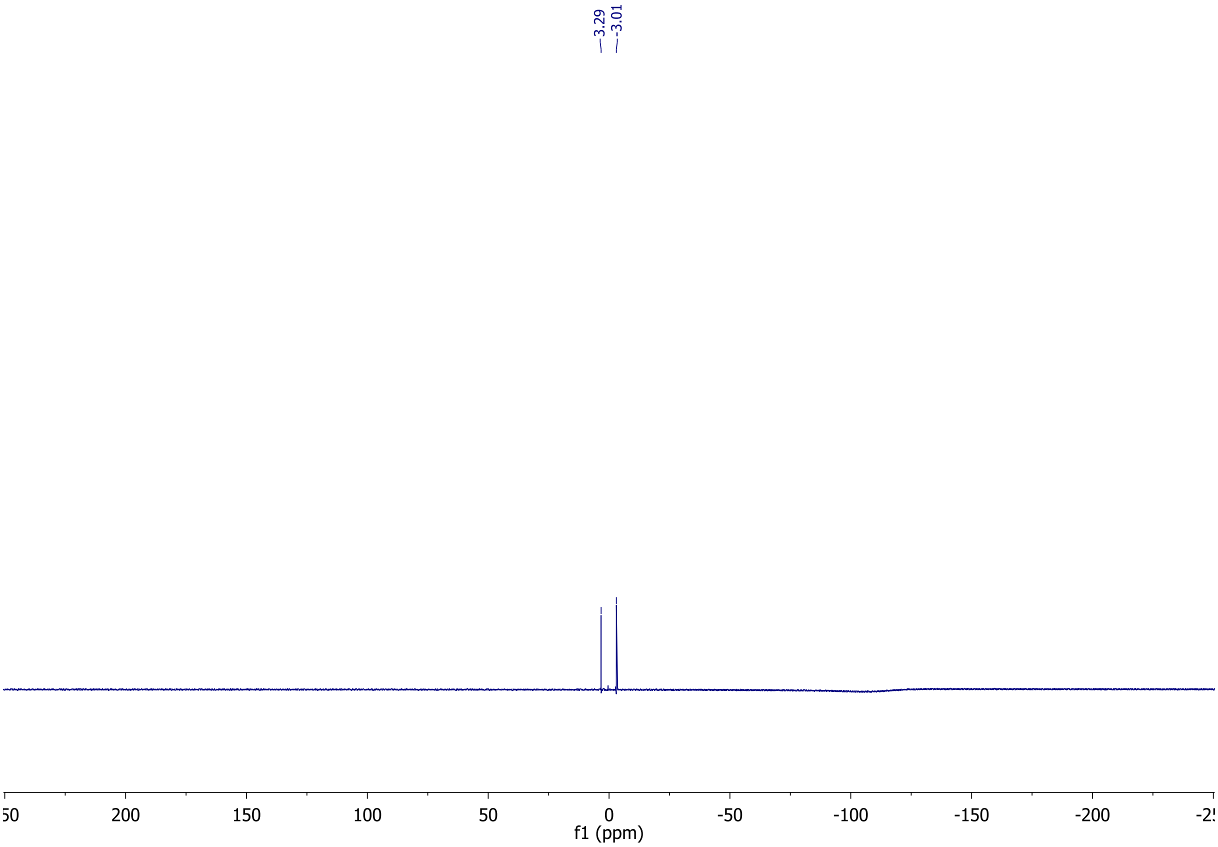

Figure S45: $^{29}Si\{^1H\}$ NMR (99 MHz, C_6D_6) of **7b**.

3.10 [{N(TMS)$_2$}(Cp*)Al{(N^{i}Pr)$_2$C}]$_2$Cd (**8a**)

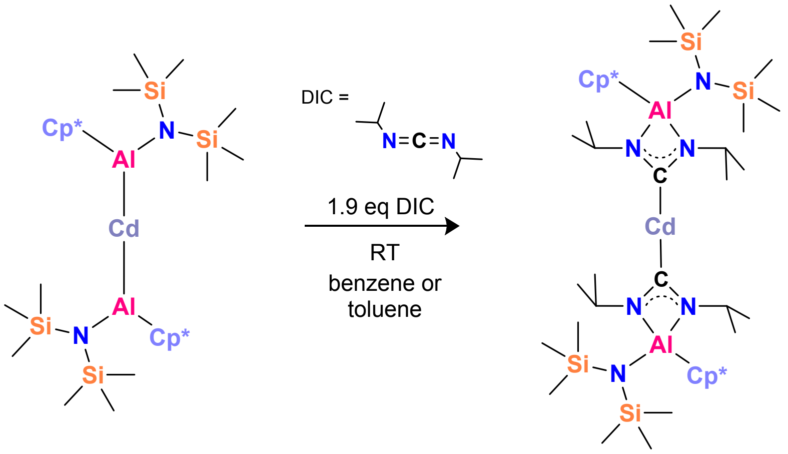

In an ampoule with PTFE valve (FengTecEx), 40.0 mg of finely ground [Cp*Al]$_4$ (0.062 mmol; 2.2 eq. of “AlCp*”) and 50 mg Cd{N(TMS)$_2$}$_2$ (0.115 mmol, 1 eq.) were suspended in 4 mL of toluene. To convert the [Cp*Al]$_4$, the mixture was sonicated at 50°C for twenty minutes and afterwards treated thermally without sonication for an additional period of ten minutes. Next, DIC (33 µL; 26.9 mg, 0.212 mmol, 1.85 eq.) was dissolved in 1 mL of toluene and added to the grey-metallic solution. Immediately after mixing, the solvent is removed under reduced pressure. The grey residue is extracted with 4 mL of *n*-pentane and subsequently filtered through a PTFE syringe filter (d = 13 mm; pore size 0.22 µm) inside the glovebox. Slow evaporation yields a colorless oil of neat **7a**. This oil can be transformed into a fibrous, colorless solid upon drying *in vacuo* and subsequently storing at –30°C in the glovebox (yield: 95 mg, 81%). Also possible is lyophilization from neat TMS which yields **8a** in the form of a colorless wax.

^{1}H NMR (500 MHz, C$_6$D$_6$, 298 K) δ: 3.25 (hept, $^3J_{HH}$ = 6.6 Hz, 4H, −*C*H of ^{i}Pr), 1.99 (s, 30H, −*C*H$_3$ of Cp*), 1.23 (ps t, $^3J_{HH}$ = 5.9 Hz, 24H, −*C*H$_3$ of ^{i}Pr),* 0.35 (s, 36H, −CH$_3$ of HMDS) ppm. * = two overlapping doublets as a pseudo triplet. **^{13}C{^{1}H} NMR** (126 MHz, C$_6$D$_6$, 298 K) δ: 202.8 (s, $^1J_{CdC}$ = 865 Hz (*Cd-satellites*), *C*N$_2$ of DIC), 119.9 (s, C_q of Cp*), 52.14 (s, $^3J_{CdC}$ = 23 Hz (*Cd-satellites*), −*C*H of ^{i}Pr), 27.4 (s, −*C*H$_3$ of ^{i}Pr), 25.9 (s, −*C*H$_3$ of ^{i}Pr), 13.9 (s, −*C*H$_3$ of Cp*), 5.94 (−*C*H$_3$ of HMDS) ppm. **^{27}Al NMR** (130.21 MHz, C$_6$D$_6$, 298 K) δ: 116.5 (s (br), $\omega_{1/2}$ = 3940 Hz) ppm. **^{29}Si{^{1}H} NMR** (100 MHz, C$_6$D$_6$, 298 K) δ: −2.8 (s, *Si* of HMDS) ppm. **^{113}Cd NMR** (111 MHz, C$_6$D$_6$, 298 K) δ: no resonance observed in a range of +850 – -2000 ppm. **Elemental Analysis** calc. for C$_{58}$H$_{94}$Al$_2$Cd$_1$N$_6$Si$_4$ (found) C 54.70 (54.99), H 9.39 (9.67), N 8.34 (8.24).

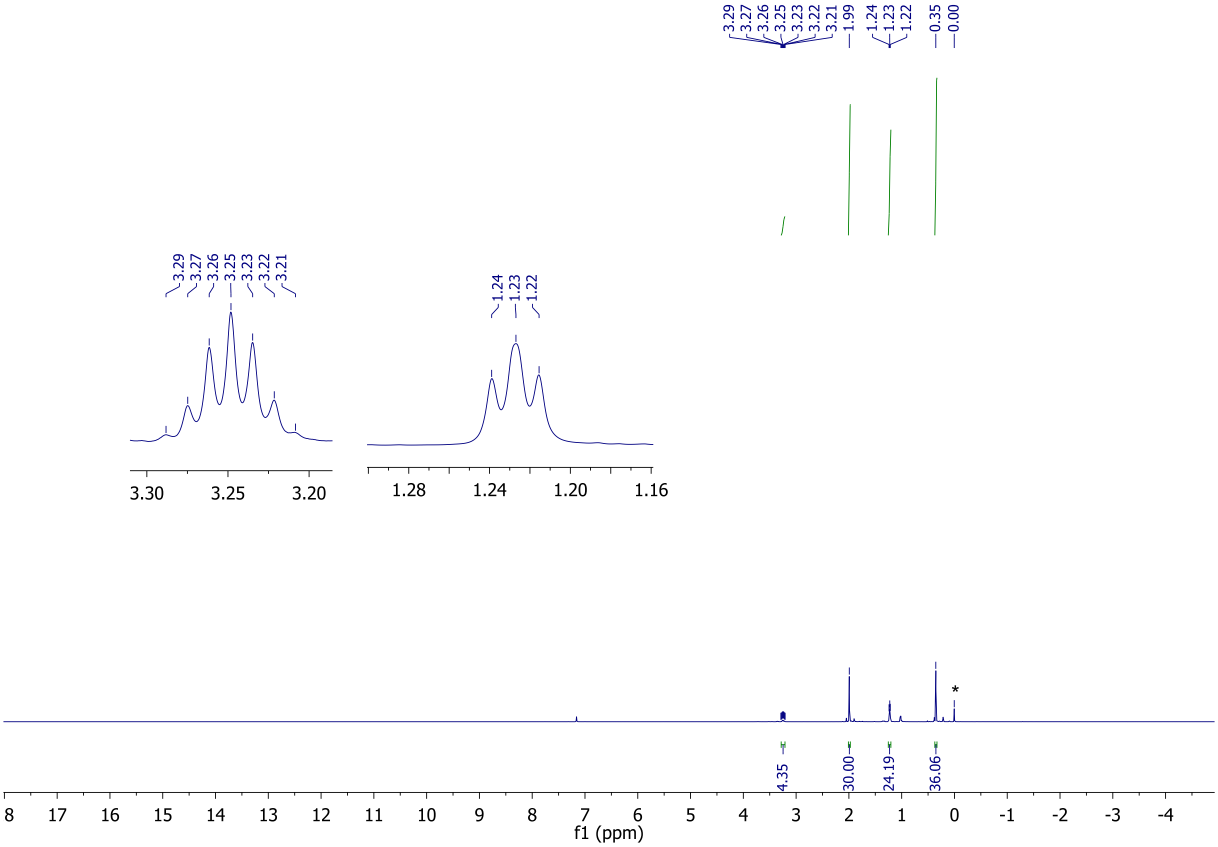

Figure S46: ^{1}H NMR (500 MHz, C_6D_6) of **8a**. * = Residual TMS.

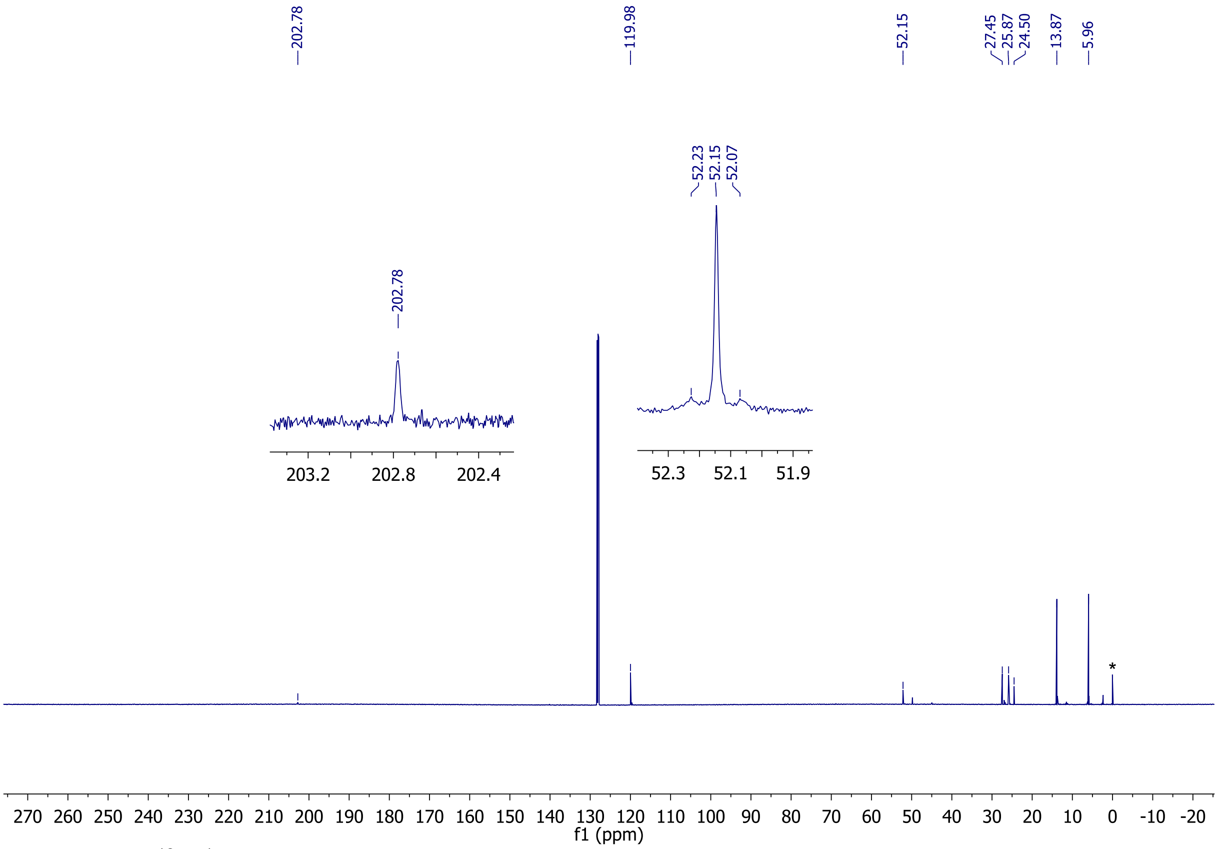

Figure S47: $^{13}C\{^{1}H\}$ NMR (126 MHz, C_6D_6) of **8a**. * = Residual TMS.

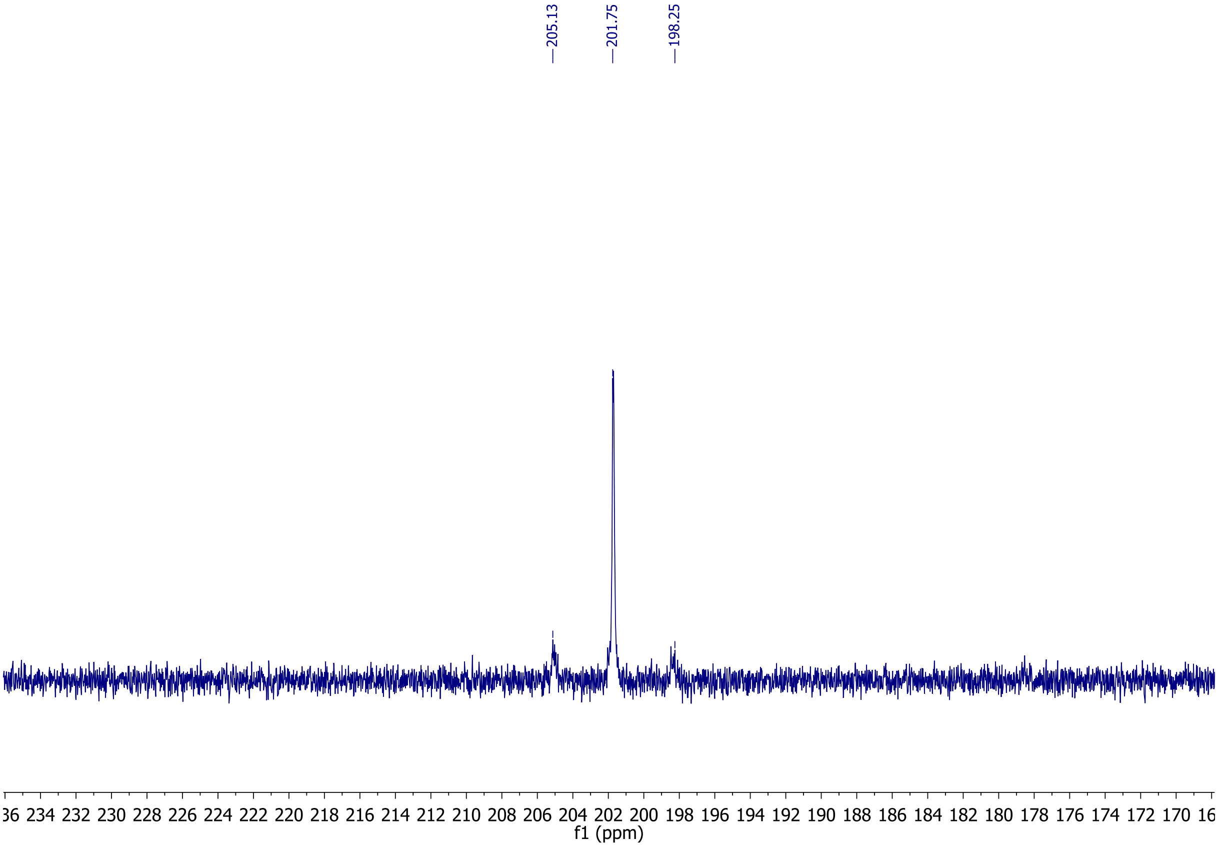

Figure S48: $^{13}C\{^{1}H\}$ NMR (126 MHz, C_6D_6) of 8**a** (high-resolution spectrum resolving Cd-C coupling.).

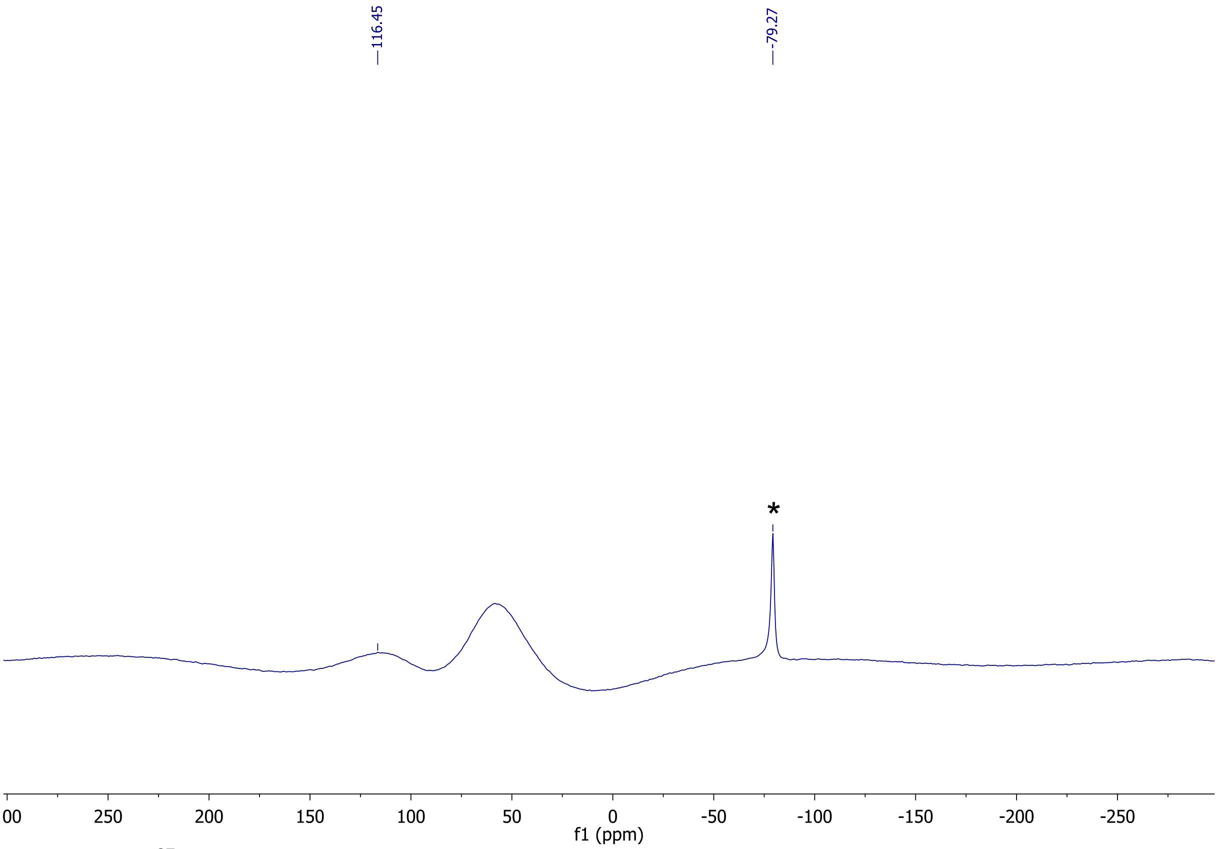

Figure S49: ^{27}Al NMR (130 MHz, C_6D_6) of 8**a**. Resonance at ~50 ppm = probe head; * = trace $(AlCp^*)_4$ as internal standard.

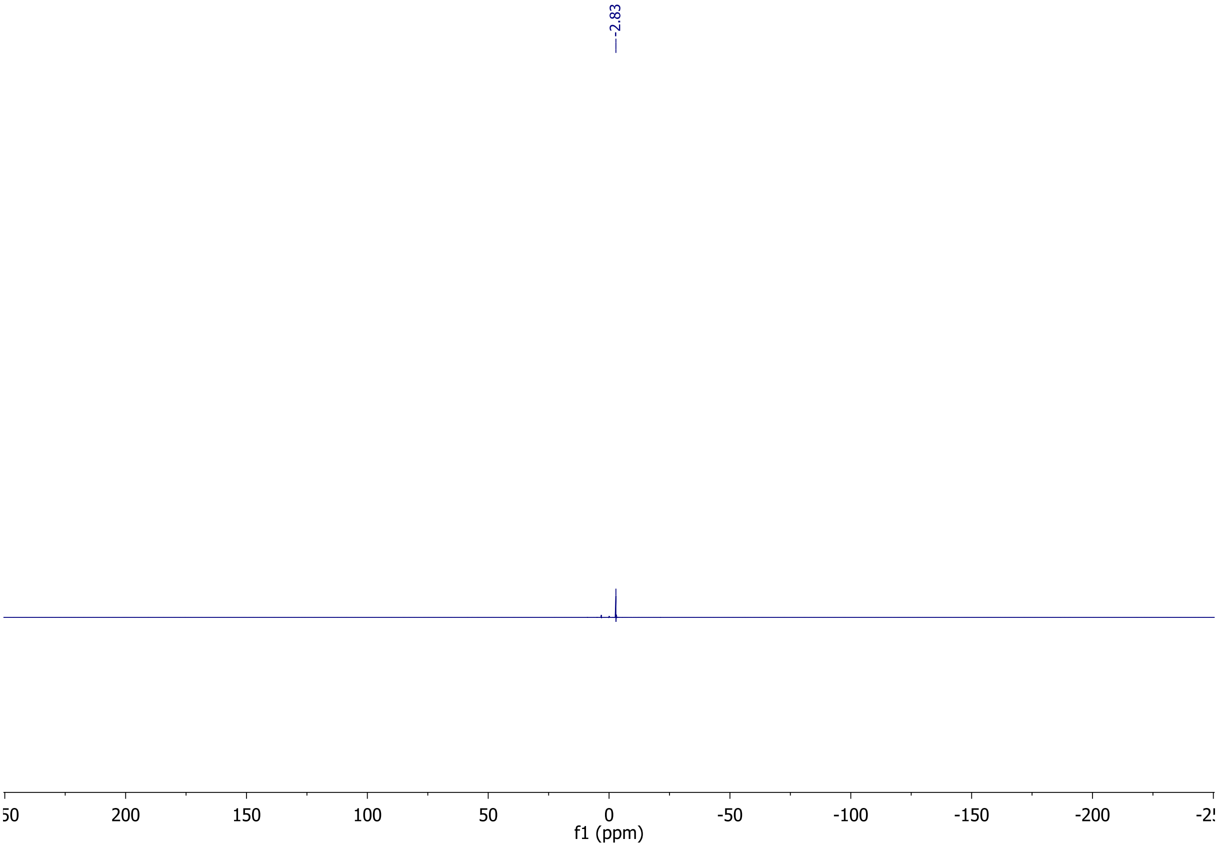

Figure S50: $^{29}Si\{^1H\}$ NMR (99 MHz, C_6D_6) of **8a**.

3.11 [{N(TMS)$_2$}(Cp*)Al{(NCy)$_2$C}]$_2$Cd (8b)

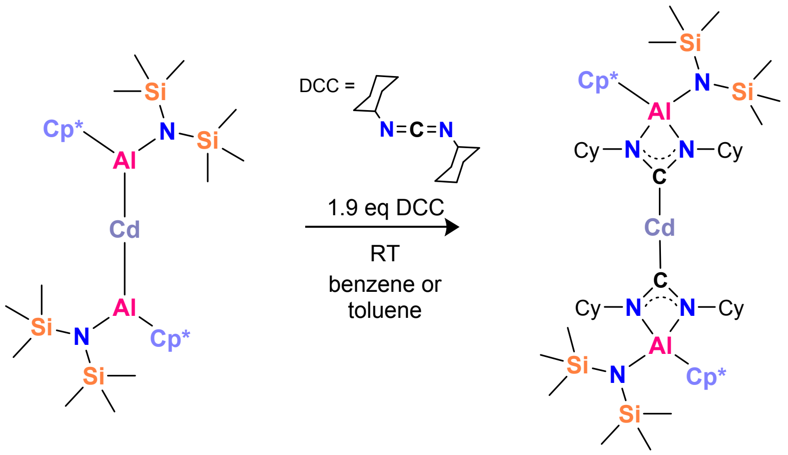

In an ampoule with PTFE valve (FengTecEx), 40.0 mg of finely ground [Cp*Al]$_4$ (0.062 mmol; 2.2 eq. of “AlCp*”) and 50 mg Cd{N(TMS)$_2$}$_2$ (0.115 mmol, 1 eq.) were suspended in 4 mL of toluene. To convert the [Cp*Al]$_4$, the mixture was sonicated at 50°C for twenty minutes and afterwards treated thermally without sonication for an additional period of ten minutes. Next, DCC (45.0 mg, 0.22 mmol, 1.9 eq.) was dissolved in 1 mL of toluene and added to the grey-metallic solution. Immediately after mixing, the solvent is removed under reduced pressure. The grey residue is extracted with 4 mL of *n*-pentane and subsequently filtered through a PTFE syringe filter (d = 13 mm; pore size 0.22 µm) inside the glovebox. Slow evaporation yields a colorless oil of neat **8b**. This oil can be transformed into a fibrous, colorless solid upon drying *in vacuo* and subsequently storing at –30°C in the glovebox (yield: 95 mg, 84%). Also possible is lyophilization from benzene which yields **8b** in the form of a colorless powder.

^{1}H NMR (500 MHz, C_6D_6, 298 K) ***δ***: 2.97–2.91 (m, 4H, –C*H* of Cy), 2.19–2.16 (m, 2H, –C*H*$_2$ of Cy), 2.09–2.02 (m+s, 34H, –C*H*$_2$ of Cy; –C*H*$_3$ of Cp*), 1.76–1.69 (m, 8H, –C*H*$_2$ of Cy), 1.56–1.35 (m, 8H, –C*H*$_2$ of Cy), 1.24–1.04 (m, 12H, –C*H*$_2$ of Cy), 0.36 (s, 36H, C*H*$_3$ of HMDS) ppm. **^{13}C{^{1}H} NMR** (126 MHz, C_6D_6, 298 K) ***δ***: 199.6 (s, (s, $^{1}J_{111\mathrm{CdC}}$ = 817 Hz; $^{1}J_{113\mathrm{CdC}}$ = 856 Hz, *C*N$_2$ of DCC), 119.5 (s, Cq of Cp*), 59.4 (s, –*C*H of Cy), 38.8 (s, –*C*H of Cy), 38.0 (s, –*C*H$_2$ of Cy), 26.7 (br s, –*C*H$_2$ of Cy), 26.3 (br s, –*C*H$_2$ of Cy), 25.9 (s, –*C*H$_2$ of Cy), 13.4 (s, –*C*H$_3$ of Cp*), 5.6 (s, –*C*H$_3$ of HMDS) ppm. **^{27}Al NMR** (130 MHz, C_6D_6, 298 K) ***δ***: no resonance observed in a range of +300 – -300 ppm. **^{29}Si{^{1}H} NMR** (100 MHz, C_6D_6, 298 K) ***δ***: −2.7 (s, *Si* of HMDS) ppm. **^{113}Cd NMR** (111 MHz, C_6D_6, 298 K) ***δ***: no resonance observed in a range of +850 – -2000 ppm. **Elemental Analysis** calc. for $C_{58}H_{110}Al_2Cd_1N_6Si_4$ (found) C 59.53 (58.57), H 9.47 (9.65), N 7.18 (6.81).

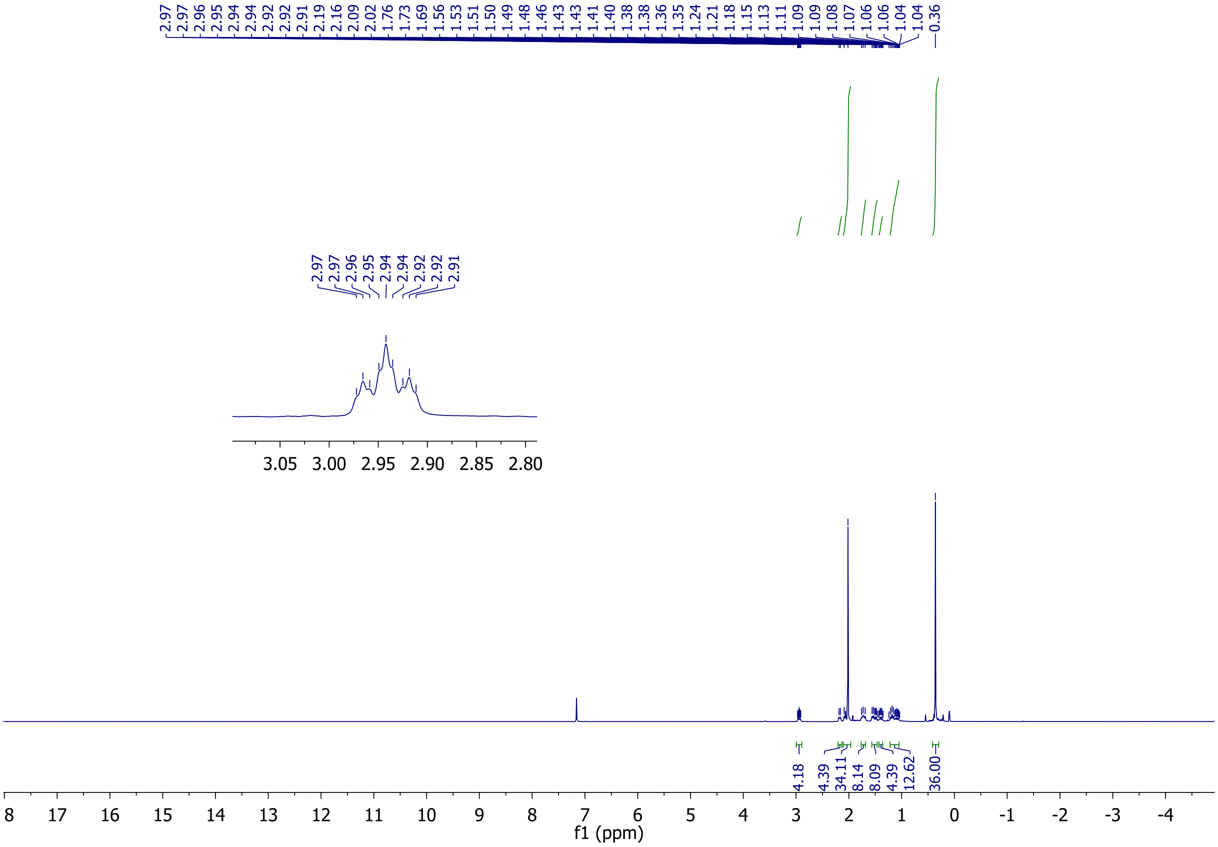

Figure S51: ^{1}H NMR (500 MHz, C_6D_6) of **8b**.

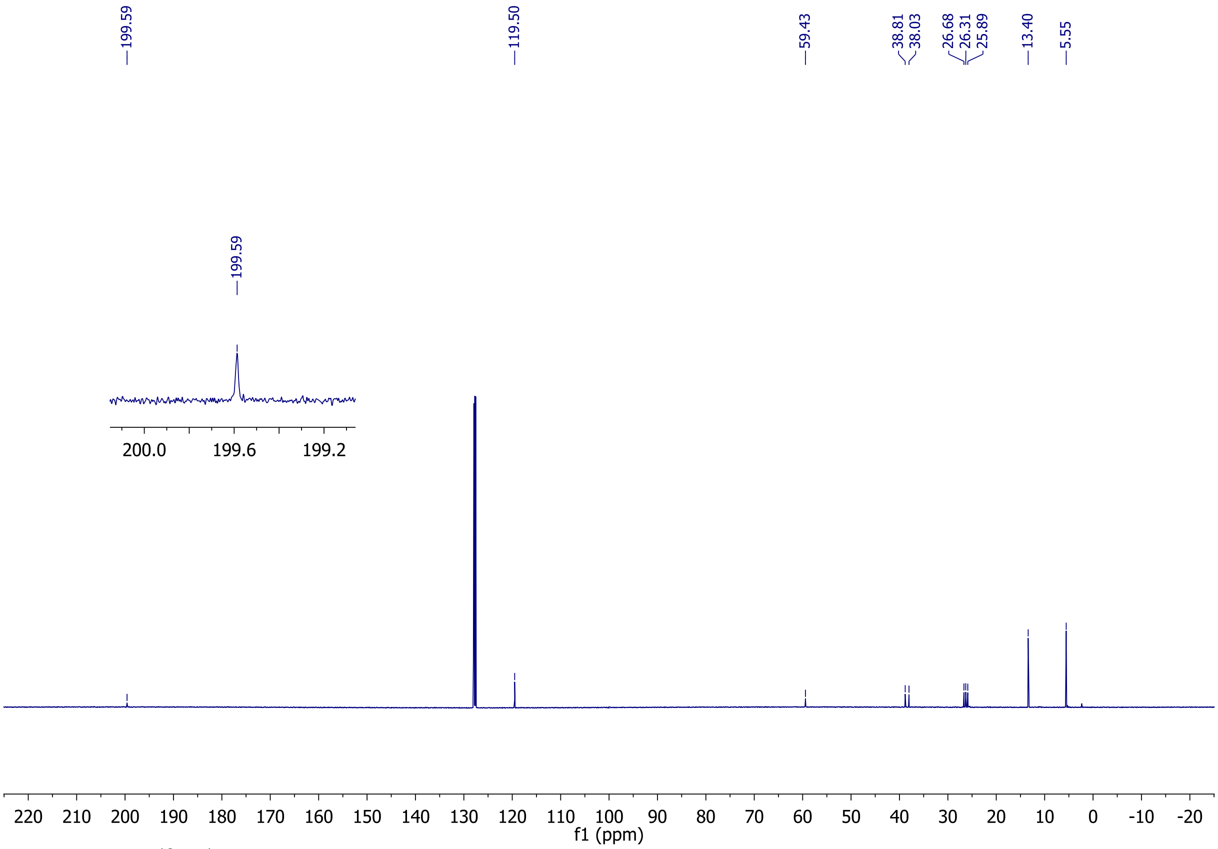

Figure S52: ^{13}C{^{1}H} NMR (126 MHz, C_6D_6) of **8b**.

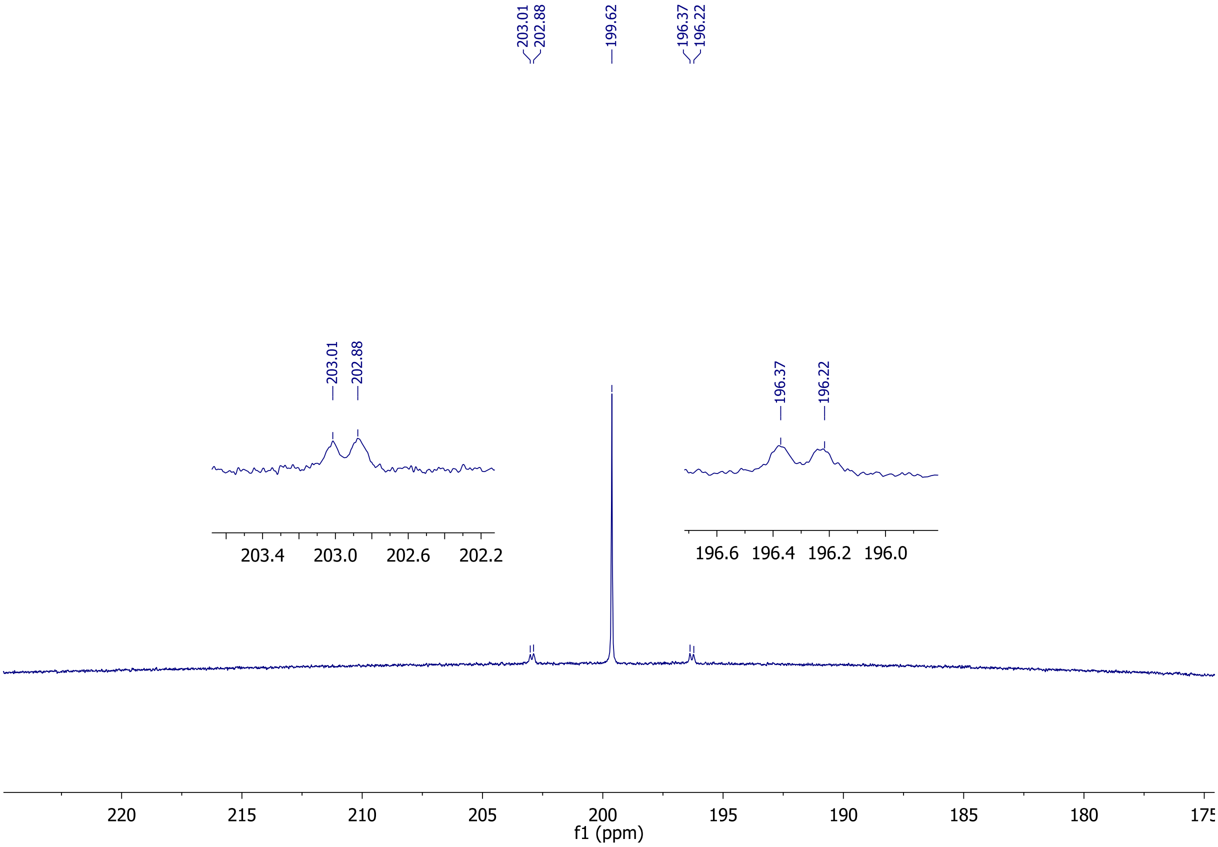

Figure S53: $^{13}C\{^{1}H\}$ NMR (126 MHz, C_6D_6) of **8b** (high-resolution spectrum resolving Cd-C coupling.).

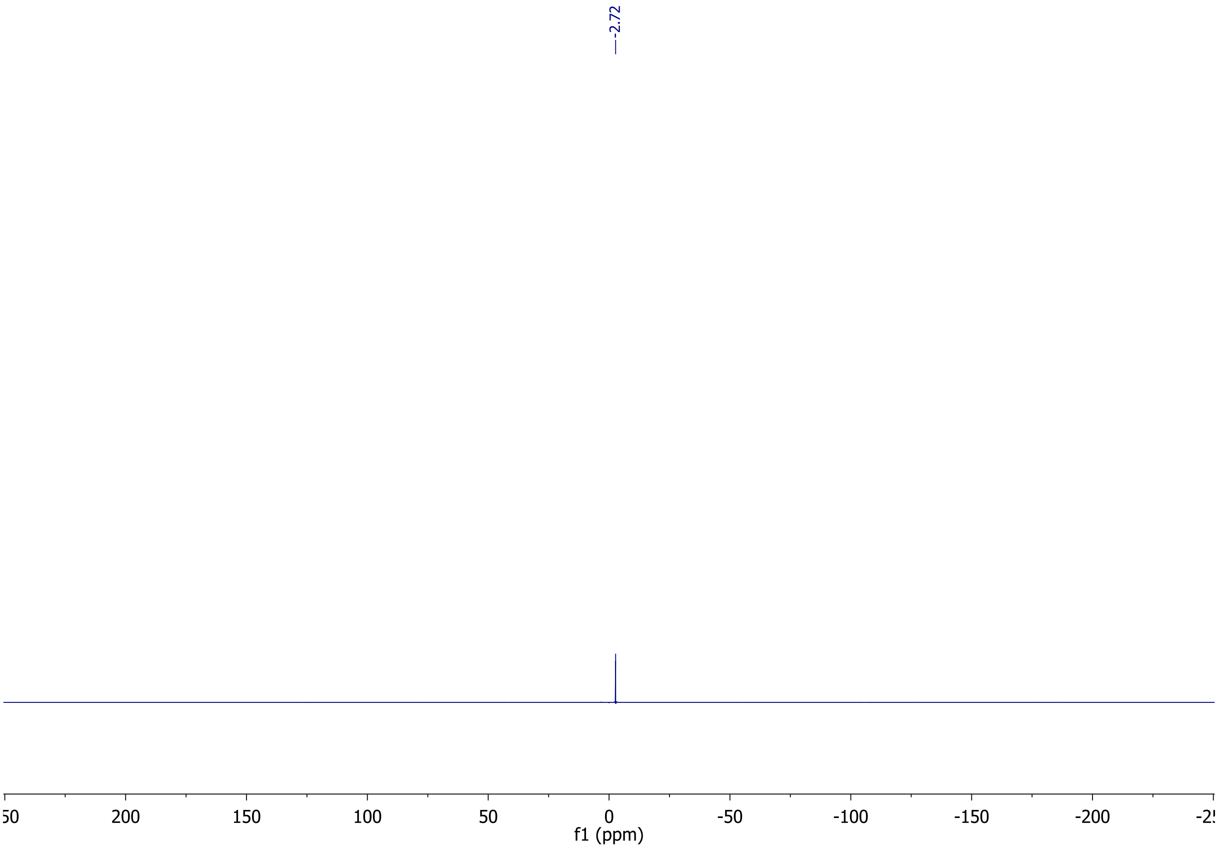

Figure S54: $^{29}Si\{^{1}H\}$ NMR (99 MHz, C_6D_6) of **8b**.

3.12 [{N(TMS)$_2$}(Cp*)Al{O$_2$C–N(TMS)$_2$}] (**9**)

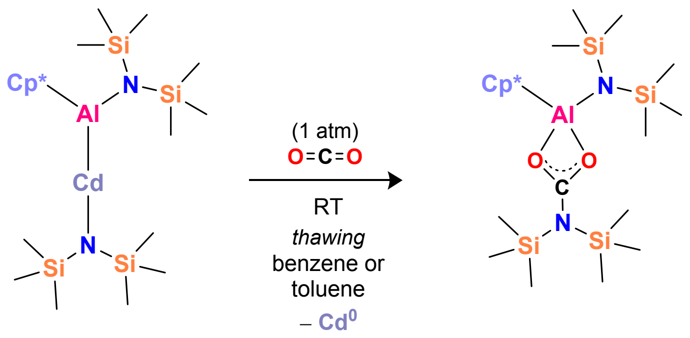

7.5 mg of finely ground [Cp*Al]$_4$ (0.046 mmol, 1.15 eq.) and 17.3 mg Cd(HMDS)$_2$ (0.04 mmol, 1 eq.) were suspended in 1 mL of C_6D_6. The mixture was then transferred to a *J. Young* NMR tube. To convert the [Cp*Al]$_4$, the mixture was sonicated at 50°C for twenty minutes and afterwards heated without sonication for an additional period of ten minutes. Once the conversion to **1** is completed, the *J. Young* tube was degassed using one freeze-pump-thaw cycle. The benzene (or toluene, respectively) solution is frozen using a dry-ice bath (or liquid nitrogen). Afterwards, the atmosphere of CO_2 is applied to the tube. Upon carefully thawing the solution, immediately black precipitates form. The conversion of **1** is quantitatively giving **9** in near quantitative yield. Running the above-mentioned protocol twice and combining the solutions in an ampule with PTFE valve (FengTecEx) gives a dark-black slurry containing colorless crystals that form when removing the solvent. Extraction with 4 mL of *n*-pentane and filtering through a syringe filter (d = 13 mm; pore size 0.22 µm) inside the glovebox yields a clear colorless solution. The solvent is allowed to evaporate which yields extremely fragile crystalline material of **9** (27.6 mg; 65 %). ***Note:*** *Attempts to analyze these crystals via elemental analysis failed due to readily formation of sticky greases upon contact with a spatula.*

^{1}H NMR (500 MHz, C_6D_6, 298 K) δ: 2.02 (s, 15H, CH_3 of Cp*), 0.31 (s, 18H, CH_3 of HMDS), 0.25 (s, 18H, CH_3 of HMDS) ppm. **^{13}C{^{1}H} NMR** (126 MHz, C_6D_6, 298 K) δ: 171.5 (s, $C_{carbamate}$), 113.5 (s, C_q of Cp*), 11.5 (s, −CH_3 of Cp*), 5.2 (s, −CH_3 of HMDS), 2.6 (s, −CH_3 of HMDS) ppm. **^{27}Al NMR** (130 MHz, C_6D_6, 298 K) δ: −1.80 (s (br), $\omega_{1/2}$ = 1880 Hz) ppm. **^{29}Si{^{1}H} NMR** (100 MHz, C_6D_6, 298 K) δ: 11.6 (s, *Si* of HMDS), −0.9 (s, *Si* of HMDS) ppm.

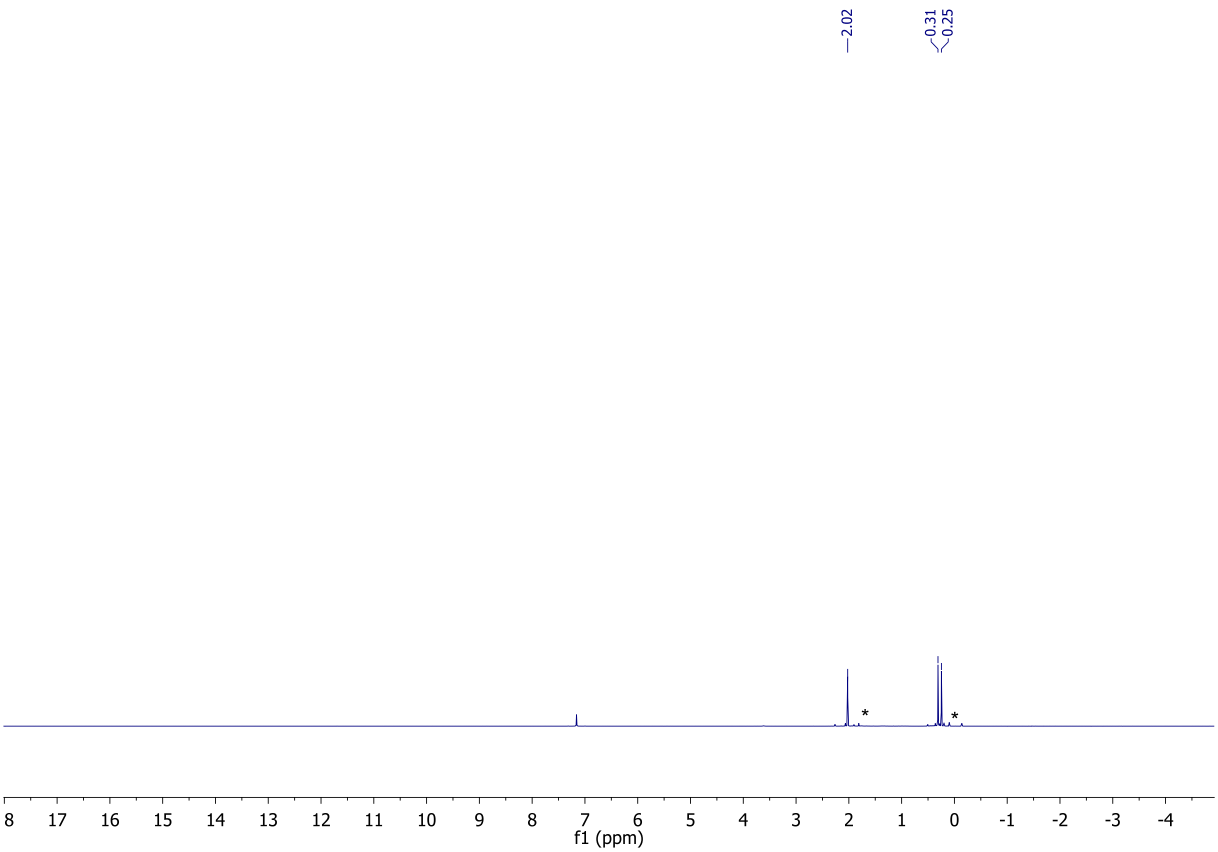

Figure S55: ^{1}H NMR (500 MHz, C_6D_6) of **9**. * = trace amounts of decomposition products, despite immediately conducted NMR spectroscopy after reaction with CO_2 and degassing the solution and headspace of the NMR tube.

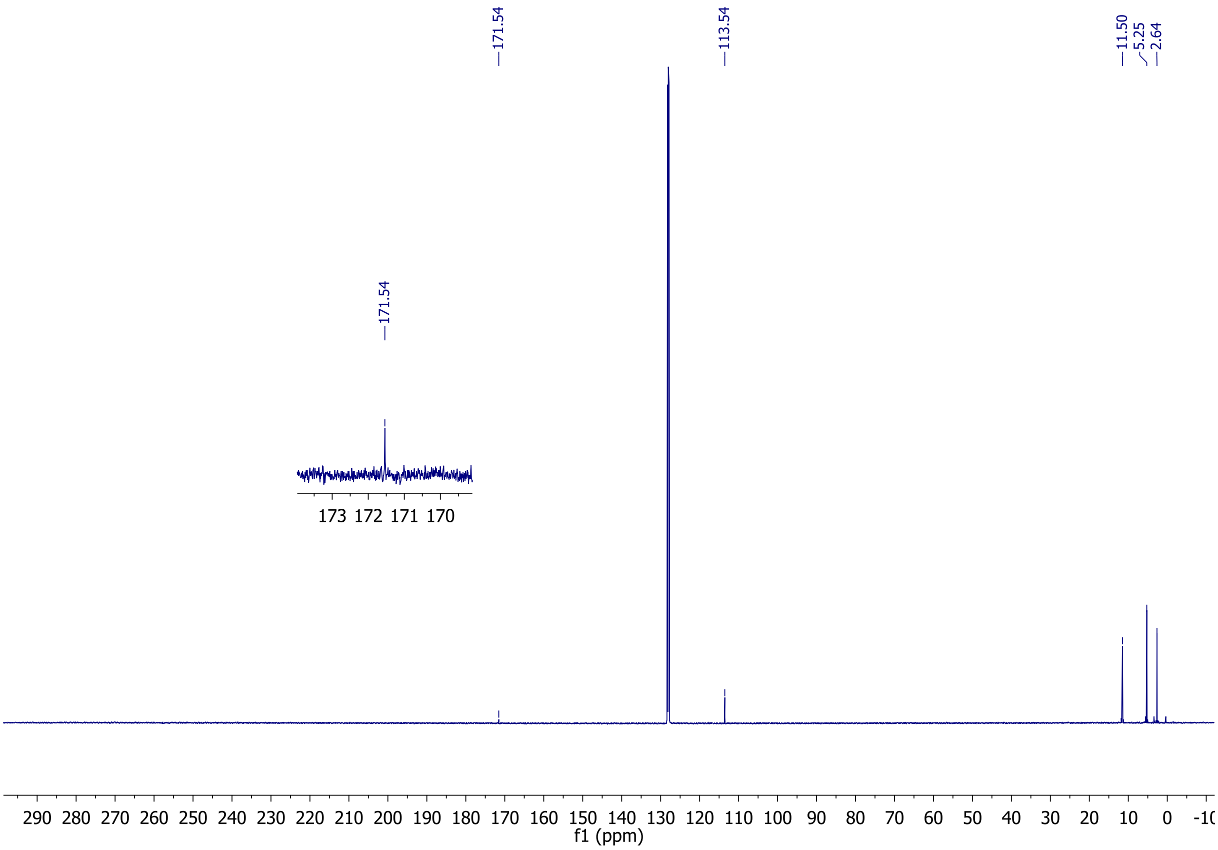

Figure S56: $^{13}C\{^1H\}$ NMR (126 MHz, C_6D_6) of **9**.

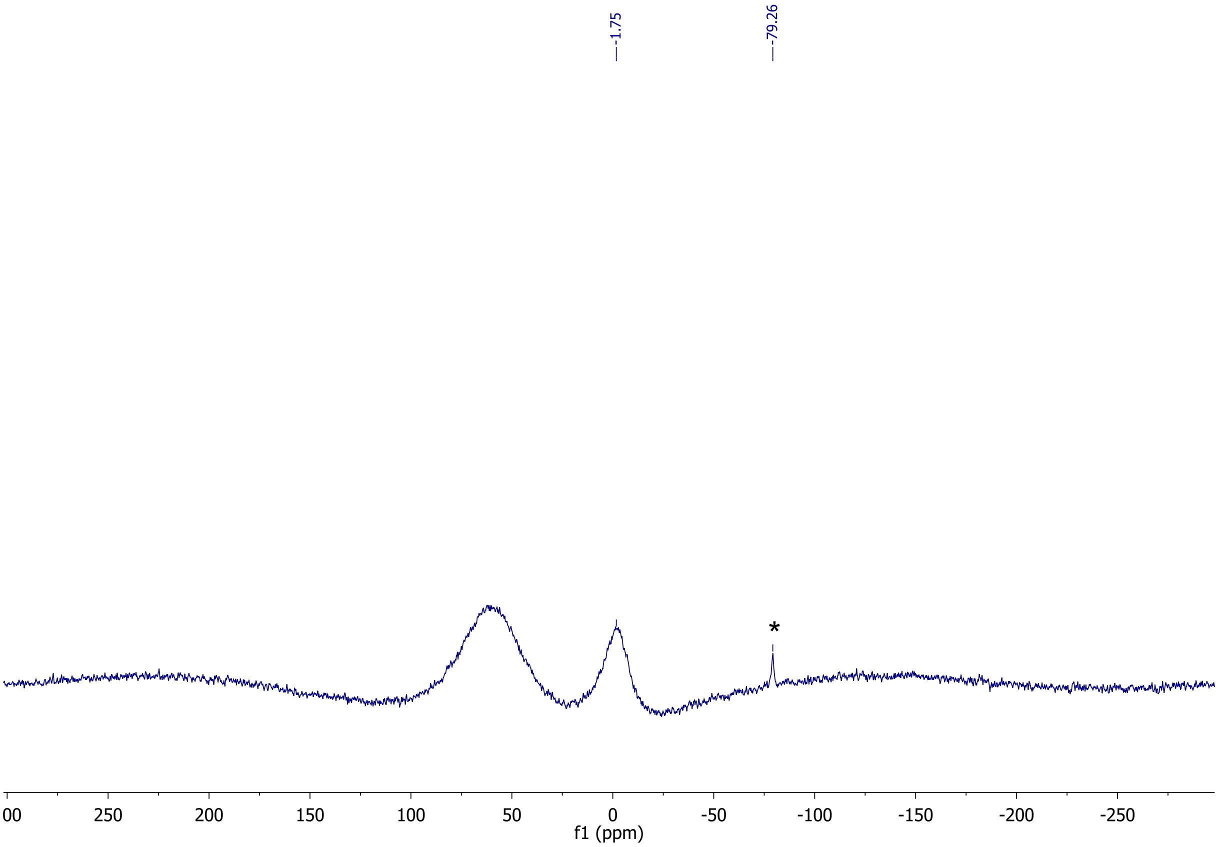

Figure S57: ^{27}Al NMR (130 MHz, C_6D_6) of **9**. Resonance at ~50 ppm = probe head; * = trace $(AlCp^*)_4$ as internal standard.

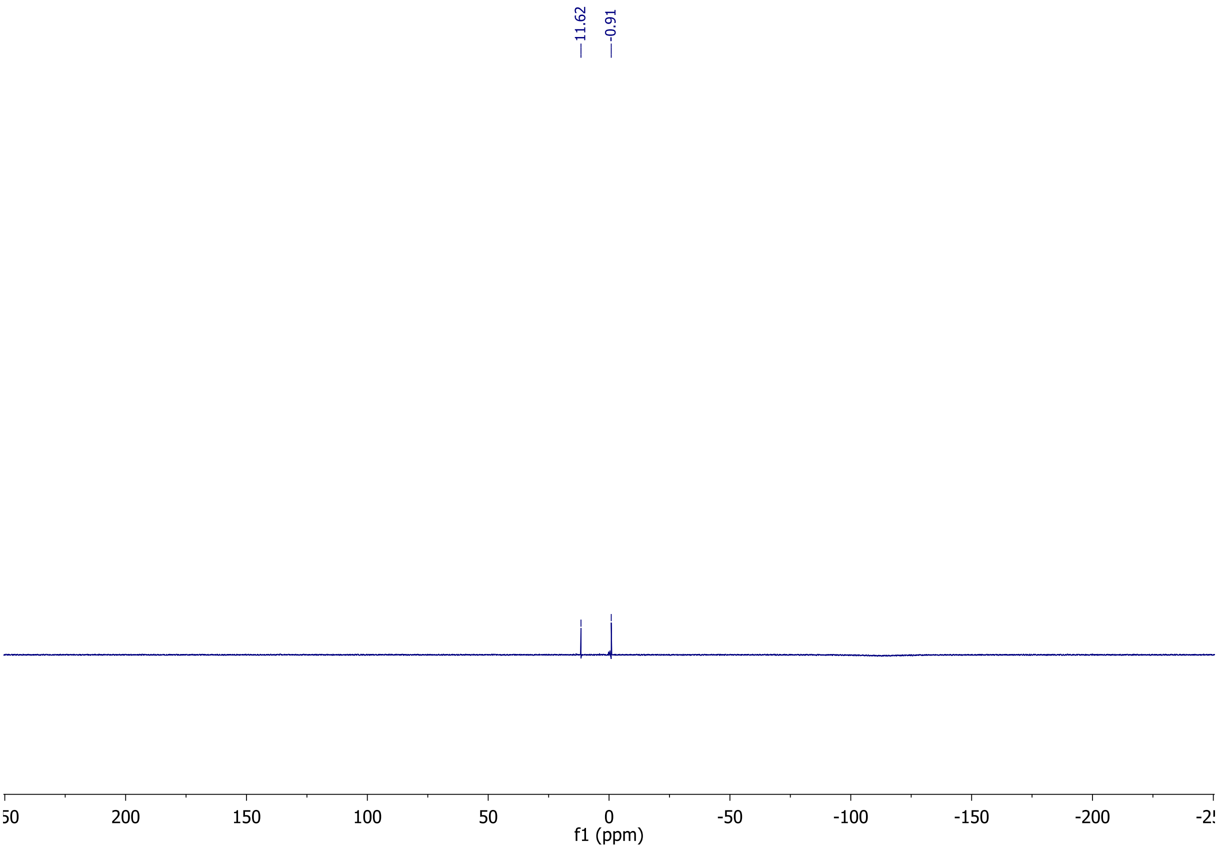

Figure S58: ^{29}Si{^{1}H} NMR (99 MHz, C_6D_6) of **9**.

4 NMR studies of Al(I)-transfer reactions

4.1 Reaction of **2** with $B(C_6F_5)_3$

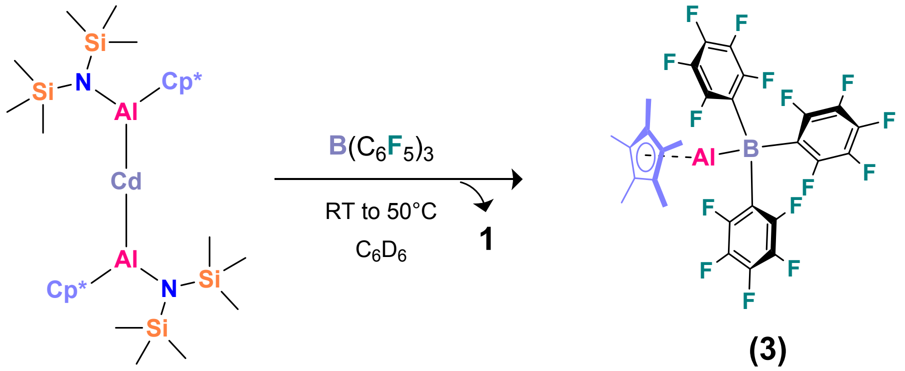

2 was synthesized according to section 4.2 with TMS as internal standard (8 µL, 0.059 mmol). After detecting quantitative conversion of $(AlCp^*)_4$ and $Cd\{N(TMS)_2\}_2$, 20.5 mg of $B(C_6F_5)_3$ (0.04 mmol, 1 eq.), dissolved in 0.4 mL of C_6D_6, was immediately added. The reaction was monitored directly after the addition of $B(C_6F_5)_3$ and after short periods at 50°C. After 30 minutes, >95% conversion of **2** to **1** and $[(Cp^*)Al–B(C_6F_5)_3]$ (**3**) is observed. The mixture of **1** and **3** was subsequently NMR spectroscopically characterized – all resonances agree with the literature and the data shown in section 4.1.[6]

^{1}H NMR (500 MHz, C_6D_6, 298 K) ***δ***: 1.92 (s, 15H, –CH_3 of Cp* of **3**), 1.30 (s, 15H, –CH_3 of Cp*), 0.31 (s, 18H, CH_3 of HMDS), 0.21 (s, 18H, –CH_3 of HMDS) ppm. **^{13}C{^{1}H} NMR** (126 MHz, C_6D_6, 298 K) ***δ***: 148.3, 146.4, 140.6, 138.6, 136.6 (m, *C*–F), 118.4 (s, C_q of Cp* of **1**), 116.2 (s, C_q of Cp* of **3**), 11.6 (s, −CH_3 of Cp*), 8.5 (s, −CH_3 of Cp* of **3**), 6.2 (s, −CH_3 of HMDS), 5.4 (s, −CH_3 of HMDS) ppm. **^{19}F NMR** (470 MHz, C_6D_6, 298 K) ***δ***: 131.0 (br s, 6F, C*F* of **3**), 158.5 (t, $^3J_{FF}$ = 21.0 Hz, 3F, C*F* of **3**), 163.6 (m, 6F, C*F* of **3**) ppm. **^{11}B{^{1}H} NMR** (160 MHz, C_6D_6, 298K) ***δ***: –29.1 (s, *B*$(C_6F_5)_3$ of **3**) ppm. **^{27}Al NMR** (130 MHz, C_6D_6, 298 K) ***δ***: –7.3 (s (br), $\omega_{1/2}$ = 1380 Hz, Cp**Al* of **3**) ppm. **^{29}Si{^{1}H} NMR** (100 MHz, C_6D_6, 298 K) ***δ***: −3.7 (s, *Si* of HMDS), −2.1 (s, *Si* of HMDS) ppm. **^{113}Cd NMR** (111 MHz, C_6D_6, 298 K) ***δ***: no resonance observed in a range of +850 – -2000 ppm.

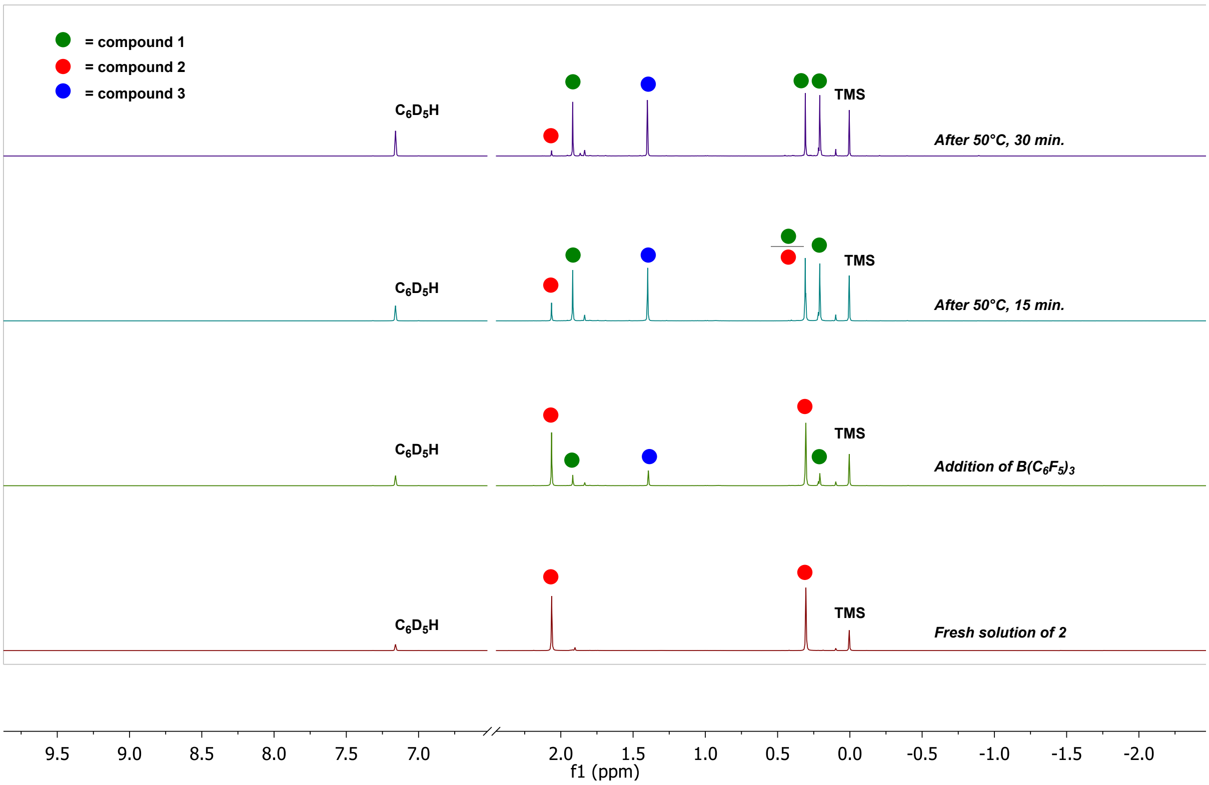

Figure S59: ^{1}H NMR (500 MHz, C_6D_6) screening for reaction of **2** with $B(C_6F_5)_3$.

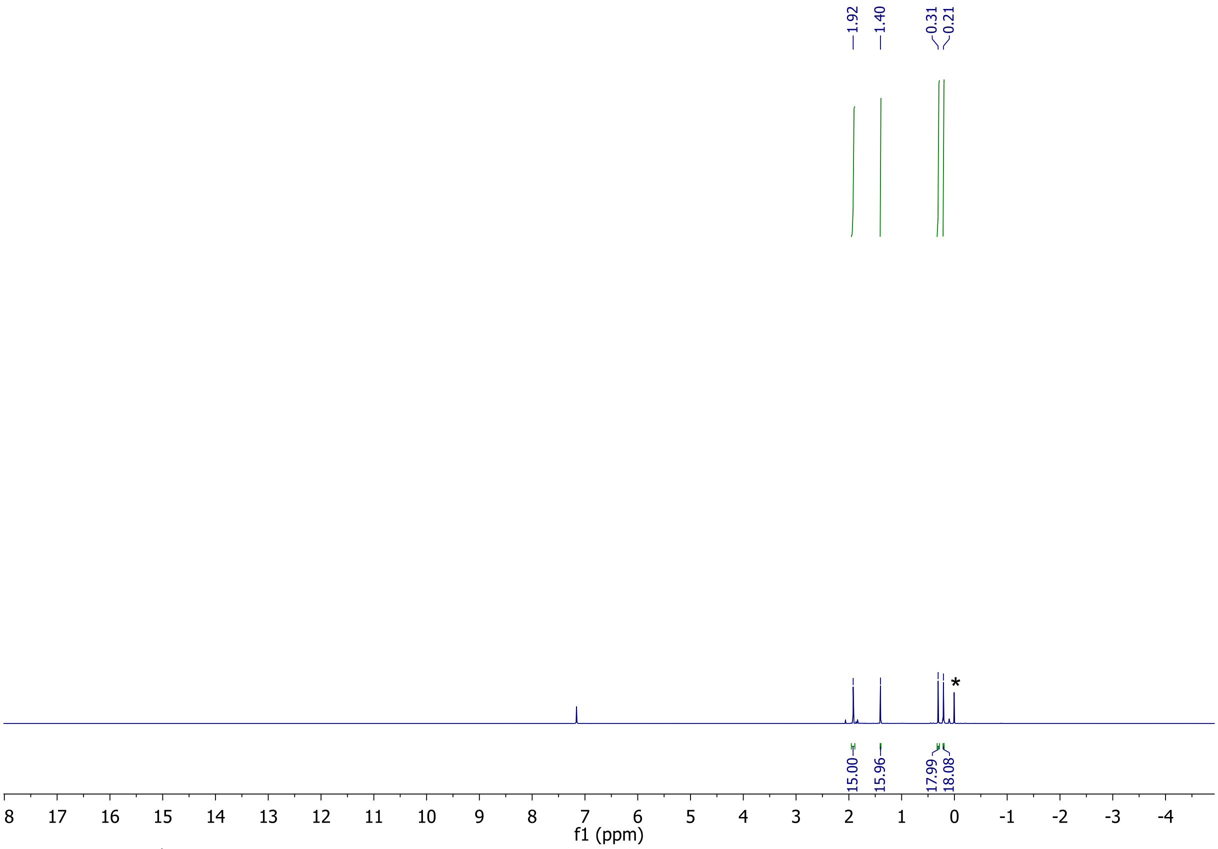

Figure S60: ^{1}H NMR (500 MHz, C_6D_6) for a mixture of **1** and **3** derived from **2** and $B(C_6F_5)_3$. * = TMS.

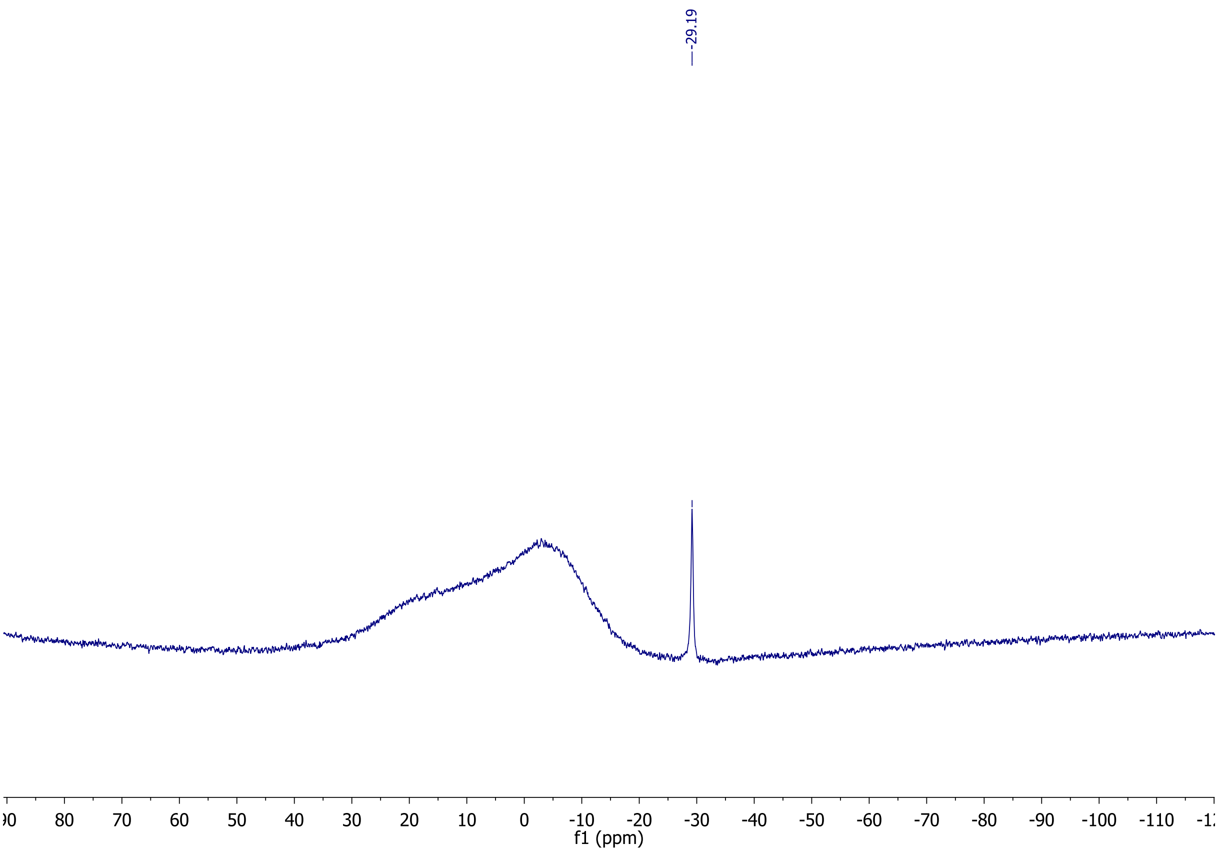

Figure S61: ^{11}B NMR (160 MHz, C_6D_6) of **3**. ~0 ppm = probe head.

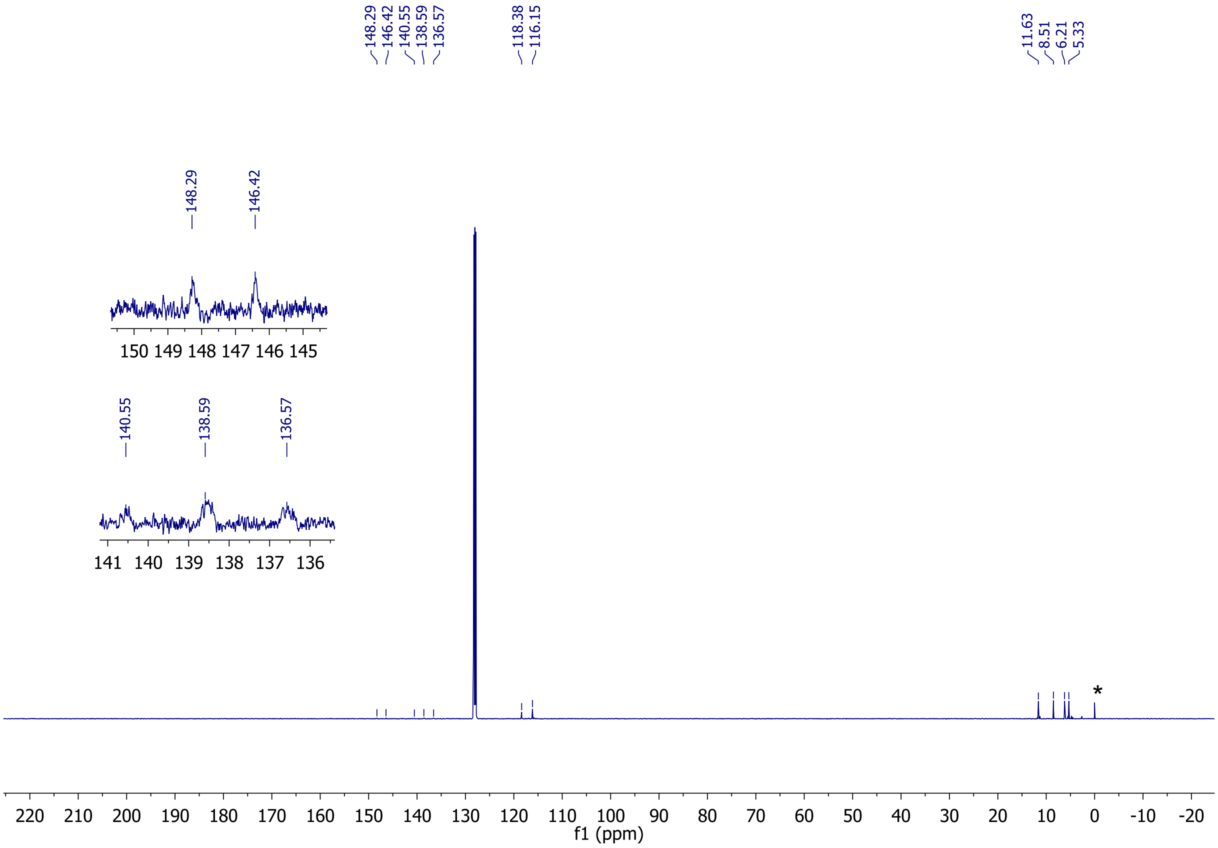

Figure S62: ^{13}C{^{1}H} NMR (126 MHz, C_6D_6) of a mixture of **1** and **3** derived from **2** and $B(C_6F_5)_3$. * = TMS.

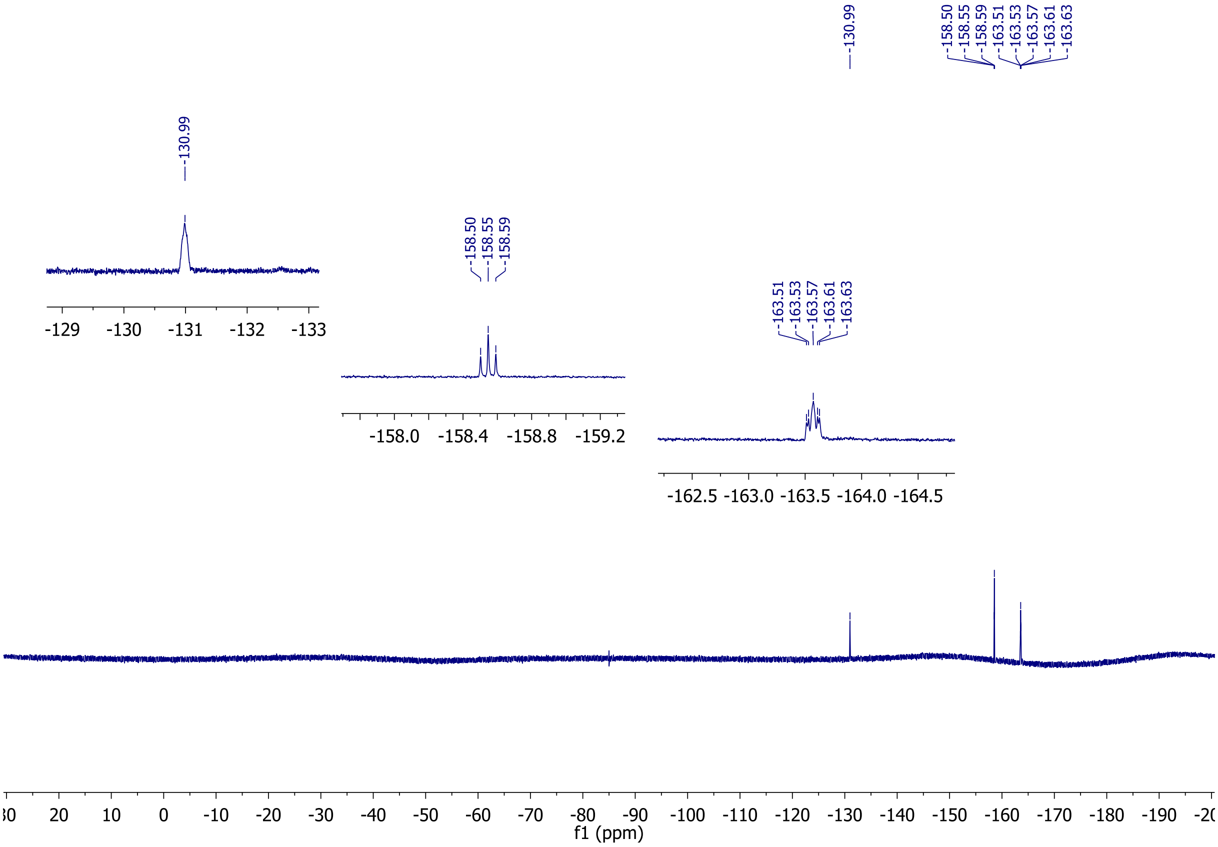

Figure S63: ^{19}F NMR (470 MHz, C_6D_6) of **3**.

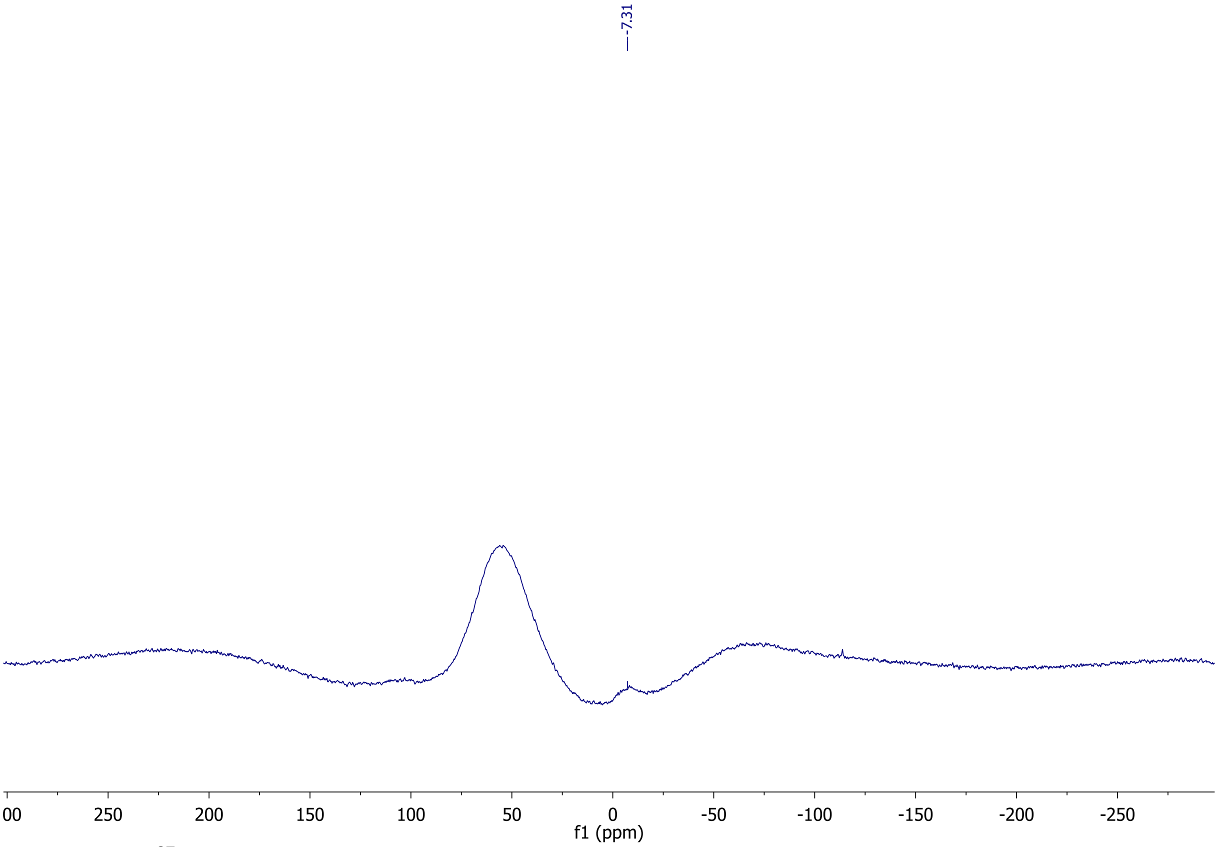

Figure S64: ^{27}Al NMR (130 MHz, C_6D_6) of **7a**. Resonance at ~50 ppm = probe head.

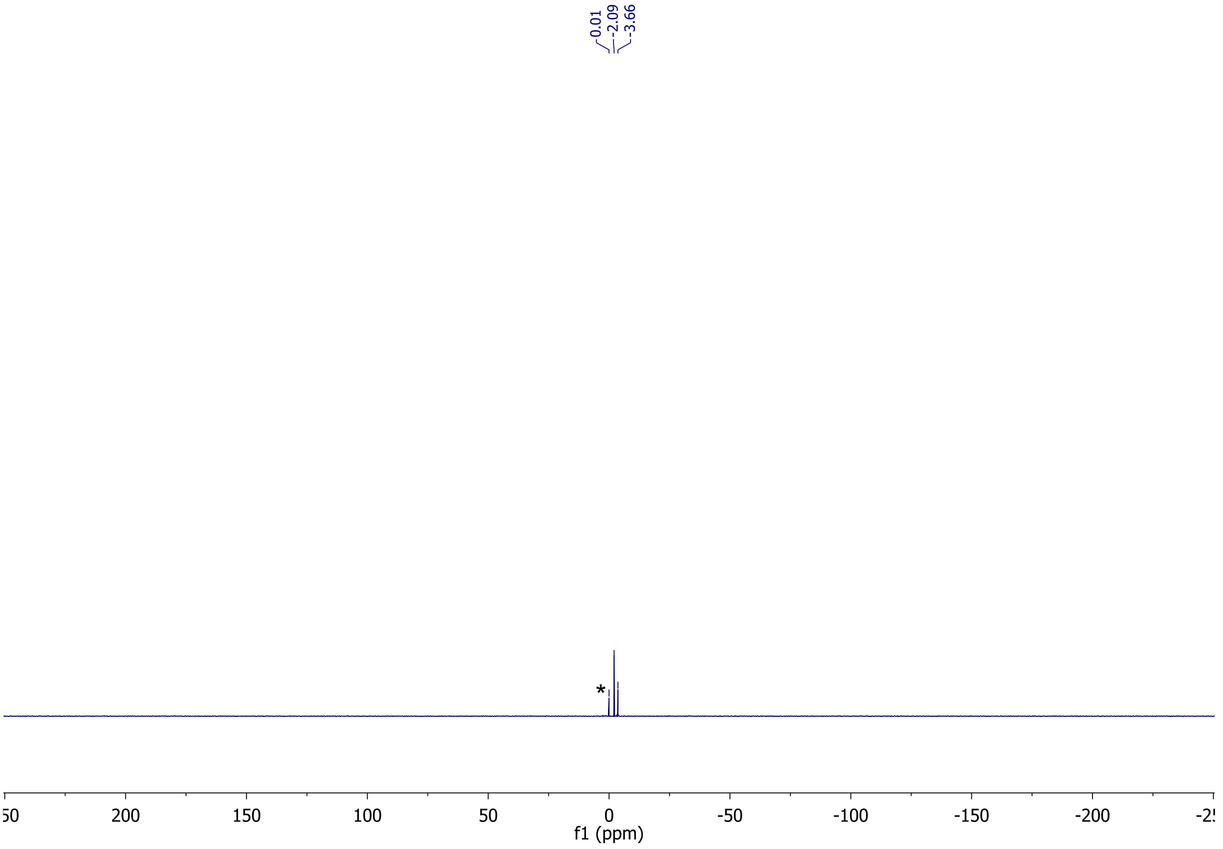

Figure S65: $^{29}Si\{^1H\}$ NMR (99 MHz, C_6D_6) of **1**. * = TMS.

4.2 Reaction of 2 with Cd{N(TMS)$_2$}$_2$

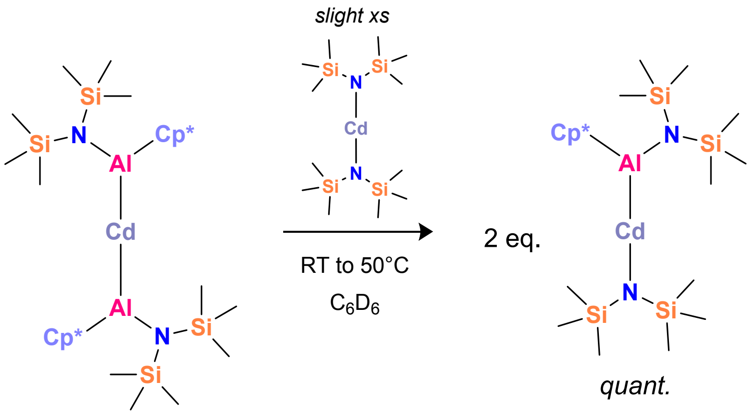

2 was synthesized according to section 4.2 with naphthalene as internal standard (3.6 mg, 0.028 mmol). After detecting quantitative conversion of (AlCp*)$_4$ and Cd{N(TMS)$_2$}$_2$, a slight excess of Cd{N(TMS)$_2$}$_2$ (0.045 mmol, 1.125 eq.), dissolved in 0.4 mL of C_6D_6, was immediately added. The reaction was monitored directly after the addition of Cd{N(TMS)$_2$}$_2$ and after heating for short periods at 50°C. After ten minutes, a quantitative conversion of **2** to **1** is observed.

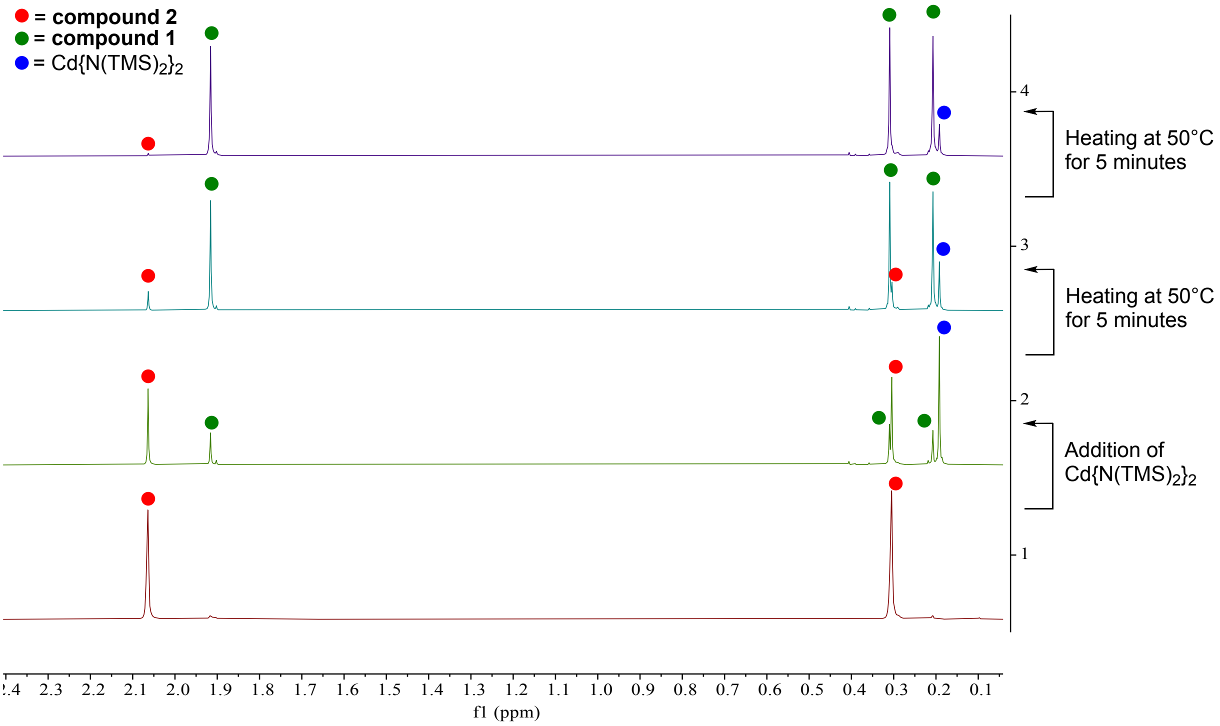

Figure S66: ^{1}H NMR (500 MHz, C_6D_6) screening for AlCp* transfer onto Cd{N(TMS)$_2$}$_2$ using **2**.

4.3 Reaction of 2 with Cd(TMP)$_2$

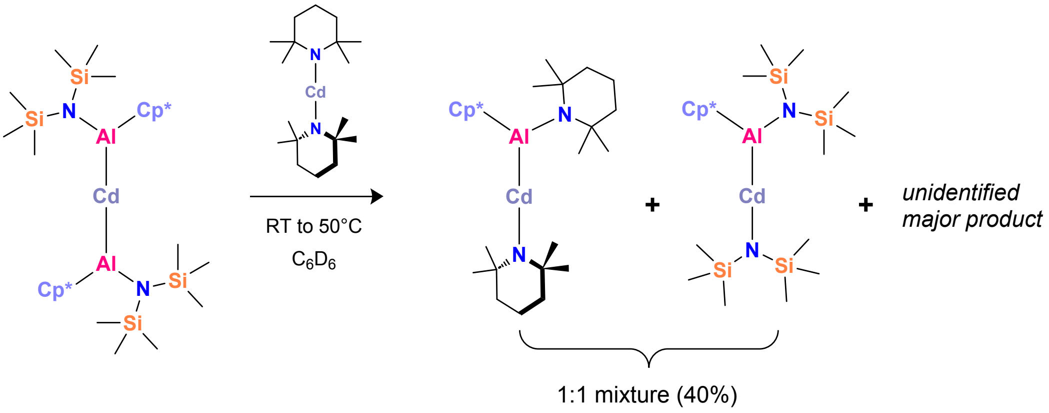

---

2 was synthesized according to section 4.2 with TMS as internal standard (8 µL, 0.059 mmol). After detecting quantitative conversion of $(AlCp^*)_4$ and Cd{N(TMS)$_2$}$_2$, stoichiometric amounts of Cd(TMP)$_2$ (15.7 mg, 0.040 mmol, 1.0 eq.), dissolved in 0.4 mL of C_6D_6, were immediately added. The reaction was monitored directly after the addition of Cd(TMP)$_2$ and after heating for two 15 min. periods at 50°C. After 30 minutes, quantitative conversion of **2** is observed. A 1:1 mixture of **1** and **4** as well as a major unidentified product form which is allegedly due to scrambling of ligands. The identification of **4** and **1** is beyond reasonable doubt due to the excellent fit of NMR data with rationally synthesized **4** and **1** in sections 4.3 and 4.1, respectively. The unidentified species, however, could not be identified. We tried isolating the species, however, similar as described in section 4.3, large amounts of black precipitates precluded isolating the compound and its characterization. An illustration of the isolated black material is depicted on the right.

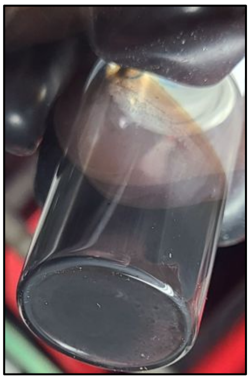

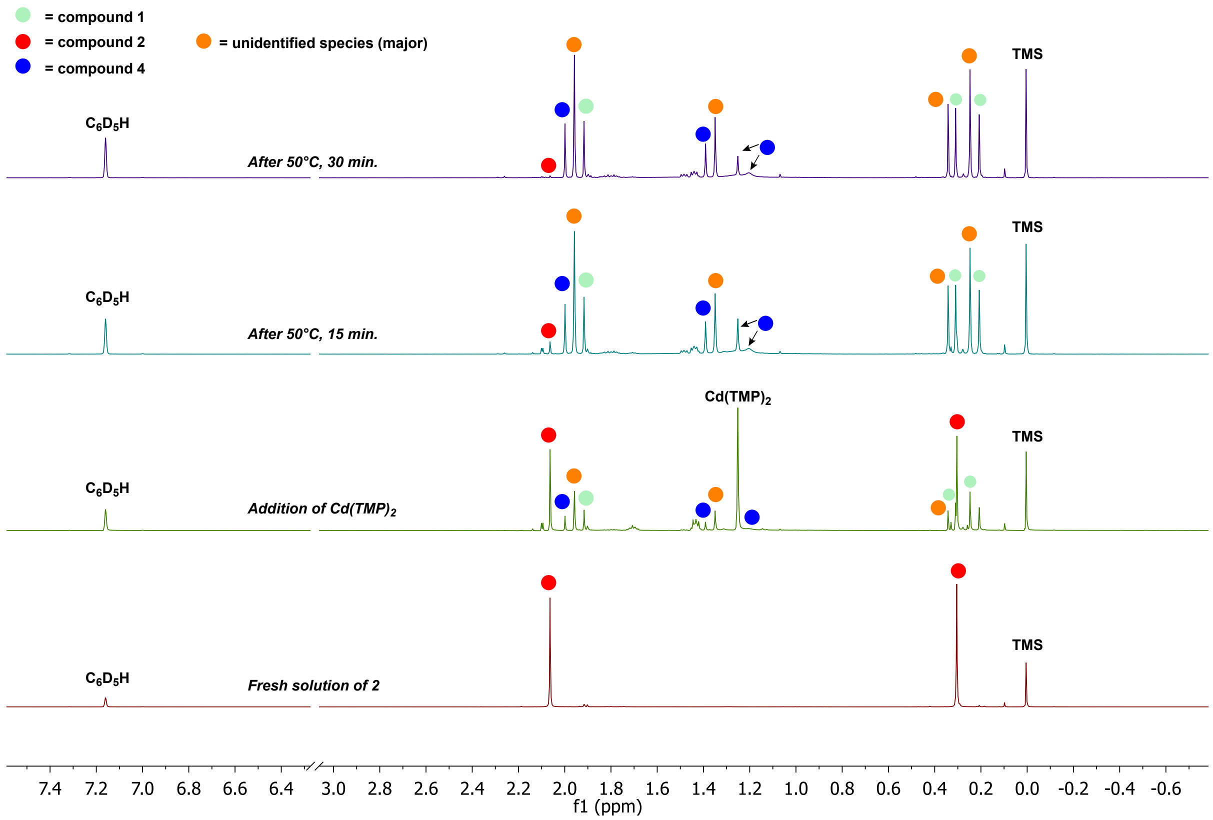

Figure S67: ^{1}H NMR (500 MHz, C_6D_6) screening for AlCp* transfer onto Cd(TMP)$_2$ using **2**. Only -CH_3 resonances have been tagged for clarity.

4.4 Reaction of 2 with $Zn\{N(TMS)_2\}_2$

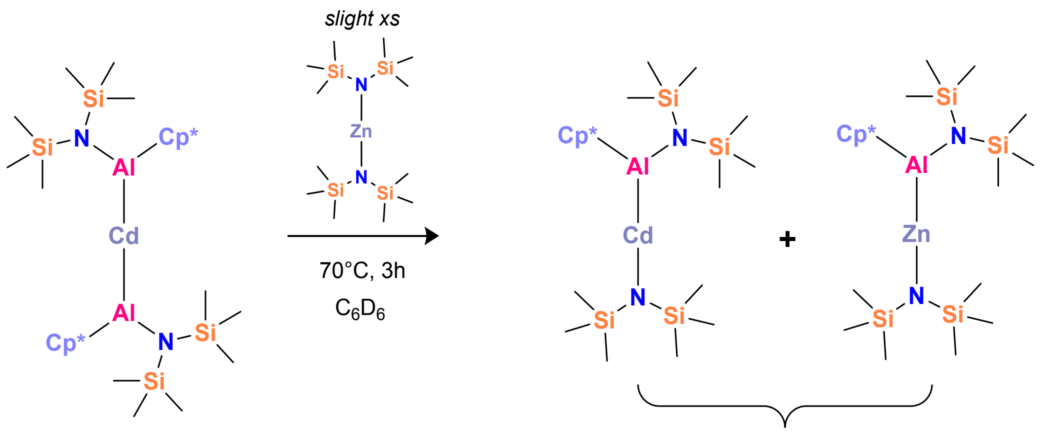

2 was synthesized according to section 4.2 with TMS as internal standard (8 µL, 0.059 mmol). After detecting quantitative conversion of $(AlCp^*)_4$ and $Cd\{N(TMS)_2\}_2$, a slight excess of $Zn\{N(TMS)_2\}_2$ dissolved in 0.4 mL of C_6D_6, was immediately added. The reaction was monitored directly after the addition of $Zn\{N(TMS)_2\}_2$ and after heating for one-hour periods at 70°C. After three hours, 68% conversion of **2** is observed furnishing a 1:1 mixture of **1** and **1^{Zn}**. NMR data agrees with the literature.[7]

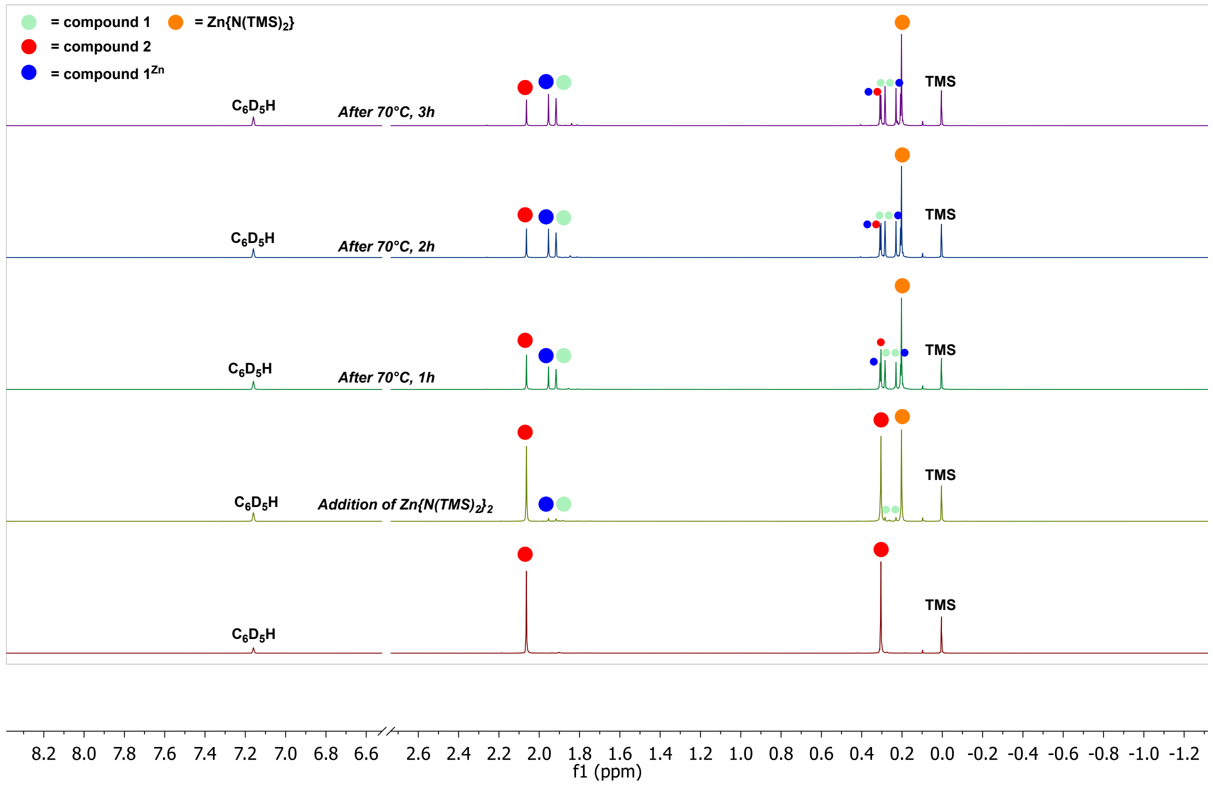

Figure S68: ^1H NMR (500 MHz, C_6D_6) screening for AlCp* transfer onto $Zn\{N(TMS)_2\}$ using **2**. Only -*C*H_3 resonances have been tagged for clarity.

4.5 Reaction of 2 with ^{t}Bu$_3$P-Ag{N(TMS)$_2$} (^{pre}5)

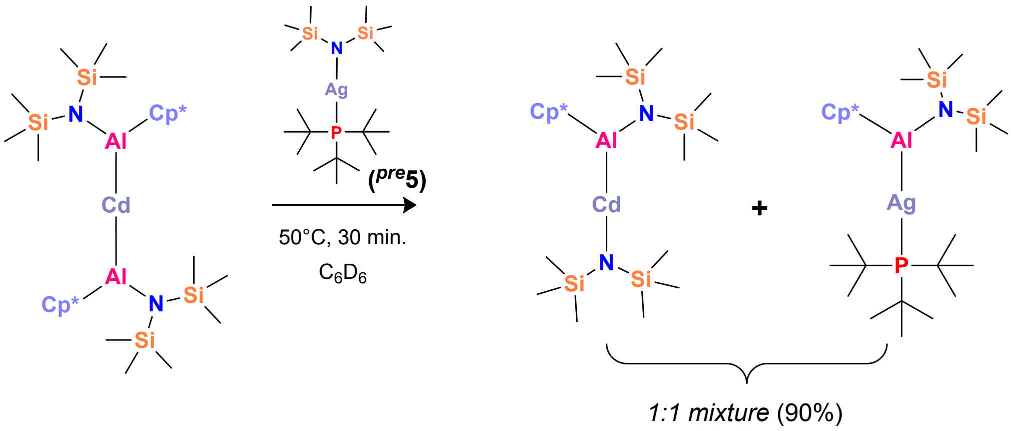

2 was synthesized according to section 4.2 with TMS as internal standard (8 µL, 0.059 mmol). After detecting quantitative conversion of (AlCp*)$_4$ and Cd{N(TMS)$_2$}$_2$, stoichiometric amounts of ^{pre}**5** (18.8 mg, 0.040 mmol, 1.0 eq.), dissolved in 0.4 mL of C_6D_6, were immediately added. The reaction was monitored directly after the addition of ^{pre}**5** and after heating for two 15 min. periods at 50°C. After 30 minutes, quantitative conversion of ^{pre}**5** is observed. An approximate 1:1 mixture of **1** and **5** (90% NMR spectroscopically determined yield).

^{1}H NMR (500 MHz, C_6D_6, 298 K) ***δ***: 2.25 (s, 15H, C*H*$_3$ of Cp* of **5**), 1.92 (s, 15H, C*H*$_3$ of Cp* of **1**), 1.12 (d, $^3J_{PH}$ = 12.1 Hz, 27H, –C*H*$_3$ of ^{t}Bu$_3$P of **5**), 0.48 (s, 18H, –C*H*$_3$ of HMDS of **5**), 0.31 (s, 18H, –C*H*$_3$ of HMDS of **1**), 0.21 (s, 18H, –C*H*$_3$ of HMDS of **1**) ppm. **^{13}C{^{1}H} NMR** (126 MHz, C_6D_6, 298 K) ***δ***: 118.4 (C_q of Cp* of **1**), 116 (ps dd, *J* = 4.0; 1.2 Hz, C_q of Cp*), 37.2 (ps dd, *J* = 4.4; 1.6 Hz, (*C*(CH$_3$)$_3$)$_3$ of ^{t}Bu$_3$P of **5**), 32.3 (d, $^2J_{CP}$ = 6.9 Hz (C(*C*H$_3$)$_3$)$_3$ of ^{t}Bu$_3$P), 12.5 (s, *C*H$_3$ of Cp* of **5**), 11.6 (s, *C*H$_3$ of Cp* of **1**), 6.3+6.3 (s, *C*H$_3$ of HMDS of **5**), 6.2 (s, *C*H$_3$ of HMDS of **1**), 5.3 (s, *C*H$_3$ of HMDS of **1**) ppm. ***Note:*** The coupling of various ^{13}C NMR resonances of **5** was resolved in this mixture and thus the analytical data differs slightly to as above reported. **^{29}Si{^{1}H} NMR** (100 MHz, C_6D_6, 298 K) ***δ***: −2.1 (s, *Si* of HMDS of **1**), −3.6 (s, *Si* of HMDS of **1**), −5.2 (s, *Si* of HMDS of **5**) ppm. **^{31}P{^{1}H} NMR** (202 MHz, C_6D_6) ***δ***: 61.3 (br d, *J* = 158 Hz; ^{t}Bu$_3$*P* of **5**) ppm.

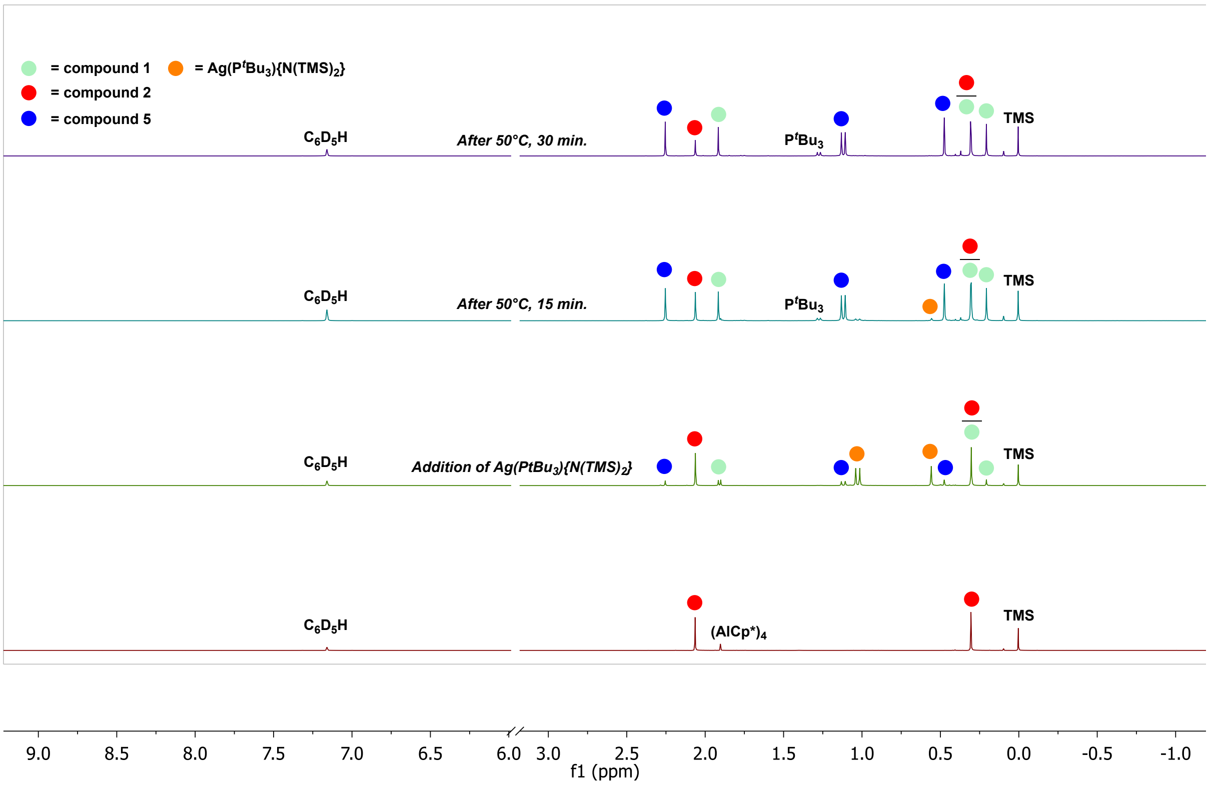

Figure S69: ^{1}H NMR (500 MHz, C_6D_6) screening for reaction of **2** with ***pre*5**.

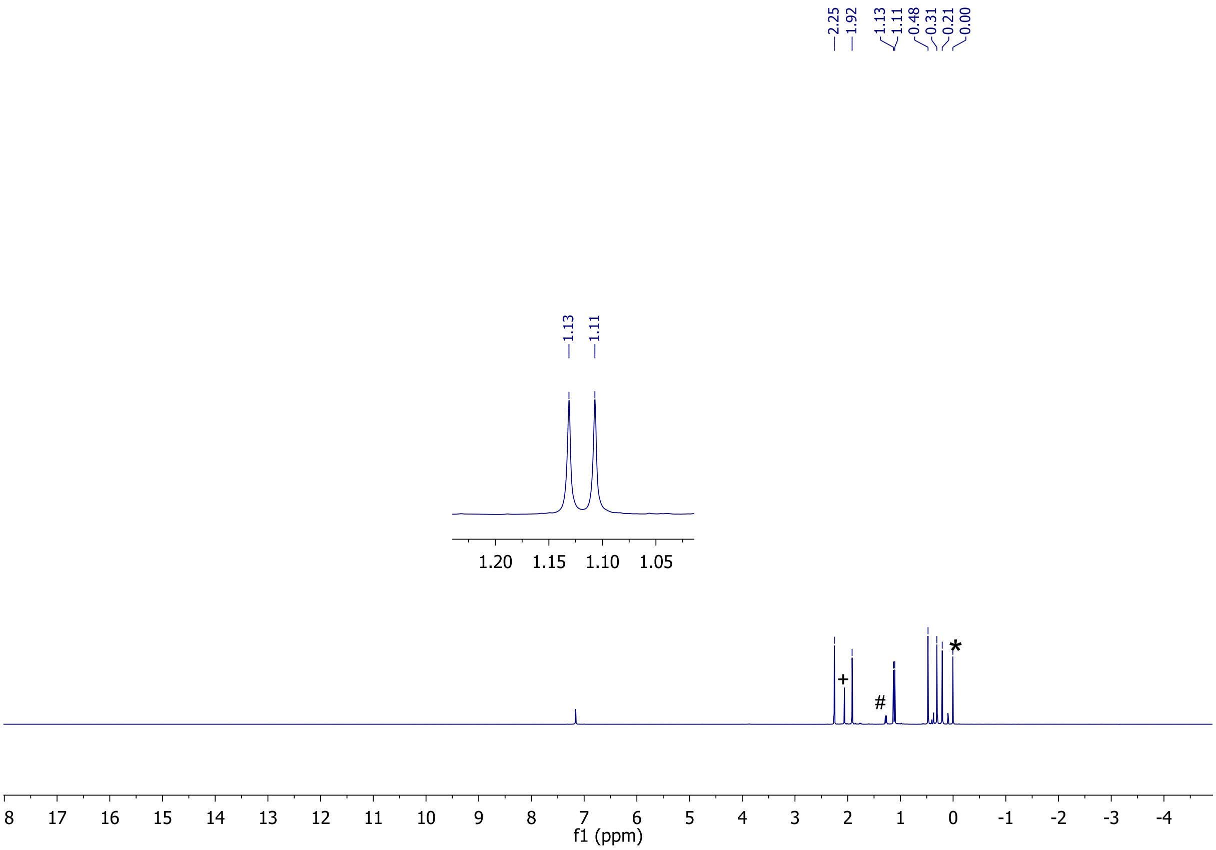

Figure S70: ^{1}H NMR (500 MHz, C_6D_6) of a mixture of **1** and **5** obtained from **2** and ***pre*5**. * = TMS; + = residual **2**; # = trace P^{t}Bu$_3$.

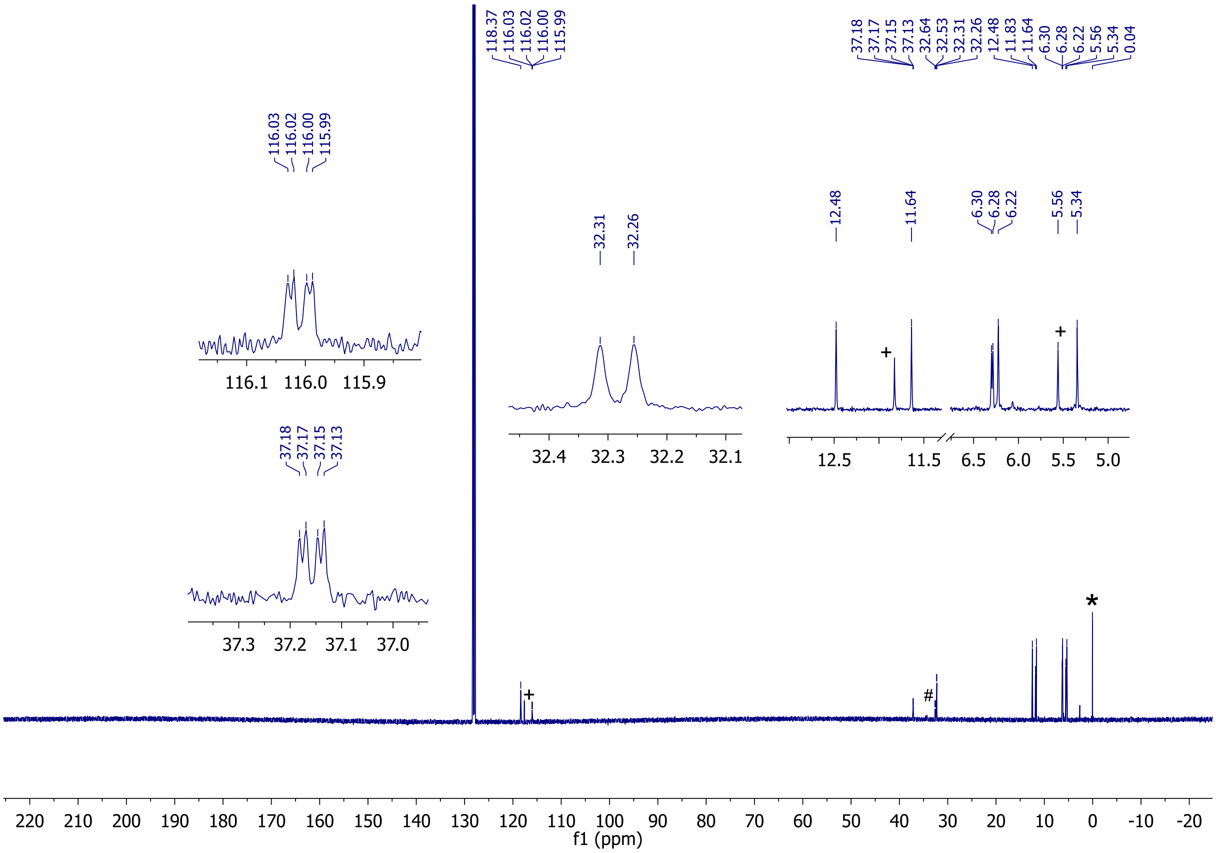

Figure S71: $^{13}C\{^1H\}$ NMR (126 MHz, C_6D_6) of a mixture of **1** and **5** derived from **2** and ***pre*5**. * = TMS; + = residual **2**; # = trace P^tBu_3.

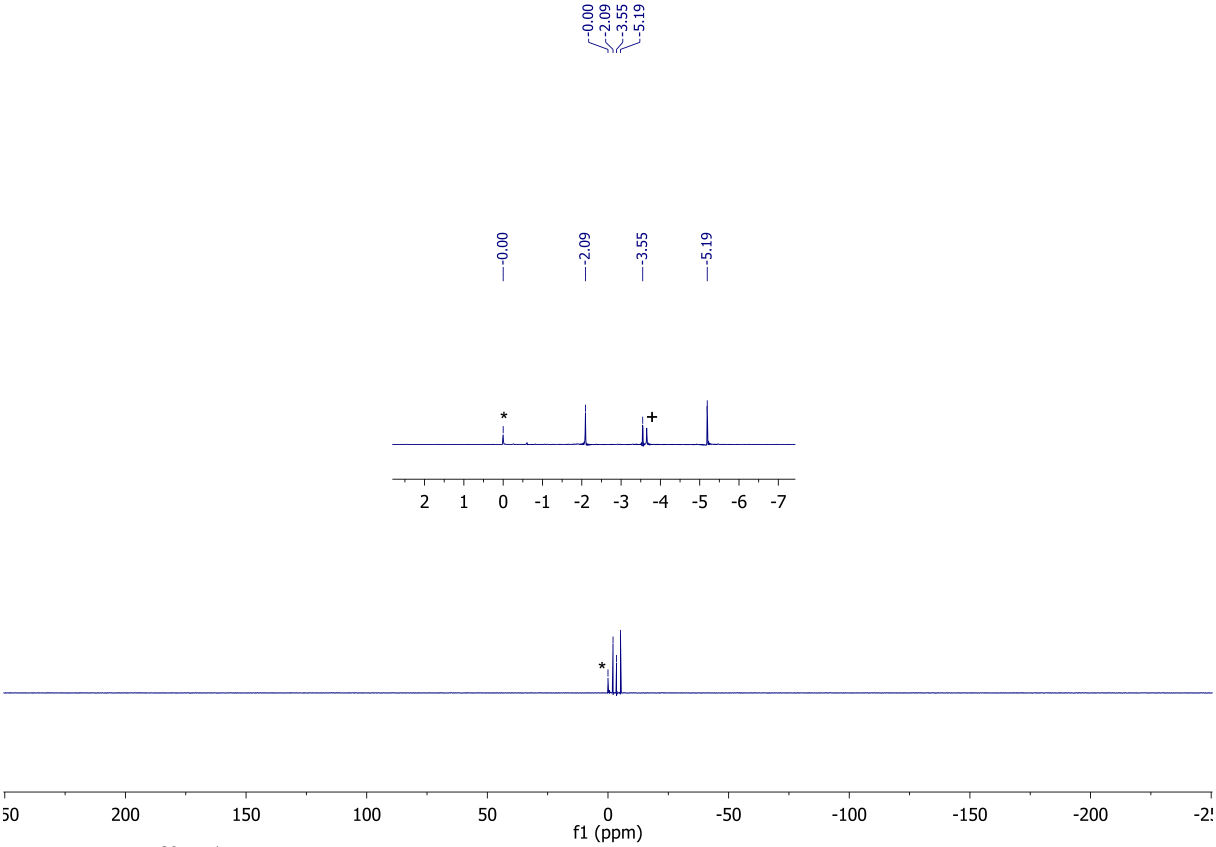

Figure S72: $^{29}Si\{^1H\}$ NMR (99 MHz, C_6D_6) of a mixture of **1** and **5** derived from **2** and ***pre*5**. * = TMS; + = residual **2**.

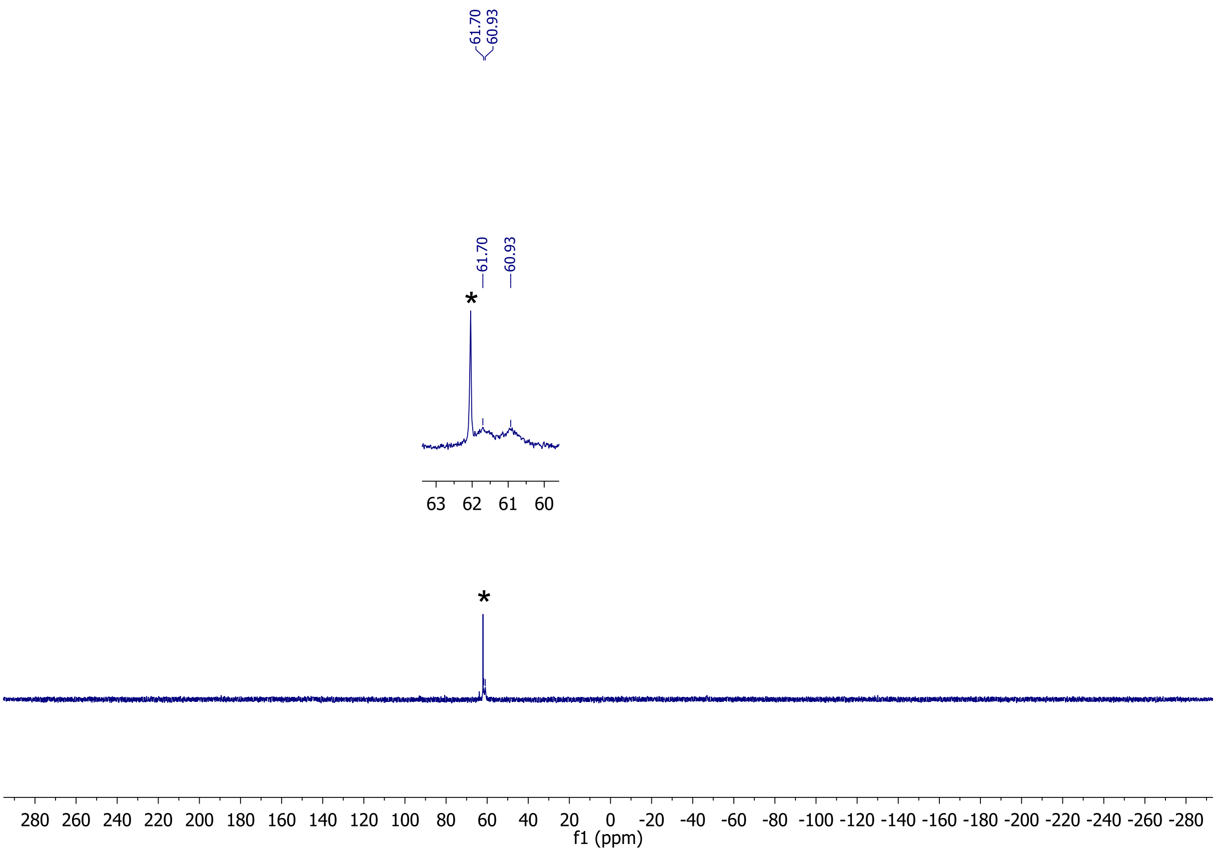

Figure S73: $^{31}P\{^{1}H\}$ NMR (99 MHz, C_6D_6) of **5**. * = trace $P^{t}Bu_3$.

5 Additional NMR studies

5.1 Thermal treatment of [({N(TMS)$_2$})(Cp*)Al–Cd({N(TMS)$_2$})] (**1**)

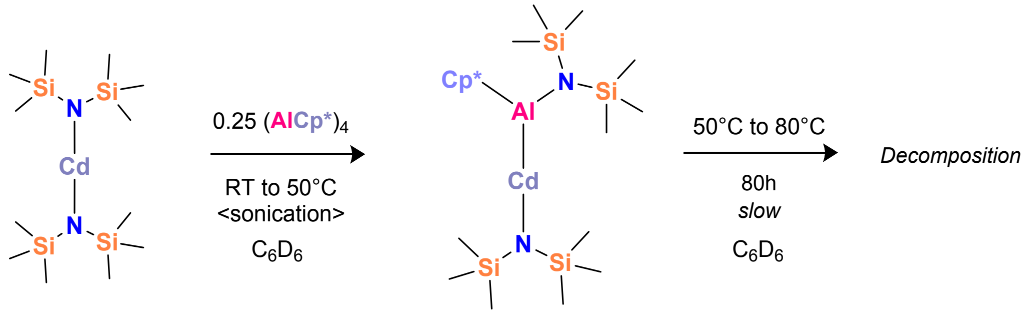

A fresh solution of **1** was synthesized on NMR scale and applied to heat over the course of 80h. Precipitation of metallic powders proceeds only slowly. We screened the formation of **1** by ^{1}H NMR and analyzed a final decomposition product by means of multinuclear NMR spectroscopy (Figures S74-S77). Regarding ^{27}Al NMR (130 MHz, C_6D_6, 298 K): No resonance for decomposition product(s) observed in a range of +300 – -300 ppm.

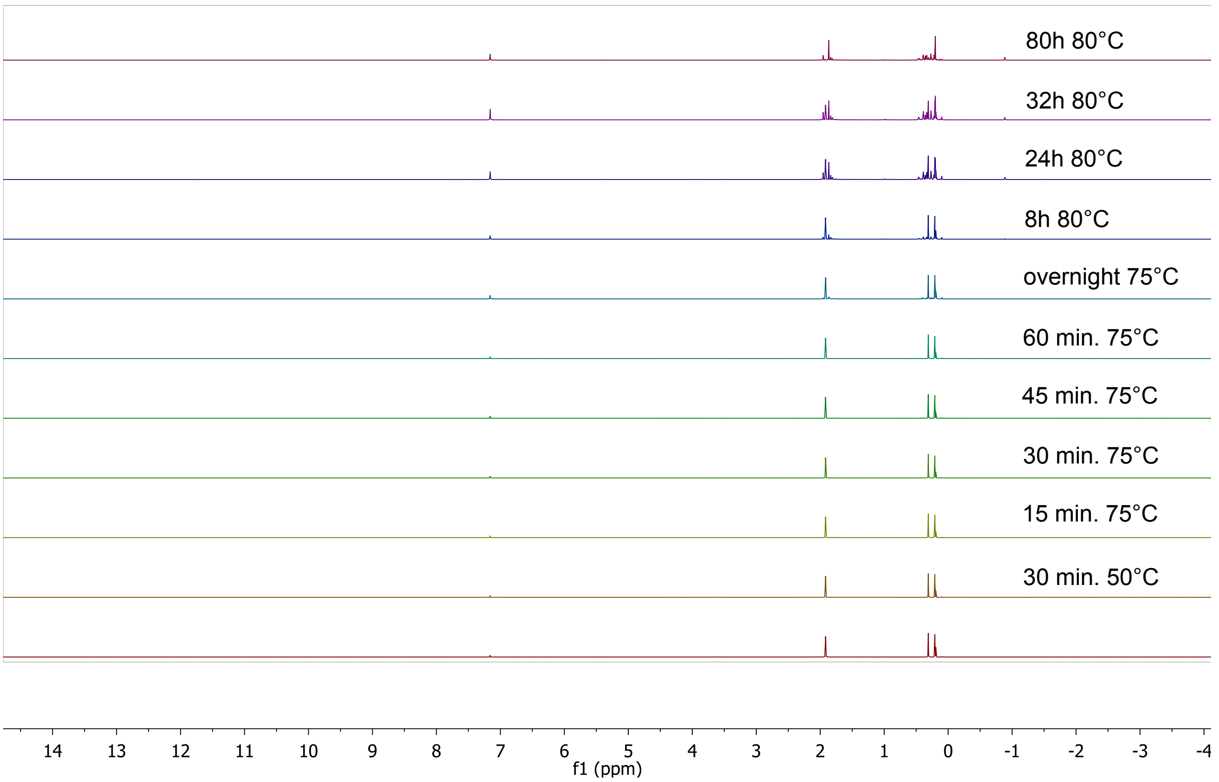

Figure S74: ^{1}H NMR (500 MHz, C_6D_6) screening for the thermal decomposition of **1** at irregular time intervals.

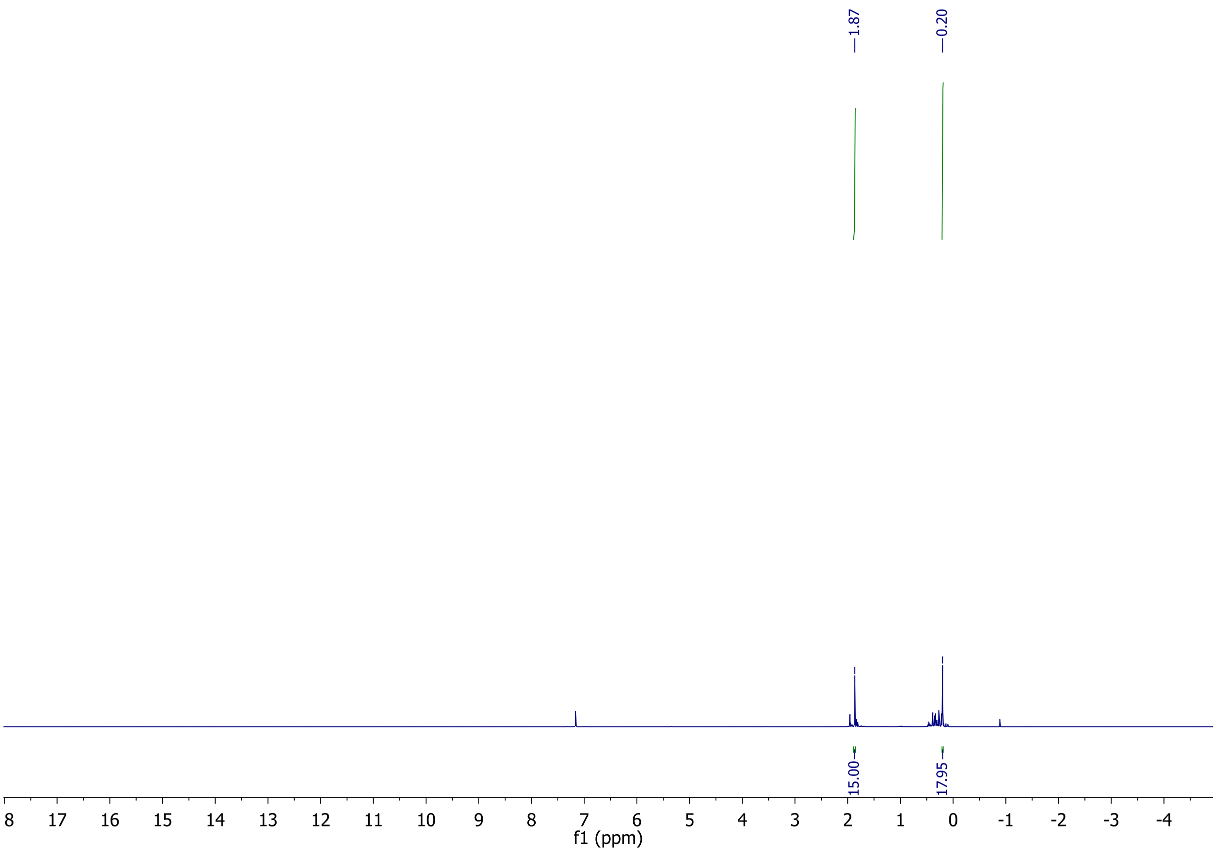

Figure S75: ^{1}H NMR (500 MHz, C_6D_6) of the final decomposition product (80h at 80°C) showing a 1:1 ratio of -{$N(TMS)_2$} groups and Cp*. Integration only done for the two major resonances.

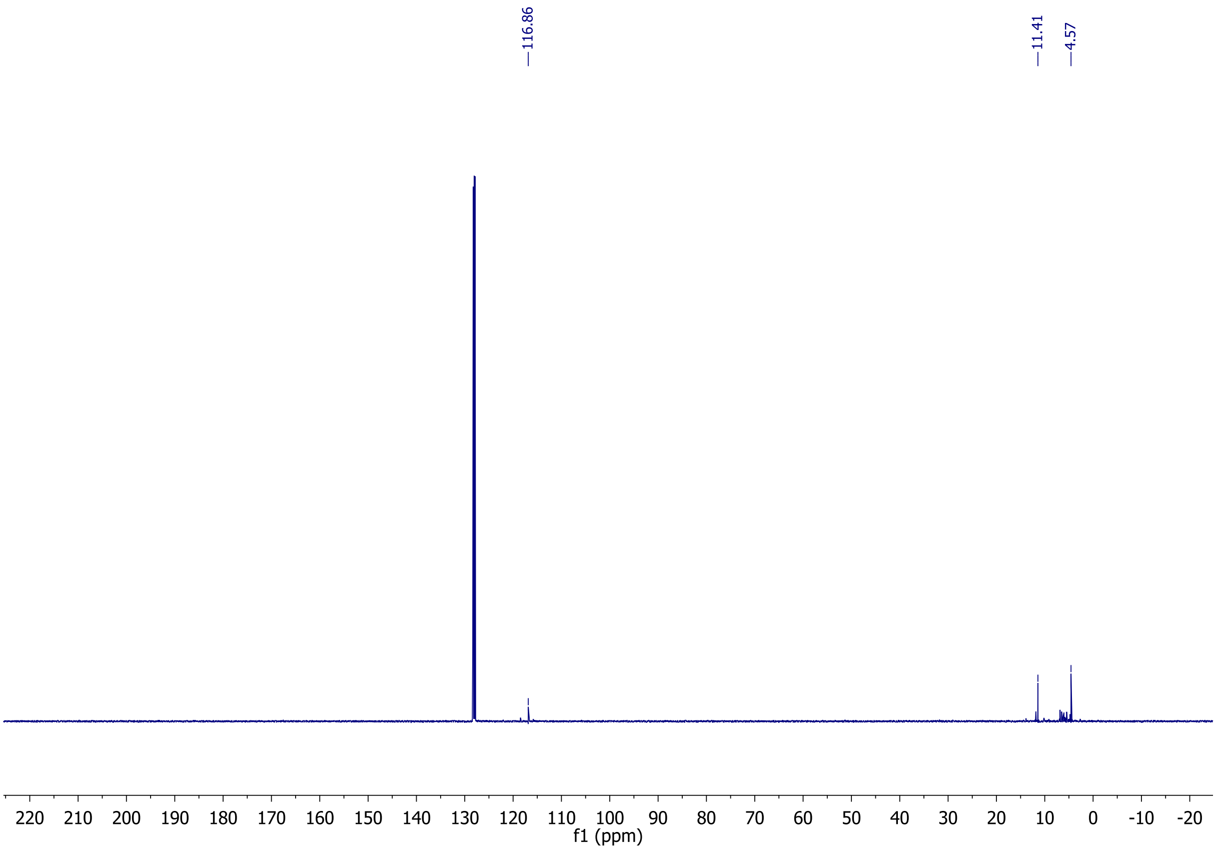

Figure S76: $^{13}C\{^{1}H\}$ NMR (126 MHz, C_6D_6) of the final decomposition product (80h at 80°C). Signal assignment only done for major resonances.

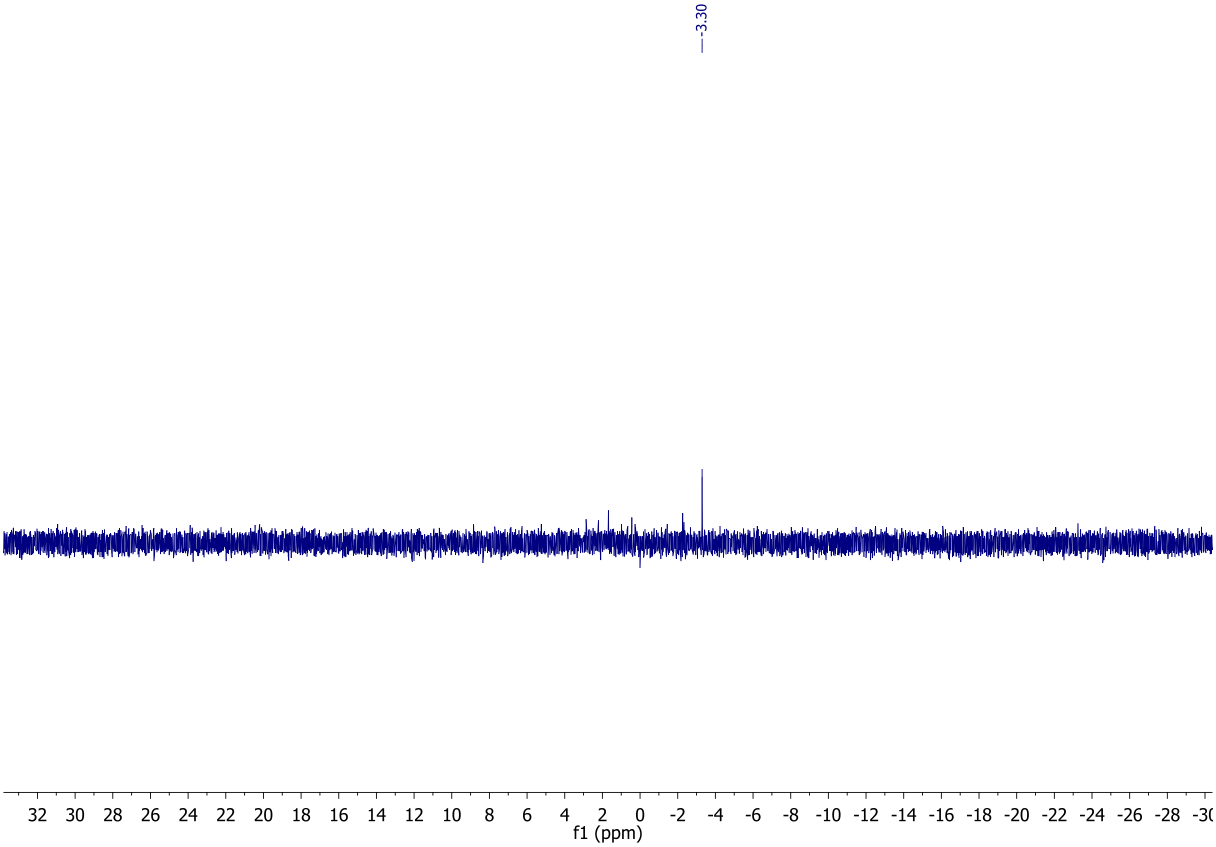

Figure S77: $^{29}Si\{^{1}H\}$ NMR (99 MHz, C_6D_6) of the final decomposition product (80h at 80°C).

5.2 Thermal treatment of [{N(TMS)$_2$}(Cp*)Al]$_2$Cd (2)

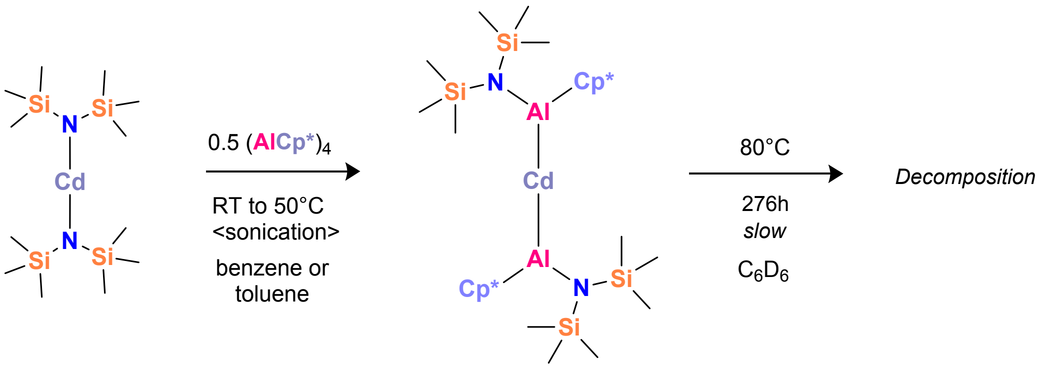

A fresh solution of **2** was synthesized on NMR scale and applied to heat over the course of 276 hours. Precipitation of metallic powders proceeds very slowly. We screened the formation of **2** by ^{1}H NMR and analyzed the selective, but very slow formation of a new species by means of multinuclear NMR spectroscopy upon heating (Figures S78-S82).

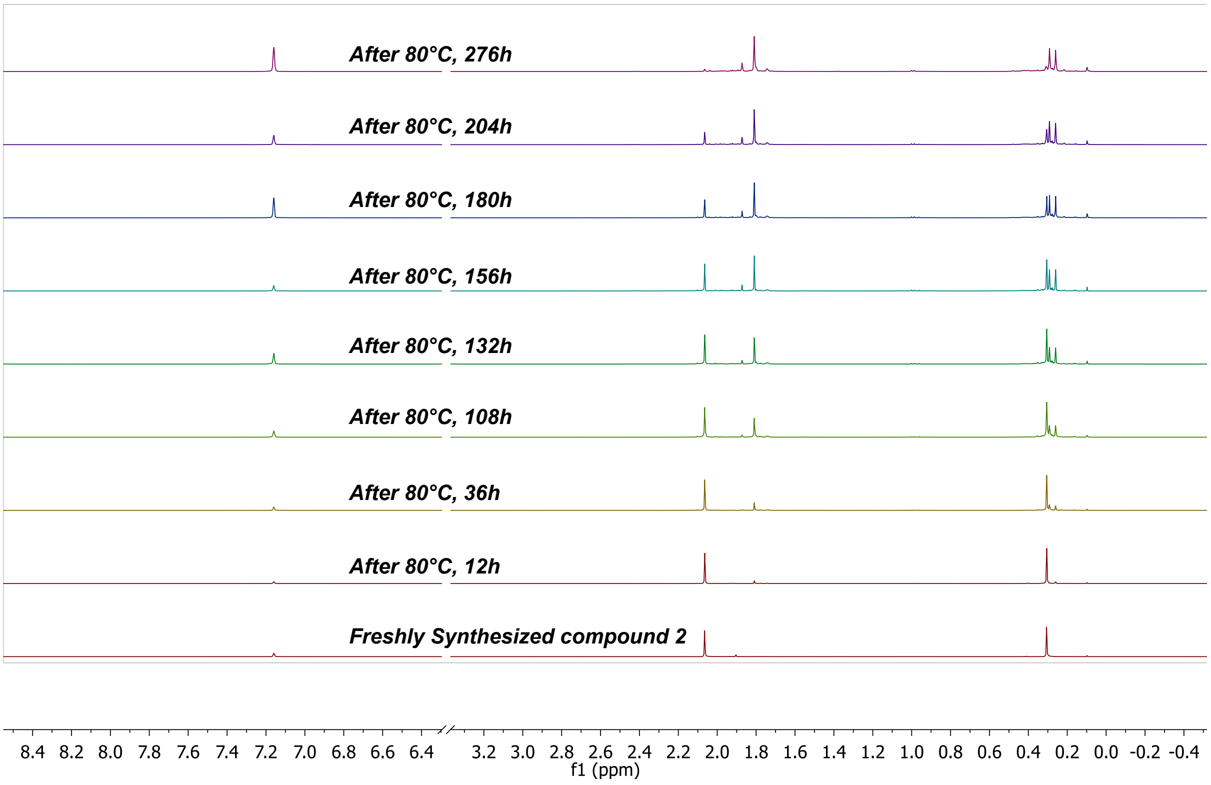

Figure S78: ^{1}H NMR (500 MHz, C_6D_6) screening for the thermal decomposition of **2** at irregular time intervals.

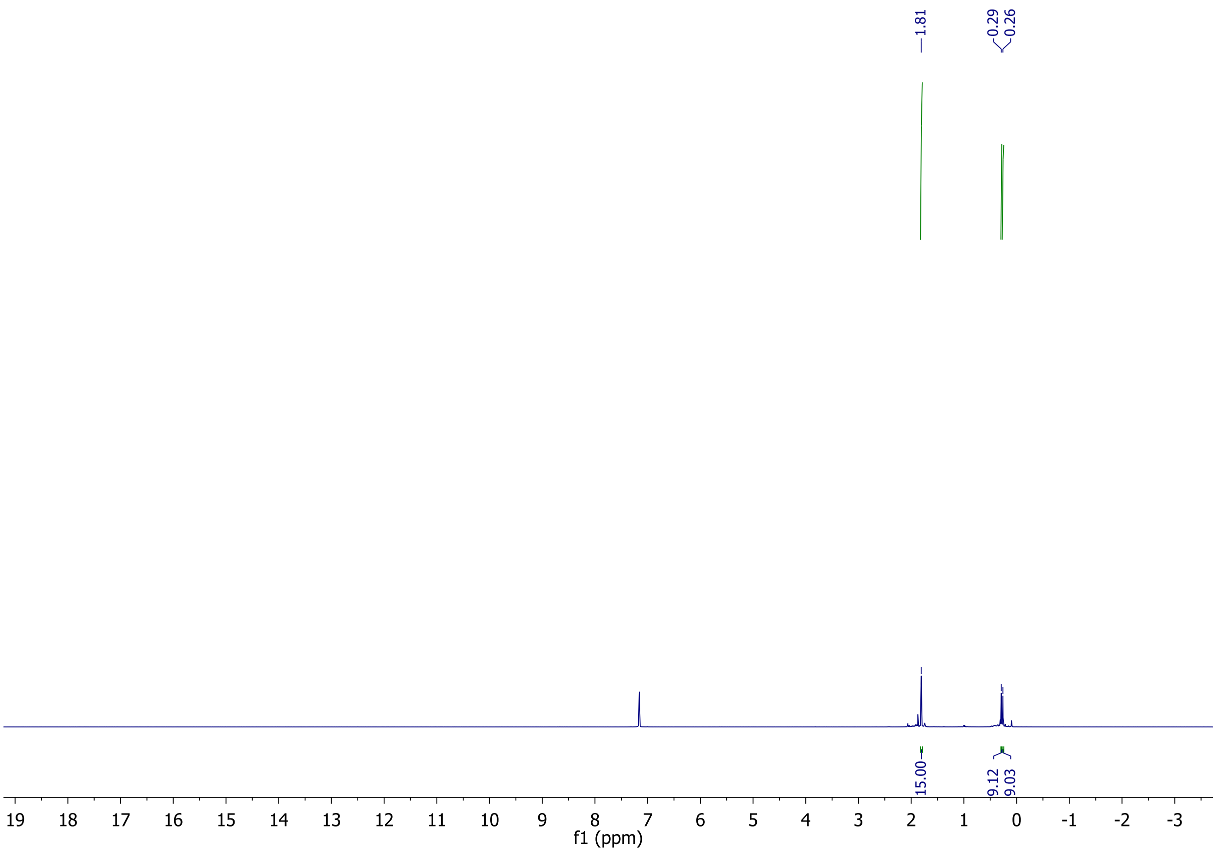

Figure S79: ^{1}H NMR (500 MHz, C_6D_6) of the final decomposition product (276h at 80°C) showing a formal 1:2 ratio of -{N(TMS)$_2$} groups and Cp*. Integration only done for the three major resonances.

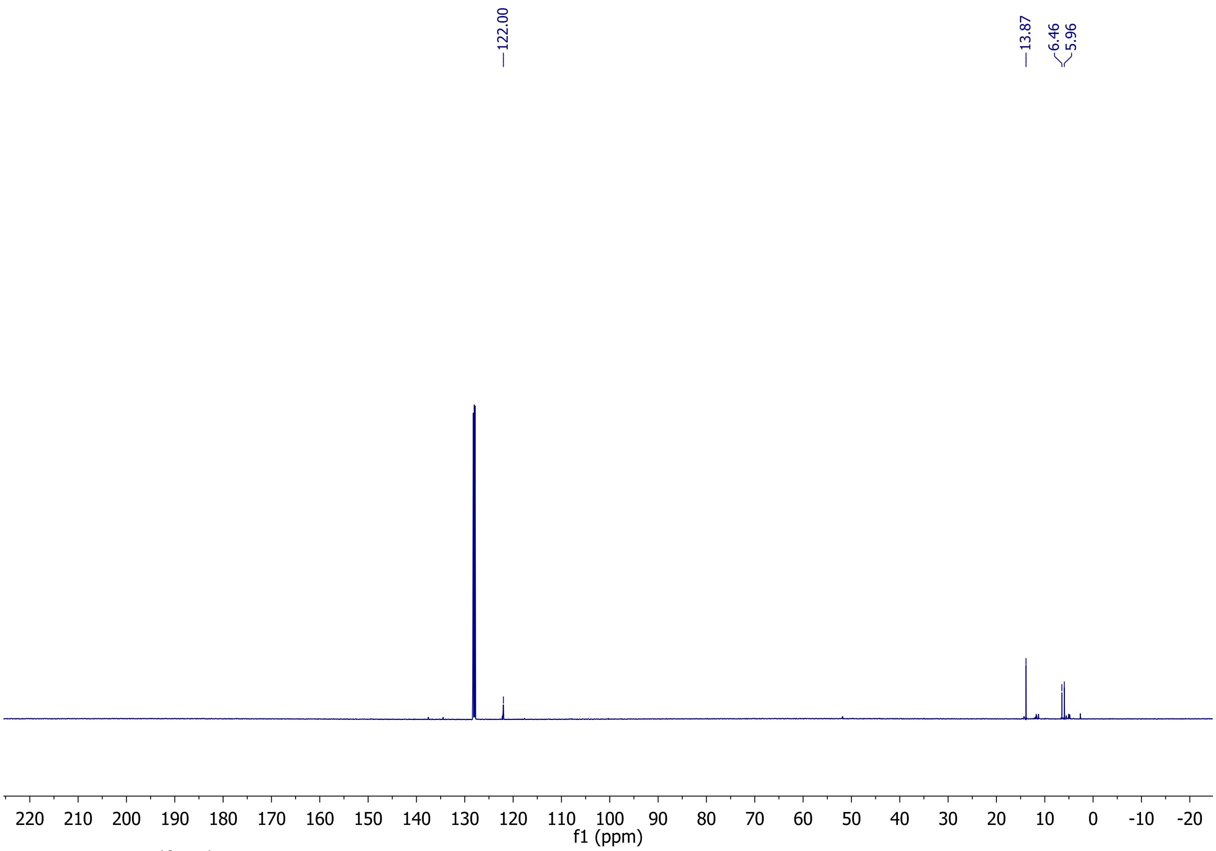

Figure S80: $^{13}C\{^{1}H\}$ NMR (126 MHz, C_6D_6) of the final decomposition product (276h at 80°C). Signal assignment only done for major resonances.

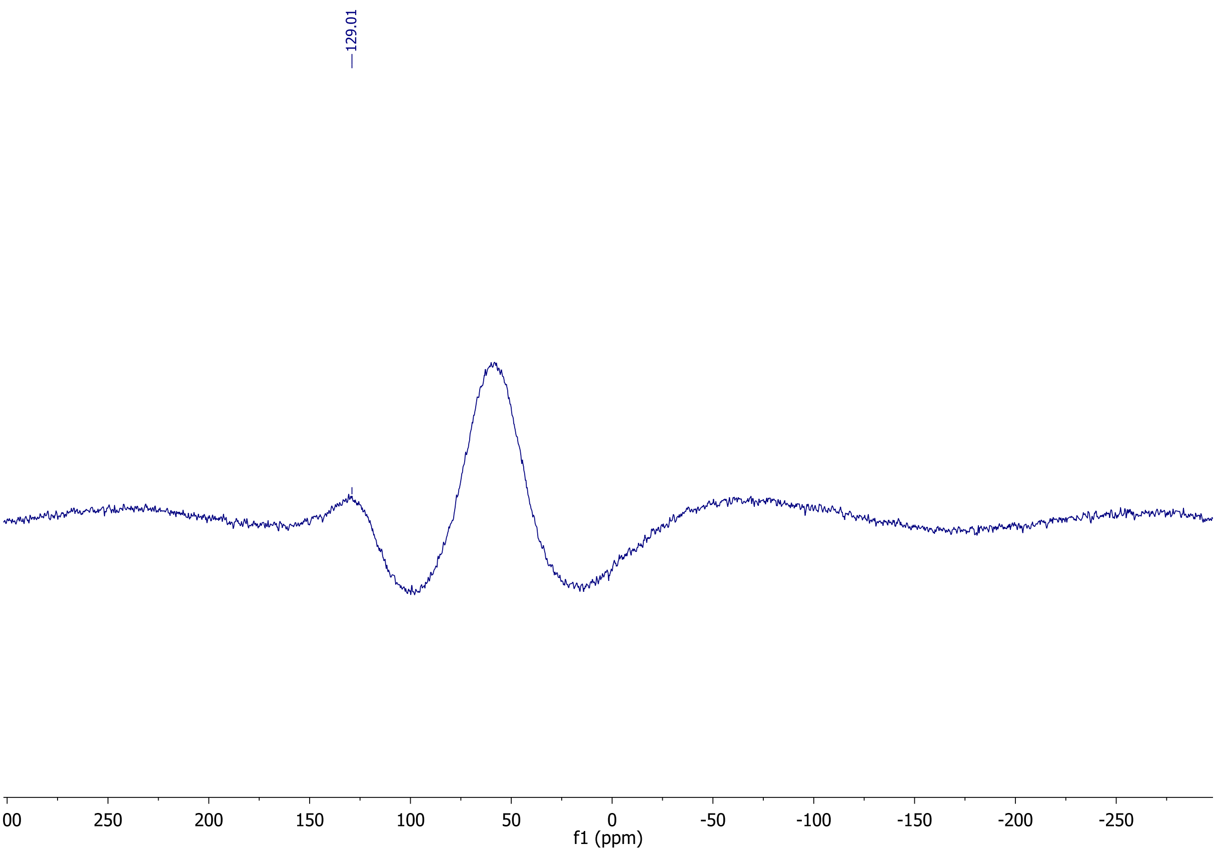

Figure S81: ^{27}Al NMR (130 MHz, C_6D_6) of the final decomposition product (276h at 80°C). Resonance at ~50 ppm = probe head.

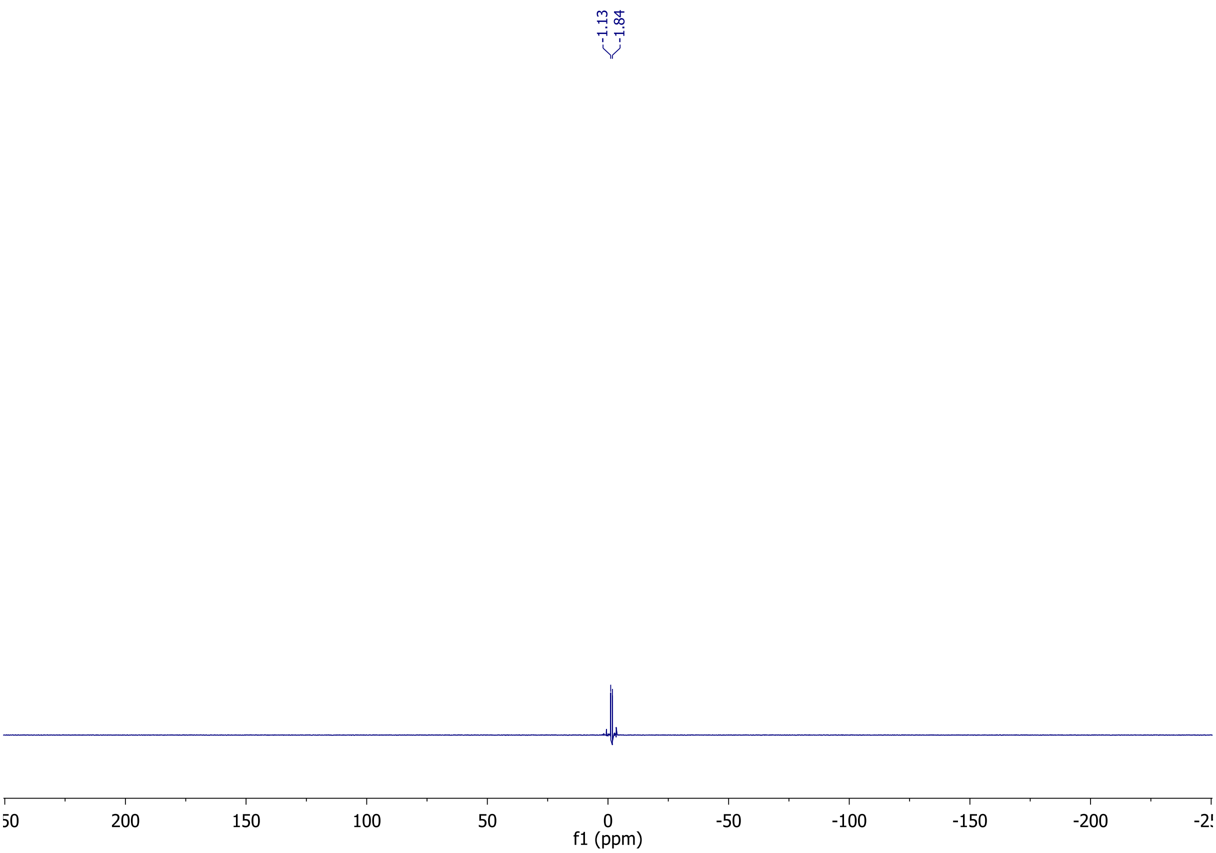

Figure S82: ^{29}Si{^{1}H} NMR (99 MHz, C_6D_6) of the final decomposition product (276h at 80°C).

5.3 Reaction of $B(C_6F_5)_3$ with **1**

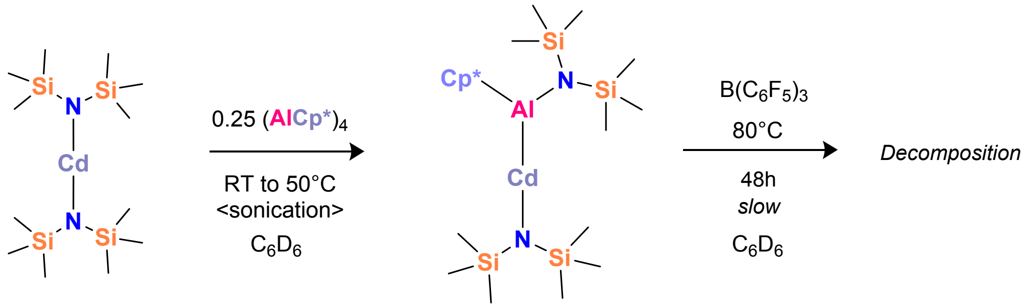

1 was synthesized according to section 4.1. After a good conversion was detected, 15 mg of $B(C_6F_5)_3$ (0.04 mmol, 1 eq.), dissolved in 0.4 ml of C_6D_6, was added to the *J. Young* NMR tube. No immediate change could be observed. Even after heating, no conversion could be determined. Prolonged heating eventually causes decomposition.

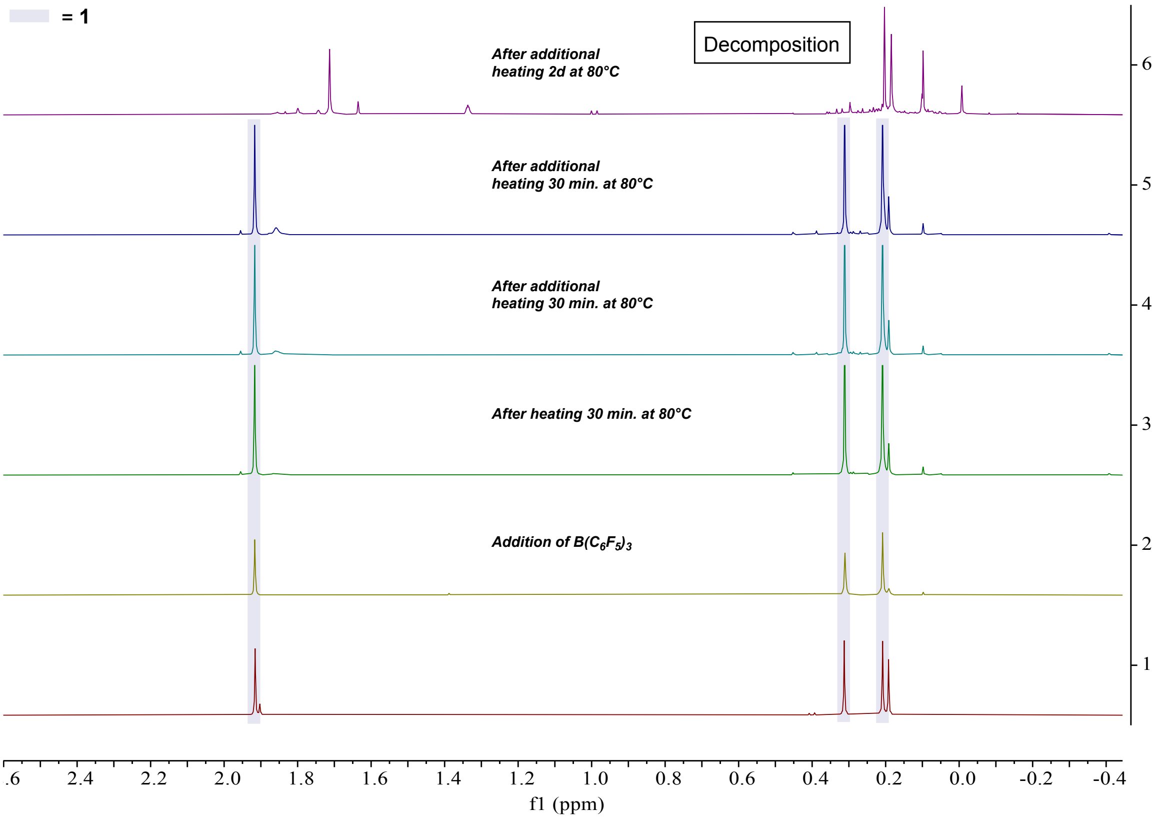

Figure S83: ^1H NMR (500 MHz, C_6D_6) screening for the decomposition of **1** in the presence of $B(C_6F_5)_3$ at irregular time intervals.

6 X-ray structure elucidation and refinement

X-ray Structure Determination:

X-ray quality crystals were selected in Fomblin YR-1800 perfluoroether (Alfa Aesar) at ambient temperature inside the glovebox. The samples were cooled to 100(2) K during measurement. A Stoe IPDS2 (STOE image plate detector system) with monochromated MoKα (λ = 0.71073 Å; fine focus) radiation or a Stoe StadiVari diffractometer with a DECTRIS PILATUS 200K detector and monochromated CuKα (λ = 1.54186 Å) radiation was employed a respective measurement. The structures were solved by intrinsic phasing (SHELXT)[8] and refined by full matrix least squares procedures (SHELXL)[9] within the Olex2 platform.[10]

Data reduction was performed using the *X-AREA* program. All absorption corrections have been done by Gaussian integration followed by a scaling of reflection intensities that was done within *STOE LANA*.[11]

All non-hydrogen atoms were refined anisotropically, hydrogen atoms were included in the refinement at calculated positions using a riding model. All special refinement details for disordered structures, molecular structure representations as well as further crystallographic details are summarized down below.

Special Refinement Details:

Compound 1: The molecular structure crystallizes with two independent molecules in the asymmetric unit. No further special refinement details need to be noted.

Compound 2: No special refinement details need to be noted.

Compound 4: No special refinement details need to be noted.

Compound ^{pre}***5:*** No special refinement details need to be noted.

Compound 5: The molecular structure crystallizes with two independent molecules in the asymmetric unit. The two independent molecules were generated through manually increasing the cell size by a factor of 2. During the cell-search we continuously find the following unit cell: a = 9.50, b = 13.62, c = 18.08, α = 98.67, β = 105.24, γ = 93.89, V = 2218.5 Å^3. The solution and refinement with these cell parameters require to describe one molecule in the asymmetric unit as an almost fully disordered molecule. The cell parameters of the doubled cell (see table below) seem crystallographically more viable than the above-mentioned.

Compound 6a: No special refinement details need to be noted.

Compound 6b: No special refinement details need to be noted.

Compound 7a: No special refinement details need to be noted.

Compound 7b·TMS: No special refinement details need to be noted.

Compound 9: No special refinement details need to be noted.

Molecular Structure Representations:

All molecular structure representations in the ESI as well as the main article have been prepared with the DIAMOND software package.[12] All ellipsoids are represented at the 50% probability level unless stated otherwise.

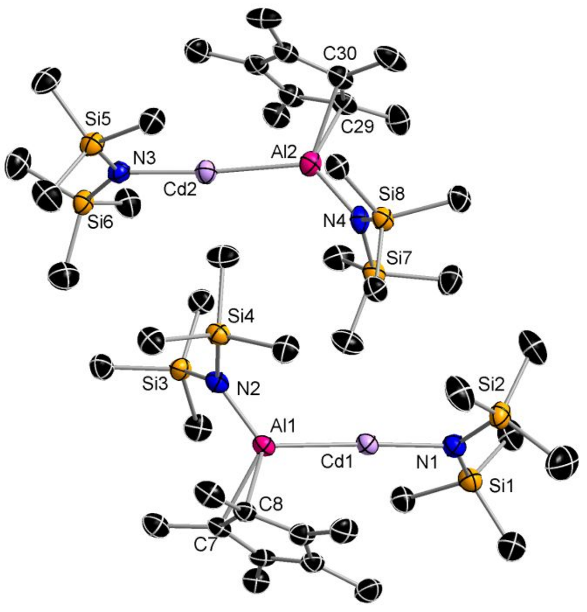

Figure S84: Full molecular structure representation of the two independent molecules of **1**. Selected atom distances [Å] and angles [°]: Al1-Cd1 2.528(2), Cd1-N1 2.094(6), Al1-N2 1.823(5), N1-Si1 1.706(6), N1-Si2 1.702(7), N2-Si3 1.728(6), N2-Si4 1.740(4), C7-Al1 2.147(8), C8-Al1 2.178(8), Al2-Cd2 2.518(2), Cd2-N3 2.077(6), Al2-N4 1.844(6), N3-Si5 1.699(5), N3-Si6 1.704(5), N4-Si7 1.728(4), N4-Si8 1.731(6), C29-Al2 2.152(10), C30-Al2 2.178(8) ; Al1-Cd1-N1 174.4(1), N2-Al1-Cd1 122.8(2), Al2-Cd2-N3 175.5(1), N4-Al2-Cd2 122.7(2).

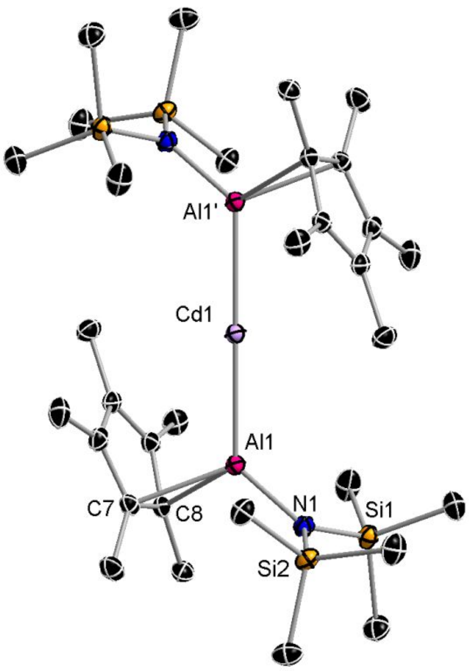

Figure S85: Enlarged molecular structure representations of **2** (50% probability level). Half a molecule is symmetry generated *via* 1-x, 1-y, 1-z. Selected atom distances [Å] and angles [°]: Al1–Cd1 2.579(1), Al1-N1 1.844(2), Al1-C7 2.210(3), Al1-C8 2.177(3); Al1-Cd1-Al1’ 180, N1-Al1-Cd1 127.50(7).

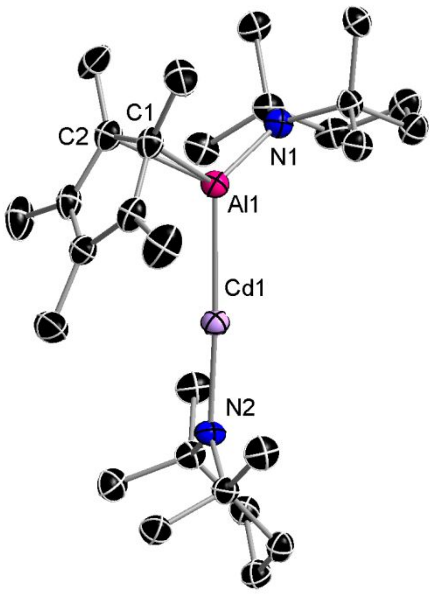

Figure S86: Enlarged molecular structure representations of **4**. Selected atom distances [Å] and angles [°]: Al1-Cd1 2.529(1), Al1-N1 1.824(3), Cd1-N2 2.067(2), C1-Al1 2.196(3), C2-Al1 2.174(3); Al1-Cd1-N2 173.72(8), N1-Al1-Cd1 123.8(1).

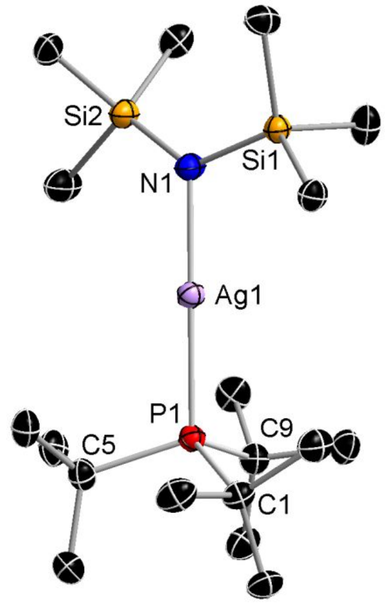

Figure S87: Enlarged molecular structure representations of ***pre*5**. Selected atom distances [Å] and angles [°]: Ag1-P1 2.352(1), Ag1-N1 2.086(3), P1-C1 1.894(5), P1-C5 1.901(4), P1-C9 1.894(4), N1-Si1 1.702(2), N1-Si2 1.689(4); P1-Ag1-N1 178.0(1), Si1-N1-Si2 126.1(1), C1-P1-Ag1 110.9(1) .C5-P1-Ag1 108.7(1), C9-P1-Ag1 107.9(1).

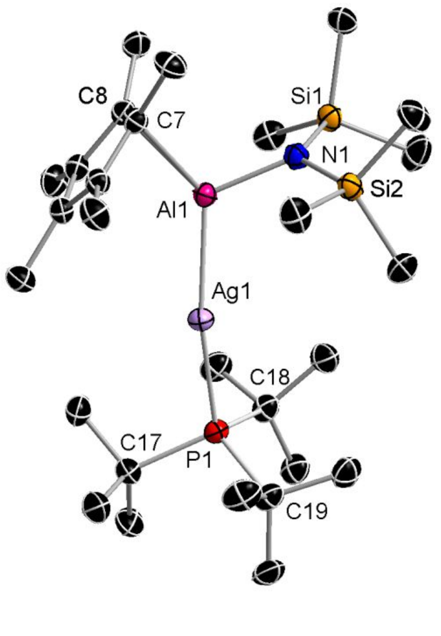

Figure S88: Enlarged molecular structure representations of **5**. Selected atom distances [Å] and angles [°]: Al1-Ag1 2.467(1), Ag1-P1 2.458(1), Al1-N1 1.872(3), Al1-C7 2.210(3), Al1-C8 2.226(3); Al1-Ag1-P1 166.10(3), N1-Al1-P1 114.01(8), C17-P1-Ag1 110.90(8), C18-P1-Ag1 105.04(8), C19-P1-Ag1 111.77(8).

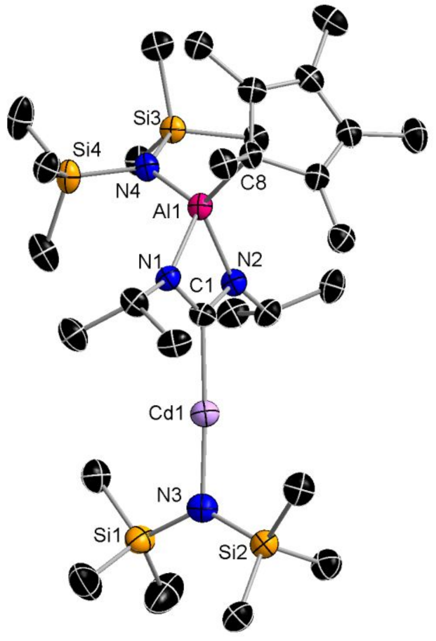

Figure S89: Enlarged molecular structure representations of **6a**. Selected atom distances [Å] and angles [°]: Al1-N1 1.928(3), Al1-N2 1.952(4), Al1-N4 1.822(5), Al1-C8 2.041(5), C1-Cd1 2.132(4), Cd1-N3 2.026(4); C1-Cd1-N3 2.132(4).

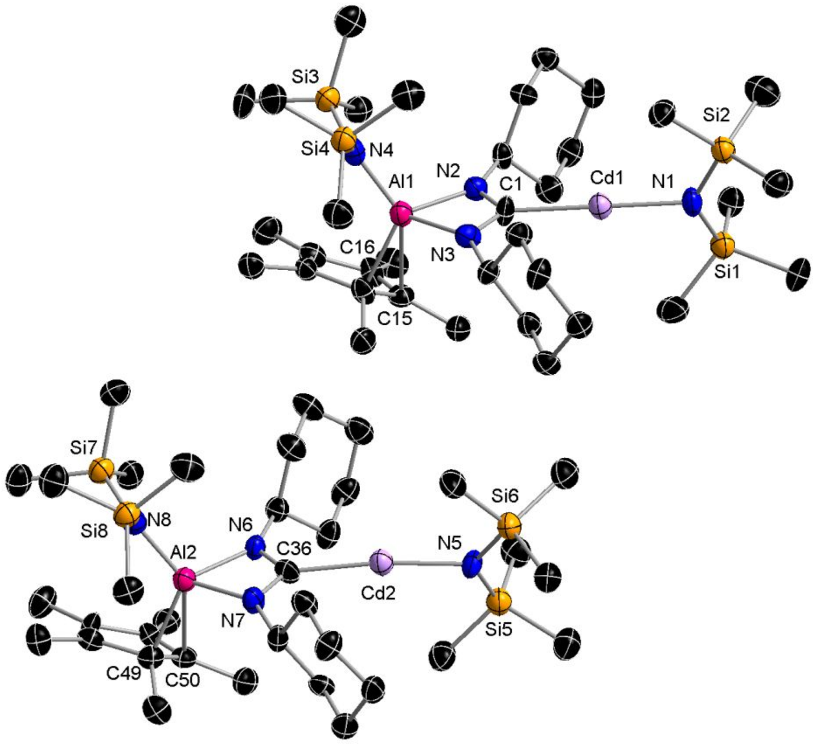

Figure S90: Full molecular structure representation of the two independent molecules of **6b**. Selected atom distances [Å] and angles [°]: Al1-N4 1.836(8), Al1-N2 1.933(6), Al1-N3 1.946(7), Cd1-C1 2.134(8), Cd1-N1 2.058(6), C14-Al1 2.054(7), C15-Al1 2.638(8); Al2-N8 1.842(8), Al2-N6 1.931(5), Al2-N7 1.937(7), Cd2-C36 2.137(8), Cd2-N5 2.043(7), C49-Al2 2.048(7), C50-Al2 2.652(9); C1-Cd1-N1 176.0(2), C36-Cd2-N5 172.3(3).

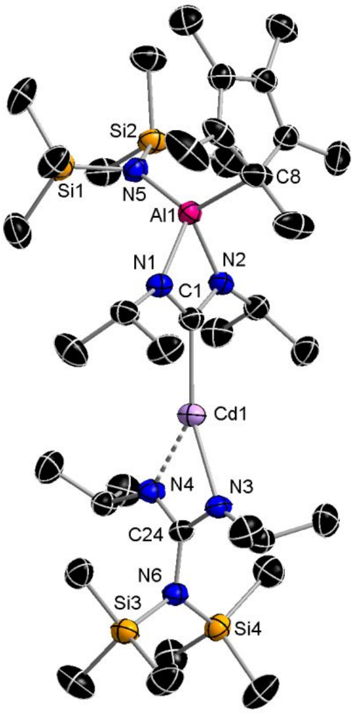

Figure S91: Enlarged molecular structure representations of **7a**. Selected atom distances [Å] and angles [°]: Al1-N5 1.8332(3), Al1-N1 1.932(3), Al1-N2 1.915(3), Al1-C8 2.049(4), Cd1-C1 2.135(3), Cd1-N3 2.181(3), Cd1-N4 2.202(3), N6-C24 1.421(4); C1-Cd1-N3 156.1(1), C1-Cd1-N4 143.0(1), N1-C1-N2 110.2(3), N3-C24-N4 114.0(3).

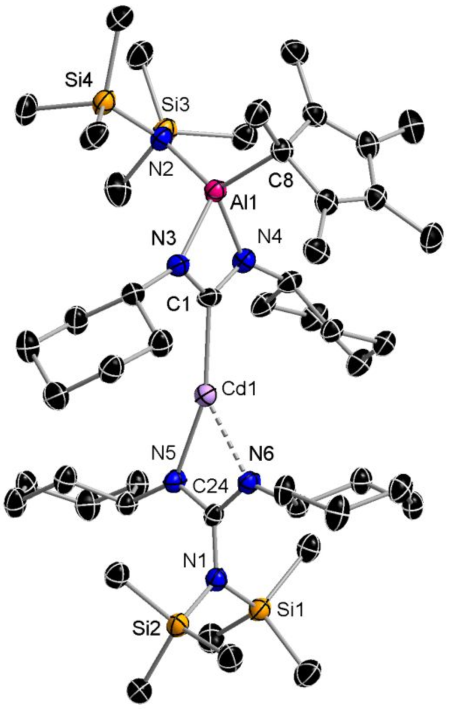

Figure S92: Enlarged molecular structure representations of **7b** (30% probability level). Selected atom distances [Å] and angles [°]: Al1-N2 1.854(4), Al1-N3 1.934(5), Al1-N4 1.919(5), Al1-C8 2.047(7), Cd1-C1 2.141(6), Cd1-N5 2.175(5), Cd1-N6 2.234(4), N1-C24 1.416(8); C1-Cd1-N5 157.0(2), C1-Cd1-N6 142.0(2), N3-C1-N4 110.5(4), N5-C24-N6 113.8(5).

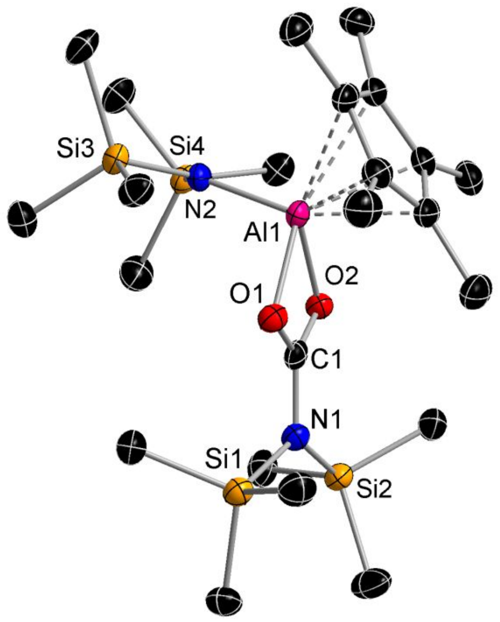

Figure S93: Enlarged molecular structure representations of **9**. Selected atom distances [Å] and angles [°]: Al1-N2 1.833(2), Al1-O1 1.923(1), Al1-O2 1.932(1), C1-O1 1.286(3), C1-O2 1.286(3), C1-N1 1.351(3), Si1-N1 1.795(2), Si2-N1 1.797(1), Si3-N2 1.726(2), Si4-N2 1.730(2); O1-C1-O2 114.4(2), N1-C1-O1 122.4(2), N1-C1-O2 123.0(2), Si1-N1-Si2 128.2(1), Si3-N2-Si4 118.5(1).

Summary of X-ray Crystallographic Refinement:

Table S1: Crystallographic details.

Compound	***1***	***2***	***4***
Empirical formula	$C_{22}H_{51}AlCdN_2Si_4$	$C_{32}H_{66}Al_2CdN_2Si_4$	$C_{28}H_{51}AlCdN_2$
Formula weight	595.38	757.58	555.08
Temperature/K	100	100	100
Crystal system	triclinic	triclinic	monoclinic
Space group	*P*–1	*P*–1	$P2_1/n$
a/Å	12.6573(9)	8.5666(8)	8.3544(5)
b/Å	16.5583(10)	9.7600(9)	15.5287(9)
c/Å	17.7068(10)	14.2668(13)	22.588(3)
α/°	110.156(5)	71.390(7)	90
β/°	98.149(5)	78.793(7)	99.207(8)
γ/°	107.377(5)	65.457(7)	90
Volume/Å^3	3198.4(4)	1025.69(18)	2892.7(4)
Z	4	1	4
ρ_{calc}g/cm^3	1.236	1.226	1.275
μ/mm^{-1}	0.872	0.714	0.802
F(000)	1256.0	402.0	1176.0
Crystal size/mm^3	0.08 × 0.07 × 0.02	0.13 × 0.083 × 0.04	0.26 × 0.137 × 0.06
Radiation	MoKα (λ = 0.71073)	MoKα (λ = 0.71073)	Mo Kα (λ = 0.71073)
2*Θ* range for data collection/°	2.546 to 52	3.02 to 54.018	3.196 to 54.108
Index ranges	-15 ≤ h ≤ 15, -20 ≤ k ≤ 20, -21 ≤ l ≤ 21	-10 ≤ h ≤ 10, -10 ≤ k ≤ 12, -18 ≤ l ≤ 18	-10 ≤ h ≤ 10, -19 ≤ k ≤ 19, -28 ≤ l ≤ 28
Reflections collected	30998	8105	18345
Independent reflections	12586 [R_{int} = 0.0582, R_{sigma} = 0.0663]	4385 [R_{int} = 0.0408, R_{sigma} = 0.0404]	6247 [R_{int} = 0.0424, R_{sigma} = 0.0364]
Data/restraints/parameters	12586/0/575	4385/0/198	6247/0/302
Goodness-of-fit on F^2	1.048	1.082	1.027
Final R indexes [I>=2σ (I)]	R_1 = 0.0722, wR_2 = 0.1574	R_1 = 0.0342, wR_2 = 0.0863	R_1 = 0.0440, wR_2 = 0.1084
Final R indexes [all data]	R_1 = 0.1164, wR_2 = 0.1786	R_1 = 0.0437, wR_2 = 0.0959	R_1 = 0.0560, wR_2 = 0.1169
Largest diff. peak/hole / e Å^{-3}	4.63/-1.19	1.00/-0.67	1.09/-1.15
CCDC #	2475328	2475319	2475327

Table S2: Crystallographic details (continuation).

Compound	***pre*5**	**5**	***6a***
Empirical formula	$C_{18}H_{45}AgNPSi_2$	$C_{28}H_{60}AlAgNPSi_2$	$C_{29}H_{65}AlCdN_4Si_4$
Formula weight	470.57	632.77	721.59
Temperature/K	100	100	100
Crystal system	monoclinic	monoclinic	triclinic
Space group	$P2_1/c$	$P2_1$/n	P–1
a/Å	12.4240(6)	13.3116(5)	9.3153(8)
b/Å	13.5326(4)	14.6822(7)	14.2766(12)
c/Å	15.8087(8)	17.8137(7)	16.7980(13)
α/°	90	90	65.411(6)
β/°	112.752(4)	91.328(3)	87.440(6)
γ/°	90	90	77.880(7)
Volume/Å^3	2451.1(2)	3480.6(3)	1983.8(3)
Z	4	4	2
ρ_{calc}g/cm^3	1.275	1.208	1.208
μ/mm^{-1}	0.986	6.087	0.716
F(000)	1000.0	1352.0	768.0
Crystal size/mm^3	0.11 × 0.08 × 0.04	0.16 × 0.097 × 0.04	0.23 × 0.16 × 0.06
Radiation	Mo Kα (λ = 0.71073)	Cu Kα (λ = 1.54186)	Mo Kα (λ = 0.71073)
2Θ range for data collection/°	3.554 to 53.822	7.804 to 144.17	2.67 to 52
Index ranges	-15 ≤ h ≤ 15, -17 ≤ k ≤ 17, -20 ≤ l ≤ 18	-16 ≤ h ≤ 16, -17 ≤ k ≤ 15, -11 ≤ l ≤ 21	-11 ≤ h ≤ 10, -17 ≤ k ≤ 17, -20 ≤ l ≤ 20
Reflections collected	13039	37308	14293
Independent reflections	5271 [R_{int} = 0.0352, R_{sigma} = 0.0411]	6773 [R_{int} = 0.0252, R_{sigma} = 0.0171]	7741 [R_{int} = 0.0697, R_{sigma} = 0.0702]
Data/restraints/parameters	5271/0/223	6773/0/327	7741/0/373
Goodness-of-fit on F^2	1.073	1.012	1.103
Final R indexes [I>=2σ (I)]	R_1 = 0.0400, wR_2 = 0.0905	R_1 = 0.0357, wR_2 = 0.0919	R_1 = 0.0630, wR_2 = 0.1703
Final R indexes [all data]	R_1 = 0.0595, wR_2 = 0.0988	R_1 = 0.0411, wR_2 = 0.0963	R_1 = 0.0812, wR_2 = 0.1830
Largest diff. peak/hole / e Å^{-3}	0.73/-1.07	1.31/-0.73	1.39/-1.87
CCDC #	2475321	2475325	2475322

Table S3: Crystallographic details (continuation).

Compound	***6b***	***7a***	***7b·TMS***
Empirical formula	$C_{35}H_{73}AlCdN_4Si_4$	$C_{36}H_{79}AlCdN_6Si_4$	$C_{52}H_{107}AlCdN_6Si_5$
Formula weight	801.71	847.79	1096.26
Temperature/K	100	100	100
Crystal system	triclinic	monoclinic	triclinic
Space group	*P*–1	*P*2$_1$/*c*	*P*–1
a/Å	16.0325(11)	15.160(3)	9.6689(6)
b/Å	17.0685(11)	30.962(4)	17.6360(9)
c/Å	18.0574(11)	10.1801(10)	21.3332(12)
α/°	106.921(5)	90	111.828(4)
β/°	90.020(5)	93.742(11)	96.275(5)
γ/°	110.183(5)	90	104.134(4)
Volume/Å^3	4409.2(5)	4768.3(11)	3192.8(3)
Z	4	4	2
ρ_{calc}g/cm^3	1.208	1.181	1.140
μ/mm^{-1}	0.651	0.607	4.031
F(000)	1712.0	1816.0	1184.0
Crystal size/mm^3	0.17 × 0.117 × 0.02	0.17 × 0.107 × 0.07	0.22 × 0.1 × 0.04
Radiation	Mo Kα (λ = 0.71073)	Mo Kα (λ = 0.71073)	Cu Kα (λ = 1.54186)
2*Θ* range for data collection/°	2.372 to 50.998	2.63 to 51.998	9.588 to 135
Index ranges	-19 ≤ h ≤ 19, -20 ≤ k ≤ 20, -19 ≤ l ≤ 21	-18 ≤ h ≤ 18, -38 ≤ k ≤ 36, -10 ≤ l ≤ 12	-11 ≤ h ≤ 3, -19 ≤ k ≤ 21, -25 ≤ l ≤ 25
Reflections collected	31103	22557	32142
Independent reflections	16376 [R_{int} = 0.1051, R_{sigma} = 0.1481]	9339 [R_{int} = 0.0404, R_{sigma} = 0.0400]	11286 [R_{int} = 0.0568, R_{sigma} = 0.0579]
Data/restraints/parameters	16376/0/845	9339/0/458	11286/0/607
Goodness-of-fit on *F*^{2}	0.978	1.060	1.050
Final R indexes [I>=2σ (I)]	R_1 = 0.0811, wR_2 = 0.1855	R_1 = 0.0530, wR_2 = 0.1283	R_1 = 0.0680, wR_2 = 0.1762
Final R indexes [all data]	R_1 = 0.1717, wR_2 = 0.2200	R_1 = 0.0717, wR_2 = 0.1384	R_1 = 0.0998, wR_2 = 0.1950
Largest diff. peak/hole / e Å^{-3}	0.99/-1.73	0.61/-0.96	1.31/-0.56
CCDC #	2475326	2475322	2475324

Table S4: Crystallographic details (continuation).

Compound	***9***
Empirical formula	$C_{23}H_{51}AlN_2O_2Si_4$
Formula weight	526.99
Temperature/K	100
Crystal system	triclinic
Space group	*P*–1
a/Å	9.8025(6)
b/Å	10.8914(7)
c/Å	15.3501(10)
α/°	80.133(5)
β/°	86.115(5)
γ/°	72.311(5)
Volume/Å^3	1538.03(18)
Z	2
ρ_{calc}g/cm^3	1.138
μ/mm^{-1}	0.243
F(000)	576.0
Crystal size/mm^3	0.18 × 0.117 × 0.04
Radiation	Mo Kα (λ = 0.71073)
2*Θ* range for data collection/°	2.694 to 53.978
Index ranges	-12 ≤ h ≤ 11, -13 ≤ k ≤ 13, -19 ≤ l ≤ 19
Reflections collected	12301
Independent reflections	6596 [R_{int} = 0.0556, R_{sigma} = 0.0644]
Data/restraints/parameters	6596/0/306
Goodness-of-fit on F^2	1.044
Final R indexes [I>=2σ (I)]	R_1 = 0.0557, wR_2 = 0.1411
Final R indexes [all data]	R_1 = 0.0812, wR_2 = 0.1565
Largest diff. peak/hole / e Å^{-3}	0.72/-0.48
CCDC #	2475320

7 Computational details

7.1 General computational details

The calculations were performed with and ORCA 5.0.4.[13,14] All structures were verified as true minima by the absence of imaginary eigenvalues in the harmonic vibrational frequency analysis except for transition states which were verified with a single imaginary eigenvalue. All geometry optimizations were performed with the r^2SCAN-3c composite electronic-structure method (considering relativistic effects using ECPs) which afforded an excellent fit for the structural parameters found in the solid state (section 7.2).[15] Tighter than default convergence criteria (*tightopt*) were chosen for both, the optimization of the structural parameters as well as the respective single-point calculations (*tightscf*). Transition states were either located using scans across the reaction coordinate, constraints or the Nudged Elastic Band (=NEB) method.[16]

The single point calculations were done according to the denotation ZORA-DLPNO-CCSD(T) CPCM(C_6H_6)/ZORA def2-TZVPP[17] {SARC-ZORA-TZVPP(Cadmium)}[18] // r^2SCAN-3c level of theory. Automatic generation of an auxiliary basis set was applied (*autoaux*).[19] Input line: ! DLPNO-CCSD(T) ZORA CPCM(C_6H_6) ZORA-DEF2-TZVPP tightscf AUTOAUX; with cadmium present, " newGTO Cd "SARC-ZORA-TZVPP" " was introduced. Implicit correction for solvation effects was conducted for all single point calculations with the CPCM (= conductor-like polarizable continuum) solvation model.[20] Correction for solvation in benzene gives consistent results.

Representation of calculated structures, IBOs as well as the canonical orbitals was done with the ChemCraft and IBOview software packages.[21–23] All canonical orbitals were plotted according to a single point calculation at the PBE0-D4 CPCM(C_6H_6)/def2-TZVPP//r^2SCAN-3c level of theory.[24,25] Time dependent DFT spectra have been obtained at the B3LYP-D3 or PBE0-D3 CPCM(C_6H_6)/def2-TZVPP//r^2SCAN-3c level of theory.[24,26,27]

7.2 Benchmarking

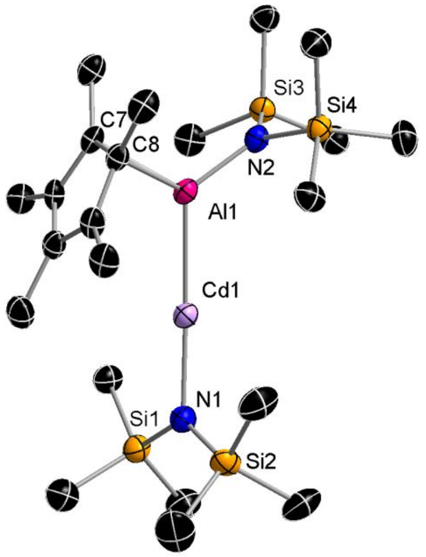

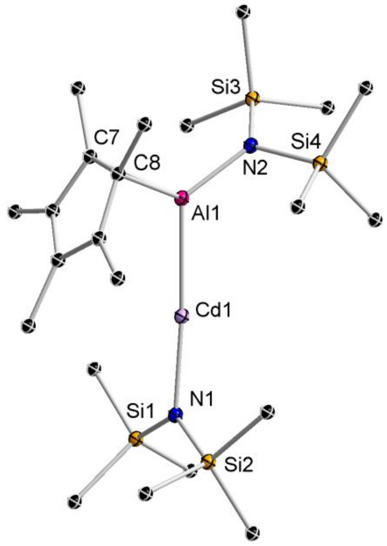

Figure S94: Comparison of structural parameters of **1** as obtained by SC-XRD at 100 K (left) with the calculated structure at the r^{2}SCAN-3c level of theory (right). All H atoms are omitted for clarity. Ellipsoids of the X-ray determined structure are set to a probability level of 50%.

Table S5: Selected atom distances and angles of **1** as obtained by SC-XRD (100 K) and as calculated with the r^{2}SCAN-3c composite method.

**Bond**	**Atom distances [Å] and angles [°]**	
	X-ray	***DFT (r^{2}SCAN-3c)***
Al1–Cd1	2.528(2)	2.5291
N1–Cd1	2.094(6)	2.0937
C7-Al1	2.147(8)	2.1355
C8-Al1	2.178(8)	2.1760
N2-Al1	1.824(6)	1.8305
N1-Si1	1.706(6)	1.7196
N2-Si1	1.702(7)	1.7194
N2-Si3	1.728(6)	1.7420
N2-Si4	1.740(4)	1.7459
Al1-Cd1-N1	174.4(1)	173.26
N2-Al1-Cd1	122.8(2)	123.96
Si1-N1-Si2	128.7(3)	128.14
Si3-N2-Si4	123.9(3)	122.33

7.3 Representation of selected IBOs

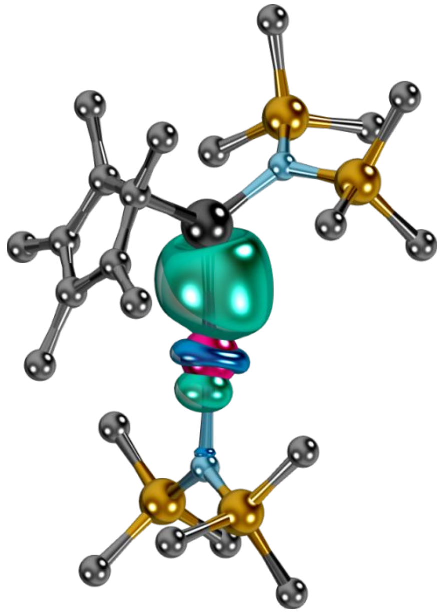

Compound 1: $q_{\sigma\text{-IBO}}$(Al, Cd) = 0.95, 0.94 = IAO partial charge distributions
Net IAO partial charge at Al: +0.37357
Net IAO partial charge at Cd: +1.00533

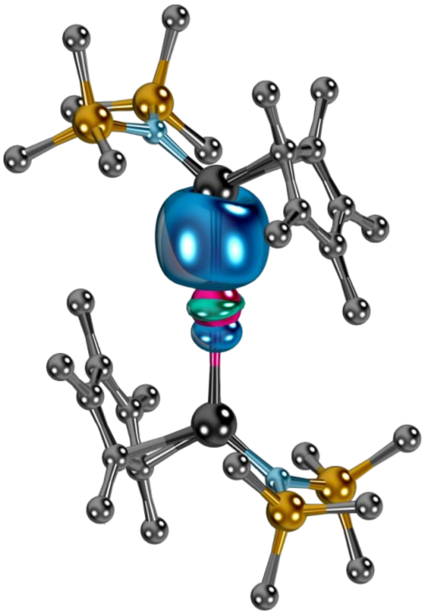

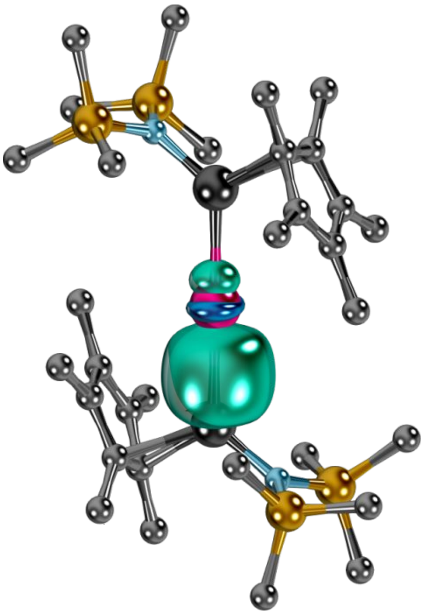

Compound 2: $q_{\sigma\text{-IBO}}$(Al, Cd) = 1.18, 0.69 = IAO partial charge distributions
$q_{\sigma\text{-IBO}}$(Al, Cd) = 1.18, 0.68 = IAO partial charge distributions
Net IAO partial charge at Al: +0.25824, +0.26428
Net IAO partial charge at Cd: +0.89069

Figure S95: Calculated IBOs (r²SCAN-3c) of **1a** (top) and **1b** (middle) and **4** (right).

7.4 Representation of selected frontier orbitals

7.4.1 [({N(TMS)$_2$})(Cp*)Al–Cd({N(TMS)$_2$})] (**1**)

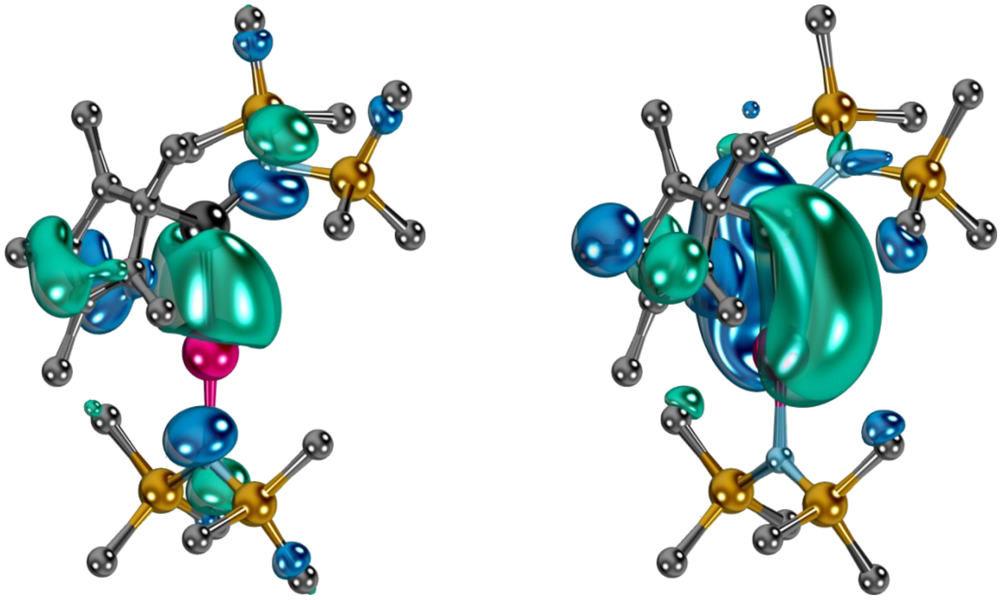

HOMO {—6.0491 eV} **LUMO** {—0.6422 eV}

Figure S96: Calculated frontier orbitals of **1**. Values denoted with { } are derived by single point calculation according to the PBE0-D4 CPCM(C_6H_6)/def2-TZVPP//r^{2}SCAN-3c level of theory.

7.4.2 [{N(TMS)$_2$}(Cp*)Al]$_2$Cd (**2**)

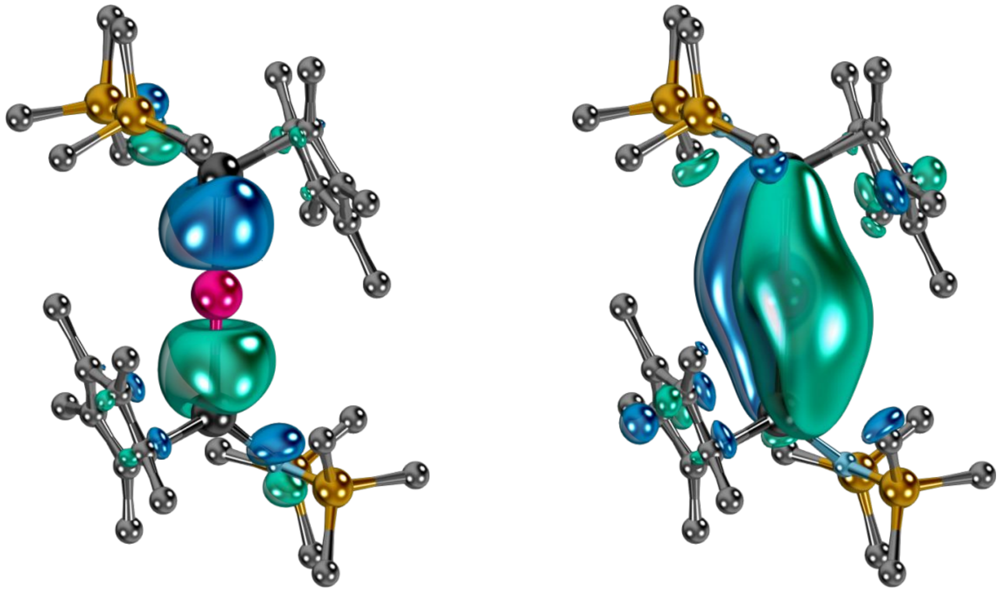

HOMO {–-4.9198 eV} **LUMO** {–-0.0014 eV}

Figure S97: Calculated frontier orbitals of **2**. Values denoted with { } are derived by single point calculation according to the PBE0-D4 CPCM(C_6H_6)/def2-TZVPP//r^{2}SCAN-3c level of theory.

7.4.3 [{N(TMS)$_2$}(Cp*)Al]$_2$Cd (**2**:*conformer*)

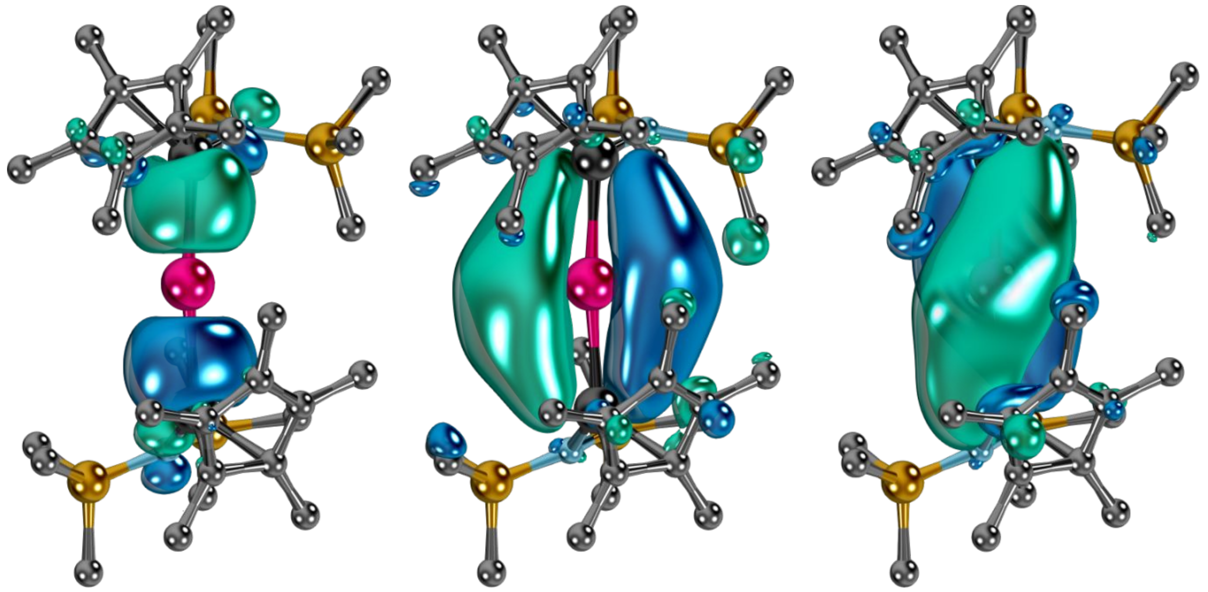

HOMO {–-4.8708 eV} **LUMO** {–-0.9415 eV} **LUMO+1** {–-0.5823 eV}

Figure S98: Calculated frontier orbitals of **2**. Values denoted with { } are derived by single point calculation according to the PBE0-D4 CPCM(C_6H_6)/def2-TZVPP//r^{2}SCAN-3c level of theory.

7.5 Representation of simulated absorption spectra

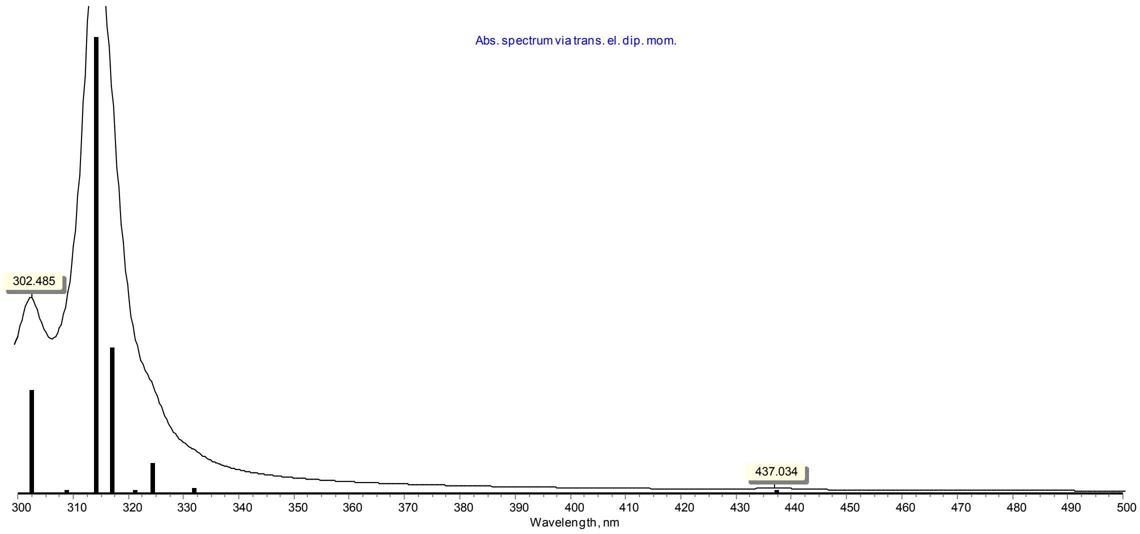

Figure S99: TD-DFT derived absorption of **2** at the B3LYP-D3 CPCM(C_6H_6)/def2-tzvpp//r^{2}SCAN-3c level of theory (visualization through *ChemCraft*).

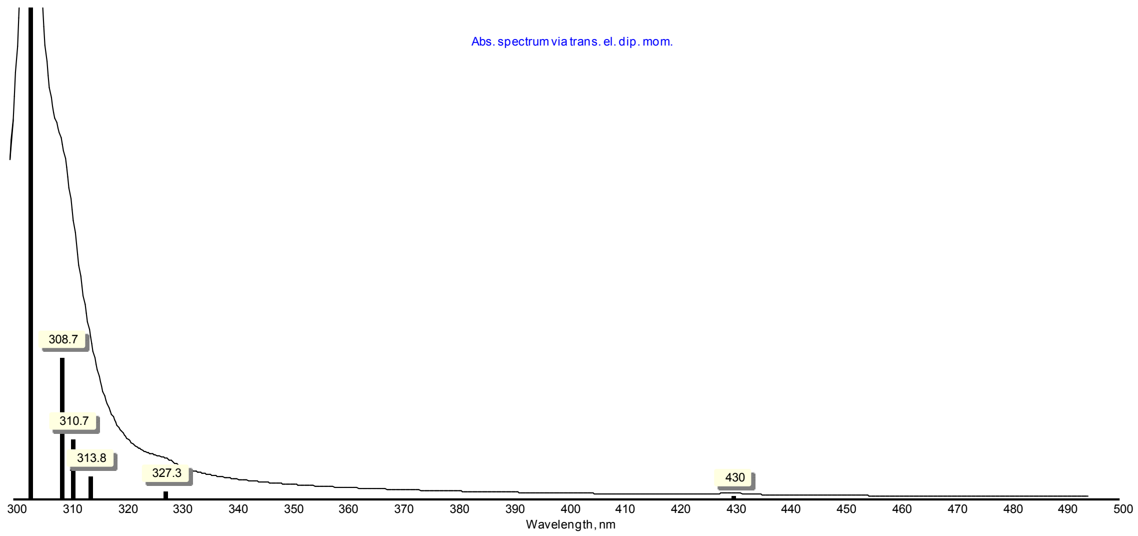

Figure S100: TD-DFT derived absorption of **2** at the Pbe0-D3 CPCM(C_6H_6)/def2-tzvpp//r^{2}SCAN-3c level of theory (visualization through *ChemCraft*).

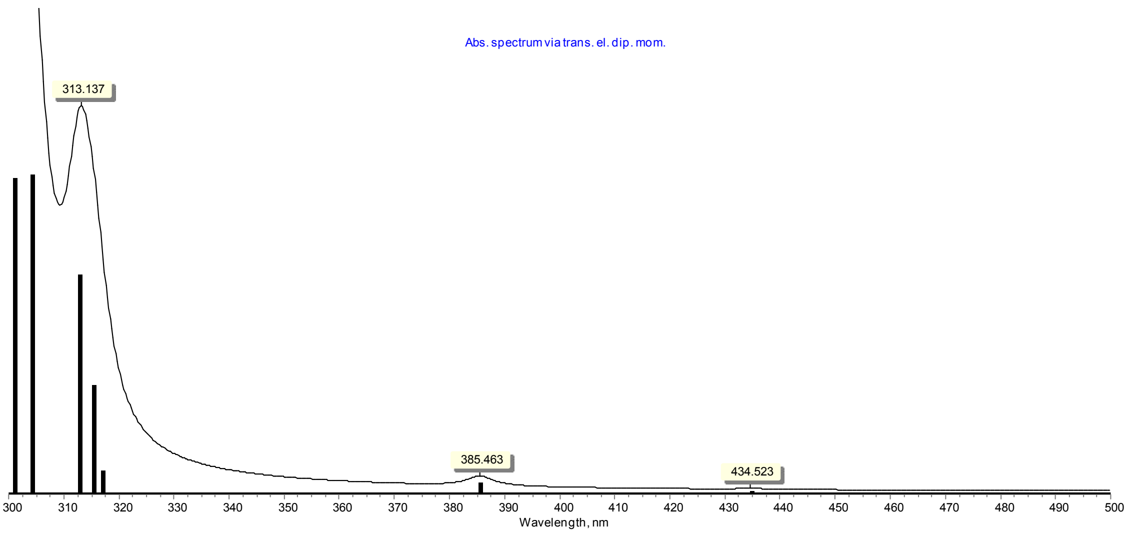

Figure S101: TD-DFT derived absorption spectrum of **2:conformer** at the B3LYP-D3 CPCM(C_6H_6)/def2-tzvpp//r^{2}SCAN-3c level of theory (visualization through *ChemCraft*).

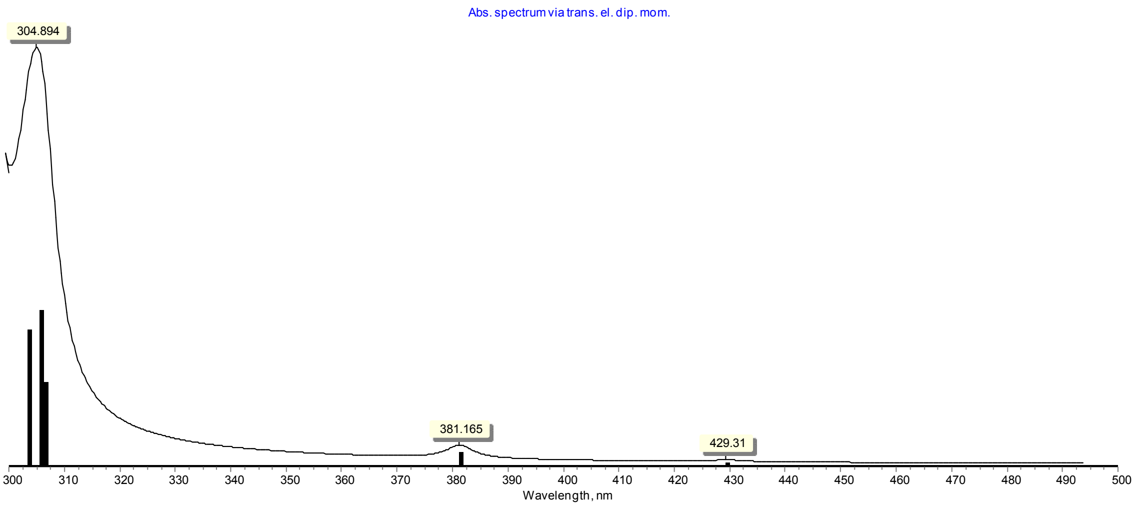

Figure S102: TD-DFT derived absorption spectrum of **2:conformer** at the Pbe0-D3 CPCM(C_6H_6)/def2-tzvpp//r^{2}SCAN-3c level of theory (visualization through *ChemCraft*).

7.6 Energies of optimized structures

Table S6: Energies of the reactants.

Compound	*E* [Eh][a]	*G* [Eh][a]	*E*(SP) [Eh][b]
**Cd{N(TMS)$_2$}$_2$**	-1914.29005	-1913.89856	-7410.92566; -7410.92742[c]
**AlCp***	-632.40698	-632.22574	-632.34878
**DIC**	-384.55032	-384.38456	-384.26069

[a] r^{2}SCAN-3c
[b] ZORA-DLPNO-CCSD(T) CPCM(C_6H_6)/ZORA def2-TZVPP//r^{2}SCAN-3c
[c] ZORA-DLPNO-CCSD(T) SMD(C_6H_6)/ZORA def2-TZVPP{SARC-ZORA-TZVPP(Cd)}//r^{2}SCAN-3c

Table S7: Energies of ground- and transition states.

Compound	*E* [Eh][a]	*G* [Eh][a]	*E*(SP) [Eh][b]
**IM1**	-2546.72094	-2546.12198	-8043.29521
**TS1** (TS$^{IM1\text{->}1}$)	-2546.71296	-2546.11391	-8043.28500
**1**	-2546.76881	-2546.16936	-8043.33690; -8043.34467[c]
**IM2**	-3179.19482	-3178.38871	-8675.70025
**TS2** (TS$^{IM2\text{->}2}$)	-3179.19162	-3178.38399	-8675.69496
**2**	-3179.22798	-3178.42222	-8675.73075; -8675.74441[c]
**IM1'**	-2931.33958	-2930.54122	-8427.62082
**TS1'** (TS$^{IM1'\text{->}IM2'}$)	-2931.32108	-2930.52237	-8427.59348
**IM2'**	-2931.33979	-2930.54344	-8427.61503
**TS2'A** (TS$^{IM2'\text{->}6a}$)	-2931.33230	-2930.53511	-8427.61059
**6a**	-2931.37811	-2930.58231	-8427.66185
**TS2'B** (TS$^{IM2'\text{->}IM3}$)	-2931.31207	-2930.51494	-8427.59154
**IM3**	-2931.34678	-2930.55425	-8427.62872
**TS3** (TS$^{IM3\text{->}6a}$)	-2931.29615	-2930.49865	-8427.57889
**7a**	-3315.97271	-3314.98072	-8811.96517

[a] r^{2}SCAN-3c
[b] ZORA-DLPNO-CCSD(T) CPCM(C_6H_6)/ZORA def2-TZVPP{SARC-ZORA-TZVPP(Cd)}//r^{2}SCAN-3c
[c] ZORA-DLPNO-CCSD(T) SMD(C_6H_6)/ZORA def2-TZVPP{SARC-ZORA-TZVPP(Cd)}//r^{2}SCAN-3c

Table S8: Energies of ground- and transition states of further species.

Compound	*E* [Eh][a]	*G* [Eh][a]	*E*(SP) [Eh][b]
**B(C$_6$F$_5$)$_3$**	-2208.21750	-2208.12018	-2208.02353
**Zn{N(TMS)$_2$}$_2$**	-3525.87437	-3525.48157	-3552.56870
**Cd(TMP)$_2$**	-984.23512	-984.69803	-6478.90799
***pre*5**	-1835.03278	-1834.49907	-7067.36974
**1^{Zn}**	-4158.33347	-4157.73241	-4184.96513
**3**	-2840.67225	-2840.36743	-2840.42930
**4**	-1617.16736	-1616.49788	-7111.31590
**5**	-2467.50130	-2466.76068	-7699.77632

[a] r^{2}SCAN-3c
[b] ZORA-DLPNO-CCSD(T) SMD(C_6H_6)/ZORA def2-TZVPP{SARC-ZORA-TZVPP(Cd)}//r^{2}SCAN-3c

7.7 XYZ coordinates

26
AlCp*

Al	-0.795474000	7.552988000	3.767259000
C	-1.464937000	9.672604000	4.446597000
C	-1.467683000	9.649198000	3.021232000
C	-0.123502000	9.476876000	4.887769000
C	-2.651210000	9.924506000	5.324546000
C	-0.127942000	9.438664000	2.581519000
C	-2.657717000	9.871758000	2.140359000
C	0.702829000	9.332118000	3.735076000
C	0.345176000	9.487460000	6.309650000
H	-2.763652000	10.996931000	5.529401000
H	-2.558264000	9.414705000	6.287690000
H	-3.578690000	9.580912000	4.858108000
C	0.334359000	9.403765000	1.157960000
H	-2.781551000	10.938878000	1.915266000
H	-2.559991000	9.344841000	1.187047000
H	-3.581282000	9.527406000	2.614069000
C	2.190352000	9.162811000	3.735892000
H	-0.431085000	9.134060000	6.994038000
H	0.620590000	10.503455000	6.620591000
H	1.224033000	8.852091000	6.450551000
H	-0.440194000	9.015745000	0.490520000
H	0.594917000	10.411276000	0.808948000
H	1.220634000	8.774954000	1.035536000
H	2.693491000	10.138194000	3.719675000
H	2.535955000	8.630473000	4.626432000
H	2.534009000	8.601998000	2.862191000

55
Cd{N(TMS)$_2$}$_2$

Cd	-1.675833000	4.901233000	3.505526000
N	0.044760000	3.827220000	3.456169000
N	-3.394728000	5.977786000	3.562335000
Si	0.177243000	2.686074000	2.163746000
Si	1.181734000	4.178350000	4.711078000
Si	-3.376564000	7.455511000	2.664853000
Si	-4.659707000	5.316463000	4.537979000
C	1.264758000	3.326960000	0.765760000
C	-1.550536000	2.386322000	1.458605000
C	0.879413000	1.043729000	2.761666000
C	2.934848000	4.305808000	4.033863000
C	0.714139000	5.835758000	5.490405000
C	1.155900000	2.875263000	6.070587000
C	-4.992829000	7.716794000	1.734184000
C	-1.960892000	7.370821000	1.415309000
C	-3.071074000	8.950370000	3.769811000
C	-6.012668000	4.521961000	3.496136000
C	-3.918032000	3.971095000	5.639436000
C	-5.445824000	6.631239000	5.634574000
H	0.881351000	4.277630000	0.376627000
H	1.306834000	2.614947000	-0.067151000
H	2.290944000	3.501520000	1.107473000
H	-2.243088000	2.015381000	2.224867000
H	-1.521923000	1.638744000	0.656967000
H	-1.982012000	3.297446000	1.020793000
H	0.252672000	0.608193000	3.547777000
H	0.938873000	0.323647000	1.937164000
H	3.003437000	5.082363000	3.263942000
H	3.644956000	4.554167000	4.831287000
H	3.264688000	3.361823000	3.584976000
H	-0.276577000	5.807143000	5.965329000
H	1.428110000	6.114068000	6.274675000
H	0.712744000	6.645408000	4.749602000
H	0.151854000	2.781086000	6.500920000
H	1.847649000	3.129750000	6.882469000
H	-5.195343000	6.884735000	1.050733000
H	-4.955538000	8.639739000	1.143658000
H	-0.979994000	7.299885000	1.906431000
H	-1.932855000	8.273666000	0.793613000
H	-2.065829000	6.512826000	0.739245000
H	-3.868027000	9.070428000	4.511903000
H	-3.016621000	9.876398000	3.185033000
H	-2.126668000	8.842108000	4.316355000
H	-5.599514000	3.735118000	2.854176000
H	-6.787763000	4.069905000	4.126495000
H	-3.115785000	4.361939000	6.278517000
H	-4.680871000	3.544797000	6.301861000
H	-3.510792000	3.135139000	5.053592000
H	-5.902618000	7.430483000	5.039650000
H	-6.235130000	6.197944000	6.259909000
H	-4.702829000	7.091682000	6.295299000
H	-6.499208000	5.256739000	2.845347000
H	-5.844671000	7.801976000	2.418626000
H	1.442117000	1.890747000	5.684636000
H	1.890979000	1.159857000	3.167472000

23
DIC

C	-1.525557000	3.316489000	4.502122000
N	-0.978237000	2.634010000	3.648529000
N	-2.101064000	3.843646000	5.443344000
C	-1.202889000	2.736562000	2.196678000
C	-1.661496000	5.076650000	6.120415000
C	-0.809152000	5.969848000	5.224425000
H	0.117164000	5.456403000	4.940746000
H	-0.540632000	6.892797000	5.748818000
H	-1.349892000	6.239696000	4.311265000
C	-0.930045000	4.698453000	7.407285000
H	-2.581585000	5.612953000	6.389551000
H	-1.549132000	4.044200000	8.027064000
H	-0.684884000	5.597767000	7.982265000
H	0.001320000	4.171044000	7.172999000
C	0.047790000	3.324021000	1.546210000
H	0.191442000	4.360868000	1.870929000
H	-0.049859000	3.313026000	0.455513000
H	0.935917000	2.750277000	1.824830000
C	-2.456028000	3.530014000	1.843680000
H	-1.323109000	1.703060000	1.841889000
H	-3.340259000	3.107791000	2.331417000
H	-2.620522000	3.520915000	0.761247000
H	-2.353142000	4.573841000	2.163648000

81
IM1

Cd	-1.784053000	5.301952000	3.311064000
Al	-0.722757000	7.808550000	3.392109000
N	-0.175806000	3.886975000	3.204421000
N	-3.861583000	5.151704000	3.701314000
C	-1.033261000	9.845076000	4.277586000
C	-0.590483000	9.977559000	2.923103000
Si	0.851036000	4.049927000	1.846416000
Si	0.142880000	3.293925000	4.782122000
Si	-4.698892000	3.943147000	2.802911000
Si	-4.551789000	6.349278000	4.712580000
C	-0.005416000	9.177463000	5.014309000
C	-2.309882000	10.375267000	4.850848000
C	0.721310000	9.403539000	2.827433000
C	-1.331270000	10.660768000	1.815782000
C	2.223025000	5.332888000	2.115690000
C	-0.141956000	4.657333000	0.355596000
C	0.526253000	4.728421000	5.968479000
C	-1.387153000	2.426571000	5.461667000
C	-3.518636000	3.157814000	1.557988000
C	-6.138232000	4.666581000	1.811459000
C	-3.364404000	6.773153000	6.126893000
C	-4.908562000	7.948339000	3.763779000

C	1.081979000	8.913599000	4.122527000
C	-0.015368000	8.885114000	6.482195000
H	-2.116447000	11.304924000	5.400020000
H	-2.772473000	9.668788000	5.545705000
H	-3.041702000	10.596866000	4.071230000
C	1.589253000	9.396860000	1.607537000
H	-1.127408000	11.738480000	1.826576000
H	-1.031810000	10.278813000	0.836490000
H	-2.412623000	10.527705000	1.907612000
H	1.767395000	6.315241000	2.298676000
H	2.883408000	5.425689000	1.244819000
H	2.841113000	5.086243000	2.987331000
H	-0.889467000	3.916462000	0.051420000
H	0.518150000	4.837191000	-0.501671000
H	-0.668833000	5.601194000	0.556689000
H	-0.331595000	5.411852000	6.044685000
H	0.758991000	4.384724000	6.983680000
H	1.382397000	5.306817000	5.598328000
H	-1.651928000	1.562609000	4.840908000
H	-1.231097000	2.072812000	6.487753000
H	-2.255176000	3.099374000	5.470105000
H	-3.192354000	3.891599000	0.809822000
H	-4.014668000	2.343510000	1.016175000
H	-2.622637000	2.741950000	2.036834000
H	-6.897926000	5.124375000	2.454821000
H	-6.636226000	3.889667000	1.218597000
H	-5.780822000	5.438897000	1.120071000
H	-2.408554000	7.164691000	5.753683000
H	-3.791656000	7.526428000	6.800669000
H	-3.140813000	5.879700000	6.722388000
H	-5.647802000	7.762804000	2.975640000
H	-5.294465000	8.746412000	4.409763000
H	-3.997824000	8.315789000	3.272335000
C	2.397282000	8.323552000	4.521108000
H	-1.030779000	8.887626000	6.885426000
H	0.560840000	9.645602000	7.023739000
H	0.429491000	7.910868000	6.707479000
H	1.007192000	9.251798000	0.693187000
H	2.119862000	10.352220000	1.512756000
H	2.339332000	8.603598000	1.652345000
H	3.063660000	9.112567000	4.891745000
H	2.281103000	7.588347000	5.321980000
H	2.897945000	7.829003000	3.685991000
C	-6.161520000	5.796146000	5.528769000
H	-6.934423000	5.531437000	4.799563000
H	-6.563139000	6.601687000	6.155531000
H	-5.994896000	4.924092000	6.170814000
C	-5.381607000	2.564715000	3.895766000
H	-5.846347000	1.778351000	3.288571000
H	-6.139502000	2.936644000	4.593636000
H	-4.580807000	2.105171000	4.486581000
C	1.676367000	2.432632000	1.336138000
H	2.184110000	2.553169000	0.371398000
H	0.934154000	1.633636000	1.228498000
H	2.422993000	2.100343000	2.064126000
C	1.601387000	2.100978000	4.857100000
H	2.536378000	2.577536000	4.540706000
H	1.436572000	1.226182000	4.219102000
H	1.742455000	1.744971000	5.884663000

81
TS1

Cd	-1.769319000	4.772790000	4.016670000
Al	-2.505854000	7.212368000	3.661594000
N	-0.159962000	3.439786000	3.789793000
N	-4.049675000	5.011891000	4.122316000
C	-3.404497000	9.153543000	4.212375000
C	-3.892088000	8.751425000	2.922910000
Si	1.106749000	4.156620000	2.867996000
Si	-0.341294000	1.824409000	4.354309000
Si	-4.772886000	4.249904000	2.749286000
Si	-4.717380000	4.985027000	5.715451000
C	-1.993744000	9.388376000	4.095670000
C	-4.231732000	9.473723000	5.420888000
C	-2.772975000	8.726861000	2.026888000
C	-5.322233000	8.486396000	2.571094000
C	1.782299000	5.695940000	3.731804000
C	0.466362000	4.720425000	1.178347000
C	2.563724000	2.995447000	2.571652000
C	1.147175000	1.219091000	5.345947000
C	-1.839723000	1.753339000	5.498831000
C	-0.618101000	0.592154000	2.949315000
C	-4.630125000	2.367261000	2.767156000
C	-3.864508000	4.808293000	1.186453000
C	-6.603072000	4.673299000	2.548592000
C	-5.343943000	3.303261000	6.304011000
C	-3.397551000	5.554915000	6.948396000
C	-6.186098000	6.161623000	5.893393000
C	-1.610820000	9.129648000	2.756009000
C	-1.102356000	9.837377000	5.210628000
H	-4.157884000	10.544037000	5.647429000
H	-3.913361000	8.926707000	6.314393000
H	-5.286001000	9.248580000	5.250426000
C	-2.797645000	8.447546000	0.555880000
H	-5.840531000	9.423160000	2.331046000
H	-5.407149000	7.828237000	1.702546000
H	-5.856371000	8.006202000	3.395457000
H	0.989640000	6.439436000	3.884238000
H	2.577439000	6.170207000	3.143463000
H	2.192903000	5.441854000	4.715877000
H	0.056598000	3.877416000	0.609270000
H	1.258349000	5.182985000	0.576646000
H	-0.336667000	5.461718000	1.293748000
H	2.260792000	2.049361000	2.110067000
H	3.280596000	3.476722000	1.895348000
H	1.427258000	1.942842000	6.119810000
H	0.912014000	0.269600000	5.842195000
H	2.022319000	1.048352000	4.711040000
H	-2.756238000	2.059873000	4.981727000
H	-2.006093000	0.736748000	5.874520000
H	-1.704958000	2.407230000	6.369981000
H	-1.484046000	0.870191000	2.337873000
H	-0.788424000	-0.421506000	3.331825000
H	-3.580135000	2.054107000	2.728092000
H	-5.132365000	1.933602000	1.893576000
H	-3.866793000	5.897507000	1.069240000
H	-4.335633000	4.379021000	0.294054000
H	-2.816323000	4.479548000	1.180776000
H	-7.206845000	4.217218000	3.341813000
H	-6.980858000	4.293649000	1.591541000
H	-6.780353000	5.753815000	2.576118000
H	-4.565103000	2.534349000	6.283776000
H	-5.707913000	3.381560000	7.336166000
H	-2.997875000	6.547682000	6.704874000
H	-3.815662000	5.609711000	7.960693000
H	-2.546618000	4.861717000	6.990854000
H	-7.000859000	5.894722000	5.211846000
H	-6.583587000	6.129299000	6.915150000
H	-5.900485000	7.196764000	5.683549000
C	-0.229362000	9.210275000	2.187823000
H	-1.525458000	9.578186000	6.185074000
H	-0.969615000	10.926100000	5.186810000
H	-0.109847000	9.381848000	5.147059000
H	-3.756881000	8.035712000	0.235846000
H	-2.635252000	9.376462000	-0.004303000
H	-2.014387000	7.742612000	0.257917000
H	-0.106608000	10.122065000	1.590277000
H	0.527904000	9.226309000	2.975581000
H	-0.010303000	8.357977000	1.535758000
H	-6.180875000	2.951093000	5.689511000
H	-5.075313000	1.924289000	3.664336000
H	0.252547000	0.552541000	2.284649000
H	3.092232000	2.765171000	3.502403000

81
1

Cd	-1.439437000	4.793764000	3.800565000
Al	-3.576360000	6.143718000	3.887136000
N	0.426541000	3.864829000	3.604128000
N	-5.249325000	5.422647000	3.707816000
C	-3.384274000	8.144341000	4.608930000
C	-3.330205000	8.175144000	3.147028000
Si	0.942682000	3.568872000	1.990952000
Si	1.281037000	3.561343000	5.065179000
Si	-5.599519000	4.643727000	2.185107000
Si	-6.181918000	5.116436000	5.146903000
C	-2.028032000	8.031409000	5.070292000
C	-4.509710000	8.758658000	5.395803000
C	-1.968428000	8.027075000	2.779653000
C	-4.452296000	8.687189000	2.288811000
C	2.142918000	4.887313000	1.366807000
C	-0.563470000	3.609867000	0.845384000
C	1.787001000	1.892725000	1.799913000
C	3.068853000	4.162436000	4.986610000
C	0.422958000	4.485648000	6.476589000
C	1.304678000	1.732262000	5.524927000
C	-5.179705000	2.806214000	2.194039000
C	-4.539266000	5.410408000	0.824219000
C	-7.405551000	4.869171000	1.704252000
C	-6.844845000	3.355264000	5.247839000
C	-5.055637000	5.335396000	6.648223000
C	-7.663720000	6.269342000	5.295111000
C	-1.177329000	7.957256000	3.957397000
C	-1.642712000	8.010775000	6.515869000
H	-4.446639000	9.854691000	5.345798000
H	-4.477086000	8.478205000	6.452266000
H	-5.488237000	8.470629000	5.001888000
C	-1.428391000	7.963572000	1.386061000
H	-4.613505000	9.755894000	2.484345000
H	-4.222492000	8.580946000	1.225870000
H	-5.399377000	8.171177000	2.484282000
H	1.679747000	5.881528000	1.381590000
H	2.462913000	4.684062000	0.337600000
H	3.040450000	4.935770000	1.993467000
H	-1.323594000	2.878451000	1.147465000
H	-0.277419000	3.380475000	-0.188062000
H	-1.036553000	4.601628000	0.828321000
H	1.126228000	1.076938000	2.113757000
H	2.074158000	1.714715000	0.756756000
H	3.116177000	5.232879000	4.754684000
H	3.582583000	3.999964000	5.941689000
H	3.637459000	3.630667000	4.214558000
H	-0.611872000	4.146658000	6.623641000
H	0.947242000	4.324731000	7.426153000
H	0.401344000	5.567318000	6.294736000
H	0.284851000	1.338981000	5.608720000
H	1.810853000	1.565839000	6.483469000
H	-4.153276000	2.649908000	2.548925000
H	-5.245469000	2.394799000	1.179615000
H	-4.799936000	6.455804000	0.634801000
H	-4.696567000	4.854914000	-0.108178000
H	-3.464443000	5.358674000	1.041799000
H	-8.089629000	4.405152000	2.423028000
H	-7.600792000	4.414571000	0.725772000
H	-7.662694000	5.932564000	1.638524000
H	-6.034529000	2.618643000	5.230260000
H	-7.386157000	3.232525000	6.194010000
H	-4.557939000	6.310799000	6.680910000
H	-5.634912000	5.241339000	7.574551000
H	-4.278401000	4.561266000	6.667951000
H	-8.326293000	6.165632000	4.428281000
H	-8.249653000	6.030702000	6.190900000
H	-7.370821000	7.321875000	5.361700000
C	0.315216000	7.801417000	3.959560000
H	-2.111477000	7.177223000	7.052492000
H	-1.956577000	8.935193000	7.017032000
H	-0.562875000	7.913363000	6.645643000
H	-2.201500000	7.692062000	0.661915000
H	-1.015158000	8.934452000	1.081631000
H	-0.616922000	7.231814000	1.300437000
H	0.777491000	8.497528000	3.250418000
H	0.738417000	8.012597000	4.945088000
H	0.646345000	6.791476000	3.676110000
H	-7.543793000	3.109868000	4.441409000
H	-5.844895000	2.219801000	2.834243000
H	1.824457000	1.138613000	4.764962000
H	2.699256000	1.834107000	2.405105000

107
IM2

Cd	-1.227237000	5.232518000	3.952062000
Al	-3.212319000	6.813492000	3.741773000
N	-4.320487000	6.557981000	2.281073000
C	-4.251787000	7.679129000	5.457880000
C	-3.448220000	8.690498000	4.779930000
Si	-3.847281000	7.350370000	0.803668000
Si	-5.532307000	5.313920000	2.369034000
C	-3.405117000	7.041459000	6.404934000
C	-5.751437000	7.666763000	5.429775000
C	-2.138567000	8.636796000	5.354853000
C	-4.041363000	9.859659000	4.043902000
C	-3.246376000	6.200567000	-0.564943000
C	-2.378268000	8.485275000	1.159851000
C	-5.262917000	8.372642000	0.093532000
C	-5.562092000	4.180303000	0.862848000
C	-5.175138000	4.201922000	3.859949000
C	-7.287277000	5.992907000	2.526744000
C	-2.118738000	7.624999000	6.331294000
C	-3.787436000	5.925655000	7.322058000
H	-6.150767000	8.400232000	6.143904000
H	-6.167821000	6.690017000	5.693825000
H	-6.131804000	7.932378000	4.439722000
C	-1.002917000	9.526080000	4.958515000
H	-4.597856000	10.502553000	4.739572000
H	-3.265173000	10.476137000	3.582479000
H	-4.737465000	9.548955000	3.256915000
H	-2.482639000	5.502818000	-0.204413000
H	-2.786217000	6.807753000	-1.354765000
H	-2.598868000	9.268463000	1.890566000
H	-2.082735000	8.985051000	0.229108000
H	-1.502421000	7.925539000	1.513701000
H	-6.093558000	7.728201000	-0.218827000
H	-4.935921000	8.934755000	-0.789323000
H	-5.656073000	9.087939000	0.824434000
H	-4.575091000	3.765169000	0.634123000
H	-6.241739000	3.343279000	1.066160000
H	-5.030598000	4.752374000	4.797904000
H	-6.011091000	3.508172000	4.014289000
H	-4.270604000	3.602975000	3.691805000
H	-7.459872000	6.800757000	1.806543000
H	-8.016099000	5.200521000	2.316795000
H	-7.505246000	6.385685000	3.523900000
C	-0.934633000	7.161942000	7.123577000
H	-4.832213000	5.630580000	7.191887000
H	-3.651782000	6.219630000	8.370389000
H	-3.164419000	5.033148000	7.155378000
H	-0.942345000	9.649097000	3.871779000
H	-1.118273000	10.527673000	5.393959000
H	-0.041477000	9.133514000	5.299812000
H	-4.046603000	5.615696000	-1.025976000
H	-5.935258000	4.688276000	-0.033087000
H	-0.910857000	7.617934000	8.122426000
H	-0.954445000	6.074259000	7.267973000
H	0.011473000	7.411046000	6.632941000
H	0.765698000	1.537681000	9.058394000
H	1.369454000	2.725829000	7.892212000
H	0.020272000	3.135262000	8.948490000
C	0.465388000	2.328277000	8.359495000
H	2.932313000	5.513331000	6.081127000
H	1.975179000	1.394118000	6.166735000
H	2.336228000	3.853070000	5.920172000
C	2.130722000	4.896758000	5.657212000

H	-2.222007000	2.955542000	9.234177000
C	-0.496632000	1.795225000	7.342840000
H	1.194646000	5.183198000	6.153530000
C	1.193975000	1.137592000	5.447554000
H	4.483868000	4.867426000	3.492659000
H	1.377523000	0.110959000	5.111599000
C	-2.682775000	2.098366000	8.734667000
C	-0.167908000	1.251260000	6.064347000
C	-1.915848000	1.690690000	7.515385000
C	3.607396000	4.376631000	3.052567000
H	2.889919000	7.458555000	3.815922000
H	-2.723166000	1.276822000	9.460743000
H	3.687611000	3.302433000	3.255973000
H	1.302459000	1.803028000	4.581567000
Si	2.017392000	5.092397000	3.778178000
C	2.002743000	6.950727000	3.418267000
H	1.118747000	7.429728000	3.860135000
Al	-1.498391000	3.051639000	5.762798000
H	3.662270000	4.516663000	1.966896000
C	-1.380637000	0.825214000	5.442286000
C	-2.460436000	1.088099000	6.342604000
N	0.605230000	4.317094000	3.197966000
C	-1.470323000	0.132586000	4.118630000
H	-1.377499000	-0.953244000	4.249492000
H	1.688278000	5.175334000	0.184741000
H	1.961291000	7.136564000	2.338262000
C	-3.900690000	0.756261000	6.106641000
H	-0.670563000	0.453198000	3.445338000
Si	0.319283000	3.663382000	1.640065000
H	-4.566514000	1.462208000	6.611168000
C	0.629862000	4.892581000	0.235465000
H	-4.134064000	-0.247157000	6.484064000
H	2.427443000	2.375001000	1.204595000
H	0.052602000	5.812873000	0.387341000
H	1.250831000	1.366838000	2.053927000
C	1.362422000	2.129657000	1.274413000
H	-2.424325000	0.320651000	3.618014000
H	-2.186003000	3.990604000	1.609509000
C	-1.499831000	3.136778000	1.521371000
H	-4.147955000	0.774878000	5.041585000
H	-1.761954000	2.419173000	2.308384000
H	0.349809000	4.478383000	-0.740913000
H	1.063149000	1.679226000	0.320067000
H	-1.710503000	2.660290000	0.555952000
H	-3.712629000	2.372654000	8.489344000

107
TS2

Cd	-0.953964000	5.180159000	4.105691000
Al	-3.058734000	6.585094000	3.836561000
N	-4.192881000	6.261708000	2.416045000
C	-4.072178000	7.254787000	5.652447000
C	-3.443494000	8.380243000	4.965920000
Si	-3.848664000	7.110307000	0.934379000
Si	-5.262157000	4.891204000	2.507587000
C	-3.094215000	6.697444000	6.520140000
C	-5.559250000	7.063772000	5.714710000
C	-2.104056000	8.473479000	5.460720000
C	-4.219707000	9.493943000	4.319245000
C	-3.082164000	6.038407000	-0.415631000
C	-2.572923000	8.460545000	1.282897000
C	-5.401167000	7.908184000	0.225084000
C	-5.208439000	3.795383000	0.974039000
C	-4.753821000	3.792894000	3.961530000
C	-7.071214000	5.384538000	2.721182000
C	-1.897456000	7.441250000	6.393845000
C	-3.262638000	5.493245000	7.390819000
H	-5.993438000	7.713685000	6.486986000
H	-5.844507000	6.034908000	5.952740000
H	-6.029776000	7.327297000	4.763540000
C	-1.118290000	9.516217000	5.038028000
H	-4.807943000	10.033126000	5.074444000
H	-3.553034000	10.221000000	3.847487000
H	-4.918928000	9.134495000	3.555752000
H	-2.267885000	5.421963000	-0.019383000
H	-2.654604000	6.685498000	-1.191674000
H	-2.933720000	9.222727000	1.979161000
H	-2.325771000	8.968182000	0.342510000
H	-1.633447000	8.052047000	1.678709000
H	-6.138234000	7.150424000	-0.065950000
H	-5.165875000	8.496075000	-0.670122000
H	-5.879369000	8.573671000	0.952592000
H	-4.189418000	3.480153000	0.726418000
H	-5.804546000	2.893988000	1.162611000
H	-4.645571000	4.334478000	4.908719000
H	-5.511805000	3.014903000	4.116667000
H	-3.798168000	3.290456000	3.764612000
H	-7.356868000	6.146173000	1.986842000
H	-7.719928000	4.513939000	2.564944000
H	-7.288871000	5.784038000	3.715964000
C	-0.617922000	7.110188000	7.101221000
H	-4.279350000	5.094131000	7.339029000
H	-3.055298000	5.739385000	8.439481000
H	-2.569859000	4.688740000	7.103163000
H	-1.122275000	9.664721000	3.952601000
H	-1.351752000	10.484885000	5.499946000
H	-0.098578000	9.254513000	5.332062000
H	-3.801635000	5.373022000	-0.900714000
H	-5.635944000	4.288112000	0.093949000
H	-0.584694000	7.552013000	8.106129000
H	-0.500730000	6.025924000	7.221856000
H	0.259471000	7.477445000	6.559184000
H	2.167586000	1.689845000	8.792627000
H	2.973295000	2.653052000	7.549178000
H	1.601346000	3.323229000	8.428681000
C	2.001139000	2.407754000	7.980156000
H	3.422580000	5.973338000	5.948180000
H	3.483887000	1.875515000	5.590376000
H	3.078467000	4.240323000	5.879907000
C	2.678283000	5.230458000	5.637018000
H	-0.722974000	2.817866000	8.877991000
C	1.068852000	1.825315000	6.962871000
H	1.782546000	5.384890000	6.251017000
C	2.726540000	1.394632000	4.967242000
H	4.758350000	5.638922000	3.297579000
H	3.069963000	0.374761000	4.756159000
C	-1.039632000	1.851151000	8.472966000
C	1.392170000	1.368669000	5.646066000
C	-0.308058000	1.521508000	7.209872000
C	3.955883000	5.004701000	2.901297000
H	2.719030000	7.860912000	3.800344000
H	-0.852010000	1.094574000	9.245447000
H	4.256105000	3.959666000	3.040708000
H	2.683650000	1.940510000	4.017936000
Si	2.328361000	5.378317000	3.785239000
C	1.912769000	7.198029000	3.462874000
H	0.996777000	7.498057000	3.989837000
Al	-0.114805000	2.990972000	5.417068000
H	3.890347000	5.192712000	1.823813000
C	0.202361000	0.808069000	5.078161000
C	-0.839754000	0.892036000	6.057563000
N	1.039046000	4.324334000	3.335008000
C	0.133131000	0.091505000	3.766076000
H	0.486801000	-0.941567000	3.879711000
H	1.710266000	5.750289000	0.386944000
H	1.744553000	7.387361000	2.396872000
C	-2.247800000	0.420183000	5.871407000
H	0.757535000	0.570961000	3.008149000
Si	0.727210000	3.851228000	1.704686000
H	-2.952167000	1.012674000	6.462120000
C	0.713589000	5.306469000	0.494702000
H	-2.355250000	-0.627555000	6.178736000
H	2.974652000	3.044528000	0.950661000
H	0.034405000	6.097230000	0.836182000
H	2.059364000	1.708600000	1.651562000
C	1.975025000	2.603842000	1.026496000
H	-0.887788000	0.045771000	3.378171000
H	-1.790831000	3.786125000	1.817172000
C	-0.993265000	3.059128000	1.606612000

H	-2.559096000	0.491118000	4.825224000
H	-1.110257000	2.227621000	2.311096000
H	0.382172000	4.992035000	-0.502581000
H	1.679283000	2.278538000	0.021516000
H	-1.181022000	2.668058000	0.598921000
H	-2.120268000	1.897476000	8.313272000

107
2

Cd	0.449879000	3.120629000	6.602126000
Al	-0.103594000	3.943049000	4.204849000
Al	0.936282000	2.289784000	9.008934000
C	-0.351415000	6.055393000	3.750332000
N	-0.053410000	2.927531000	2.659169000
C	-1.589703000	5.593906000	4.358358000
C	0.523670000	6.424434000	4.814833000
C	0.865874000	0.158980000	9.425527000
N	1.017358000	3.348657000	10.524948000
C	2.221829000	0.495700000	9.010410000
C	0.131361000	-0.150397000	8.238630000
C	-0.236525000	6.420019000	2.297110000
Si	1.485040000	2.736205000	1.867485000
Si	-1.429928000	1.944989000	2.255887000
C	-1.443404000	5.739349000	5.771896000
C	-2.895423000	5.504507000	3.622278000
C	-0.158121000	6.235389000	6.043490000
C	1.910092000	6.972508000	4.679611000
C	0.515705000	-0.251446000	10.829393000
Si	-0.469902000	3.655801000	11.375015000
Si	2.484818000	4.238843000	10.810267000
C	2.266175000	0.370830000	7.592342000
C	3.393571000	0.482690000	9.948922000
C	0.994935000	-0.026028000	7.130495000
C	-1.310060000	-0.551051000	8.189543000
H	0.771253000	6.757274000	2.043354000
H	-0.925539000	7.241613000	2.060408000
H	-0.482585000	5.579122000	1.638065000
C	2.364672000	1.126640000	2.309323000
C	2.656062000	4.110101000	2.417249000
C	1.316794000	2.826798000	-0.007217000
C	-0.983630000	0.163209000	1.827646000
C	-2.581857000	1.850875000	3.750522000
C	-2.379081000	2.627273000	0.775806000
C	-2.481027000	5.384878000	6.790547000
H	-2.794132000	5.008181000	2.653152000
H	-3.291790000	6.511528000	3.430915000
H	-3.652624000	4.963740000	4.197435000
C	0.423357000	6.489069000	7.403296000
H	2.242511000	6.988896000	3.639515000
H	2.643085000	6.388028000	5.248212000
H	1.954539000	8.001374000	5.057968000
H	-0.552686000	-0.456704000	10.934427000
H	1.050775000	-1.172524000	11.097139000
H	0.785369000	0.511911000	11.568270000
C	-1.144445000	5.404976000	11.155773000
C	-1.817488000	2.516437000	10.700915000
C	-0.290582000	3.348000000	13.225353000
C	2.199842000	6.069045000	11.165115000
C	3.562906000	4.166683000	9.258615000
C	3.472697000	3.575743000	12.274517000
C	3.446654000	0.642886000	6.714570000
H	3.133058000	0.914558000	10.918863000
H	3.722747000	-0.549905000	10.130084000
H	4.253474000	1.034844000	9.558417000
C	0.637316000	-0.241104000	5.689341000
H	-1.846893000	-0.228301000	9.085368000
H	-1.825155000	-0.116351000	7.326427000
H	-1.414541000	-1.641641000	8.116347000
H	2.505573000	1.047767000	3.393747000
H	3.358053000	1.105578000	1.844284000
H	1.824115000	0.235158000	1.979194000
H	2.310328000	5.101636000	2.111383000
H	3.640666000	3.948614000	1.961759000
H	2.804434000	4.123216000	3.504915000
H	0.727227000	1.994918000	-0.408566000
H	2.303150000	2.788873000	-0.484864000
H	0.829144000	3.759213000	-0.313904000
H	-0.369325000	0.082960000	0.924306000
H	-1.905101000	-0.403132000	1.644498000
H	-0.447497000	-0.327304000	2.647490000
H	-2.094288000	1.331269000	4.584890000
H	-3.486619000	1.287245000	3.492311000
H	-2.904870000	2.832619000	4.112705000
H	-2.728325000	3.652509000	0.935389000
H	-3.256499000	2.007407000	0.554779000
H	-1.744641000	2.635047000	-0.117655000
H	-2.027914000	5.102567000	7.747663000
H	-3.105057000	4.547875000	6.459904000
H	-3.151471000	6.231601000	6.990748000
H	0.207242000	5.678095000	8.110379000
H	0.024755000	7.411490000	7.846320000
H	1.511396000	6.596635000	7.357893000
H	-1.175219000	5.695083000	10.099418000
H	-2.171356000	5.446273000	11.539624000
H	-0.563022000	6.160826000	11.690519000
H	-1.603439000	1.458582000	10.876600000
H	-2.764841000	2.746345000	11.203560000
H	-1.983426000	2.656241000	9.624551000
H	0.405058000	4.061556000	13.682427000
H	-1.254936000	3.458758000	13.735387000
H	0.086594000	2.339733000	13.430023000
H	1.738501000	6.240259000	12.143843000
H	3.168838000	6.583388000	11.170137000
H	1.571551000	6.544389000	10.404310000
H	3.106666000	4.735218000	8.438799000
H	4.542263000	4.614520000	9.467524000
H	3.743445000	3.149432000	8.892587000
H	3.922196000	2.597952000	12.076998000
H	4.285695000	4.267325000	12.527412000
H	2.836514000	3.474361000	13.160973000
H	3.195152000	1.323613000	5.890675000
H	4.273711000	1.089404000	7.273114000
H	3.817697000	-0.283259000	6.256449000
H	0.750433000	0.666201000	5.079019000
H	1.282675000	-1.003496000	5.234661000
H	-0.396317000	-0.580954000	5.578504000

104
IM1’

Cd	9.526694000	14.486851000	14.726865000
C	14.048249000	14.140573000	15.369939000
Al	11.265866000	13.851212000	16.518967000
N	12.987520000	14.726329000	15.677928000
N	14.967867000	13.514075000	14.927896000
N	11.258404000	14.497914000	18.267301000
C	11.457499000	11.791029000	16.252757000
N	8.274432000	14.870688000	13.050611000
C	12.850137000	16.179631000	15.270208000
C	16.367503000	13.244767000	15.265777000
Si	9.788586000	15.267224000	18.842810000
Si	12.568648000	14.379692000	19.410720000
C	10.105112000	11.547350000	16.798200000
C	12.638816000	11.130983000	16.947958000
C	11.304551000	11.646481000	14.792652000
Si	6.560390000	14.822158000	13.175029000
Si	9.140997000	15.260866000	11.620172000
H	11.791332000	16.267942000	14.987291000
C	13.116406000	17.069286000	16.475370000
C	13.723336000	16.532018000	14.076462000
C	16.470984000	11.911545000	16.002266000
H	16.871906000	13.141391000	14.296115000
C	17.004106000	14.392897000	16.040250000
C	8.231842000	14.709329000	17.936746000
C	9.414874000	14.881609000	20.656401000
C	9.878033000	17.140512000	18.632520000
C	14.232965000	14.052592000	18.581745000
C	12.364680000	12.987436000	20.671797000

C	12.863553000	15.988973000	20.361800000
C	9.225355000	11.414626000	15.741915000
C	9.820525000	11.328880000	18.250292000
H	13.595303000	11.426728000	16.505667000
H	12.566259000	10.036841000	16.870191000
H	12.677801000	11.380717000	18.012669000
C	9.968095000	11.460379000	14.502285000
C	12.429510000	11.680194000	13.808454000
C	6.017932000	14.920608000	14.979751000
C	5.728220000	16.265339000	12.278870000
C	5.820812000	13.235250000	12.458965000
C	10.940192000	14.692146000	11.791192000
C	8.449674000	14.418848000	10.076017000
C	9.204660000	17.119673000	11.280548000
H	14.170381000	17.016014000	16.772453000
H	12.492778000	16.768309000	17.320878000
H	12.883976000	18.108810000	16.221138000
H	13.472050000	17.541206000	13.736595000
H	14.786722000	16.524903000	14.343153000
H	16.012063000	11.979841000	16.993328000
H	17.526246000	11.651296000	16.128454000
H	15.981429000	11.111349000	15.440509000
H	16.888794000	15.340381000	15.504972000
H	18.074429000	14.200320000	16.164066000
H	16.553904000	14.485891000	17.032848000
H	8.052319000	15.300033000	17.034380000
H	7.367807000	14.861031000	18.596577000
H	8.252916000	13.653843000	17.647054000
H	9.160947000	13.822025000	20.778888000
H	8.529773000	15.461778000	20.945934000
H	10.208548000	15.121753000	21.368082000
H	10.714245000	17.589507000	19.178550000
H	8.953539000	17.611850000	18.987764000
H	9.990720000	17.398920000	17.571956000
H	14.227679000	13.186013000	17.914968000
H	14.973556000	13.856951000	19.367301000
H	14.565828000	14.928135000	18.017554000
H	11.424370000	13.041822000	21.226779000
H	13.184147000	13.033552000	21.399878000
H	12.407578000	12.002092000	20.194496000
H	13.134666000	16.802824000	19.679240000
H	13.705620000	15.845779000	21.050284000
H	12.010648000	16.327423000	20.955995000
C	7.744911000	11.197805000	15.806701000
H	8.745953000	11.304079000	18.453593000
H	10.265302000	12.106844000	18.880684000
H	10.234498000	10.366820000	18.585244000
C	9.334708000	11.291394000	13.153882000
H	12.162983000	11.139503000	12.895266000
H	13.333889000	11.213653000	14.215207000
H	12.705638000	12.697447000	13.501974000
H	6.431644000	14.109670000	15.591177000
H	4.925681000	14.853343000	15.051854000
H	6.319442000	15.871489000	15.434929000
H	6.073754000	17.226703000	12.675964000
H	4.639650000	16.220500000	12.405035000
H	5.932898000	16.256834000	11.202213000
H	6.064754000	13.126241000	11.396346000
H	4.727886000	13.234582000	12.552901000
H	6.199429000	12.344911000	12.974131000
H	11.440516000	15.186182000	12.634474000
H	11.512436000	14.935549000	10.887585000
H	11.008562000	13.609929000	11.951021000
H	8.448117000	13.328669000	10.191337000
H	9.057342000	14.664515000	9.196639000
H	7.421548000	14.731391000	9.861846000
H	8.202825000	17.531798000	11.118826000
H	9.808231000	17.345977000	10.393000000
H	9.643453000	17.653113000	12.132692000
H	7.483133000	10.158869000	15.565233000
H	7.214666000	11.830188000	15.084998000
H	7.340447000	11.416043000	16.798823000
H	8.630215000	10.450941000	13.159505000
H	10.081011000	11.093378000	12.378999000
H	8.769443000	12.181409000	12.842641000
H	13.567075000	15.843493000	13.240422000

104
TS1'

Cd	10.180249000	14.091335000	14.774074000
C	12.950899000	14.467086000	15.208574000
Al	11.451173000	13.718887000	17.096722000
N	13.051669000	14.584187000	16.494563000
N	13.404667000	14.769350000	14.112596000
N	10.800791000	14.615475000	18.572828000
C	11.475298000	11.651107000	17.165146000
N	8.788320000	14.734097000	13.294278000
C	14.227208000	15.326229000	17.065623000
C	13.463605000	14.086516000	12.831163000
Si	9.535842000	15.808389000	18.362319000
Si	11.750653000	14.653813000	20.036033000
C	10.090063000	11.390147000	16.683541000
C	11.784554000	11.046118000	18.535274000
C	12.335396000	11.253618000	16.034607000
Si	7.196606000	14.074418000	13.219757000
Si	9.453418000	15.840787000	12.152668000
H	14.029259000	15.342951000	18.141486000
C	15.545325000	14.605496000	16.814526000
C	14.240485000	16.762729000	16.554591000
C	14.583002000	13.046585000	12.838087000
H	12.494607000	13.571040000	12.696549000
C	13.665110000	15.108601000	11.718538000
C	8.125721000	15.190564000	17.272062000
C	8.698711000	16.271559000	19.989652000
C	10.214850000	17.372328000	17.556302000
C	13.332608000	13.602297000	19.896431000
C	10.822985000	13.961836000	21.522695000
C	12.344837000	16.397923000	20.466391000
C	10.150709000	10.961773000	15.374520000
C	8.914908000	11.386703000	17.607911000
H	12.838800000	11.144287000	18.798993000
H	11.542292000	9.974312000	18.527062000
H	11.201238000	11.513180000	19.335370000
C	11.538128000	10.903203000	14.968333000
C	13.823832000	11.156900000	16.122478000
C	6.818449000	13.081418000	14.784673000
C	5.823547000	15.373049000	13.143651000
C	6.967451000	12.959119000	11.709265000
C	10.297554000	14.930765000	10.724651000
C	8.189067000	16.978228000	11.332510000
C	10.673895000	16.989952000	13.029839000
H	15.727704000	14.493019000	15.739995000
H	15.553260000	13.615072000	17.278956000
H	16.371314000	15.185240000	17.239822000
H	15.016675000	17.331713000	17.077320000
H	14.453409000	16.801315000	15.481556000
H	15.555127000	13.538800000	12.949926000
H	14.583968000	12.492796000	11.893441000
H	14.454833000	12.338703000	13.659639000
H	12.867581000	15.853418000	11.717978000
H	13.670203000	14.607559000	10.746012000
H	14.624038000	15.620719000	11.851298000
H	8.277796000	15.378500000	16.202628000
H	7.209696000	15.727668000	17.550458000
H	7.927626000	14.122679000	17.404220000
H	8.172368000	15.409448000	20.414922000
H	7.946721000	17.040917000	19.775493000
H	9.364764000	16.673838000	20.757175000
H	10.988018000	17.848390000	18.169364000
H	9.419329000	18.107058000	17.382782000
H	10.660453000	17.134695000	16.581380000
H	13.589703000	13.256656000	18.889514000
H	13.248622000	12.711609000	20.526644000
H	14.187758000	14.181670000	20.264462000
H	9.979028000	14.585074000	21.829659000
H	11.503445000	13.876177000	22.378964000
H	10.435424000	12.959614000	21.305314000
H	12.958072000	16.820228000	19.661315000
H	12.965185000	16.360613000	21.370531000
H	11.531400000	17.103735000	20.658015000
C	9.034989000	10.485830000	14.496069000
H	7.961445000	11.457901000	17.077655000

H	8.967706000	12.211721000	18.327837000
H	8.894762000	10.456953000	18.195132000
C	11.955801000	10.452944000	13.602403000
H	14.239347000	10.604517000	15.275150000
H	14.127157000	10.624054000	17.032458000
H	14.314143000	12.137327000	16.152151000
H	7.722139000	12.686357000	15.258149000
H	6.161573000	12.231720000	14.564548000
H	6.313091000	13.713524000	15.522347000
H	5.972774000	16.146436000	13.906332000
H	4.862273000	14.885467000	13.350595000
H	5.739134000	15.868344000	12.172719000
H	7.088401000	13.532229000	10.781942000
H	5.965508000	12.513227000	11.690773000
H	7.700787000	12.144822000	11.686963000
H	11.024329000	14.189116000	11.072148000
H	10.810178000	15.613829000	10.036393000
H	9.537419000	14.387226000	10.150427000
H	7.482378000	16.429004000	10.701106000
H	8.718630000	17.689243000	10.686028000
H	7.613569000	17.554252000	12.064354000
H	10.126960000	17.589998000	13.768160000
H	11.145027000	17.686129000	12.324808000
H	11.480715000	16.471521000	13.561425000
H	9.248440000	9.473655000	14.130953000
H	8.901193000	11.118856000	13.608878000
H	8.081109000	10.451553000	15.025320000
H	11.607039000	9.431795000	13.402538000
H	13.040627000	10.461857000	13.476581000
H	11.523829000	11.087791000	12.817752000
H	13.275569000	17.246010000	16.735375000

104
IM2’

Cd	10.290302000	15.420091000	16.276044000
C	11.499641000	15.610606000	14.451212000
Al	9.285162000	16.388144000	13.307857000
N	12.756068000	15.563686000	14.672061000
N	10.881879000	15.407409000	13.250275000
N	9.772363000	14.503091000	18.070772000
N	7.757035000	15.394309000	13.426685000
C	9.179351000	18.258750000	12.273863000
C	13.323906000	15.754914000	15.989120000
C	11.646287000	14.740125000	12.159179000
Si	9.465076000	12.814948000	17.900125000
Si	10.049880000	15.385656000	19.523509000
Si	6.484392000	15.356009000	14.623154000
Si	7.586923000	14.374283000	12.008264000
C	7.797618000	18.589563000	12.469070000
C	9.834868000	18.444010000	13.578485000
C	9.908344000	18.414246000	10.963500000
H	12.571268000	16.232216000	16.668750000
C	13.729423000	14.413634000	16.603748000
C	14.515437000	16.706934000	15.893014000
C	12.736516000	15.608430000	11.526349000
H	10.901718000	14.513646000	11.386275000
C	12.229652000	13.395993000	12.611593000
C	9.735859000	12.338576000	16.085163000
C	10.636050000	11.756811000	18.934562000
C	7.702333000	12.362158000	18.394555000
C	9.809665000	17.227034000	19.179463000
C	11.812669000	15.176510000	20.166071000
C	8.859706000	14.866794000	20.890410000
C	7.031511000	16.169878000	16.231007000
C	4.904564000	16.198084000	14.020628000
C	6.008280000	13.584911000	15.067909000
C	8.432184000	15.212538000	10.533615000
C	8.329959000	12.672833000	12.287567000
C	5.809851000	14.157252000	11.416924000
C	7.591070000	18.932972000	13.813126000
C	6.748547000	18.505143000	11.407890000
C	11.303477000	18.692906000	13.762252000
C	8.826713000	18.836054000	14.496432000
H	10.878861000	17.913109000	10.968853000
H	10.096687000	19.477326000	10.760057000
H	9.337666000	18.009033000	10.123946000
H	14.433477000	13.898743000	15.941295000
H	12.854538000	13.767373000	16.742094000
H	14.204600000	14.555837000	17.580261000
H	14.938687000	16.897925000	16.884862000
H	9.786010000	18.521120000	16.401896000
H	15.294303000	16.268658000	15.259851000
H	12.328652000	16.534243000	11.110933000
H	13.213240000	15.060333000	10.705356000
H	13.499394000	15.861184000	12.266447000
H	11.486283000	12.806665000	13.157499000
H	13.096099000	13.540962000	13.260285000
H	12.543809000	12.823166000	11.732285000
H	9.159837000	12.955290000	15.380817000
H	9.436028000	11.298575000	15.906578000
H	10.794813000	12.418965000	15.804959000
H	11.681833000	11.957718000	18.674808000
H	10.446455000	10.688633000	18.774342000
H	10.516007000	11.953917000	20.006228000
H	7.561885000	12.475115000	19.475350000
H	7.468723000	11.322219000	18.136094000
H	6.967964000	13.005946000	17.898946000
H	10.586876000	17.612956000	18.505578000
H	9.876196000	17.812762000	20.103968000
H	8.833130000	17.428719000	18.723363000
H	12.039505000	14.127099000	20.384682000
H	11.979341000	15.755103000	21.082793000
H	12.537616000	15.519996000	19.417774000
H	7.817180000	14.994481000	20.577972000
H	9.019532000	15.467919000	21.793316000
H	8.998613000	13.815734000	21.168754000
H	7.590319000	15.477461000	16.872903000
H	6.153010000	16.489666000	16.805133000
H	7.647541000	17.058865000	16.062577000
H	5.118052000	17.079779000	13.410774000
H	4.294667000	16.521644000	14.872510000
H	4.292058000	15.517591000	13.421189000
H	5.600025000	13.020944000	14.223671000
H	5.233347000	13.608072000	15.844775000
H	6.855724000	13.019369000	15.468616000
H	7.781851000	16.005601000	10.148185000
H	8.572240000	14.477048000	9.731779000
H	9.413012000	15.666596000	10.710763000
H	9.379799000	12.751834000	12.586704000
H	8.277296000	12.057686000	11.381555000
H	7.801895000	12.141698000	13.087291000
H	5.170355000	13.591909000	12.099765000
H	5.841287000	13.604582000	10.469194000
H	5.328884000	15.121052000	11.219970000
C	6.307817000	19.360898000	14.458229000
H	5.948050000	19.230733000	11.580228000
H	6.282162000	17.510939000	11.378075000
H	7.163782000	18.702635000	10.414660000
H	11.921662000	18.036671000	13.144006000
H	11.611258000	18.537515000	14.800759000
H	11.536899000	19.732680000	13.493071000
C	9.081230000	19.208951000	15.921020000
H	6.425201000	20.340054000	14.938132000
H	5.973622000	18.660417000	15.232583000
H	5.497287000	19.447088000	13.730719000
H	8.164491000	19.219301000	16.517281000
H	9.526011000	20.211574000	15.983799000
H	14.213947000	17.661075000	15.449828000

104
TS2’A

Cd	10.271078000	14.541902000	16.236213000
C	11.254877000	15.785961000	14.744706000
Al	9.315254000	16.728359000	13.091569000
N	12.276046000	16.466309000	15.100609000
N	10.833137000	15.730073000	13.409444000

N	9.736632000	13.271887000	17.795599000
N	7.831297000	16.017035000	13.874583000
C	9.177652000	17.958617000	11.372157000
C	12.742679000	16.475574000	16.470329000
C	11.768613000	15.211214000	12.376067000
Si	9.838644000	11.573837000	17.500447000
Si	9.552890000	14.014666000	19.344286000
Si	6.954463000	16.561301000	15.278604000
Si	7.351073000	14.542931000	13.067759000
C	7.850277000	18.460223000	11.638396000
C	10.057863000	18.610672000	12.353828000
C	9.642511000	17.501316000	10.011148000
H	12.038396000	15.924923000	17.141020000
C	14.098421000	15.774776000	16.559340000
C	12.817210000	17.920781000	16.961318000
C	13.242051000	15.616083000	12.486378000
H	11.390134000	15.589199000	11.413656000
C	11.678182000	13.677890000	12.334308000
C	10.851301000	11.255167000	15.935523000
C	10.703838000	10.636723000	18.893913000
C	8.136263000	10.790541000	17.284029000
C	9.235699000	15.867650000	19.146792000
C	11.099937000	13.871426000	20.417795000
C	8.094120000	13.278847000	20.286349000
C	7.991610000	17.832440000	16.197577000
C	5.280092000	17.306463000	14.825211000
C	6.567051000	15.149933000	16.473875000
C	7.994302000	14.556904000	11.290663000
C	8.095986000	13.049810000	13.929534000
C	5.483840000	14.333874000	12.946360000
C	7.898228000	19.321648000	12.738229000
C	6.616694000	18.049043000	10.899832000
C	11.548301000	18.736494000	12.264083000
C	9.242286000	19.392197000	13.200845000
H	10.642843000	17.061512000	10.040113000
H	9.686317000	18.355418000	9.322306000
H	8.975374000	16.754215000	9.573042000
H	14.824323000	16.273728000	15.908274000
H	14.013781000	14.730307000	16.240284000
H	14.476460000	15.794358000	17.587038000
H	13.177537000	17.959057000	17.994740000
H	10.489982000	19.750027000	14.925779000
H	13.499521000	18.499493000	16.329537000
H	13.376129000	16.688803000	12.619177000
H	13.758124000	15.300468000	11.571531000
H	13.723371000	15.118943000	13.332685000
H	10.656550000	13.340343000	12.138604000
H	11.997545000	13.258675000	13.296377000
H	12.332045000	13.268582000	11.555425000
H	10.373291000	11.653987000	15.033236000
H	10.979190000	10.177776000	15.775113000
H	11.854271000	11.693832000	16.016804000
H	11.730422000	10.991556000	19.037682000
H	10.748834000	9.568444000	18.649402000
H	10.179352000	10.729340000	19.851282000
H	7.528584000	10.937472000	18.184412000
H	8.211741000	9.711689000	17.102428000
H	7.592425000	11.235054000	16.443809000
H	10.069046000	16.387305000	18.654599000
H	9.118732000	16.329197000	20.134942000
H	8.324427000	16.075653000	18.577344000
H	11.327134000	12.835009000	20.685548000
H	10.983249000	14.439169000	21.349048000
H	11.971514000	14.275030000	19.887401000
H	7.167336000	13.394191000	19.712398000
H	7.957573000	13.773006000	21.255613000
H	8.233915000	12.209089000	20.479152000
H	8.996940000	17.453223000	16.421373000
H	7.523056000	18.099510000	17.151794000
H	8.108684000	18.748248000	15.614116000
H	5.321218000	17.841581000	13.872789000
H	4.940835000	18.011727000	15.593426000
H	4.516618000	16.526275000	14.737141000
H	5.910365000	14.395599000	16.026800000
H	6.032296000	15.562560000	17.339411000
H	7.448984000	14.623454000	16.857544000
H	7.452227000	15.286323000	10.680272000
H	7.831015000	13.566304000	10.847953000
H	9.067479000	14.764268000	11.191215000
H	9.188165000	13.142118000	13.890623000
H	7.825251000	12.100160000	13.453050000
H	7.793246000	12.997446000	14.980430000
H	5.004349000	14.173989000	13.916616000
H	5.262879000	13.457148000	12.325334000
H	5.010757000	15.203166000	12.477095000
C	6.761334000	20.099015000	13.327382000
H	5.949038000	18.902040000	10.741119000
H	6.046355000	17.288875000	11.451446000
H	6.855990000	17.634694000	9.916180000
H	11.971413000	18.020956000	11.557115000
H	12.037868000	18.575374000	13.230457000
H	11.811277000	19.742438000	11.908733000
C	9.773533000	20.276636000	14.285731000
H	6.588381000	19.867876000	14.384553000
H	5.825215000	19.906731000	12.797600000
H	6.960681000	21.176144000	13.263335000
H	8.977112000	20.672344000	14.921562000
H	10.302441000	21.135998000	13.851201000
H	11.829299000	18.392257000	16.921570000

104
6a

Cd	7.372874000	11.182325000	3.395892000
Si	4.990054000	12.833332000	2.059760000
N	6.351695000	12.927322000	3.120374000
C	8.460508000	9.363723000	3.642582000
C	4.315581000	11.069137000	2.120595000
C	5.465251000	13.208009000	0.274906000
C	3.626361000	14.023423000	2.576986000
Si	7.073367000	14.317373000	3.850938000
Al	9.605791000	7.323207000	3.979073000
N	9.151475000	8.726299000	2.693342000
N	8.495476000	8.718491000	4.807026000
H	5.044450000	10.329795000	1.758493000
H	3.428438000	10.961939000	1.485374000
H	4.027440000	10.787397000	3.140689000
H	5.859105000	14.225750000	0.175734000
H	4.606389000	13.113400000	-0.400109000
H	6.243203000	12.519087000	-0.076781000
H	3.287726000	13.821721000	3.599125000
H	2.761621000	13.931248000	1.909509000
H	3.961736000	15.066042000	2.534663000
C	7.373811000	15.691909000	2.598182000
C	8.740739000	13.798335000	4.581343000
C	6.023333000	15.005602000	5.253856000
N	8.735527000	5.719339000	3.771472000
C	11.633318000	7.354166000	4.217918000
C	9.453319000	9.334428000	1.393091000
C	7.742637000	9.200316000	5.955452000
H	8.015294000	15.346608000	1.779531000
H	7.858625000	16.555817000	3.067755000
H	6.434103000	16.044422000	2.157122000
H	8.632550000	13.030132000	5.360315000
H	9.239192000	14.652112000	5.055615000
H	9.428160000	13.415125000	3.815226000
H	5.053531000	15.362058000	4.890664000
H	6.523844000	15.847302000	5.747142000
H	5.830066000	14.237078000	6.011065000
Si	8.359807000	4.735499000	5.159621000
Si	8.111555000	5.304740000	2.200817000
C	12.004609000	6.014093000	4.728183000
C	11.782341000	8.255828000	5.390549000
C	12.294514000	7.784961000	2.907602000
H	10.212349000	8.688314000	0.936005000
C	8.239139000	9.348809000	0.461090000
C	10.081921000	10.731341000	1.496028000
H	7.463007000	10.255884000	5.744010000
C	8.605641000	9.219272000	7.214059000
C	6.436242000	8.426912000	6.137094000

C	6.498855000	4.437384000	5.318806000
C	9.217669000	3.056876000	5.158215000
C	8.910883000	5.580493000	6.757576000
C	9.377978000	5.717185000	0.863457000
C	6.487354000	6.207569000	1.871837000
C	7.792082000	3.460237000	1.959757000
C	12.217638000	6.103247000	6.077870000
C	12.157006000	4.822660000	3.840103000
C	12.065540000	7.489742000	6.492064000
C	11.739340000	9.746693000	5.290066000
H	12.027132000	7.124871000	2.073787000
H	13.388524000	7.760309000	3.010684000
H	12.011056000	8.802686000	2.624431000
H	7.437830000	9.983613000	0.864856000
H	7.832950000	8.342746000	0.328832000
H	8.510082000	9.752666000	-0.520367000
H	10.527724000	11.011393000	0.535843000
H	10.859350000	10.762253000	2.264975000
H	9.340999000	11.508988000	1.729000000
H	8.028139000	9.597001000	8.064143000
H	9.477423000	9.862543000	7.067276000
H	8.967777000	8.217982000	7.457950000
H	6.634197000	7.373091000	6.346091000
H	5.831575000	8.478186000	5.224553000
H	5.854174000	8.840935000	6.967534000
H	5.943726000	5.380286000	5.259568000
H	6.274725000	3.977424000	6.289190000
H	6.101527000	3.773072000	4.544897000
H	9.034633000	2.470396000	4.254200000
H	8.859845000	2.470095000	6.013621000
H	10.299976000	3.178747000	5.270982000
H	9.790665000	6.223079000	6.628802000
H	9.199958000	4.803564000	7.475595000
H	8.111723000	6.163573000	7.222092000
H	9.717455000	6.755006000	0.884378000
H	8.960040000	5.528190000	-0.132742000
H	10.261558000	5.078154000	0.978362000
H	5.703170000	5.819493000	2.532730000
H	6.144321000	6.082874000	0.837706000
H	6.585092000	7.279726000	2.074883000
H	8.726037000	2.889045000	1.994121000
H	7.359439000	3.319316000	0.961211000
H	7.097346000	3.019251000	2.679854000
C	12.545939000	5.002893000	7.036989000
H	12.897227000	5.011573000	3.050553000
H	11.212844000	4.562943000	3.343906000
H	12.495015000	3.943047000	4.394604000
C	12.199669000	7.929639000	7.916244000
H	12.588390000	10.121479000	4.700417000
H	11.798999000	10.221417000	6.273542000
H	10.826667000	10.111510000	4.800902000
H	11.795051000	4.925703000	7.834594000
H	13.510631000	5.178656000	7.530333000
H	12.599884000	4.029930000	6.541651000
H	11.461241000	7.431696000	8.560122000
H	12.058476000	9.007881000	8.027764000
H	13.188172000	7.678803000	8.321522000

104
TS2'B

Cd	10.426795000	14.551313000	16.022506000
C	11.150109000	16.037356000	14.387572000
Al	9.440934000	16.805155000	13.491322000
N	12.246434000	15.708512000	15.094939000
N	10.868079000	15.610024000	13.141681000
N	9.955993000	13.268120000	17.572005000
N	7.873384000	15.995906000	14.017158000
C	9.208605000	18.134978000	11.718120000
C	12.537116000	16.553207000	16.270685000
C	11.782536000	14.877581000	12.238915000
Si	10.090001000	11.562092000	17.277980000
Si	9.662259000	13.989712000	19.124335000
Si	6.895341000	16.496777000	15.370729000
Si	7.396115000	14.580484000	13.112821000
C	7.856101000	18.544334000	11.777615000
C	9.893721000	18.769373000	12.853705000
C	9.932939000	17.621301000	10.503551000
H	11.609916000	17.038875000	16.645401000
C	13.170371000	15.713123000	17.377242000
C	13.496810000	17.664947000	15.841832000
C	13.121681000	15.591008000	12.032564000
H	11.269586000	14.838438000	11.267334000
C	11.998251000	13.438717000	12.710268000
C	11.141557000	11.269634000	15.740831000
C	10.936219000	10.670670000	18.709249000
C	8.414647000	10.734420000	17.035760000
C	9.483185000	15.853080000	18.909654000
C	11.066878000	13.740886000	20.361296000
C	8.089752000	13.306065000	19.904997000
C	7.773622000	17.863471000	16.320323000
C	5.201100000	17.159552000	14.865757000
C	6.565958000	15.064526000	16.563186000
C	8.024531000	14.617977000	11.340916000
C	8.123884000	13.056269000	13.949667000
C	5.529328000	14.351205000	12.996116000
C	7.683625000	19.415798000	12.880573000
C	6.764873000	18.117307000	10.847701000
C	11.363751000	19.092993000	12.869057000
C	8.914435000	19.566796000	13.529126000
H	10.866608000	17.117339000	10.768332000
H	10.196761000	18.454149000	9.836194000
H	9.332492000	16.913672000	9.925117000
H	14.092190000	15.250033000	17.008967000
H	12.508107000	14.904915000	17.713569000
H	13.411591000	16.331226000	18.248184000
H	13.747539000	18.309497000	16.692021000
H	9.839637000	20.030077000	15.430753000
H	14.422004000	17.227690000	15.449985000
H	12.979379000	16.603237000	11.642507000
H	13.720913000	15.035402000	11.302692000
H	13.678633000	15.652499000	12.970594000
H	11.047880000	12.899902000	12.771089000
H	12.485729000	13.424305000	13.690003000
H	12.639744000	12.901449000	12.003174000
H	10.659325000	11.633176000	14.826494000
H	11.315242000	10.196528000	15.596447000
H	12.124155000	11.750485000	15.826634000
H	11.927015000	11.087026000	18.919944000
H	11.062498000	9.612439000	18.450018000
H	10.348874000	10.709449000	19.633059000
H	7.743082000	10.963058000	17.870965000
H	8.523335000	9.644366000	16.982814000
H	7.924199000	11.065413000	16.115192000
H	10.410255000	16.313223000	18.541834000
H	9.261549000	16.326853000	19.873432000
H	8.670924000	16.117813000	18.226570000
H	11.152355000	12.705335000	20.702560000
H	10.887206000	14.365165000	21.245531000
H	12.034903000	14.034926000	19.940443000
H	7.222206000	13.461080000	19.254135000
H	7.887647000	13.793677000	20.866116000
H	8.175070000	12.229951000	20.096047000
H	8.851351000	17.695529000	16.434454000
H	7.344989000	17.964777000	17.324363000
H	7.644512000	18.818554000	15.806950000
H	5.247943000	17.664952000	13.896803000
H	4.843762000	17.888095000	15.603713000
H	4.450205000	16.366790000	14.796553000
H	5.924187000	14.296110000	16.117822000
H	6.044474000	15.442718000	17.451881000
H	7.478085000	14.561515000	16.905227000
H	7.552104000	15.421061000	10.767642000
H	7.773381000	13.667758000	10.853781000
H	9.108566000	14.744993000	11.274052000
H	9.219049000	13.120364000	13.910167000
H	7.839684000	12.112032000	13.470902000
H	7.821260000	13.009860000	15.000697000
H	5.049439000	14.161133000	13.960463000
H	5.326064000	13.485792000	12.353524000

H	5.043532000	15.222221000	12.543822000
C	6.407888000	20.102674000	13.269649000
H	6.191681000	18.980973000	10.490859000
H	6.051716000	17.437093000	11.334382000
H	7.163561000	17.606092000	9.967255000
H	11.998596000	18.216578000	12.712015000
H	11.649980000	19.536295000	13.826726000
H	11.601730000	19.822148000	12.081763000
C	9.226999000	20.502151000	14.654136000
H	6.468710000	21.182197000	13.079893000
H	6.169535000	19.977114000	14.332219000
H	5.555167000	19.721108000	12.701536000
H	8.319502000	20.884010000	15.130961000
H	9.788397000	21.372178000	14.285680000
H	13.048647000	18.277693000	15.056671000

104
IM3

Cd	7.556231000	11.472086000	2.692982000
Si	4.673439000	12.821095000	2.375977000
N	6.270839000	13.007844000	3.017742000
C	9.147408000	8.942865000	3.235046000
C	4.495692000	11.061919000	1.710247000
C	4.349395000	14.014468000	0.956777000
C	3.360814000	13.092113000	3.699496000
Si	6.880225000	14.232334000	4.079337000
Al	9.780333000	7.286260000	4.107707000
N	8.853505000	9.899667000	2.361584000
N	8.827574000	8.870509000	4.527480000
H	5.225874000	10.836679000	0.920725000
H	3.504081000	10.916020000	1.265370000
H	4.604131000	10.308846000	2.501161000
H	4.416649000	15.056493000	1.287819000
H	3.349330000	13.863776000	0.533179000
H	5.080608000	13.876043000	0.152094000
H	3.485752000	12.387412000	4.529314000
H	2.354964000	12.952142000	3.286430000
H	3.405350000	14.105990000	4.113471000
C	6.242930000	15.936044000	3.598080000
C	8.766255000	14.231473000	3.962634000
C	6.420405000	13.893095000	5.874682000
N	8.760985000	5.778173000	3.899027000
C	11.790340000	7.157646000	4.382677000
C	9.461061000	9.837774000	1.020330000
C	8.071162000	9.840994000	5.313978000
H	6.531394000	16.192070000	2.572680000
H	6.653285000	16.702272000	4.266235000
H	5.150795000	15.997460000	3.663643000
H	9.210114000	13.290468000	4.318996000
H	9.196546000	15.027197000	4.582334000
H	9.105488000	14.397480000	2.932708000
H	5.333286000	13.857536000	6.006564000
H	6.815903000	14.664237000	6.546353000
H	6.824903000	12.927500000	6.202872000
Si	8.129618000	4.976610000	5.302939000
Si	8.264961000	5.368197000	2.288912000
C	11.954399000	5.798654000	4.942470000
C	11.895066000	8.063502000	5.552608000
C	12.537678000	7.532065000	3.106682000
H	10.156635000	8.988047000	1.030268000
C	8.399576000	9.602718000	-0.050260000
C	10.259101000	11.110285000	0.745112000
H	8.259624000	10.860245000	4.907399000
C	8.570370000	9.868393000	6.754275000
C	6.571752000	9.543232000	5.231255000
C	6.252489000	4.777819000	5.220663000
C	8.899721000	3.282619000	5.583001000
C	8.473782000	6.033579000	6.826608000
C	9.635525000	5.795176000	1.061983000
C	6.704597000	6.312458000	1.800498000
C	7.939340000	3.523807000	2.066592000
C	12.004321000	5.894904000	6.311315000
C	12.049769000	4.570858000	4.096712000
C	11.962009000	7.295938000	6.690143000
C	12.043276000	9.545438000	5.425488000
H	12.319136000	6.841140000	2.285153000
H	13.623566000	7.513852000	3.277393000
H	12.269616000	8.538243000	2.764338000
H	7.691321000	10.444504000	-0.082836000
H	7.834882000	8.688147000	0.152660000
H	8.855378000	9.521699000	-1.042766000
H	10.760726000	11.053336000	-0.226587000
H	11.013094000	11.274412000	1.521258000
H	9.597238000	11.989832000	0.718379000
H	7.994584000	10.589249000	7.344400000
H	9.626178000	10.147620000	6.790897000
H	8.469349000	8.881741000	7.214958000
H	6.357582000	8.591117000	5.728176000
H	6.239899000	9.439555000	4.190548000
H	5.981307000	10.332700000	5.709760000
H	5.767013000	5.754316000	5.106590000
H	5.880938000	4.326257000	6.148728000
H	5.919715000	4.143116000	4.393052000
H	8.731655000	2.606519000	4.738505000
H	8.480107000	2.810848000	6.479766000
H	9.980620000	3.379626000	5.727199000
H	9.491104000	6.447623000	6.842767000
H	8.356717000	5.431606000	7.735736000
H	7.761979000	6.863070000	6.888911000
H	9.959240000	6.838403000	1.140956000
H	9.290645000	5.634216000	0.033330000
H	10.514575000	5.159701000	1.221135000
H	5.851680000	5.996325000	2.411822000
H	6.439796000	6.156684000	0.747702000
H	6.843968000	7.388196000	1.963839000
H	8.848759000	2.939818000	2.248072000
H	7.627544000	3.335602000	1.031825000
H	7.153145000	3.133131000	2.720088000
C	12.063610000	4.785514000	7.314114000
H	12.843327000	4.668323000	3.343825000
H	11.111573000	4.374310000	3.560951000
H	12.277199000	3.685193000	4.696263000
C	11.984482000	7.756205000	8.114264000
H	13.011640000	9.798920000	4.970843000
H	12.006253000	10.043308000	6.398740000
H	11.268856000	9.990595000	4.787656000
H	11.148331000	4.736637000	7.920405000
H	12.897881000	4.925290000	8.012969000
H	12.191445000	3.809826000	6.838076000
H	11.164237000	7.309824000	8.692874000
H	11.891518000	8.842665000	8.195097000
H	12.916596000	7.465181000	8.615850000

104
TS3

Cd	-0.526335000	1.786625000	-1.217775000
Si	-3.173991000	2.561578000	-2.962566000
N	-2.291397000	2.813306000	-1.498107000
C	1.845778000	1.723994000	0.236165000
C	-2.441418000	1.084523000	-3.878094000
C	-3.071204000	4.043373000	-4.123501000
C	-4.990590000	2.190079000	-2.621409000
Si	-2.737061000	3.849725000	-0.185284000
Al	1.095272000	-0.831282000	0.443173000
N	1.584440000	1.471349000	-1.048395000
N	1.054951000	0.964881000	1.052142000
H	-1.390900000	1.241926000	-4.156793000
H	-2.989448000	0.894402000	-4.808554000
H	-2.499922000	0.175250000	-3.271113000
H	-3.556876000	4.929451000	-3.702335000
H	-3.556480000	3.821293000	-5.081636000
H	-2.026242000	4.303960000	-4.328716000
H	-5.093109000	1.315115000	-1.969249000
H	-5.531158000	1.984514000	-3.553006000
H	-5.494452000	3.029882000	-2.129905000
C	-3.592175000	5.418759000	-0.785675000

C	-1.173465000	4.368391000	0.733909000
C	-3.896097000	2.985650000	1.024748000
N	-0.382297000	-1.890837000	0.678559000
C	2.834216000	-1.717152000	-0.162900000
C	2.404414000	2.131021000	-2.089595000
C	0.901873000	1.462143000	2.445389000
H	-2.940710000	5.996469000	-1.450298000
H	-3.847340000	6.054320000	0.070967000
H	-4.523031000	5.209806000	-1.323988000
H	-0.615922000	3.514605000	1.138976000
H	-1.413579000	5.017322000	1.584649000
H	-0.494005000	4.922376000	0.074659000
H	-4.845549000	2.733487000	0.538378000
H	-4.121020000	3.619996000	1.890522000
H	-3.466214000	2.048663000	1.397902000
Si	-0.694368000	-2.833824000	2.123291000
Si	-1.483762000	-2.007863000	-0.671378000
C	2.611480000	-3.153182000	0.164369000
C	3.697373000	-1.221283000	0.956172000
C	3.313172000	-1.414915000	-1.579598000
H	3.457883000	1.883439000	-1.893270000
C	2.017228000	1.591478000	-3.460141000
C	2.243638000	3.648749000	-2.028406000
H	0.979728000	2.556173000	2.387192000
C	2.021202000	0.979328000	3.363796000
C	-0.491181000	1.111922000	2.954853000
C	-2.436298000	-2.509249000	2.771860000
C	-0.524030000	-4.692905000	1.869561000
C	0.525194000	-2.411530000	3.498938000
C	-0.533265000	-1.682255000	-2.277303000
C	-2.944847000	-0.827770000	-0.502790000
C	-2.259117000	-3.708145000	-0.917051000
C	3.158653000	-3.417731000	1.391107000
C	1.929797000	-4.112794000	-0.753143000
C	3.809123000	-2.218090000	1.892118000
C	4.467822000	0.053997000	0.901458000
H	2.576525000	-1.702444000	-2.336526000
H	4.240538000	-1.968002000	-1.784257000
H	3.520544000	-0.351153000	-1.710431000
H	0.977349000	1.858434000	-3.700336000
H	2.107388000	0.502447000	-3.507912000
H	2.650881000	2.029613000	-4.237454000
H	2.879313000	4.136828000	-2.775178000
H	2.504413000	4.015089000	-1.032039000
H	1.201323000	3.932321000	-2.239402000
H	1.881592000	1.374601000	4.376329000
H	2.985932000	1.326795000	2.984819000
H	2.053950000	-0.112600000	3.424683000
H	-0.658270000	0.032413000	2.950316000
H	-1.261272000	1.562974000	2.319850000
H	-0.629801000	1.476918000	3.977873000
H	-2.624409000	-1.439505000	2.916489000
H	-2.572497000	-3.007850000	3.739221000
H	-3.209254000	-2.895341000	2.098162000
H	-1.266544000	-5.113439000	1.187513000
H	-0.643041000	-5.193753000	2.838433000
H	0.469354000	-4.943508000	1.488381000
H	1.550116000	-2.306421000	3.123414000
H	0.532300000	-3.246927000	4.209599000
H	0.267537000	-1.512070000	4.063990000
H	0.004946000	-0.726567000	-2.331959000
H	-1.221278000	-1.708498000	-3.130054000
H	0.209265000	-2.473285000	-2.433139000
H	-3.505318000	-1.069565000	0.407679000
H	-3.632321000	-0.935343000	-1.350927000
H	-2.676783000	0.233128000	-0.434822000
H	-1.517469000	-4.496388000	-1.080202000
H	-2.890245000	-3.656645000	-1.813330000
H	-2.904447000	-4.010600000	-0.086492000
C	3.172631000	-4.709818000	2.145349000
H	2.290581000	-4.004553000	-1.783743000
H	0.842498000	-3.965588000	-0.769115000
H	2.111530000	-5.148700000	-0.452971000
C	4.523456000	-2.164120000	3.205818000
H	5.214520000	0.004856000	0.095929000
H	5.009447000	0.234341000	1.834040000
H	3.838600000	0.934647000	0.699396000
H	2.570218000	-4.654668000	3.062226000
H	4.192832000	-4.967649000	2.454644000
H	2.786588000	-5.540506000	1.549394000
H	3.884453000	-2.521902000	4.024017000
H	4.840691000	-1.148623000	3.455502000
H	5.417250000	-2.801884000	3.204346000

127
7a

Cd	6.913357000	19.456250000	6.542659000
N	5.596306000	19.705473000	4.756177000
C	8.862400000	19.294731000	7.409230000
N	4.709416000	19.542451000	6.801830000
C	4.484372000	19.690287000	5.493382000
C	5.673415000	20.086215000	3.359415000
Al	10.970817000	19.120194000	8.491180000
N	9.523857000	20.311087000	7.974180000
N	9.559471000	18.157872000	7.519797000
C	3.705134000	19.225063000	7.798789000
N	3.182693000	19.823866000	4.941479000
C	6.198586000	18.913545000	2.524044000
H	4.670931000	20.364207000	2.994957000
C	6.604586000	21.292306000	3.199571000
N	11.047899000	18.780154000	10.296161000
C	12.526673000	19.312568000	7.138383000
C	13.265380000	18.165066000	7.654083000
C	8.978966000	21.663045000	8.080169000
C	9.161527000	16.920089000	6.842537000
C	4.049710000	17.894408000	8.475902000
C	3.631712000	20.336729000	8.850228000
H	2.721323000	19.127086000	7.311300000
Si	2.473316000	18.421010000	4.132333000
Si	2.371386000	21.387383000	5.107387000
H	7.212614000	18.650137000	2.848393000
H	6.238297000	19.172410000	1.459843000
H	5.568660000	18.027119000	2.644251000
H	6.251832000	22.146047000	3.785758000
H	6.679480000	21.597088000	2.149862000
H	7.611973000	21.034298000	3.550470000
Si	11.270313000	20.022617000	11.496935000
Si	10.579232000	17.201365000	10.865853000
C	12.043489000	19.398941000	5.708334000
C	13.064310000	20.479113000	7.831068000
C	13.213556000	16.799177000	7.041998000
C	14.112139000	18.614011000	8.661896000
H	9.787146000	22.269832000	8.503488000
C	8.630949000	22.291983000	6.725633000
C	7.795870000	21.736873000	9.051034000
H	10.039733000	16.264183000	6.887772000
C	8.833096000	17.092527000	5.351951000
C	8.020728000	16.198552000	7.564394000
H	5.013746000	17.976558000	8.994423000
H	4.127096000	17.086299000	7.742396000
H	3.291573000	17.621284000	9.218264000
H	2.856582000	20.124418000	9.595302000
H	3.418473000	21.306591000	8.391196000
H	4.591587000	20.418332000	9.374315000
C	2.490808000	18.542332000	2.254952000
C	3.408741000	16.890270000	4.683098000
C	0.676324000	18.251757000	4.660603000
C	1.011798000	21.391788000	6.406770000
C	1.582184000	21.851133000	3.464340000
C	3.667761000	22.676003000	5.530235000
C	9.854639000	20.073111000	12.753843000
C	12.868317000	19.811020000	12.477821000
C	11.274199000	21.749663000	10.739577000
C	11.168595000	15.850907000	9.694027000
C	11.368959000	16.709416000	12.510786000
C	8.705105000	17.098042000	11.097712000
H	11.681615000	18.430401000	5.352451000
H	12.863246000	19.708647000	5.044873000
H	11.225884000	20.119467000	5.586864000

C	13.997259000	20.038170000	8.762597000
C	12.802372000	21.891540000	7.409844000
H	12.217686000	16.558341000	6.655692000
H	13.485470000	16.010415000	7.747898000
H	13.904829000	16.730936000	6.189406000
C	14.963314000	17.760867000	9.551183000
H	9.458236000	22.182961000	6.017188000
H	8.417143000	23.358752000	6.852121000
H	7.736510000	21.849068000	6.265308000
H	6.913645000	21.222047000	8.646854000
H	7.509633000	22.777565000	9.240137000
H	8.049877000	21.260771000	10.003148000
H	7.821836000	17.487275000	5.182598000
H	8.866037000	16.118454000	4.851961000
H	9.544594000	17.762125000	4.860671000
H	8.300101000	15.955001000	8.591829000
H	7.756712000	15.272568000	7.041421000
H	7.120524000	16.828456000	7.602604000
H	1.951474000	19.426344000	1.900283000
H	1.987957000	17.661866000	1.835568000
H	3.500709000	18.575462000	1.836778000
H	4.494732000	17.023699000	4.638705000
H	3.146622000	16.043398000	4.038469000
H	3.143427000	16.619016000	5.710304000
H	0.582511000	18.188784000	5.750001000
H	0.263119000	17.329183000	4.235247000
H	0.047294000	19.077185000	4.311527000
H	0.213600000	20.683242000	6.164607000
H	0.560699000	22.390948000	6.451607000
H	1.380961000	21.151395000	7.407443000
H	2.319880000	21.856386000	2.654666000
H	1.162498000	22.861528000	3.540831000
H	0.763762000	21.183849000	3.174869000
H	4.320197000	22.361127000	6.351347000
H	3.179142000	23.611403000	5.826085000
H	4.298656000	22.891035000	4.661594000
H	8.886461000	20.185263000	12.252096000
H	9.990397000	20.941067000	13.411629000
H	9.797870000	19.187368000	13.393492000
H	12.791037000	19.009636000	13.218248000
H	13.110020000	20.735973000	13.015352000
H	13.708686000	19.577647000	11.818622000
H	11.797302000	21.797102000	9.781156000
H	11.771461000	22.445427000	11.425619000
H	10.251049000	22.111568000	10.598606000
H	10.927224000	16.027242000	8.644624000
H	10.731651000	14.886404000	9.979990000
H	12.258118000	15.760953000	9.768067000
H	12.462348000	16.716220000	12.441413000
H	11.059731000	15.680679000	12.736319000
H	11.077740000	17.329243000	13.362949000
H	8.401001000	17.632649000	12.004236000
H	8.358877000	16.061690000	11.194106000
H	8.181176000	17.559359000	10.253333000
C	14.794893000	20.920330000	9.677722000
H	13.124957000	22.612161000	8.168166000
H	11.741841000	22.073588000	7.200494000
H	13.349985000	22.127762000	6.485245000
H	15.999121000	18.119005000	9.588759000
H	14.992128000	16.724770000	9.202478000
H	14.587550000	17.748413000	10.583618000
H	15.425190000	20.334604000	10.352881000
H	14.158744000	21.559689000	10.301468000
H	15.459083000	21.583885000	9.108607000

34
$B(C_6F_5)_3$

B	-4.243373000	-0.330018000	0.520128000
F	-0.566371000	2.577511000	2.140677000
C	-0.989768000	1.648859000	1.282658000
C	-2.293658000	1.181613000	1.319672000
F	-3.096898000	1.699955000	2.259468000
C	-2.773656000	0.191454000	0.454370000
C	-1.849968000	-0.299307000	-0.475745000
F	-2.220360000	-1.233859000	-1.362659000
C	-0.545540000	0.160675000	-0.558966000
F	0.296666000	-0.320294000	-1.473985000
C	-0.113326000	1.137578000	0.331478000
F	1.136772000	1.581909000	0.273860000
F	-6.589797000	-0.946631000	2.128261000
F	-8.573381000	0.768970000	2.674865000
C	-6.513283000	0.287904000	1.611788000
C	-7.549245000	1.156996000	1.914328000
C	-5.408591000	0.658763000	0.835789000
C	-7.515679000	2.453133000	1.411353000
F	-8.505956000	3.295664000	1.681012000
C	-5.416517000	1.976656000	0.363182000
C	-6.445605000	2.866601000	0.625197000
F	-4.414835000	2.420731000	-0.408866000
F	-6.422416000	4.107626000	0.138346000
F	-6.557597000	-1.370520000	-0.903044000
C	-5.687060000	-2.268090000	-0.419834000
F	-7.049891000	-3.965326000	-1.349769000
C	-5.962290000	-3.603086000	-0.668791000
C	-4.545247000	-1.840129000	0.267558000
C	-5.085662000	-4.575134000	-0.198865000
C	-3.689012000	-2.855553000	0.709420000
F	-5.338838000	-5.859966000	-0.419570000
C	-3.943499000	-4.201622000	0.501201000
F	-2.580478000	-2.548533000	1.397757000
F	-3.110964000	-5.137119000	0.958977000

55
Zn{N(TMS)$_2$}$_2$

Zn	-1.674675000	4.900424000	3.512561000
N	-0.126125000	3.930808000	3.463681000
N	-3.222139000	5.872411000	3.561934000
Si	0.002096000	2.785372000	2.170207000
Si	1.018141000	4.287765000	4.714899000
Si	-3.201757000	7.349805000	2.657420000
Si	-4.493632000	5.213227000	4.536505000
C	1.091214000	3.426951000	0.775128000
C	-1.731318000	2.495645000	1.478623000
C	0.701986000	1.144553000	2.771568000
C	2.763760000	4.438163000	4.026409000
C	0.523752000	5.934629000	5.496737000
C	1.010060000	2.973914000	6.062713000
C	-4.809484000	7.598444000	1.710845000
C	-1.770610000	7.256940000	1.428324000
C	-2.916634000	8.844455000	3.766338000
C	-5.845281000	4.428624000	3.487402000
C	-3.747175000	3.865521000	5.629143000
C	-5.272669000	6.530193000	5.633616000
H	0.709056000	4.378150000	0.386246000
H	1.133240000	2.715358000	-0.058082000
H	2.117325000	3.600057000	1.117814000
H	-2.409302000	2.101707000	2.246168000
H	-1.708791000	1.768412000	0.658421000
H	-2.178048000	3.414442000	1.074353000
H	0.082370000	0.716087000	3.567111000
H	0.749471000	0.418995000	1.951122000
H	2.817851000	5.212904000	3.253556000
H	3.475035000	4.698982000	4.818768000
H	3.103514000	3.496761000	3.579286000
H	-0.476510000	5.895682000	5.949659000
H	1.220924000	6.213787000	6.295572000
H	0.528220000	6.748282000	4.760491000
H	0.007762000	2.864891000	6.493469000
H	1.699832000	3.230930000	6.875419000
H	-5.006723000	6.759016000	1.034933000
H	-4.768536000	8.514795000	1.110410000
H	-0.799310000	7.147166000	1.930622000
H	-1.712074000	8.171745000	0.826736000
H	-1.886611000	6.415456000	0.733884000
H	-3.724163000	8.962210000	4.497206000
H	-2.857424000	9.770203000	3.181682000

H	-1.979471000	8.739373000	4.325671000
H	-5.432706000	3.643423000	2.843065000
H	-6.622010000	3.975484000	4.114888000
H	-2.963788000	4.261842000	6.287444000
H	-4.513574000	3.413958000	6.270230000
H	-3.309596000	3.049454000	5.037821000
H	-5.735311000	7.326429000	5.039042000
H	-6.056859000	6.097525000	6.265771000
H	-4.526279000	6.994003000	6.288075000
H	-6.329459000	5.166884000	2.838868000
H	-5.666354000	7.691021000	2.388167000
H	1.307705000	1.995822000	5.669299000
H	1.718715000	1.259129000	3.165042000

57
$Cd(TMP)_2$

Cd	4.690291000	8.550328000	1.423823000
N	2.800517000	7.791346000	1.615906000
N	6.564819000	9.267469000	1.038019000
C	1.775345000	8.428718000	0.776328000
C	2.446872000	7.269771000	2.943399000
C	7.511600000	8.313792000	0.442255000
C	7.053177000	10.344255000	1.909411000
C	0.525441000	7.525447000	0.712539000
C	1.371295000	9.856789000	1.210723000
C	2.352267000	8.536934000	-0.642688000
C	1.188751000	6.383025000	2.827323000
C	3.608747000	6.381190000	3.408662000
C	2.231652000	8.346898000	4.031760000
C	8.666901000	9.089140000	-0.225569000
C	6.760201000	7.548039000	-0.655347000
C	8.079508000	7.261324000	1.421978000
C	8.221873000	11.076719000	1.214902000
C	5.906413000	11.350038000	2.083347000
C	7.478689000	9.895211000	3.326665000
H	-0.274095000	8.052226000	0.175064000
H	0.779565000	6.631352000	0.126150000
C	0.050837000	7.083753000	2.092940000
H	0.801856000	9.873578000	2.142006000
H	0.756411000	10.337499000	0.439806000
H	2.270084000	10.469136000	1.358893000
H	3.179350000	9.263838000	-0.684657000
H	1.590736000	8.885040000	-1.349167000
H	2.726990000	7.563391000	-0.975804000
H	1.461886000	5.476058000	2.270037000
H	0.871378000	6.069199000	3.830434000
H	4.511430000	6.980464000	3.607653000
H	3.847413000	5.636962000	2.641616000
H	3.359169000	5.863818000	4.341583000
H	1.327912000	8.936425000	3.867866000
H	3.083198000	9.039269000	4.045105000
H	2.150375000	7.886087000	5.023946000
H	9.421560000	8.377951000	-0.586522000
H	8.261549000	9.612241000	-1.102885000
C	9.294630000	10.117973000	0.708772000
H	6.281560000	8.247398000	-1.349242000
H	5.984801000	6.894311000	-0.224597000
H	7.441807000	6.901081000	-1.218146000
H	8.759619000	7.694207000	2.157529000
H	8.631683000	6.484054000	0.879497000
H	7.259598000	6.779098000	1.968927000
H	7.812749000	11.630102000	0.358195000
H	8.651440000	11.813081000	1.906945000
H	5.083224000	10.916364000	2.673247000
H	5.514550000	11.653154000	1.106528000
H	6.246690000	12.241791000	2.621136000
H	8.401072000	9.311375000	3.329578000
H	6.690006000	9.276494000	3.773155000
H	7.638378000	10.764954000	3.975811000
H	-0.804919000	6.404291000	1.993386000
H	-0.309084000	7.943876000	2.672786000
H	10.074273000	10.678490000	0.177753000
H	9.796691000	9.622056000	1.549996000

68
*pre*5

Ag	10.123197000	2.117951000	3.437585000
Si	7.573562000	2.377429000	1.499209000
P	12.430359000	2.244210000	3.856715000
Si	7.097865000	2.048843000	4.512205000
N	8.082688000	2.138509000	3.115570000
C	6.071395000	1.319758000	1.046107000
C	8.957761000	1.916462000	0.293236000
C	7.127501000	4.179058000	1.130649000
C	12.790461000	1.588149000	5.627738000
C	13.429650000	1.218810000	2.574007000
C	12.929316000	4.098588000	3.733297000
C	5.661908000	3.281509000	4.478095000
C	6.355504000	0.328383000	4.763434000
C	8.109316000	2.438833000	6.066571000
H	6.292578000	0.253851000	1.174221000
H	5.776892000	1.481355000	0.002123000
H	5.202793000	1.554524000	1.671977000
H	9.832591000	2.566141000	0.426720000
H	8.626652000	2.021259000	-0.747228000
H	9.280263000	0.878527000	0.440583000
H	6.281636000	4.513416000	1.741554000
H	6.858628000	4.322996000	0.076932000
H	7.975599000	4.837451000	1.355490000
C	14.268588000	1.314491000	5.935700000
C	11.972271000	0.299208000	5.842732000
C	12.249594000	2.600491000	6.651593000
C	14.923692000	1.560752000	2.488235000
C	13.282451000	-0.277136000	2.900030000
C	12.774627000	1.402328000	1.191707000
C	12.929982000	4.514509000	2.252334000
C	14.289706000	4.451711000	4.349392000
C	11.828591000	4.948549000	4.399578000
H	6.030545000	4.311449000	4.409101000
H	5.055336000	3.199762000	5.388201000
H	4.996868000	3.109382000	3.624126000
H	5.697079000	0.060498000	3.929228000
H	5.768496000	0.267189000	5.687984000
H	7.146948000	-0.428951000	4.812661000
H	8.888307000	1.684941000	6.237607000
H	7.471437000	2.457253000	6.958898000
H	8.596625000	3.418304000	5.981121000
H	14.677090000	0.500122000	5.333789000
H	14.357235000	1.014047000	6.987686000
H	14.896842000	2.196761000	5.792552000
H	10.910356000	0.464341000	5.631168000
H	12.065580000	0.006307000	6.896002000
H	12.312358000	-0.540595000	5.238727000
H	12.842811000	3.515402000	6.700625000
H	12.292140000	2.133955000	7.643416000
H	11.203415000	2.857348000	6.455481000
H	15.101084000	2.565189000	2.097290000
H	15.403740000	0.857584000	1.795501000
H	15.432439000	1.472475000	3.451318000
H	13.830682000	-0.577729000	3.794476000
H	13.696502000	-0.846985000	2.059221000
H	12.231509000	-0.566002000	3.007346000
H	11.725656000	1.091278000	1.210956000
H	13.303828000	0.763157000	0.473520000
H	12.817350000	2.423124000	0.815343000
H	13.754836000	4.080468000	1.685112000
H	13.042701000	5.604636000	2.207018000
H	11.983906000	4.262545000	1.760532000
H	14.300868000	4.327458000	5.434637000
H	14.502224000	5.509224000	4.146285000
H	15.108631000	3.865184000	3.926418000
H	10.848706000	4.752073000	3.949594000
H	12.070335000	6.006584000	4.236908000
H	11.744482000	4.793364000	5.474032000

81
1^{Zn}

Zn	-1.401826000	4.886189000	3.755300000
Al	-3.462665000	6.110915000	3.842639000
N	0.242267000	3.997830000	3.597173000
N	-5.138681000	5.379547000	3.700723000
C	-3.316738000	8.125024000	4.564125000
C	-3.246280000	8.152067000	3.106633000
Si	0.793700000	3.666470000	1.995332000
Si	1.051726000	3.641254000	5.080119000
Si	-5.527750000	4.604868000	2.186605000
Si	-6.013958000	5.042101000	5.168028000
C	-1.968331000	8.013078000	5.043882000
C	-4.463233000	8.713764000	5.338565000
C	-1.879156000	8.005993000	2.754135000
C	-4.359880000	8.655448000	2.233093000
C	2.067523000	4.928554000	1.405552000
C	-0.677254000	3.762705000	0.812968000
C	1.571441000	1.956018000	1.846765000
C	2.853366000	4.198386000	5.053428000
C	0.169627000	4.565927000	6.472783000
C	1.009933000	1.805138000	5.502307000
C	-5.066585000	2.776836000	2.167296000
C	-4.539066000	5.403985000	0.791318000
C	-7.354628000	4.791417000	1.769522000
C	-6.644671000	3.268897000	5.273583000
C	-4.837930000	5.261953000	6.631495000
C	-7.507365000	6.169271000	5.384282000
C	-1.102276000	7.937071000	3.940174000
C	-1.602168000	8.019477000	6.494658000
H	-4.410844000	9.811188000	5.309969000
H	-4.451847000	8.414351000	6.390259000
H	-5.429311000	8.422656000	4.917242000
C	-1.326372000	7.954630000	1.365074000
H	-4.517283000	9.728016000	2.409925000
H	-4.124796000	8.529513000	1.173529000
H	-5.310358000	8.147302000	2.432125000
H	1.643898000	5.939747000	1.385862000
H	2.418487000	4.695320000	0.392991000
H	2.942056000	4.953510000	2.065190000
H	-1.469908000	3.056619000	1.089147000
H	-0.368790000	3.529337000	-0.213083000
H	-1.116176000	4.768895000	0.790596000
H	0.872892000	1.171387000	2.157907000
H	1.867316000	1.754420000	0.810389000
H	2.932312000	5.269753000	4.835094000
H	3.337464000	4.013407000	6.019730000
H	3.429289000	3.660915000	4.290891000
H	-0.877369000	4.251920000	6.581516000
H	0.659921000	4.380316000	7.435867000
H	0.178439000	5.649182000	6.303708000
H	-0.022248000	1.437740000	5.536299000
H	1.467912000	1.614090000	6.480334000
H	-4.027622000	2.642624000	2.493251000
H	-5.150860000	2.371076000	1.151947000
H	-4.851961000	6.434449000	0.600431000
H	-4.696227000	4.833095000	-0.131780000
H	-3.457752000	5.402220000	0.979344000
H	-8.004922000	4.313035000	2.509843000
H	-7.572669000	4.333862000	0.797196000
H	-7.636065000	5.849254000	1.713551000
H	-5.823315000	2.546296000	5.221375000
H	-7.152301000	3.127983000	6.235739000
H	-4.349994000	6.242575000	6.655531000
H	-5.383065000	5.152361000	7.576716000
H	-4.051518000	4.497173000	6.616414000
H	-8.188014000	6.081664000	4.529764000
H	-8.069823000	5.897968000	6.285828000
H	-7.225209000	7.222900000	5.474162000
C	0.390795000	7.790545000	3.961943000
H	-2.036757000	7.169665000	7.034582000
H	-1.965260000	8.932942000	6.982284000
H	-0.520478000	7.976064000	6.638310000
H	-2.086773000	7.661799000	0.635397000
H	-0.938063000	8.936155000	1.061552000
H	-0.495362000	7.245046000	1.286583000
H	0.858382000	8.496198000	3.265578000
H	0.798535000	7.995995000	4.955375000
H	0.728260000	6.784523000	3.674999000
H	-7.365446000	3.020136000	4.487686000
H	-5.699965000	2.170456000	2.821081000
H	1.548454000	1.207371000	4.759353000
H	2.471424000	1.867768000	2.466338000

60
3

B	-3.448304000	-0.586850000	2.268821000
F	1.517444000	-0.876035000	2.018982000
C	0.416613000	-0.188592000	1.685071000
C	-0.840804000	-0.647411000	2.035281000
F	-0.885401000	-1.802345000	2.745659000
C	-2.037175000	0.011280000	1.745994000
C	-1.867332000	1.217285000	1.062949000
F	-2.927362000	1.980155000	0.734190000
C	-0.624706000	1.717814000	0.685543000
F	-0.531367000	2.877323000	0.025061000
C	0.528304000	1.010979000	0.993651000
F	1.728011000	1.478502000	0.639044000
F	-4.551876000	-0.526970000	-0.381477000
F	-6.789015000	0.742714000	-1.179588000
C	-5.244025000	0.177612000	0.529278000
C	-6.387039000	0.830930000	0.091739000
C	-4.772505000	0.246010000	1.839741000
C	-7.118796000	1.599107000	0.990799000
F	-8.221057000	2.236784000	0.591895000
C	-5.540564000	1.026096000	2.687635000
C	-6.692610000	1.698404000	2.307378000
F	-5.175943000	1.167078000	4.002131000
F	-7.388869000	2.430750000	3.186184000
F	-5.315323000	-2.201016000	3.740718000
C	-4.658721000	-2.857557000	2.740239000
F	-5.876677000	-4.797294000	3.360620000
C	-4.970677000	-4.197185000	2.577061000
C	-3.713217000	-2.165733000	1.985808000
C	-4.322509000	-4.916923000	1.581591000
C	-3.102542000	-2.929853000	0.991522000
F	-4.604264000	-6.207994000	1.390977000
C	-3.382721000	-4.276470000	0.785040000
F	-2.201398000	-2.377338000	0.163531000
F	-2.765736000	-4.960272000	-0.183807000
Al	-3.221266000	-0.492100000	4.389832000
C	-1.535918000	-0.154985000	5.755414000
C	-1.971108000	-1.511305000	5.892555000
C	-3.340691000	-1.497648000	6.321683000
C	-3.750578000	-0.130199000	6.464596000
C	-2.637546000	0.701844000	6.101527000
C	-2.603610000	2.198504000	6.140757000
C	-0.173524000	0.313512000	5.344758000
C	-1.159045000	-2.750990000	5.679346000
C	-4.158348000	-2.706075000	6.652982000
H	-1.851357000	2.599303000	5.456452000
H	-2.352566000	2.549017000	7.149115000
H	-3.568542000	2.634423000	5.868827000
C	-5.080402000	0.335582000	6.968425000
H	0.399329000	-0.484576000	4.867986000
H	0.392083000	0.655764000	6.219405000
H	-0.224357000	1.147897000	4.638211000
H	-0.227497000	-2.538186000	5.153742000
H	-1.703418000	-3.497823000	5.093024000
H	-0.908677000	-3.207119000	6.644460000
H	-3.912641000	-3.551839000	6.004752000
H	-5.228428000	-2.511179000	6.553785000
H	-3.968406000	-3.016809000	7.687731000
H	-5.900932000	-0.274823000	6.580859000
H	-5.276069000	1.372360000	6.687873000
H	-5.109372000	0.273741000	8.062970000

3
4

Cd	-1.396054000	4.855510000	3.763824000
Al	-3.583984000	6.138018000	3.772107000
N	0.479036000	3.948328000	3.609428000
N	-5.250744000	5.369772000	3.699314000
C	-3.492810000	8.193837000	4.445507000
C	-3.320732000	8.181430000	2.995351000
C	0.928143000	3.421836000	2.318459000
C	1.259035000	3.654512000	4.813981000
C	-5.780832000	4.866943000	2.411561000
C	-5.894996000	4.908007000	4.950533000
C	-2.187239000	8.101839000	5.018900000
C	-4.720671000	8.741919000	5.117245000
C	-1.923082000	8.085600000	2.745505000
C	-4.353901000	8.692751000	2.031850000
C	2.110019000	4.216607000	1.707992000
C	-0.233424000	3.546354000	1.323082000
C	1.301307000	1.926704000	2.424415000
C	2.546941000	4.506684000	4.943818000
C	0.390489000	3.998253000	6.030900000
C	1.620071000	2.154967000	4.890550000
C	-5.990961000	3.339422000	2.454352000
C	-4.740870000	5.153507000	1.317640000
C	-7.090485000	5.562700000	1.979087000
C	-6.093174000	3.378809000	4.937942000
C	-4.958295000	5.237543000	6.122779000
C	-7.240285000	5.608050000	5.245419000
C	-1.238795000	8.046356000	3.977545000
C	-1.894424000	8.064074000	6.486295000
H	-4.765415000	9.832103000	4.987903000
H	-4.725517000	8.544474000	6.193276000
H	-5.638631000	8.321486000	4.694748000
C	-1.279531000	8.012017000	1.397253000
H	-4.415205000	9.788323000	2.090473000
H	-4.106687000	8.437993000	0.996733000
H	-5.352379000	8.298711000	2.246802000
H	1.885215000	5.288577000	1.736544000
H	2.277791000	3.922020000	0.664688000
H	3.045923000	4.056669000	2.245865000
H	-1.114515000	3.001061000	1.687308000
H	0.039353000	3.133998000	0.345318000
H	-0.504796000	4.601586000	1.168349000
C	2.240526000	1.644319000	3.593326000
H	0.373793000	1.355292000	2.575919000
H	1.745977000	1.587039000	1.478687000
H	2.309854000	5.563422000	4.783613000
H	2.986042000	4.394961000	5.942932000
H	3.312465000	4.221731000	4.220436000
H	-0.539052000	3.413033000	6.021962000
H	0.916768000	3.783399000	6.967507000
H	0.138943000	5.069735000	6.035035000
H	0.694761000	1.591813000	5.081591000
H	2.294679000	1.977148000	5.739551000
C	-6.786851000	2.888589000	3.673014000
H	-5.000968000	2.860558000	2.485607000
H	-6.481314000	3.015807000	1.526148000
H	-4.565678000	6.229875000	1.201730000
H	-5.091196000	4.777350000	0.350682000
H	-3.785257000	4.657052000	1.534552000
H	-7.949040000	5.249682000	2.575040000
H	-7.318435000	5.327742000	0.932475000
H	-6.991961000	6.648339000	2.076406000
H	-5.101261000	2.906845000	4.997757000
H	-6.654014000	3.077852000	5.833284000
H	-4.803307000	6.318760000	6.220764000
H	-5.389660000	4.886691000	7.066296000
H	-3.982482000	4.746137000	6.006705000
H	-8.037322000	5.296297000	4.568988000
H	-7.570404000	5.377548000	6.265487000
H	-7.129240000	6.692774000	5.156396000
C	0.247108000	7.911142000	4.129401000
H	-2.765096000	7.732115000	7.059361000
H	-1.615553000	9.057831000	6.860975000
H	-1.068732000	7.384747000	6.722140000
H	-1.943776000	7.558103000	0.655018000
H	-1.012181000	9.011786000	1.028821000
H	-0.356362000	7.422387000	1.424649000
H	3.217487000	2.116201000	3.422412000
H	2.433002000	0.566488000	3.667768000
H	-7.815746000	3.268692000	3.625263000
H	-6.865947000	1.794566000	3.686680000
H	0.768321000	8.761818000	3.673148000
H	0.540099000	7.879082000	5.182704000
H	0.636208000	6.999474000	3.654342000

94
5

Ag	7.901089000	3.849614000	7.771539000
P	5.512350000	3.615917000	7.150755000
Al	10.327469000	3.775663000	8.180027000
C	4.504868000	2.937412000	8.641327000
C	4.803893000	5.311852000	6.600758000
C	5.424588000	2.369502000	5.688897000
N	11.458993000	3.436771000	6.729657000
C	11.211495000	3.283292000	10.131555000
C	11.542497000	4.657297000	9.796369000
C	9.924281000	3.301965000	10.763032000
C	10.432518000	5.457902000	10.172273000
C	3.133291000	2.340010000	8.302114000
C	5.373610000	1.878876000	9.353329000
C	4.311764000	4.067711000	9.666217000
C	5.332236000	6.381924000	7.578538000
C	5.406681000	5.675373000	5.232866000
C	3.275331000	5.398702000	6.507515000
C	5.690723000	0.954803000	6.231321000
C	4.106092000	2.358421000	4.904065000
C	6.597558000	2.666880000	4.730838000
Si	11.810929000	1.792567000	6.309029000
Si	11.600718000	4.718002000	5.565793000
C	12.242569000	2.202705000	10.288468000
C	12.927980000	5.105606000	9.436721000
C	9.453747000	4.621756000	10.772758000
C	9.221509000	2.079017000	11.263133000
C	10.284321000	6.935492000	9.993374000
H	2.478897000	3.058850000	7.802098000
H	2.639530000	2.034796000	9.234324000
H	3.210765000	1.450560000	7.673223000
H	5.509317000	0.965157000	8.776061000
H	4.879234000	1.603345000	10.293803000
H	6.366063000	2.277605000	9.595630000
H	5.264082000	4.537303000	9.933560000
H	3.893701000	3.628048000	10.580350000
H	3.613754000	4.836683000	9.330083000
H	4.941410000	6.284200000	8.590474000
H	5.034770000	7.369848000	7.204056000
H	6.427467000	6.356818000	7.632422000
H	6.499497000	5.598523000	5.240173000
H	5.150119000	6.719516000	5.015156000
H	5.011582000	5.067915000	4.416760000
H	2.856419000	4.642530000	5.838838000
H	2.999775000	6.383780000	6.108074000
H	2.790416000	5.300538000	7.481496000
H	6.612923000	0.915128000	6.821674000
H	5.819335000	0.282859000	5.373862000
H	4.864913000	0.563018000	6.827878000
H	3.241897000	2.158042000	5.542351000
H	4.151695000	1.563410000	4.148172000
H	3.929071000	3.296601000	4.373338000
H	6.511049000	3.619672000	4.210344000
H	6.625494000	1.878048000	3.968169000
H	7.556817000	2.657619000	5.262609000
C	10.780736000	0.620477000	7.375208000
C	13.640825000	1.382654000	6.519985000
C	11.357886000	1.344884000	4.528407000
C	11.453490000	6.397775000	6.413559000
C	10.213334000	4.698121000	4.281765000
C	13.266717000	4.681342000	4.680841000

H	11.782940000	1.218681000	10.420695000
H	12.862677000	2.392757000	11.176126000
H	12.915943000	2.146327000	9.427885000
H	13.319566000	4.565498000	8.566448000
H	13.612689000	4.924376000	10.276349000
H	12.960564000	6.174745000	9.213476000
C	8.100266000	5.108371000	11.186026000
H	8.176957000	2.284768000	11.512372000
H	9.702138000	1.682690000	12.167630000
H	9.229097000	1.278520000	10.513641000
H	9.339384000	7.189025000	9.496065000
H	11.095955000	7.357450000	9.395622000
H	10.283285000	7.447655000	10.964574000
H	10.929582000	0.764226000	8.449571000
H	11.039716000	-0.420193000	7.144410000
H	9.711455000	0.753824000	7.167785000
H	14.251862000	1.990196000	5.842654000
H	13.838776000	0.327950000	6.293356000
H	13.991193000	1.578376000	7.538768000
H	10.281631000	1.463756000	4.357166000
H	11.611088000	0.293280000	4.344856000
H	11.885720000	1.943589000	3.778986000
H	10.516853000	6.492331000	6.977800000
H	11.456685000	7.188929000	5.653665000
H	12.284589000	6.590066000	7.098478000
H	10.204489000	3.790393000	3.671011000
H	10.289062000	5.559766000	3.607081000
H	9.248252000	4.758739000	4.802031000
H	14.087632000	4.739757000	5.404950000
H	13.356148000	5.536850000	4.000493000
H	13.411553000	3.772563000	4.087140000
H	7.556755000	5.534624000	10.329126000
H	8.167543000	5.895985000	11.947453000
H	7.485226000	4.304420000	11.600857000

8 References

[1] A. M. Borys, *Organometallics* **2023**, *42*, 182–196.

[2] C. Ganesamoorthy, S. Loerke, C. Gemel, P. Jerabek, M. Winter, G. Frenking, R. A. Fischer, *Chem. Commun*. **2013**, *49*, 2858.

[3] D.-Y. Lee, J. F. Hartwig, *Org. Lett.* **2005**, *7*, 1169–1172.

[4] H. Bürger, W. Sawodny, U. Wannagat, *J. Organomet. Chem*. **1965**, *3*, 113–120.

[5] D. R. Armstrong, A. R. Kennedy, R. E. Mulvey, J. A. Parkinson, S. D. Robertson, *Chem. Sci*. **2012**, *3*, 2700.

[6] J. D. Gorden, A. Voigt, C. L. B. Macdonald, J. S. Silverman, A. H. Cowley, *J. Am. Chem. Soc.* **2000**, *122*, 950–951.

[7] F. Dankert, E. Hevia, *Chem. – Eur. J.* **2024**, 30, e202304336.

[8] G. M. Sheldrick, *Acta Crystallogr. A: Found. Adv*. **2015**, *71*, 3–8.

[9] G. M. Sheldrick, *Acta Crystallogr. C: Struct. Chem*. **2015**, *71*, 3–8.

[10] O. V. Dolomanov, L. J. Bourhis, R. J. Gildea, J. A. K. Howard, H. Puschmann, *J. Appl. Crystallogr*. **2009**, *42*, 339–341.

[11] X-AREA; integrated LANA; XRED32. Stoe & Cie: Darmstadt, Germany, **2020**.

[12] K. Brandenburg, DIAMOND, V. 4.6.8. **2022**.

[13] F. Neese, WIREs *Comput. Mol. Sci*. **2012**, *2*, 73–78.

[14] F. Neese, WIREs Comput. Mol. Sci. 2022, 12, e1606.

[15] S. Grimme, A. Hansen, S. Ehlert, J.-M. Mewes, *J. Chem. Phys*. **2021**, *154*, 64103.

[16] G. Henkelman, H. Jónsson, *J. Chem. Phys*. **2000**, *113*, 9978–9985.

[17] F. Weigend, R. Ahlrichs, *Phys. Chem. Chem. Phys*. **2005**, *7*, 3297.

[18] J. D. Rolfes, F. Neese, D. A. Pantazis, *J. Comput. Chem*. **2020**, *41*, 1842–1849.

[19] G. L. Stoychev, A. A. Auer, F. Neese, *J. Chem. Theory Comput.* **2017**, *13*, 554–562.

[20] A. V. Marenich, C. J. Cramer, D. G. Truhlar, *J. Phys. Chem. B* **2009**, 113, 6378–6396.

[21] G. Knizia, IBOView – A program for chemical analysis; see http://www.iboview.org/.

[22] G. Knizia, J. E. M. N. Klein, *Angew. Chem. Int. Ed*. **2015**, *54*, 5518–5522; G. Knizia, J. E. M. N. Klein, Angew. Chem. **2015**, *127*, 5609–5613.

[23] Chemcraft – graphical software for visualization of quantum chemistry computations. https://chemcraftprog.com.

[24] C. Adamo, V. Barone, *J. Chem. Phys*. **1999**, *110*, 6158–6170.

[25] E. Caldeweyher, C. Bannwarth, S. Grimme, *J. Chem. Phys*. **2017**, *147*, 034112.

[26] S. Grimme, J. Antony, S. Ehrlich, H. Krieg, *J. Chem. Phys*. **2010**, *132*, 154104.

[27] A. D. Becke, *J. Chem. Phys*. **1993**, *98*, 5648–5652.
